# Out of the net: An agent-based model to study human movements influence on local-scale malaria transmission

**DOI:** 10.1371/journal.pone.0193493

**Published:** 2018-03-06

**Authors:** Francesco Pizzitutti, William Pan, Beth Feingold, Ben Zaitchik, Carlos A. Álvarez, Carlos F. Mena

**Affiliations:** 1 Universidad San Francisco de Quito, Instituto de Geografía, Quito, Ecuador; 2 Duke University, Duke global Health Institute, Durham, NC, United States of America; 3 SUNY-Albany, School of Public Health, Rensselaer, Albany, NY, United States of America; 4 Johns Hopkins University, Morton K. Blaustein Department of Earth & Planetary Sciences, Baltimore, MD, United States of America; 5 DIRESA-Loreto Epidemiology Department, Iquitos, Loreto, Peru; Food and Drug Administration, UNITED STATES

## Abstract

Though malaria control initiatives have markedly reduced malaria prevalence in recent decades, global eradication is far from actuality. Recent studies show that environmental and social heterogeneities in low-transmission settings have an increased weight in shaping malaria micro-epidemiology. New integrated and more localized control strategies should be developed and tested. Here we present a set of agent-based models designed to study the influence of local scale human movements on local scale malaria transmission in a typical Amazon environment, where malaria is transmission is low and strongly connected with seasonal riverine flooding. The agent-based simulations show that the overall malaria incidence is essentially not influenced by local scale human movements. In contrast, the locations of malaria high risk spatial hotspots heavily depend on human movements because simulated malaria hotspots are mainly centered on farms, were laborers work during the day. The agent-based models are then used to test the effectiveness of two different malaria control strategies both designed to reduce local scale malaria incidence by targeting hotspots. The first control scenario consists in treat against mosquito bites people that, during the simulation, enter at least once inside hotspots revealed considering the actual sites where human individuals were infected. The second scenario involves the treatment of people entering in hotspots calculated assuming that the infection sites of every infected individual is located in the household where the individual lives. Simulations show that both considered scenarios perform better in controlling malaria than a randomized treatment, although targeting household hotspots shows slightly better performance.

## Introduction

The appearance and recurrence of malaria pathogens is a persistent threat for more than 91 countries and territories all over the world with implications for human health, economy and ecosystem integrity [1]. For this reason in the last decades many efforts were made to control malaria worldwide [1]. Control methods include distribution of Insecticide-Treated Bed Nets (ITNs), Indoor Residual Spraying (IRSs), diagnostic, distribution of anti-malaria treatments, and agricultural areas management [2]. These efforts often, but not always, produced the expected results: between 2000 and 2015 the global malaria incidence was reduced by 41%, mortality rate by 62%, and 17 previously malaria endemic countries attained 3 consecutive years of zero indigenous cases [1]. Furthermore, many previously high-malaria-transmission countries currently bear significantly lower malaria prevalence than in the past. However, it is common to observe malaria-reduction trend reversals, which are caused by multiple factors that increase malaria potential including climate change, wars, socio-economic weakening and migration [3]. Furthermore, when malaria incidence decreases in a given population, it is common for adherence to preventative measures to decrease as well [3]. Additionally when policy makers in developing countries foresee the possibility of malaria eradication, they may prematurely divert the limited amount of public resources dedicated to control malaria, to other areas of public health concern [3]. These practices leave endemic areas exposed to resurgence risks. On the other hand, it is clear that, in the areas where malaria reaches low levels of transmission, mass–campaigns are no longer efficient or effective, and thus optimizing resources dedicated to malaria control in areas of low and decreasing transmission is a relevant challenge. Lack of improved, evidence-based strategies tailoring when and where to concentrate control efforts at a global, regional and local scale remains an inadequately-solved barrier which must be overcome to make malaria elimination an actuality.

Malaria’s transmission process is inherently spatial: Infections occur in certain locations in space and the disease spreads when involved individuals, both hosts and vectors, move through a geographical space [4–5]. The geographical environment—meaning its geomorphological, biophysical and hydrological characteristics—presents heterogeneities that make the transmission spatially uneven. Especially in low-transmission conditions, environmentally-based hotspots of high malaria-transmission risk are generated in virtually all malaria-endemic contexts [6–7]. Environmentally-based hotspots of malaria have been observed in relation to proximity to forests fringes [8], aquatic habitats like swamps [9], rice fields [10–11] and marshes [12], among other ecological features. An analogous hotspot formation process is observed when the disease meets human-based heterogeneities. Several factors contribute to the creation of human-based hotspots, including poor housing design [13], genetic factors [14], differential coverage of prevention methods or anti-malaria treatments [15], and other socio-economics conditions [7]. Hotspot formation concentrates the disease’s burden on a small fraction of the exposed populations: from analysis of transmission rates an empirical relationship was observed suggesting that 20% of the exposed population contributes to at least 80% of the net transmission potential [16]. Malaria hotspots can be unstable [17], but also they can be stable over multiple years, and, since they are predictive of future malaria infections [18], they can be targeted to reduce malaria incidence [7]. Control programs should identify and target specific high-risk populations such as adults exposed to work-related infections or people living in transmission hotspots [19]. Developing and deploying validated malaria hotspot targeting interventions is now a top priority for optimizing controlling strategies in low-transmission settings [6].

Local scale malaria transmission modeling is a remarkable tool with broad capabilities for identifying and managing malaria hotspots. Modeling can help in understanding where, when and how hotspots are created, in testing the effect of specific, optimized control strategies and in guiding the implementation of field control strategies. Since every geographic area presents distinct patterns of environmentally and human-based heterogeneities and those heterogeneities strongly determine malaria hotspots formation, a malaria transmission model should be able to include detailed descriptions of environment, vector and host populations in order to capture those heterogeneities. For this reason models of malaria based on compartmental structures, in which individuals are categorized in homogeneous groups as in the Ross-McDonald models family, are not suitable [20]. A new class of models, Agent-Based Models (ABM), is emerging and permits accounting for much finer details of the individual-based and heterogeneous features of vector-borne diseases transmission [21]. ABMs are computer models that study complex epidemiological systems with computer simulations. Every individual is represented in ABMs explicitly as an agent. Agents interact among each other and with an environment creating the complex network of interactions and feedbacks typical of infectious diseases transmission. Trough ABMs it is possible not only to study the influence of the environment and socio-economics conditions on malaria hotspots formation but it is also possible analyze the influence of human movements on hotspots formation. Malaria hotspots form in geographic areas which promote a higher-than-normal malaria infectious biting rate and in many circumstances human local scale movements may have a strong influence in determining when and where human-mosquito contacts occur. However, starting from the observation that most anopheline species have nocturnal biting habits, the majority of published ABM studies on malaria transmission [22–24], represent humans as located within households during the entire simulations length. In such a configuration, humans do not move from their homes and their protection against mosquito bites is maintained constantly high because in most malaria-endemic ITNs are assumed to be widespread. In contrast in low-transmission areas a more complex pattern of malaria transmission epidemiology is emerging [25]. The proportion of adult men among all infected people is increasing, and malaria burden appears to be increasingly connected with work-related activities [26–27]. Therefore, in low-transmission settings it appears particularly appropriate not to limit the representation of malaria transmission only to periods when humans are protected under ITNs, because transmission during normal daily human activity can be significant. Several studies showed that anopheline mosquitoes’ biting hours do not overlap exactly with human sleeping hours [28–29]. In tropical latitudes, sunrise is at approximately 6:00 am and sunset is at approximately 6:00 pm. Humans carry out important activities around 6:00 am: people wake up, eat, prepare to the day, travel to go to work among other actions. Most social and recreational activities are concentrated after 6pm: people move outdoor, usually on foot, to shop, meet, collect water, cook meals and, depending on age, commonly do not retire to bed until after 8:00–9:00 pm. Moreover, some working activities overlap partially with vectors’ biting hours, fueling the observed increasing proportion of adult men among overall malaria cases [26–30]. The overlap of mosquito biting hours and human daily activities is accentuated by the appearance of behavioral avoidances that mosquitoes develop to adapt to a human environment dominated by ITNs and IRSs [31–35].

In order to study the influence of human movement on the efficacy of a targeted malaria control efforts inside hotspots, two sets of ABMs local scale malaria transmission scenarios representing two distinct communities of the Peruvian Amazon were developed. Across Peru in the 20^th^ century, malaria incidence fluctuated between periods of near pre-eradication to periods of large-scale outbreaks, mainly afflicting the Amazon departments. The two most notable outbreaks of the 20^th^ century struck Peru 60 years apart: the first began in Cuzco department in the 1930s and the second in the Loreto department in the mid-1990s [36]. Since 2012, evidence of an emerging serious outbreak in Loreto department has started to appear [37]. The ABMs presented here reproduce mosquito vectors and human hosts at an individual-based level. The models are also composed by a spatially explicit representation of the geographical space where the communities are located. Human agents move through the simulation area following an individualized time schedule that reproduces daily activities typical of people living in Amazon communities. During a simulation day, human agents pass through various environments and change their protection status against mosquito bites, resulting in different Entomological Inoculation Rates (EIR). To study the effect of human movement on malaria transmission, for every community we ran and compared two “what if” movement-testing scenarios. The first “what if” scenario corresponded to a model of human movement representing, as closely as possible, real world human daily displacements within the community. In this baseline scenario every activity corresponded to a specific protection level against mosquito bites. In the second “what if” scenario, humans did not move from their respective households, but they did change protection state against mosquito bites depending on the hour of the day just as in the first “Human movement” scenario. The baseline scenario is then used to study the efficacy of malaria control strategies designed to target hotspots. In this respect, we note that the position and size of malaria hotspots depend strictly on the geographical distribution of infection sites where the transmission occurred between an infected mosquito and a susceptible human host. When malaria is surveyed in the field it is practically impossible to know precisely where and when a mosquito-human transmission event happened. For this reason, it is usually assumed that the infection site corresponds to the household position of the infected individual and the time of exposure is the moment when the individual is registered as a confirmed case at the health facility. Therefore usually, the information about the exact infection site is lost. Clearly the geographical hotspots, which are malaria hotspots calculated considering the exact sites of infections, portray more precisely the uneven geographical distribution of malaria risk than household hotspots calculated considering households as infection sites. At first glance then, it could be logical to think that the best control strategy could be treating people entering inside geographical hotspots. On the other hand, it is also true that people exposed to infection inside geographical hotspots, spend part of their time in their respective households increasing the infection risk of people living with them and also of people living in the household’s surrounding area. Additionally, both the geographical and household hotspots capture the exposure heterogeneities deriving from specific households-level environmental exposures. To compare the efficacy of control strategies targeting geographical and household hotspots we considered a second set of “what-if” control-testing scenarios. The first control-testing scenario consists in considering ABMs where individuals that spend part of their time inside geographical hotspots are protected against malaria, while the second tested strategy consists in protect individuals that inhabit households that are included inside household hotspots.

## Methods

### Short models description

**Fig 1:**
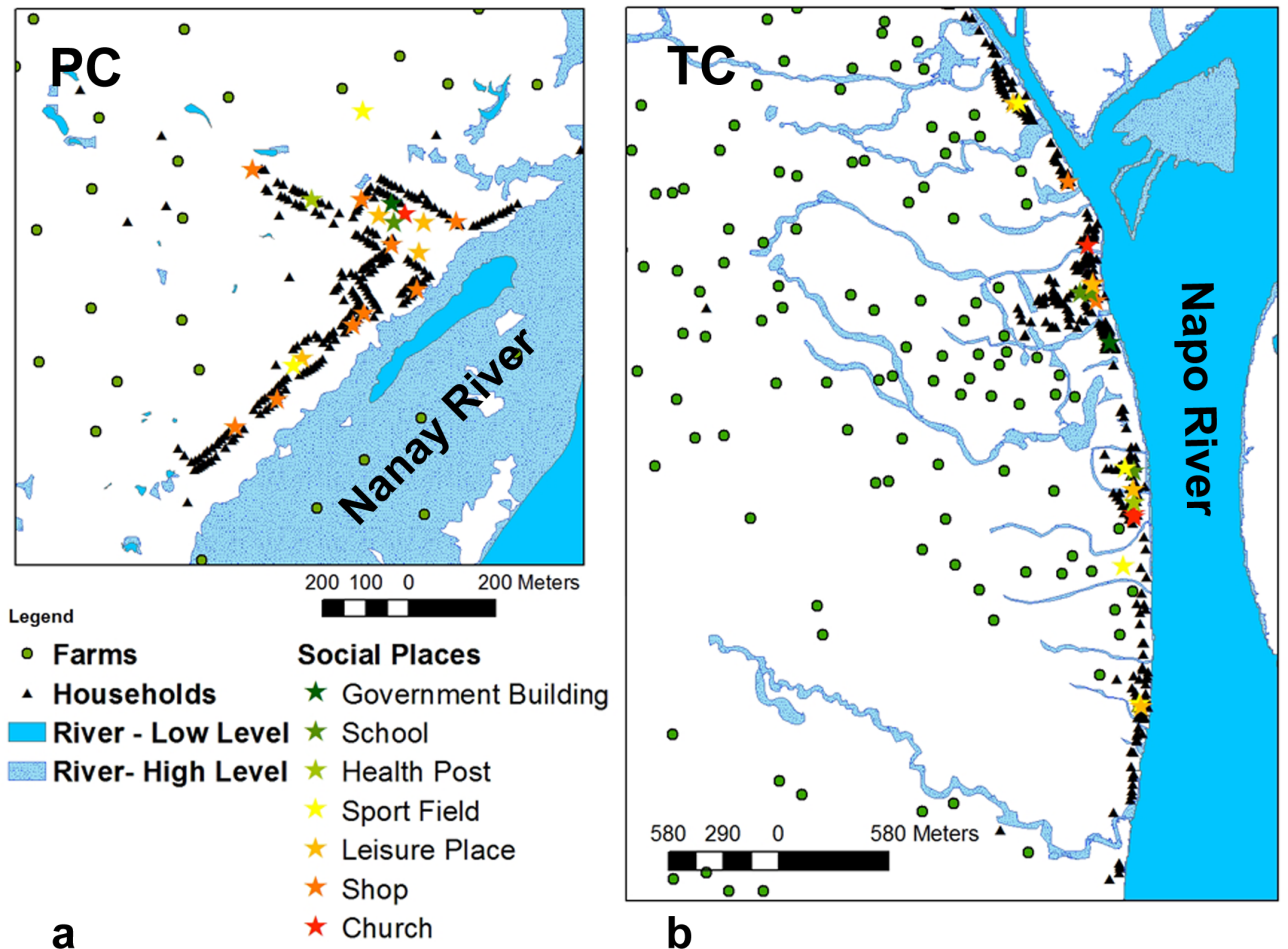
The ABMs study areas centered on the communities of Padre Cocha (PC) (a) and San Luis de Tacsha Curaray (TC) (b).

The ABMs presented in this paper are dedicated to the study of malaria transmission in the environment of two small riverine communities, Padre Cocha (PC) and San Luis de Tacsha Curaray (TC), located in the Peruvian Amazon (see Fig 1). The dominant environmental characteristics of this area of low malaria transmission is periodic river flooding, which yields mosquito breeding sites. The geographical space around the two communities is represented in the ABMs explicitly, including an elevation model, a land cover model, and a meteorological model. The ABMs also include a hydrological module that converts river levels into corresponding extensions of flooded areas around the communities. Flooded areas and other stagnant water bodies are considered, in the ABMs, suitable breeding sites for mosquito agents, connecting seasonal river flooding with malaria incidence. Three types of agents are included in the model: *Plasmodia*, mosquitoes and humans agents. The considered species of *Plasmodia* agents are those observed in the study areas: *P*. *vivax* and *P*. *falciparum* [36]. Mosquito agents are parameterized to reproduce the characteristics of *Anopheles darlingi*, the almost-exclusive vector of malaria in the study areas [38]. Mosquitoes move through the simulation areas with the objective of attaining a blood meal from either animals near human settlements or from humans. After that they search for suitable aquatic habitats to lay eggs and then they repeat the cycles from blood meal to oviposition until death. Larval development is not represented explicitly in the model [24]. Simulated mosquito agents do not have memory about already visited sites, but they are sensitive to the environment they go through and they are attracted by simulation pixels containing human agents or breeding sites. Humans are represented as genetically homogeneous individuals. Several “what-if” scenarios are presented in this paper in which human agents’ behaviors are depicted differently. A common feature shared by all scenarios is that, while sleeping at home, a high fraction of human agents is protected by ITNs. ITNs not only protect humans but also kill mosquito agents. When human agents are not sleeping at home, human behaviors and infection risks depend on the considered scenario. Two groups of scenarios are presented here: the first group, called “movement-testing” scenarios, is designed to study the effect of human movements on malaria transmission while the second group, called “control-testing” scenarios is aimed to study the effectiveness of control interventions when malaria hotspots are targeted. One movement-testing scenario considered is the “**Human Movement**” scenario, or **baseline scenario**, where humans move freely during waking hours carrying out several activities like work, school, sport, etc.. During diurnal activities humans are exposed to mosquito bites depending on the specific activity they are carrying out. During night hours a fraction of human agents are protected by ITNs. The second movement-testing scenario considered is the “**No Human Movement**” scenario, in which human agents behave exactly as in the “Human Movement” scenario with the exception that they never move from the positions of their respective households. A second set of “what-if” scenarios, created as variation of the baseline scenario, is then considered to study the effect on malaria transmission of protecting specific groups of human agents. The definition of the target groups of human agents follows three different strategies. The first control strategy considers the protection of an increasing fraction of human agents selected at random inside the human population. The second control strategy considers the protection of an escalating proportion of the human agents that, at any time during the simulation, enter inside a malaria hotspots when malaria hotspots are calculated considering the actual infection sites (**geographical hotspots**). The third set of control-testing scenarios considers as constantly protected an escalating fraction of humans entering in a malaria hotspots when the malaria hotspots are calculated locating the human malaria cases in the household locations of infected individuals (**households hotspots**).

Environmental, entomological and *Plasmodia* modules, describing how agents behave during the simulations are implemented in the ABMs as shown by Pizzitutti et al. [24]. A resume of parameter sets used in the ABMs modules is shown in Tables 1, 2 and 3 for both PC and TC simulations. The human module is implemented as in [24], excluding the human movement component described in detail here in subsection “Human module”.

**Table 1:** Environmental module parameters.

Environmental Module			
Parameter name	PC	TC	Notes
**Simulation time step**	1 hour	1 hour	
**Study area extension**	2370 x 2520 m	10252 x 4200 m	
**Centroid of the study area bounding box**	3°41'54.17"S, 73°16'43.24"W	2°48'15.83"S, 73°32'35.23"W	
**Geographical grid cover pixel size**	10 m	10 m	
**Mosquito grid cover pixel size**	30 m	30 m	
**Geographical grid extension**	237 x 252 pixels	398 x 971 pixels	
**Mosquito grid extension**	79 x 84 pixels	131 x 324 pixels	
**Number of mosquito agents generated per oviposition**	13.5 mosquito agents/oviposition	7.0 mosquito agents/oviposition	calibration parameter
**Maximum number of mosquito agents generated per unit of breeding area every 12 simulation hours**	0.30 mosquito agents/m2/12 h	1.43 mosquito agents/m2/12h	calibration parameter
**Reduction factor of the number of mosquitoes generated per unit of breeding area per unit of time in permanent water covers**	1.5	14.0	calibration parameter

**Table 2:** Entomological module parameters.

Entomological Module (PC & TC)		
Parameter name	Value	Notes and references
Aquatic stage development time	15 days	[39]
Random walk pixels weight	0.85	**host-seeking** mode calibration parameter
Random walk pixels weight	0.85	**blood-seeking** mode calibration parameter
Biting hours	From 6pm to 6 am	[28]
Probability to fail the bite when human is sleeping	0–1	0 when the human agent is protected against malaria 1 when the human agent is not protected against malaria
Resting time after blood meal	4 hours	[40]
Time required for eggs maturation after the blood meal	48 hours	[39]
Daily survival probability function	$p_{s}=e^{-\frac{1}{-4.4+1.3∙T-0.03∙T^{2}}}$	[41], T is the temperature
Survival probability factor during a rainy day	0.7	calibration parameter
Minimum rain of a rainy day	100 mm /day	calibration parameter
Probability of having a blood meal from domestic animals	TC: 0.42, PC: 0.88	calibration parameter

**Table 3:** Plasmodia module parameters.

Plasmodia Module (PC & TC)			
Parameter name	*P*. *falciparum*	*P*. *vivax*	Notes and references
T_min_ extrinsic incubation	16°C	14.5°C	[42], minimum temperature of incubation in mosquitoes
DD extrinsic incubation	111°C DD	105°C DD	[42], number of degree days to complete the sporozoites development in mosquitoes
Intrinsic incubation time	(9–14) days	(12–17) days	[43–44], (min—max) range. Actual value extracted as uniformly distributed between min and max
Transmission efficiency from asymptomatic human to mosquito	0.1	0.1	[45]
Transmission efficiency from human to mosquito	0.4	0.4	[46]
Transmission efficiency from mosquito to human	1	1	The value is chosen to maximize the number of infectious bites and reduce the simulations computational weight
Human infectious period if treated	300 hours	24 hours	[47], calibration parameter only for *P*. *falciparum*
Recurrence time		203 days	[48], only for *P*. *vivax*
Recurrence risk		0.3	[48], only for *P*. *vivax*
Gametocytemia starting time	(10–14) days	(9–13) days	[47–49–50], (min—max) range. Actual value extracted as uniformly distributed between min and max

### Process overview and scheduling

The models’ time step is 1 hour. Every 12 hours mosquito agents emerge as adult mosquitoes from aquatic habitats where eggs were previously laid by other mosquito agents. A simulation geographical pixel is considered suitable as aquatic habitat for mosquitoes if it is covered by water, but also if it is not in the middle of a river where the flowing water would wash away the eggs. Eggs hatch into adult mosquitoes if the water cover persists for more than the time needed for eggs to mature (Table 1, Entomological Module Parameters). The mosquito agents attempt to find blood meals and subsequently to lay eggs. To achieve these goals the mosquitoes move through the simulation area from one pixel to an adjacent one every time step following an algorithm described in detail by Pizzitutti et al. (Pizzitutti et al., 2015). The Plasmodium agents are represented as living inside the bodies of human and mosquito agents, passing through several stages of development that correspond to different stages of infection of both vectors and hosts (see [24] for details). The simulation area water cover changes according to the river level registered on the date corresponding to the simulation time: higher river levels flood more extended areas. The algorithm used by the model to simulate the rivers’ floods is described by Pizzitutti et al. in [24]. The “No Human Movement” model scenarios represent humans that are statically assigned to their household position. While in the baseline “Human Movement” scenario, every simulation day, a new 24 hour daily schedule is created for every human agent. During the 24 hours of a simulation day, human agents change their positions every time step following the assigned daily schedule.

### Study areas

The ABM study areas are defined by two rectangular bounding boxes centered on the communities of PC and TC, both located in the Loreto Department of Peru (see Fig 1). PC [3°41′54.17″S, 73°16′43.24″W] is situated close to the river Nanay, 5.5 km north-east of the city of Iquitos, the capital of the Loreto district. TC [2°48'15.83"S, 73°32'35.23"W] is positioned on the shore of the Napo River, 100 km north of Iquitos. Both communities are surrounded by cleared agricultural areas and vast tropical rain forest patches (both primary forest and secondary regrowth). Much of the land around the communities is subjected to periodic river flooding. The main differences between the PC and TC are the registered malaria incidence and the spatial configuration of community buildings. TC is stretched along the river, with few houses located more than 100 m far from the river banks, and overall has low malaria incidence during the study period. In contrast, PC has a well-defined village center, with houses both close to the river and houses as far as 700 m from the river. PC, during the study period, had relatively high malaria incidence. Malaria is endemic in both communities and is transmitted almost exclusively by *Anopheles darlingi* [38]. The simulation area around PC is 2370 m wide and 2520 m long while the bounding box defining the simulation area around TC is 10252 m wide and 4200 m long. The ABMs included on average 2093 human agents distributed in 349 households in TC simulations while the PC simulations included an average number of 1400 human agents distributed in 244 households.

### Inputs to the models

Meteorological data in PC are obtained from government meteorological station number 843770 (SPQT) located near the Iquitos airport, approximately 9500 m from PC. Meteorological data for TC are obtained from the “Servicio Nacional de Meteorología e Hidrología de Perú” (SENAMHI—Governmental Peruvian Institute of Meteorology and Hydrography), meteorological station 000177 located in Santa Clotilde [2°29'14.9''S, 73°40'45.2''W], approximately 37 km north of TC. As described by Pizzitutti et al. (2015), the ABM’s hydrological module converts river levels into corresponding water covers over the study areas. The inputs to the hydrological module are: the river levels time series, a study area elevation model and two high resolution satellite images per community taken in correspondence to the high and to the low water levels. The Nanay river levels were obtained from the paper of Bautista et al. [51] while the Napo river levels are obtained from the SENAMHI hydrological station 240111 [3°28' 55.4''S, 73°4'24.6''W], located in Belllavista 91 Km South from TC. Elevation models for both communities are from processed Shuttle Radar Topography Mission (SRTM) [52]. The high resolution satellite images of PC were acquired from Google Earth, while the images of TC are from satellite Rapid Eye (low river level) and satellite SPOT6 (high river level). Malaria counts used to calibrate the models are epidemiological data released by the DIRESA-Loreto (Direccion Regional de Salud, Loreto–Regional Health Directorate of Loreto). Data used to model human daily activities are adapted from the paper of Chuquiyauri et al [26] and from field surveys data [53].

### Human behavior module

Every human agent is assigned randomly to a village house and the number of humans assigned to a house is determined by a Gaussian distribution as specified in Table 4. The minimum number of people assigned to a household is 1. A certain fraction of human agents are represented as asymptomatic. All the asymptomatic individuals are able to transmit the pathogen to mosquitoes biting them (no transmission blocking) (see Table 4). No human or mosquito superinfection is considered in the models. As usual in the Peruvian Amazon, no seasonal malaria chemoprevention is included in the model.

**Table 4:** Human module parameters.

Parameter name	PC	TC	Notes
Average number of human agents	1400	2093	
Fraction of human agents protected against mosquitoes bites while sleeping	0.84 ^(*)^	0.89	^(*)^Average fraction of protected agents. See [24] for details.
Parameters of the Gaussian distribution of the number of human agents assigned to every house	mean = 6 σ = 3	mean = 6 σ = 3	
Fraction of asymptomatic human agents	0.07	0.04	*P*. *falciparum*, *calibration parameter*
Fraction of asymptomatic human agents	0.05	0.07	*P*. *vivax*, *calibration parameter*

Following the census population distribution data shown in Table 5, published by the INEI (Instituto Nacional de Estadística e Informática—Peruvian National Institute of Statistics and Informatics) [54], every human agent is assigned an age in the ABM simulation.

**Table 5:** Loreto population age structure.

Age Segment (years)	
**0–5**	17%
**6–12**	17%
**13–17**	12%
**18–24**	13%
**25–39**	20%
**40–55**	13%
**> 55**	75%

2007 country-wide census, INEI (www.inei.gob.pe).

Then human agents are separated in age segments [54], and every age segment is associated to a number of ideal number of sleeping hours (Table 6).

**Table 6:** Human agents age segments.

Age segment	Age min	Age max	Number sleep hours
**child**	0	12	11
**young**	13	24	8
**adult**	25	49	8
**senior**	50	69	8
**retired**	70	-	7

Sleep durations are from ref.: [55]

The humans belonging to young, adult and senior age segments are “workers”. To each worker is assigned a working activity. Then, based on the parameters shown in Table 7, working hours are filled in individuals’ daily schedules. To every worker agent is assigned a work location inside or outside the simulation area. Farmers are placed on designated farms. The positions of farms are determined assigning a farm location to every cleared plot around the community in the simulation area. Due to constant rainfalls and stable temperatures, in the Amazon there are no agricultural seasons as in others areas of the world. The farmers can use their land, when not flooded, continuously all over the year. Craftsmen and “others” are assigned to a workshop in the village. Office workers are assigned to health posts, government buildings and shop keepers to shops, etc. Unemployed workers do not have a defined working place, but during the working hours these agents move randomly in an area close to the community houses. “Homemaker” laborer are assigned their respective houses as working locations.

**Table 7:** Human work parameters.

Work	From	To	Time per week	Days	Max. hours per day	Working population fraction
**farmer**	5:00	20:00	6	1 2 3 4 5 6	10	0.12
**office**	8:00	18:00	5	1 2 3 4 5	8	0.01
**shop keeper**	6:00	23:00	7	1 2 3 4 5 6 7	12	0.03
**transport**	0:00	23:00	6	1 2 3 4 5 6 7	10	0.1
**other**	7:00	19:00	6	1 2 3 4 5 6	10	0.1
**homemaker**	6:00	20:00	6	1 2 3 4 5 6	10	0.31
**craftsman**	7:00	19:00	6	1 2 3 4 5 6	10	0.03
**unemployed**	8:00	20:00	6	1 2 3 4 5 6	10	0.05

Data from ref. [26], and from personal communications from J. Lana and B. Pan authors of the study in ref. [53]

Following the respective priorities, the remaining 24 hours of the day are filled with sleeping and with the additional non-work activities (Table 8). The column ‘priority’ in Table 8 indicates the importance that is given to every activity when the time schedule of each agent have to be filled. High priority values indicates important activities that will be added to the schedule before low priority value activities. For workers, the work activity scores always the highest value.

**Table 8:** Additional human agent daily activities parameters.

**Child**	**From**	**To**	**Time per week**		**Days**	**Max. hours per day**	**Priority**
**Sport Field**	16:00	21:00		7	1 2 3 4 5 6 7	4	4
**Leisure Place**	16:00	21:00		7	1 2 3 4 5 6 7	4	4
**Shop**	7:00	21:00		7	1 2 3 4 5 6 7	1	3
**Church**	18:00	19:00		1	7	1	5
**Governmental**	7:00	18:00		1	1 2 3 4 5	1	6
**Health Post**	8:00	14:00		1	1 2 3 4 5	1	3
**School**	7:00	12:00		6	1 2 3 4 5 6	6	1
**Sleep**	19:00	6:00		7	1 2 3 4 5 6 7	16	2
**Young**	**From**	**To**		**Time per week**	**Days**	**Max. hours per day**	**Priority**
**Sport Field**	16:00	22:00		6	1 2 3 4 5 6 7	4	3
**Leisure Place**	16:00	22:00		6	1 2 3 4 5 6 7	4	3
**Shop**	7:00	22:00		5	1 2 3 4 5 6 7	1	3
**Church**	18:00	19:00		1	7	1	4
**Governmental**	7:00	18:00		1	1 2 3 4 5	1	5
**Health Post**	8:00	14:00		1	1 2 3 4 5	1	2
**Sleep**	22:00	6:00		7	1 2 3 4 5 6 7	12	1
**Adult**	**From**	**To**		**Time per week**	**Days**	**Max. hours per day**	**Priority**
**Sport Field**	16:00	22:00		5	1 2 3 4 5 6 7	4	3
**Leisure Place**	16:00	22:00		5	1 2 3 4 5 6 7	4	3
**Shop**	7:00	22:00		7	1 2 3 4 5 6 7	1	3
**Church**	18:00	19:00		1	7	1	3
**Governmental**	7:00	18:00		1	1 2 3 4 5	1	3
**Health Post**	8:00	14:00		1	1 2 3 4 5	1	2
**Sleep**	22:00	6:00		7	1 2 3 4 5 6 7	12	1
**Senior**	**From**	**To**		**Time per week**	**Days**	**Max. hours per day**	**Priority**
**Sport Field**	16:00	22:00		5	1 2 3 4 5 6 7	4	3
**Leisure Place**	16:00	22:00		5	1 2 3 4 5 6 7	4	3
**Shop**	7:00	22:00		7	1 2 3 4 5 6 7	1	2
**Church**	18:00	19:00		1	7	1	2
**Governmental**	7:00	18:00		1	1 2 3 4 5	1	2
**Health Post**	8:00	14:00		1	1 2 3 4 5	1	2
**Sleep**	22:00	6:00		7	1 2 3 4 5 6 7	12	1
**Retired**	**From**	**To**		**Time per week**	**Days**	**Max. hours per day**	**Priority**
**Leisure Place**	16:00	22:00		7	1 2 3 4 5 6 7	6	3
**Shop**	7:00	22:00		7	1 2 3 4 5 6 7	2	2
**Church**	18:00	19:00		1	7	1	1
**Governmental**	7:00	18:00		1	1 2 3 4 5	1	2
**Health Post**	8:00	14:00		1	1 2 3 4 5	1	2
**Sleep**	22:00	6:00		7	1 2 3 4 5 6 7	12	1

Data from ref. [26], and from personal communications from J. Lana and B. Pan, authors of the study in ref. [53]

Twenty-four hour simulation day schedules are generated in this way for each individual human agent. Following their assigned schedule, the humans change location depending on the locations of assigned activities. Additionally, human agents change protection state against mosquito bites depending on location and activity, as specified in Table 9.

**Table 9:** Dependence on location of protection against mosquitoes bites.

Location–activity	Protection against mosquitoes bites
**Household–sleeping**	0 for the fraction of human agents sleeping under a bed net 1 for the fraction of human agents not sleeping under a bed net
**Inside building of the community–not sleeping**	0.7
**Inside office**	0.6
**Farm—farming**	1

The protection against mosquitoes bite is expressed as the probability of been bitten when a mosquito agent attempt to get a blood meal from the human agent. The fraction of human agents sleeping under a bed net is different for the ABMs of the two considered communities and is specified by the parameter: “Fraction of human agents protected against mosquitoes bites while sleeping” (see Table 4).

### Models calibration and validation

The baseline “Human Movement” scenarios for both communities were calibrated in order to be validated against observed malaria incidence time series. For PC the study period starts from the beginning of 1996 to the end of 1998, while the TC simulations were calibrated against malaria epidemiological data starting from the beginning of 2011 to the end of 2012. For both ABMs a calibration vector was composed with the simulation parameters indicated as “calibration parameter” in Tables 1, 2 and 3 [56]. A calibration vector is a vector whose elements are model’s parameters whose values have to be tuned in order to achieve the validation [24]. At the beginning of the calibration process, 10 random calibration vector values were generated inside reasonable values ranges. Then 15 independent runs were simulated for each calibration vector value. The fitness of every calibration model was then evaluated in term of a score function, being the score function, the square distance between the time series of the observed malaria incidence and the simulated incidence time series obtained as average over the 15 independent outputs. The calibration vector values corresponding to the 3 most performing or “fitting” models were then mutated changing the values of some vector elements or alternatively they were recombined among them creating new calibration vectors as recombination products. Then the simulations corresponding to the newly created calibration vectors were run 15 times more and the process was repeated until the performance of new generations of models is no more increasing. Once the model was calibrated, 150 independent runs were then simulated. The data presented in the “Results” section are the product of these averages over these 150 simulations. The number of 150 repetitions was chosen to get the statistical error associated to the averages below 5%. Every simulation run of both calibration and production is preceded by a 365 days equilibration period.

The model have been implemented in the MASON [57] environment. MASON is a free, Java-based, discrete-event, multi-agent simulation library core used to reduce the repetitive code writing effort necessary to develop an ABM.

### Spatial analysis

Once the model was validated the simulation outputs were analyzed to identify hotspot positions inside the simulation areas. To ease the comparison with other malaria local transmission studies, in this paper the SaTScan v9.4.4 [58] software was used to spatially analyze the outputs and produce maps of high malaria transmission clusters or malaria hotspots. Several studies have employed SaTScan to evidence the formation of stable and unstable malaria hotspots [10–17–18–59]. SaTScan is a software package, based on the statistical method developed by Kulldorff [60], where multiple circular windows around infection sites in a geographical space are built. For ABMs scenarios where positions of humans are kept constantly located at their houses, the considered sites of infections are human houses, while for scenarios with moving humans the sites of infection are the actual pixels of the simulation where mosquito-human transmission occur. In the first case the reference population is the number of human agents resident in every household while in the second case the reference population is the normalized number of people that have spent some time in the considered pixel. SaTScan software changes the circular windows size around each populated site. Then for each considered circle, the number of observed malaria cases and the number of expected malaria cases are compared. A likelihood ratio test is calculated to compare the malaria incidence inside and outside the circle permitting the identification of hotspots as circles of higher than expected incidence. The hotspots shown in this paper here are statistically significant high risk clusters (p < 0.05) calculated by SaTScan through a purely Poisson spatial analysis including in the considered circles a maximum of 50% of the simulations human population.

## Results

### Movement-testing scenarios

**Fig 2:**
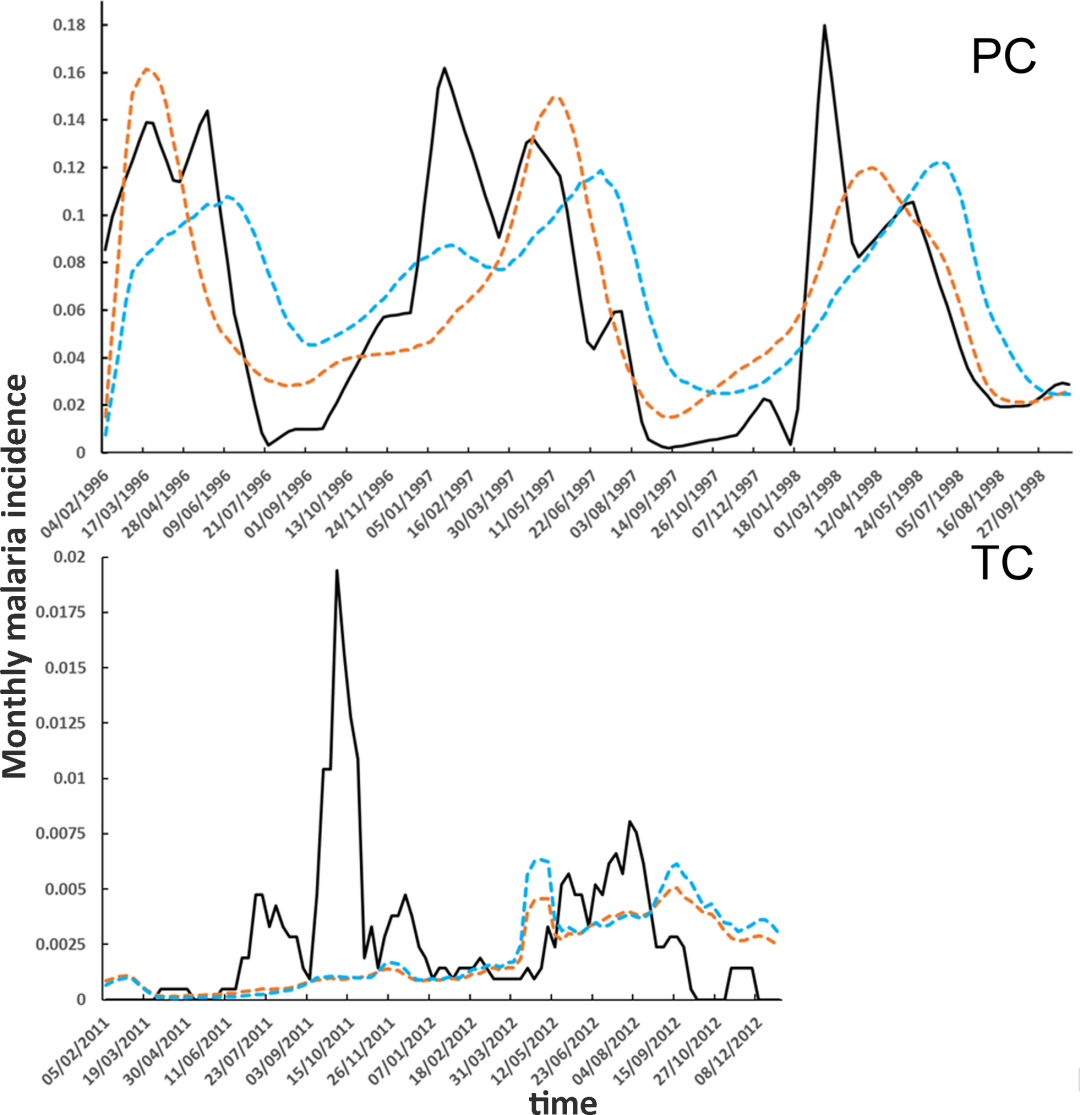
Simulated malaria incidences. Solid black line: observed incidence; dashed brown line: “Human Movement scenario”, dashed blue line: “No Human Movement” scenario. PC: Padre Cocha, TC: Tacsha Curaray.

The baseline “Human Movement” scenario was designed to accurately represent daily human activities. Alternatively, the “No Human Movement” scenario was conceived as a variation of the baseline scenarios to study the effects of human movement on malaria transmission. The main interest in the “No Human Movement” scenario was to compare a realistic representation of human movements with an unrealistic representation, which has been extensively used in the past to represent humans in malaria transmission ABMs. As shown in Fig 2 the PC simulations study period corresponds to the mid 90’s Loreto malaria outbreak while the TC simulations study period corresponds to the descendent tail of the same 90’s outbreak [36]. For that reason the incidence in PC is considerably higher than in TC. Also the periodic effect of river flooding is more accentuated in PC as shown by the pronounced periodic incidence peaks. The temporal behavior of simulated malaria monthly incidences, shown in Fig 2, reflects the evolution of corresponding observed malaria incidences with the exception of some evident differences. First of all, we note that the simulated curves are smoother than the observed ones. This outcome is expected since the simulated curves are the product of 150 independent runs of the same model. On the other hand the observed incidence curves are built considering the malaria cases registered at the local health posts without the intervention of any averaging process. Since, just like the output of a single simulation, the observed series of malaria incidence can be regarded as a single realization of a highly stochastic process the statistical fluctuation are large relatively to the average values. This explains why the observed time series present fluctuations that produce for instance spikes like the high peak of malaria incidence in TC in June 2011. Additionally we want to note that a model will always reproduce approximatively what observed in the real world. This is true especially with models like the ones presented in this paper that are not forecasting models but they are focused on mechanism reproduction and scenario planning.

A comparison between the simulated incidence curves of “Human Movement” and “No Human Movement” scenarios show that the movement of human agents through the simulation area does not seem to produce major effects on malaria incidence in both PC and TC.

Even if the overall malaria incidence is not affected by human movement, human movement changes the malaria risk levels to which certain categories of human agents, like the farmers, are exposed. When a farmer agent does not move from its home it is exposed to EIRs that are in average lower than those, the same agent, experiments when it spends part of its daily time working in the farm. The farmers, when movement is considered (see Table 10), are the group of agents that show the highest change in relative malaria incidence, as observed in many epidemiological studies in Loreto [26],. On the other hand, as shown in Table 9, close to the households in the communities, due to IRSs the EIR is lowered respect to the place where the farms are located. The group of people that shows the smaller relative malaria incidence is the group of children due to the more extended sleeping time under bed nets.

**Table 10:** Simulated relative malaria incidence as calculated inside specific groups of human agents.

	PC		TC	
Agent Type	Human Movement	No Human Movement	Human Movement	No Human Movement
**farmer**	0.15	0.08	0.0029	0.0018
**shop keeper**	0.11	0.08	0.0010	0.0024
**other manual**	0.08	0.08	0.0017	0.0015
**other**	0.08	0.08	0.0021	0.0016
**craftsman**	0.07	0.08	0.0022	0.0018
**unemployed**	0.07	0.08	0.0015	0.0019
**office**	0.06	0.08	0.0020	0.0020
**homemaker**	0.06	0.07	0.0018	0.0018
**transport**	0.06	0.07	0.0018	0.0020
**child**	0.05	0.06	0.0008	0.0012

**Fig 3:**
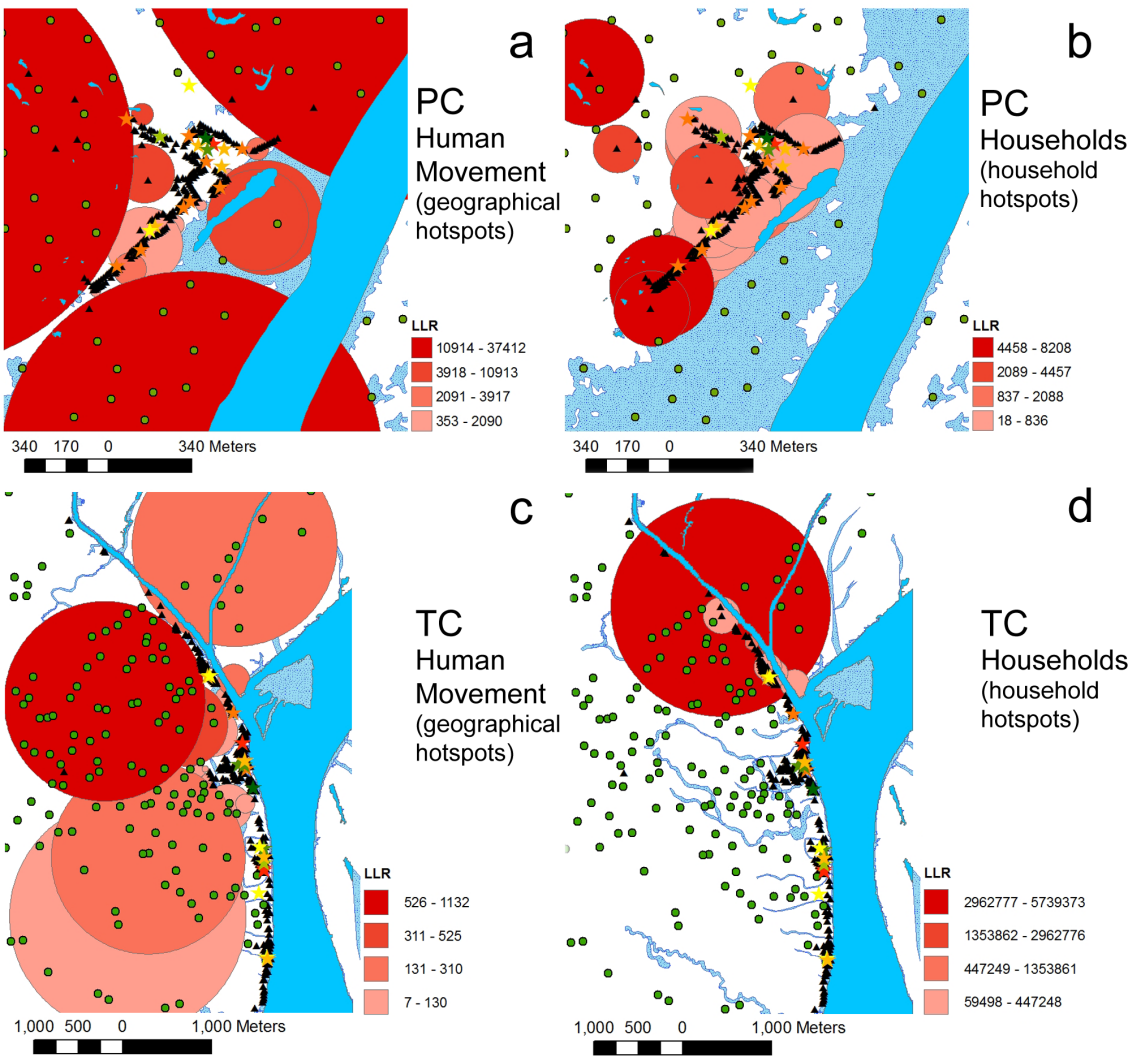
Malaria hotspots. Malaria hotspots revealed by the baseline “Human Movement” scenario. **a** and **c**: hotspots calculated locating human malaria cases in the actual place of infection. **b** and **d**: hotspots calculated locating the human malaria cases in the households of infected individuals. PC: Padre Cocha C, TC: Tacsha Curaray. LLR: log-likelihood ratio.

An additional difference between the movement-testing scenarios is the spatial distribution of high risk malaria clusters. When the SaTScan spatial analysis is considered for the “Human Movement” scenario, big primary and secondary hotspots both for PC and SC are observed in the areas where farms are located (Fig 3, panes **a** and **c**). On the other hand since in the “No Human Movements” scenario all infections occur in the location of human households, this scenario produces only hotspots centered on household positions (hotspots not shown). We note that, following both simulated scenarios, as observed by Bautista et al. [51], the village center of PC is a place of relatively low transmission risk.

### Control-testing scenarios

**Fig 4:**
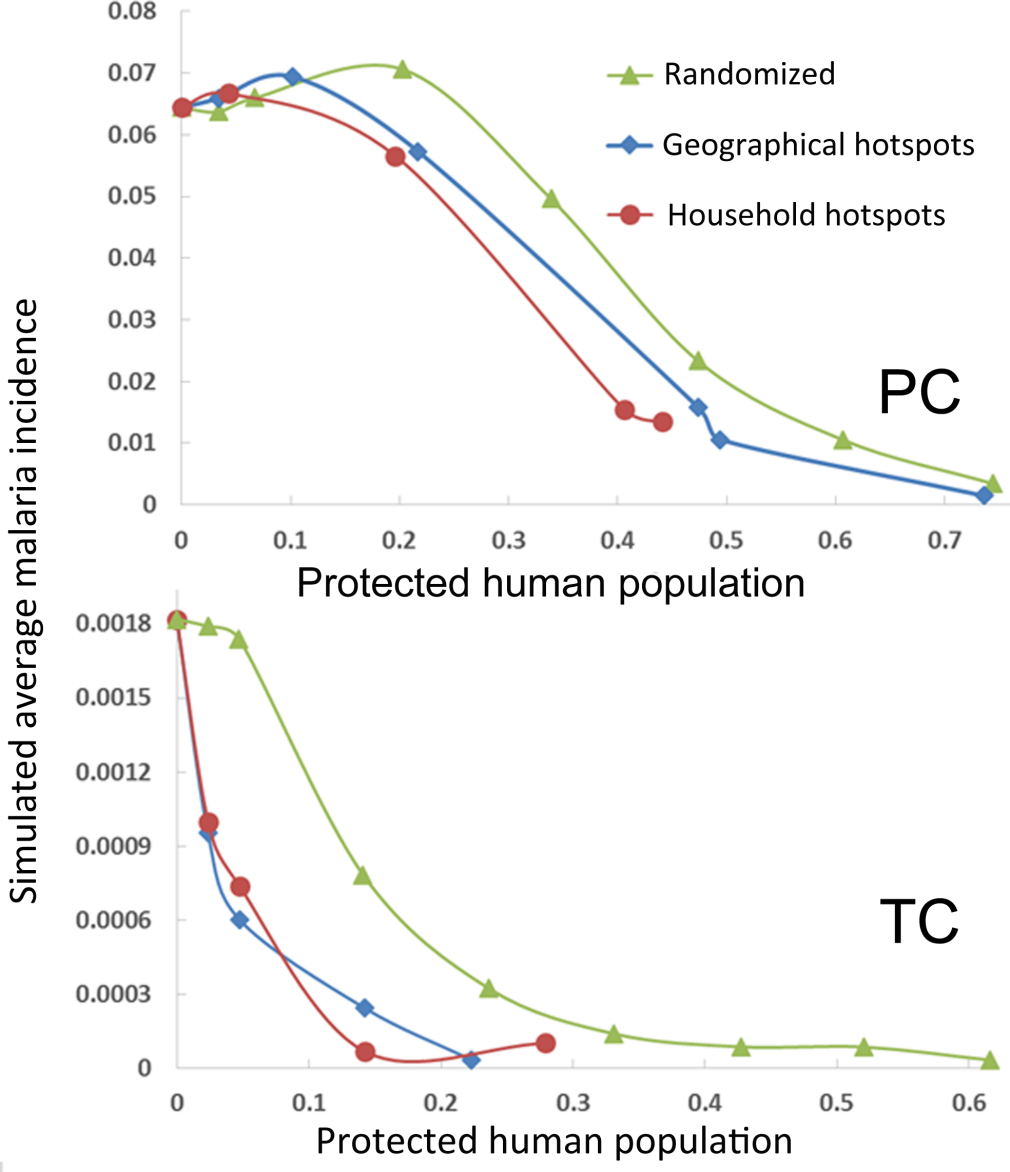
Simulation outputs of malaria control-testing scenarios. The monthly malaria incidence, averaged over the entire ABM study period is presented as a function of the fraction of protected human population in three different control-testing scenarios. Every scenario considers as protected from mosquito bites different categories of human agents. Randomized: an increasing fraction of human agents selected at random in the human population is protected against mosquito bites. Geographical (Households) hotspots: an increasing fraction of human agents entering inside geographical (households) hotspots is protected against mosquito bites. PC: Padre Cocha, TC: Tacsha Curaray.

The “what if” control-testing scenarios are designed to study the effectiveness of targeting malaria hotspots as a control strategy. As expected (see Fig 4), when increasing proportions of randomly picked human agents are protected against mosquito bites, the monthly average incidence shows a remarkable reduction. The malaria incidence is reduced by almost 95% when 75% and 62% of human agents are protected against mosquito bites in PC and TC respectively. The simulations in PC show a slower decline in malaria incidence than TC as a function of the fraction of protected humans. This difference is probably due the fact that the baseline scenario presented a lower incidence in TC than PC.

When human agents protected against mosquito bites are not selected randomly, but instead are selected among the agents that entered hotspots at least once during the simulation, the malaria incidence reduction is more pronounced especially when considering low fractions of protected people. The control-testing scenarios considered are: control of people inside geographical hotspots and control of people inside household hotspots. Both geographic and household hotspots for PC and TC are shown in Fig 3. When 34% of humans are protected in PC, the household scenario is 1.7 times more efficient in reducing malaria incidence than the randomized treatment while the geographical hotspots scenario is 1.3 times more efficient. Considering the simulation outputs for TC we can observe that when the protected fraction is 14% household hotspots and geographical hotspots scenarios are respectively 7.8 and 2.6 times more efficient in controlling malaria than the randomized treatment scenario. When higher fraction of protected people are considered, the geographical hotspots and household hotspot scenarios tend to perform similarly to the randomized scenarios. Nevertheless, we can observe that in the case of TC simulation, due to low baseline malaria incidence, the geographic and household scenarios produce almost complete malaria eradication at values of protected people fraction of 0.28 and 0.14 respectively. In contrast, the randomized protection scenario produces almost complete malaria eradication only when the fraction of protected people is 0.43. In this respect we can conclude that in TC, if eradication costs are considered, the malaria control of households scenario is about 3 times cheaper than the randomized protection scenario while the geographical human protection is about 1.4 times cheaper than the randomized protection.

## Discussion

Malaria transmission modeling and specifically ABMs are a valuable resource to malaria management and control, both because models can improve our understanding of malaria transmission dynamics and also because through modeling it is possible to test control strategies and optimize the management of resources assigned to malaria prevention [19–61]. The ABMs presented in this paper are dedicated to studying local malaria transmission by implementing two sets of “what-if” scenarios in two different locations in Loreto, Peru. When the baseline scenario outputs (“Human Movement” scenario) were compared with the “No Human Movement” scenario outputs, the resulting simulated malaria incidence time series appeared to be similar. Those two scenarios differed only in representing movements of human agents. The comparable incidences time series generated by these two scenarios seems to suggest, as noted in other theoretical studies [62], that mosquito dispersal dominates the malaria transmission process at local scale, relegating the dispersion due to human local scale movements to a negligible role. It is worthwhile to note that the average mosquito flight ranges in the simulations were: 266 m (σ = 230 m, maximum flight range: 2704 m) and 258 m (σ = 128 m, maximum flight range: 1944) for PC and SC respectively. Clearly if we only consider mosquitoes that have given one infectious bite, the flight ranges are longer because these mosquitoes tend to show a longer life span: 371 m (σ = 256 m, maximum flight range: 1944 m) and 523 m (σ = 239 m, maximum flight range: 982) for PC and SC respectively. Therefore, even if the linear extensions of PC and particularly of TC (9 km along the Napo River) are much larger than the mosquitoes’ flight ranges observed in the simulations, no effects of human movement on malaria incidence are observed. Mosquitoes seems to create a mixing network that continuously covers the entire extension of the simulation areas. In this respect we can define local scale malaria transmission as the transmission that is dominated by the mosquitoes’ dispersal. In contrast large scale malaria transmission occurs when the contact network generated by the mosquitoes’ movements is no more able to connect all the exposed individuals and the major determinant of pathogen spreading are human movements. The simulated values of mosquito agents flight ranges, that are compatible with what has been observed in the field for *A*. *darlingi* [63], can explain also the higher influence of local scale human movements in shaping the transmission of other vector-borne diseases like dengue virus. In the case of dengue, the vector, *Aedes aegypti*, shows typically shorter flight range (100 m) than anophelines [64] limiting the spreading effect of vector movements. It is worth noting that mosquito movements, as represented in the models, only approximately portrays the real dispersal of *A*. *darlingi*. Many factors contributing to the dynamics of mosquito dispersal are neglected or included only partially in the ABMs. For instance it is well known that mosquitoes can locate humans following the concentration gradients of several compounds emitted by humans. This process is only partially represented in the models through weights that push the mosquito agent random walk toward pixels containing humans or human households [24]. It is then possible, during windy days that plumes of human odors expand beyond the extension of the random walk weighting mechanism, which is only equal to a mosquito grid pixel (30 m). Another factor not considered in the ABMs is the supposed propensity that some species of *Anopheles* have shown in memorizing the location of places earlier visited [65]. Although there are some serious doubts about the validity of observation about *Culicidae* spatial learning [66], supposing that mosquitoes partially guide their movement using memories about locations of human houses and breeding sites, the mosquitoes dispersion would be much higher in the model than in the real world.

Noticeably human movements are essential for hotspot formation, not because humans contribute to the disease spreading, but because humans spend differing amounts of time in places with dissimilar infection risks. The inclusion of human movements in the baseline scenario generated work-related hotspots centered on farms. The same hotspots do not appear in the “No Human Movement” scenario since, in this second scenario, all the hotspots are centered on households where the human are located during the simulations. In this regard, the ABM simulations tell us that human movements modify the spatial distribution of malaria hotspots and their inclusion in the models is essential to evaluate the correct spatial patterns of malaria risk. The formation of hotspots around farms confirms field observations about increased work-related risks of malaria. ABMs do show that farming is connected with an increased risk of malaria due to the higher EIRs experimented in farms outside the community. These results seem to suggest that spatially explicit ABMs where no human movements are represented, as seen in recent publications [22–24–67], are suitable to track the overall incidence of local scale malaria transmission. Moreover, it is worth noting that when the effect of mosquito bite prevention methods like ITNs is included, it is necessary to consider the change in protection state passing from sleeping to waking hours, as in the “No human Movement” scenario because simulations showed that the “No Human Movement” scenario presents a much lower malaria incidence if the protection state of human agents is not changed from ITN protected to the daily life not protected state (data not showed).

The second set of control-testing scenarios showed that controlling malaria targeting hotspots is more efficient, in terms of resource requirements, with respect to controlling malaria targeting randomly selecting exposed individuals. The first of these control-testing scenarios protects from malaria human agents entering into geographical hotspots. In this scenario, the areas delimited by hotspots typically contain farms, extended mosquito breeding sites, and contain low people densities. As a consequence, the EIR is much higher in these places than inside the households near the village centers. This first control-testing scenario represents an ideal experiment where it is supposed that it would be possible to efficiently protect from malaria all the people entering in malaria high risk areas during the simulations. We note that the actual way by which human agents are protected from malaria is not specified because not relevant. This protection could be any method of vector control or pathogen control such as clothes, mosquitoes repellents, antimalarial treatments, a hypothetical vaccine or any combination of these methods. The ideal setting of this first control-testing scenario is nearly impossible to implement in the real world because it is impossible to know the exact location of every infection site. Still, we considered this scenario as a reference for the second set of control-testing scenarios, which involved protecting individuals inside household hotspots. This second scenario corresponds to what is generally done when epidemiological malaria data are collected, given the impossibility of knowing exactly the infection sites for all cases. Furthermore, the household hotspots scenario was designed to test if a strategy aimed to protect or treat people living in households where registered infection incidences are above the averages, produces results comparable to those obtained targeting people that actually are exposed to the highest EIRs inside geographical hotspots. It is clear that household hotspots do not correspond to the real observed transmission dynamics since the real transmission sites are not considered. Nevertheless, it is possible to imagine that a subset of the people infected in geographical hotspots contribute to the cases involved in the formation of household hotspots. Moreover household hotspots are also shaped by environmental heterogeneities. It is thus not completely unexpected that, as partially observed in the field by Bejon et al. [17], the simulation results characterize the household control strategy as, on average, more efficient than the geographical hotspots targeting strategy. Protecting or treating people in household hotspots could result in an increased level of community protection, blocking the transmission in households where environmental factors are particularly adverse. We want to note that the ABMs presented here do not include human-based heterogeneities that are formed when certain socio-economics conditions cluster, which may generate an increase in malaria risk. Those socio-economics conditions can include poor household construction, low economic conditions, high density of work-related exposed individuals, etc. Furthermore clusters of malaria reservoirs (such as clusters of asymptomatic individuals) do generate high-risk hotspots. Such human-based heterogeneities are connected with living conditions and are therefore significant at the household level. Presumably, the inclusion in the ABMs of these human-based heterogeneities would result in an increase efficiency of household-control strategy. Along the same lines, we acknowledge, that within this modeling framework, we do not take into consideration in- and out-migration from and between communities. Taking into consideration that ABMs have the ability to capture the broad spectrum of migration behaviors among individual agents [68] and that migration is one of the factors contributing to the reemergence of malaria at global scales [69], temporal and long-term migration will be the next following step within our modeling efforts.

## Conclusions

World malaria eradication will be an arduous challenge for future years. Not only global warming and anthropogenic environment changes [70] make this challenge increasingly difficult to address, but the control techniques developed in past decades have been demonstrated not to be fully effective in eliminating malaria [19]. During the past three decades several weakness of malaria control methods have emerged like resistance to antimalarial drugs, resistance to IRSs, appearance of mosquito progressive adaptation strategies. Also we are becoming aware of environmental issues associated with the use of insecticides to alter the environment where mosquitoes live. It is reasonable to say that malaria will be defeated only by expanding our knowledge about the ecology of mosquitoes [71] and adopting a systematic and multi-pronged control approach, including a coordinated set of methods able to tackle the disease from several fronts [72].

The major strength of this study is that, contrary to previous ABM implementations, in which humans have typically been represented as statically assigned to the positions their respective households, the ABMs presented here let humans move around the simulation area. The simulation results show that when community-level malaria incidence is considered, the community-scale human movement inclusion has a little effect on community-simulated incidences. Nevertheless, the models show that the changing human protection states from night to day have a profound influence on individual malaria risks. Human movement has an obvious strong influence on determining the places where mosquito-human and human-mosquito transmission events occur, thus determining an increased risk of malaria for certain categories of individuals that spend time inside high EIR areas, like in the case of farmers in the presented simulations. Simulations showed that malaria control strategies designed to target cases inside household hotspots produce good results in terms of resource optimization.

The models presented here have several limitations. First, the simulations presented are limited to only two communities, while the complexity of the malaria transmission process would require to repeat the same theoretical results across multiple environmental and social conditions where malaria. Moreover it would be interesting to test targeted control of malaria inside hotspots generated by human-based heterogeneities, given that considering human-based heterogeneities the control strategy would in principle be more effective. A possible future expansion of the presented ABMs would include the explicit representation of human activities and movements outside the communities, such as those of laboring miners, fishermen and loggers, and also to consider a full implementation of human movement including migration and long-term travels out of the communities.

## Supporting information

S1 File ABMs input data. (ZIP)

S2 File ABMs source code. (ZIP)
