# Supplementary material for: Out of the net: An agent-based model to study human movements influence on local-scale malaria transmission
Source: PLoS One. 2018 Mar 6;13(3):e0193493. doi: 10.1371/journal.pone.0193493 (PMC5839546; doi:10.1371/journal.pone.0193493)
Supplement: S2 File — (ZIP) [file pone.0193493.s002.zip › S2/docs/classdocs/allclasses-frame.html]

All Classes


**All Classes**
  

|  |
| --- |
| AbstractGrid2D   AbstractGrid3D   AbstractScrollable   AdjustablePortrayal2D   Arrow   AsynchronousSteppable   AxesPortrayal3D   Bag   BranchGroupPortrayal3D   CapturingCanvas3D   CausedRuntimeException   ChartGenerator   CircledPortrayal2D   CircledPortrayal3D   CollectionProperties   *ColorMap*   ColorWell   ConePortrayal3D   Console   Continuous2D   Continuous3D   ContinuousPortrayal2D   ContinuousPortrayal3D   *Controller*   CubePortrayal3D   CylinderPortrayal3D   *DataCuller*   DenseGrid2D   DisclosurePanel   Display2D   Display3D   Double2D   Double3D   DoubleBag   DoubleGrid2D   DoubleGrid3D   DrawInfo2D   *DrawPolicy*   Edge   EdgeDrawInfo2D   FacetedPortrayal2D   FastHexaObjectGridPortrayal2D   FastHexaValueGridPortrayal2D   FastObjectGridPortrayal2D   FastValueGridPortrayal2D   FieldPortrayal   FieldPortrayal2D   FieldPortrayal3D   *Fixed2D*   *Grid2D*   *Grid3D*   GUIState   Heap   HexagonalPortrayal2D   HexaObjectGridPortrayal2D   HexaSparseGridPortrayal2D   HexaValueGridPortrayal2D   HistogramGenerator   HistogramSeriesAttributes   HTMLBrowser   ImagePortrayal2D   ImagePortrayal3D   *Indexed*   Inspector   Int2D   Int3D   IntBag   Interval   IntGrid2D   IntGrid3D   LabelledList   LabelledPortrayal2D   LabelledPortrayal3D   LightPortrayal3D   LocationWrapper   *MakesSimState*   *Manipulating2D*   MersenneTwisterFast   MeshPortrayal   MethodStep   MinGapDataCuller   MiniHistogram   MovablePortrayal2D   MovieEncoder   MovieMaker   MultiStep   MutableDouble   MutableDouble2D   MutableDouble3D   MutableInt2D   MutableInt3D   Network   Network.IndexOutIn   NetworkPortrayal2D   NumberTextField   ObjectGrid2D   ObjectGrid3D   ObjectGridPortrayal2D   ObjectGridPortrayal2D.Message   ObjectGridPortrayal3D   *Orientable2D*   *Oriented2D*   OrientedPortrayal2D   OvalPortrayal2D   ParallelSequence   PDFEncoder   PNGEncoder   *Portrayal*   *Portrayal2D*   *Portrayal3D*   Prefs   PrimitivePortrayal3D   *Propertied*   Properties   PropertyField   *Proxiable*   QuadPortrayal   QuadPortrayal.DoubleFilter   QuadPortrayal.Filter   QuadPortrayal.IntFilter   QuadPortrayal.ObjectFilter   RandomSequence   RateAdjuster   RectanglePortrayal2D   *Scalable2D*   ScatterPlotGenerator   ScatterPlotSeriesAttributes   Schedule   Schedule.Key   SelectionBehavior   Sequence   SeriesAttributes   Shape3DPortrayal3D   ShapePortrayal2D   SharedPortrayal3D   SimApplet   SimpleColorMap   SimpleController   SimpleEdgePortrayal2D   SimpleInspector   SimplePortrayal2D   SimplePortrayal3D   SimpleProperties   SimState   SparseField   SparseField.LocationAndIndex   *SparseField2D*   *SparseField3D*   SparseFieldPortrayal3D   SparseGrid2D   SparseGrid2DPortrayal3D   SparseGrid3D   SparseGridPortrayal2D   SparseGridPortrayal3D   SpatialNetwork2D   SpherePortrayal3D   *Steppable*   *Stoppable*   TentativeStep   TilePortrayal   TimeSeriesAttributes   TimeSeriesChartGenerator   ToolTipBehavior   TrailedPortrayal2D   TrailedPortrayal2D.TrailDrawInfo2D   TransformedPortrayal2D   TransformedPortrayal3D   Utilities   *Valuable*   ValueGrid2DPortrayal3D   ValueGridCellInfo   ValueGridPortrayal2D   ValueGridPortrayal3D   ValuePortrayal2D   ValuePortrayal2D.DoubleFilter   ValuePortrayal2D.Filter   ValuePortrayal2D.IntFilter   ValuePortrayal3D   ValuePortrayal3D.DoubleFilter   ValuePortrayal3D.Filter   ValuePortrayal3D.IntFilter   WeakStep   WireFrameBoxPortrayal3D   WordWrap |
