# Supplementary material for: Out of the net: An agent-based model to study human movements influence on local-scale malaria transmission
Source: PLoS One. 2018 Mar 6;13(3):e0193493. doi: 10.1371/journal.pone.0193493 (PMC5839546; doi:10.1371/journal.pone.0193493)
Supplement: S2 File — (ZIP) [file pone.0193493.s002.zip › S2/docs/classdocs/constant-values.html]

Constant Field Values


---


|  |  |  |  |  |  |  |  |  |  |
| --- | --- | --- | --- | --- | --- | --- | --- | --- | --- |
| |  |  |  |  |  |  |  | | --- | --- | --- | --- | --- | --- | --- | | **Overview** | Package | Class | **Tree** | **Deprecated** | **Index** | **Help** | | |  |
| PREV   NEXT | **FRAMES**    **NO FRAMES**     **All Classes** |


---


# Constant Field Values


---

**Contents**

- sim.display.\*- sim.engine.\*- sim.field.\*- sim.portrayal.\*- sim.util.\*

| sim.display.\* |
| --- |

| sim.display.Console | | |
| --- | --- | --- |
| `public static final int` | `DEFAULT_GUTTER` | `5` |
| `public static final int` | `DEFAULT_HEIGHT` | `380` |
| `public static final int` | `DEFAULT_WIDTH` | `380` |
| `public static final int` | `MAXIMUM_STEPS` | `20` |
| `public static final java.lang.String` | `NAME_INDICATOR` | `"NAME:"` |
| `public static final java.lang.String` | `ONLY_INDICATOR` | `"ONLY"` |
| `public static final int` | `PS_PAUSED` | `2` |
| `public static final int` | `PS_PLAYING` | `1` |
| `public static final int` | `PS_STOPPED` | `0` |

| sim.display.Display2D | | |
| --- | --- | --- |
| `public static final int` | `TYPE_PDF` | `1` |
| `public static final int` | `TYPE_PNG` | `2` |
| `public static final int` | `UPDATE_RULE_ALWAYS` | `3` |
| `public static final int` | `UPDATE_RULE_INTERNAL_TIME` | `1` |
| `public static final int` | `UPDATE_RULE_NEVER` | `4` |
| `public static final int` | `UPDATE_RULE_STEPS` | `0` |
| `public static final int` | `UPDATE_RULE_WALLCLOCK_TIME` | `2` |

| sim.display.Prefs | | |
| --- | --- | --- |
| `public static final java.lang.String` | `APP_PREFERENCES` | `"edu/gmu/mason/app/"` |
| `public static final java.lang.String` | `MASON_PREFERENCES` | `"edu/gmu/mason/global/"` |

| sim.display.SimpleController | | |
| --- | --- | --- |
| `public static final int` | `PS_PAUSED` | `2` |
| `public static final int` | `PS_PLAYING` | `1` |
| `public static final int` | `PS_STOPPED` | `0` |

| sim.engine.\* |
| --- |

| sim.engine.ParallelSequence | | |
| --- | --- | --- |
| `public static final int` | `CPUS` | `-1` |

| sim.engine.Schedule | | |
| --- | --- | --- |
| `public static final double` | `AFTER_SIMULATION` | `1d/0d` |
| `public static final double` | `BEFORE_SIMULATION` | `-1.0` |
| `public static final double` | `EPOCH` | `0.0` |
| `public static final double` | `MAXIMUM_INTEGER` | `9.007199254740992E15` |

| sim.field.\* |
| --- |

| sim.field.SparseField | | |
| --- | --- | --- |
| `public static final int` | `INITIAL_BAG_SIZE` | `16` |
| `public static final int` | `LARGE_BAG_RATIO` | `4` |
| `public static final int` | `MIN_BAG_SIZE` | `32` |
| `public static final int` | `REPLACEMENT_BAG_RATIO` | `2` |

| sim.field.grid.DenseGrid2D | | |
| --- | --- | --- |
| `public static final int` | `INITIAL_BAG_SIZE` | `16` |
| `public static final int` | `LARGE_BAG_RATIO` | `4` |
| `public static final int` | `MIN_BAG_SIZE` | `32` |
| `public static final int` | `REPLACEMENT_BAG_RATIO` | `2` |

| sim.portrayal.\* |
| --- |

| sim.portrayal.FieldPortrayal2D | | |
| --- | --- | --- |
| `public static final int` | `DEFAULT` | `0` |
| `public static final int` | `DONT_USE_BUFFER` | `2` |
| `public static final int` | `USE_BUFFER` | `1` |

| sim.portrayal.SimpleInspector | | |
| --- | --- | --- |
| `public static final int` | `DEFAULT_MAX_PROPERTIES` | `25` |

| sim.portrayal.SimplePortrayal2D | | |
| --- | --- | --- |
| `public static final int` | `TYPE_HIT_OBJECT` | `1` |
| `public static final int` | `TYPE_SELECTED_OBJECT` | `0` |

| sim.portrayal.network.SimpleEdgePortrayal2D | | |
| --- | --- | --- |
| `public static final int` | `ALWAYS_SCALE` | `2` |
| `public static final int` | `NEVER_SCALE` | `0` |
| `public static final int` | `SCALE_WHEN_SMALLER` | `1` |
| `public static final int` | `SHAPE_LINE` | `0` |
| `public static final int` | `SHAPE_TRIANGLE` | `1` |

| sim.portrayal.simple.AdjustablePortrayal2D | | |
| --- | --- | --- |
| `public static final double` | `CIRCLE_RADIUS` | `30.0` |
| `public static final double` | `KNOB_RADIUS` | `5.0` |
| `public static final double` | `SLOP` | `5.0` |

| sim.portrayal.simple.CircledPortrayal2D | | |
| --- | --- | --- |
| `public static final double` | `DEFAULT_OFFSET` | `0.0` |
| `public static final double` | `DEFAULT_SCALE` | `2.0` |

| sim.portrayal.simple.LabelledPortrayal2D | | |
| --- | --- | --- |
| `public static final int` | `ALIGN_CENTER` | `0` |
| `public static final int` | `ALIGN_LEFT` | `1` |
| `public static final int` | `ALIGN_RIGHT` | `-1` |
| `public static final int` | `ALWAYS_SCALE` | `2` |
| `public static final double` | `DEFAULT_OFFSET_X` | `0.0` |
| `public static final double` | `DEFAULT_OFFSET_Y` | `10.0` |
| `public static final double` | `DEFAULT_SCALE_X` | `0.0` |
| `public static final double` | `DEFAULT_SCALE_Y` | `0.5` |
| `public static final int` | `NEVER_SCALE` | `0` |
| `public static final int` | `SCALE_WHEN_SMALLER` | `1` |

| sim.portrayal.simple.OrientedPortrayal2D | | |
| --- | --- | --- |
| `public static final int` | `DEFAULT_OFFSET` | `0` |
| `public static final double` | `DEFAULT_SCALE` | `0.5` |
| `public static final int` | `SHAPE_COMPASS` | `2` |
| `public static final int` | `SHAPE_KITE` | `1` |
| `public static final int` | `SHAPE_LINE` | `0` |

| sim.portrayal.simple.TrailedPortrayal2D | | |
| --- | --- | --- |
| `public static final double` | `DEFAULT_MAXIMUM_JUMP` | `0.75` |

| sim.portrayal3d.\* |
| --- |

| sim.portrayal3d.simple.CircledPortrayal3D | | |
| --- | --- | --- |
| `public static final double` | `DEFAULT_SCALE` | `2.0` |

| sim.portrayal3d.simple.SpherePortrayal3D | | |
| --- | --- | --- |
| `public static final int` | `DEFAULT_DIVISIONS` | `15` |

| sim.portrayal3d.simple.ValuePortrayal3D | | |
| --- | --- | --- |
| `public static final int` | `SHAPE_CUBE` | `0` |
| `public static final int` | `SHAPE_SQUARE` | `1` |

| sim.util.\* |
| --- |

| sim.util.gui.PropertyField | | |
| --- | --- | --- |
| `public static final int` | `SHOW_CHECKBOX` | `0` |
| `public static final int` | `SHOW_LIST` | `4` |
| `public static final int` | `SHOW_SLIDER` | `3` |
| `public static final int` | `SHOW_TEXTFIELD` | `1` |
| `public static final int` | `SHOW_VIEWBUTTON` | `2` |

| sim.util.media.PNGEncoder | | |
| --- | --- | --- |
| `public static final int` | `FILTER_NONE` | `0` |
| `public static final int` | `FILTER_SUB` | `1` |
| `public static final int` | `FILTER_UP` | `2` |

| sim.util.media.chart.ChartGenerator | | |
| --- | --- | --- |
| `public static final long` | `FORCE_KEY` | `-2L` |

---


|  |  |  |  |  |  |  |  |  |  |
| --- | --- | --- | --- | --- | --- | --- | --- | --- | --- |
| |  |  |  |  |  |  |  | | --- | --- | --- | --- | --- | --- | --- | | **Overview** | Package | Class | **Tree** | **Deprecated** | **Index** | **Help** | | |  |
| PREV   NEXT | **FRAMES**    **NO FRAMES**     **All Classes** |


---
