# Supplementary material for: Out of the net: An agent-based model to study human movements influence on local-scale malaria transmission
Source: PLoS One. 2018 Mar 6;13(3):e0193493. doi: 10.1371/journal.pone.0193493 (PMC5839546; doi:10.1371/journal.pone.0193493)
Supplement: S2 File — (ZIP) [file pone.0193493.s002.zip › S2/docs/classdocs/deprecated-list.html]

Deprecated List


---


|  |  |  |  |  |  |  |  |  |  |
| --- | --- | --- | --- | --- | --- | --- | --- | --- | --- |
| |  |  |  |  |  |  |  | | --- | --- | --- | --- | --- | --- | --- | | **Overview** | Package | Class | **Tree** | **Deprecated** | **Index** | **Help** | | |  |
| PREV   NEXT | **FRAMES**    **NO FRAMES**     **All Classes** |


---


## **Deprecated API**


---

**Contents**

- Deprecated Exceptions- Deprecated Methods- Deprecated Constructors

| **Deprecated Exceptions** | |
| --- | --- |
| sim.util.CausedRuntimeException             *Use standard RuntimeExceptions in Java 1.4 now* |

| **Deprecated Methods** | |
| --- | --- |
| sim.util.gui.LabelledList.addComponent(Component)             *Inserts a component spanning all five columns. Synonym for add(comp)* |
| sim.field.network.Network.cloneGraph() |
| sim.display.SimpleController.doChangeCode(Runnable) |
| sim.display.Controller.doChangeCode(Runnable) |
| sim.display.Console.doChangeCode(Runnable) |
| sim.portrayal.FieldPortrayal2D.getClipLocation(DrawInfo2D)             *use getPositionLocation* |
| sim.util.media.chart.ChartGenerator.getDomainAxisLabel()             *Returns the name of the Domain Axis label -- usually this is the X axis.* |
| sim.util.media.chart.ChartGenerator.getGlobalAttributeCount()             *Use getNumGlobalAttributes* |
| sim.portrayal.simple.TrailedPortrayal2D.getGrowTrailOnlyWhenSelected()             *use getOnlyGrowTrailWhenSelected* |
| sim.display.SimpleController.getIncrementSeedOnPlay()             *renamed to getIncrementSeedOnStop* |
| sim.display.Console.getIncrementSeedOnPlay()             *renamed to getIncrementSeedOnStop* |
| sim.portrayal.network.SpatialNetwork2D.getObjectLocation(Object) |
| sim.util.Properties.getProperties(Object, boolean, boolean, boolean)             *use the full version* |
| sim.util.media.chart.ChartGenerator.getRangeAxisLabel()             *Returns the name of the Range Axis Label -- usually this is the Y axis.* |
| sim.display.Console.getThreadPriority()             *We may eliminate thread priority as an option* |
| sim.display.GUIState.isInspectorVolatile() |
| sim.portrayal.simple.OrientedPortrayal2D.isLineShowing()             *use isOrientationShowing()* |
| sim.display.Display2D.InnerDisplay2D.paintComponent(Graphics, boolean)             *use paintComponent() or paint(...)* |
| sim.display.Display2D.InnerDisplay2D.removeListeners()             *Use Display2D.removeListeners instead.* |
| sim.portrayal.FieldPortrayal.reset()             *Use setDirtyField(false);* |
| sim.portrayal.grid.FastObjectGridPortrayal2D.reset()             *Use setDirtyField(false); Resets the underlying FastValueGridPortrayal2D.* |
| sim.portrayal.grid.FastHexaObjectGridPortrayal2D.reset()             *Resets the underlying FastHexaValueGridPortrayal2D.* |
| sim.display.GUIState.scheduleAtExtreme(Steppable, boolean)             *use scheduleAtStart and scheduleAtEnd instead* |
| sim.display.GUIState.scheduleImmediate(boolean, Steppable)             *use scheduleImmediatelyBefore and scheduleImmediatelyAfter instead* |
| sim.display.GUIState.scheduleImmediateRepeat(boolean, Steppable)             *use scheduleRepeatingImmediatelyBefore and scheduleRepeatingImmediatelyAfter instead* |
| sim.util.media.chart.ChartGenerator.setDomainAxisLabel(String)             *Sets the name of the Domain Axis label -- usually this is the X axis.* |
| sim.util.media.chart.ChartGenerator.setDomainAxisRange(double, double) |
| sim.portrayal.simple.TrailedPortrayal2D.setGrowTrailOnlyWhenSelected(boolean)             *use setOnlyGrowTrailWhenSelected* |
| sim.display.SimpleController.setIncrementSeedOnPlay(boolean)             *renamed to setIncrementSeedOnStop* |
| sim.display.Console.setIncrementSeedOnPlay(boolean)             *renamed to setIncrementSeedOnStop* |
| sim.util.MutableDouble3D.setLength(double)             *use resize instead [renaming]* |
| sim.util.MutableDouble2D.setLength(double)             *use resize instead [renaming]* |
| sim.portrayal.simple.OrientedPortrayal2D.setLineShowing(boolean)             *use setOrientationShowing()* |
| sim.util.MutableInt2D.setLocation(Int2D)             *use setTo* |
| sim.util.MutableInt3D.setLocation(Int3D)             *use setTo* |
| sim.util.MutableInt2D.setLocation(int, int)             *use setTo* |
| sim.util.MutableInt3D.setLocation(int, int, int)             *use setTo* |
| sim.util.MutableInt2D.setLocation(MutableInt2D)             *use setTo* |
| sim.util.MutableInt3D.setLocation(MutableInt3D)             *use setTo* |
| sim.util.MutableInt2D.setLocation(Point)             *use setTo* |
| sim.portrayal.simple.ValuePortrayal2D.setParent(ValueGridPortrayal2D)             *does nothing now* |
| sim.util.media.chart.ChartGenerator.setRangeAxisLabel(String)             *Sets the name of the Range Axis label -- usually this is the Y axis.* |
| sim.util.media.chart.ChartGenerator.setRangeAxisRange(double, double) |
| sim.display.Console.setThreadPriority(int)             *We may eliminate thread priority as an option* |
| sim.engine.AsynchronousSteppable.stopper()             *Will be deleted in the future.* |
| sim.engine.Schedule.time()             *use getTime()* |

| **Deprecated Constructors** | |
| --- | --- |
| sim.display.Display2D(double, double, GUIState, long) |
| sim.display3d.Display3D(double, double, GUIState, long) |
| sim.portrayal.grid.HexaSparseGridPortrayal2D(DrawPolicy)             *Use setDrawPolicy.* |
| sim.util.SimpleProperties(Object, boolean, boolean)             *Use the full form* |
| sim.portrayal.grid.SparseGridPortrayal2D(DrawPolicy)             *Use setDrawPolicy.* |
| sim.portrayal.simple.ValuePortrayal2D(ValueGridPortrayal2D) |

---


|  |  |  |  |  |  |  |  |  |  |
| --- | --- | --- | --- | --- | --- | --- | --- | --- | --- |
| |  |  |  |  |  |  |  | | --- | --- | --- | --- | --- | --- | --- | | **Overview** | Package | Class | **Tree** | **Deprecated** | **Index** | **Help** | | |  |
| PREV   NEXT | **FRAMES**    **NO FRAMES**     **All Classes** |


---
