# Supplementary material for: Out of the net: An agent-based model to study human movements influence on local-scale malaria transmission
Source: PLoS One. 2018 Mar 6;13(3):e0193493. doi: 10.1371/journal.pone.0193493 (PMC5839546; doi:10.1371/journal.pone.0193493)
Supplement: S2 File — (ZIP) [file pone.0193493.s002.zip › S2/docs/classdocs/index-all.html]

 
 
 
 
 
 
Index
 

 

 

 
function windowTitle()
{
    if (location.href.indexOf('is-external=true') == -1) {
        parent.document.title="Index";
    }
}
 
 
 

 

 
 


 
   
  
 
 
 
   
 
   
          Overview     
        Package   
        Class   
          Tree     
          Deprecated     
       Index    
          Help     
   
 
 
  
 
 
 

 
  
 PREV 
 NEXT  
  
    FRAMES     
   NO FRAMES     
  
    All Classes  ');
  }
  //-->
 
 
    All Classes  
 


  
 
 
  
 

 A   B   C   D   E   F   G   H   I   J   K   L   M   N   O   P   Q   R   S   T   U   V   W   X   Y   Z   _   
    
 A  
 
   AbstractGrid2D   - Class in  sim.field.grid  A concrete implementation of the Grid2D methods; used by several subclasses.   AbstractGrid2D()   - 
Constructor for class sim.field.grid. AbstractGrid2D 
  
   AbstractGrid3D   - Class in  sim.field.grid  A concrete implementation of the Grid3D methods; used by several subclasses.   AbstractGrid3D()   - 
Constructor for class sim.field.grid. AbstractGrid3D 
  
   AbstractScrollable   - Class in  sim.util.gui  AbstractScrollable is a JPanel with basic implementation of the Scrollable interface,
    making possible simple anonymous subclasses.   AbstractScrollable()   - 
Constructor for class sim.util.gui. AbstractScrollable 
  
   add(double)   - 
Method in class sim.field.grid. DoubleGrid2D 
 Sets each value in the grid to that value added to  withThisMuch 
        Returns the modified grid.
   add(IntGrid2D)   - 
Method in class sim.field.grid. DoubleGrid2D 
 Sets the value at each location in the grid to that value added to the value at the equivalent location in the provided grid.
   add(DoubleGrid2D)   - 
Method in class sim.field.grid. DoubleGrid2D 
 Sets the value at each location in the grid to that value added to the value at the equivalent location in the provided grid.
   add(double)   - 
Method in class sim.field.grid. DoubleGrid3D 
 Sets each value in the grid to that value added to  withThisMuch 
        Returns the modified grid.
   add(IntGrid3D)   - 
Method in class sim.field.grid. DoubleGrid3D 
 Sets the value at each location in the grid to that value added to the value at the equivalent location in the provided grid.
   add(DoubleGrid3D)   - 
Method in class sim.field.grid. DoubleGrid3D 
 Sets the value at each location in the grid to that value added to the value at the equivalent location in the provided grid.
   add(int)   - 
Method in class sim.field.grid. IntGrid2D 
 Sets each value in the grid to that value added to  withThisMuch 
        Returns the modified grid.
   add(IntGrid2D)   - 
Method in class sim.field.grid. IntGrid2D 
 Sets the value at each location in the grid to that value added to the value at the equivalent location in the provided grid.
   add(int)   - 
Method in class sim.field.grid. IntGrid3D 
 Sets each value in the grid to that value added to  withThisMuch 
        Returns the modified grid.
   add(IntGrid3D)   - 
Method in class sim.field.grid. IntGrid3D 
 Sets the value at each location in the grid to that value added to the value at the equivalent location in the provided grid.
   add(Object)   - 
Method in class sim.util. Bag 
  
   add(Double2D)   - 
Method in class sim.util. Double2D 
  
   add(double)   - 
Method in class sim.util. DoubleBag 
  
   add(Component, Component)   - 
Method in class sim.util.gui. LabelledList 
 Creates a new row, with the given components in the left and right columns of the row respectively.
   add(Component, Component, Component, Component, Component)   - 
Method in class sim.util.gui. LabelledList 
 Creates a new row, with the given components in the farLeft, left, center, right, and farRight columns respectively.
   add(Component)   - 
Method in class sim.util.gui. LabelledList 
 Inserts a component spanning all five columns.
   add(BufferedImage)   - 
Method in class sim.util.gui. MovieMaker 
 Add an image to the movie stream.
   add(Object, Comparable)   - 
Method in class sim.util. Heap 
 Adds an element to the heap with the given key.
   add(int)   - 
Method in class sim.util. IntBag 
  
   add(BufferedImage)   - 
Method in class sim.util.media. MovieEncoder 
 Adds an image to the movie.
   add(MutableDouble2D, MutableDouble2D)   - 
Method in class sim.util. MutableDouble2D 
 Sets me to the sum of other1 and other2, returning me.
   add(Double2D, MutableDouble2D)   - 
Method in class sim.util. MutableDouble2D 
 Sets me to the sum of other1 and other2, returning me.
   add(MutableDouble2D, Double2D)   - 
Method in class sim.util. MutableDouble2D 
 Sets me to the sum of other1 and other2, returning me.
   add(MutableDouble3D, MutableDouble3D)   - 
Method in class sim.util. MutableDouble3D 
 Sets me to the sum of other1 and other2, returning me.
   addAll(Collection)   - 
Method in class sim.util. Bag 
  
   addAll(int, Collection)   - 
Method in class sim.util. Bag 
  
   addAll(Object[])   - 
Method in class sim.util. Bag 
  
   addAll(int, Object[])   - 
Method in class sim.util. Bag 
  
   addAll(Bag)   - 
Method in class sim.util. Bag 
  
   addAll(int, Bag)   - 
Method in class sim.util. Bag 
  
   addAll(double[])   - 
Method in class sim.util. DoubleBag 
  
   addAll(int, double[])   - 
Method in class sim.util. DoubleBag 
  
   addAll(DoubleBag)   - 
Method in class sim.util. DoubleBag 
  
   addAll(int, DoubleBag)   - 
Method in class sim.util. DoubleBag 
  
   addAll(int[])   - 
Method in class sim.util. IntBag 
  
   addAll(int, int[])   - 
Method in class sim.util. IntBag 
  
   addAll(IntBag)   - 
Method in class sim.util. IntBag 
  
   addAll(int, IntBag)   - 
Method in class sim.util. IntBag 
  
   addComponent(Component)   - 
Method in class sim.util.gui. LabelledList 
  Deprecated.   Inserts a component spanning all five columns.  Synonym for add(comp) 
   addEdge(Object, Object, Object)   - 
Method in class sim.field.network. Network 
 Add an edge, storing info as the edge's associated information object.
   addEdge(Edge)   - 
Method in class sim.field.network. Network 
 Add an edge.
   addGlobalAttribute(Component)   - 
Method in class sim.util.media.chart. ChartGenerator 
 Adds a global attribute panel to the frame
   addIn(Double2D)   - 
Method in class sim.util. MutableDouble2D 
 Adds other into me, returning me.
   addIn(MutableDouble2D)   - 
Method in class sim.util. MutableDouble2D 
 Adds other into me, returning me.
   addIn(double, double)   - 
Method in class sim.util. MutableDouble2D 
 Adds the x and y values into my x and y values, returning me.
   addIn(Double3D)   - 
Method in class sim.util. MutableDouble3D 
 Adds other into me, returning me.
   addIn(MutableDouble3D)   - 
Method in class sim.util. MutableDouble3D 
 Adds other into me, returning me.
   addIn(double, double, double)   - 
Method in class sim.util. MutableDouble3D 
 Adds the x, y, and z values into my x, y, and z values, returning me.
   addLabelled(String, Component)   - 
Method in class sim.util.gui. LabelledList 
 Creates a new row, with the given components in the right column, and a JLabel of the given string in the left column.
   addLegend()   - 
Method in class sim.util.media.chart. ChartGenerator 
 Add a legend to the chart unless the chart already has one.
   addNode(Object)   - 
Method in class sim.field.network. Network 
 Add a node
   addObjectsToLocation(Bag, int, int)   - 
Method in class sim.field.grid. DenseGrid2D 
 Adds an object to a given location.
   addObjectsToLocation(Bag, Int2D)   - 
Method in class sim.field.grid. DenseGrid2D 
  
   addObjectsToLocation(Object[], int, int)   - 
Method in class sim.field.grid. DenseGrid2D 
 Adds an object to a given location.
   addObjectsToLocation(Object[], Int2D)   - 
Method in class sim.field.grid. DenseGrid2D 
  
   addObjectsToLocation(Collection, int, int)   - 
Method in class sim.field.grid. DenseGrid2D 
 Adds an object to a given location.
   addObjectToLocation(Object, int, int)   - 
Method in class sim.field.grid. DenseGrid2D 
 Adds an object to a given location.
   addObjectToLocation(Object, Int2D)   - 
Method in class sim.field.grid. DenseGrid2D 
  
   addSeries(double[], int, String, SeriesChangeListener)   - 
Method in class sim.util.media.chart. HistogramGenerator 
 Adds a series, plus a (possibly null) SeriesChangeListener which will receive a  single 
        event if/when the series is deleted from the chart by the user.
   addSeries(double[][], String, org.jfree.data.general.SeriesChangeListener)   - 
Method in class sim.util.media.chart. ScatterPlotGenerator 
  
   addSeries(XYSeries, SeriesChangeListener)   - 
Method in class sim.util.media.chart. TimeSeriesChartGenerator 
 Adds a series, plus a (possibly null) SeriesChangeListener which will receive a  single 
        event if/when the series is deleted from the chart by the user.
   addToAsynchronousRegistry(AsynchronousSteppable)   - 
Method in class sim.engine. SimState 
 Registers an AsynchronousSteppable to get its pause() method called prior to checkpointing,
        its resume() method to be called after checkpointing or recovery, and its stop()
        method to be called at finish() time.
   AdjustablePortrayal2D   - Class in  sim.portrayal.simple  A wrapper for other Portrayal2Ds which makes it possible to do any or all of the following with the mouse:

      AdjustablePortrayal2D(SimplePortrayal2D)   - 
Constructor for class sim.portrayal.simple. AdjustablePortrayal2D 
  
   after   - 
Variable in class sim.display. GUIState 
  
   AFTER_SIMULATION   - 
Static variable in class sim.engine. Schedule 
 The time which indicates that the Schedule is finished.
   afterSize   - 
Variable in class sim.display. GUIState 
  
   align   - 
Variable in class sim.portrayal.simple. LabelledPortrayal2D 
 One of ALIGN_CENTER, ALIGN_LEFT, or ALIGN_RIGHT
   ALIGN_CENTER   - 
Static variable in class sim.portrayal.simple. LabelledPortrayal2D 
  
   ALIGN_LEFT   - 
Static variable in class sim.portrayal.simple. LabelledPortrayal2D 
  
   ALIGN_RIGHT   - 
Static variable in class sim.portrayal.simple. LabelledPortrayal2D 
  
   allControllers   - 
Static variable in class sim.display. Console 
 A weak container for all current consoles and other controllers which wish to be there.
   allNodes   - 
Variable in class sim.field.network. Network 
 All the objects in the sparse field.
   allObjects   - 
Variable in class sim.field. SparseField 
 All the objects in the sparse field.
   ALWAYS_SCALE   - 
Static variable in class sim.portrayal.network. SimpleEdgePortrayal2D 
  
   ALWAYS_SCALE   - 
Static variable in class sim.portrayal.simple. LabelledPortrayal2D 
  
   angle()   - 
Method in class sim.util. Double2D 
 Returns the length of the vector between -Pi and Pi.
   angle()   - 
Method in class sim.util. MutableDouble2D 
 Returns the length of the vector between -Pi and Pi.
   APP_PREFERENCES   - 
Static variable in class sim.display. Prefs 
  
   appearanceForColor(Color)   - 
Static method in class sim.portrayal3d. SimplePortrayal3D 
 Creates an Appearance equivalent to a flat opaque surface of the provided color, needing no lighting.
   appearanceForColors(Color, Color, Color, Color, double, double)   - 
Static method in class sim.portrayal3d. SimplePortrayal3D 
 Creates an Appearance with the provided lit colors.
   appearanceForImage(Image, boolean)   - 
Static method in class sim.portrayal3d. SimplePortrayal3D 
 Creates an Appearance using the provided image.
   Arrow   - Class in  sim.portrayal3d.simple      Arrow(double, Double3D, Double3D, String, String, Appearance)   - 
Constructor for class sim.portrayal3d.simple. Arrow 
 Creates a 3D arrow between points  startPoint  and
  endPoint  if either label is not null, it adds a Text2D object
 at the appropriate end.
   asynchronousRegistry()   - 
Method in class sim.engine. SimState 
 Returns all the AsynchronousSteppable items presently in the registry.
   AsynchronousSteppable   - Class in  sim.engine  Fires up a separate thread which runs until the simulation model requests it be halted.   AsynchronousSteppable()   - 
Constructor for class sim.engine. AsynchronousSteppable 
  
   attach(FieldPortrayal2D, String)   - 
Method in class sim.display. Display2D 
 Attaches a portrayal to the Display2D, along with the provided human-readable name for the portrayal.
   attach(FieldPortrayal2D, String, Rectangle2D.Double)   - 
Method in class sim.display. Display2D 
 Attaches a portrayal to the Display2D, along with the provided human-readable name for the portrayal.
   attach(FieldPortrayal2D, String, boolean)   - 
Method in class sim.display. Display2D 
 Attaches a portrayal to the Display2D, along with the provided
        human-readable name for the portrayal.
   attach(FieldPortrayal2D, String, double, double, boolean)   - 
Method in class sim.display. Display2D 
 Attaches a portrayal to the Display2D, along with the provided human-readable name for the portrayal.
   attach(FieldPortrayal2D, String, Rectangle2D.Double, boolean)   - 
Method in class sim.display. Display2D 
 Attaches a portrayal to the Display2D, along with the provided 
        human-readable name for the portrayal.
   attach(Inspector, String)   - 
Method in class sim.display. Display2D 
 A convenience function: creates a popup menu item of the given name which, when selected, will display the
        given inspector in the Console.
   attach(Portrayal3D, String)   - 
Method in class sim.display3d. Display3D 
 Attaches a portrayal to the Display3D, along with the provided human-readable name for the portrayal.
   attach(Portrayal3D, String, boolean)   - 
Method in class sim.display3d. Display3D 
 Attaches a portrayal to the Display3D, along with the provided human-readable name for the portrayal.
   attach(Inspector, String)   - 
Method in class sim.display3d. Display3D 
 A convenience function: creates a popup menu item of the given name which, when selected, will display the
        given inspector in the Console.
   autoSpinTransformGroup   - 
Variable in class sim.display3d. Display3D 
 The TransformGroup which used to spin the underlying model.
   awakeFromCheckpoint()   - 
Method in class sim.engine. SimState 
 Called after the SimState was created by reading from a checkpointed object.
   AxesPortrayal3D   - Class in  sim.portrayal3d.simple  Draws coordinate system axes 1 unit long each, centered at the origin, 
 and labelled "O", "X", "Y", and "Z".   AxesPortrayal3D(double, boolean)   - 
Constructor for class sim.portrayal3d.simple. AxesPortrayal3D 
  
 
 
    
 B  
 
   Bag   - Class in  sim.util  Maintains a simple array (objs) of Objects and the number of objects (numObjs) in the array
    (the array can be bigger than this number).   Bag()   - 
Constructor for class sim.util. Bag 
  
   Bag(int)   - 
Constructor for class sim.util. Bag 
 Creates a Bag with a given initial capacity.
   Bag(Bag)   - 
Constructor for class sim.util. Bag 
 Adds the objects from the other Bag without copying them.
   Bag(Object[])   - 
Constructor for class sim.util. Bag 
 Creates a Bag with the given elements.
   Bag(Collection)   - 
Constructor for class sim.util. Bag 
 Creates a Bag with the given elements.
   baseWidth   - 
Variable in class sim.portrayal.network. SimpleEdgePortrayal2D 
  
   before   - 
Variable in class sim.display. GUIState 
  
   BEFORE_SIMULATION   - 
Static variable in class sim.engine. Schedule 
 The time which indicates that the Schedule hasn't started yet.
   beforeSize   - 
Variable in class sim.display. GUIState 
  
   beginCapturing(boolean)   - 
Method in class sim.display3d. CapturingCanvas3D 
 sets the capturing regime and area
   betterToString(Object)   - 
Method in class sim.util. Properties 
 Call this to get a prettier print-name for an object -- converting arrays to a nicer format, for example.
   BranchGroupPortrayal3D   - Class in  sim.portrayal3d.simple  Loads a Lightwave 3D scene file (.lwo or .lws extension) or a Wavefront object file (.obj extension) into a BranchGroup, 
 then attaches this as a portrayal.   BranchGroupPortrayal3D(BranchGroup)   - 
Constructor for class sim.portrayal3d.simple. BranchGroupPortrayal3D 
 Constructs a BranchGroupPortrayal3D with the given scene file loader without changing its appearance, scale, or transform.
   BranchGroupPortrayal3D(BranchGroup, double)   - 
Constructor for class sim.portrayal3d.simple. BranchGroupPortrayal3D 
 Constructs a BranchGroupPortrayal3D with the given scene file loader without changing its appearance, but scaling it.
   BranchGroupPortrayal3D(BranchGroup, Transform3D)   - 
Constructor for class sim.portrayal3d.simple. BranchGroupPortrayal3D 
 Constructs a BranchGroupPortrayal3D with the given scene file loader without changing its appearance, but transforming it.
   BranchGroupPortrayal3D(BranchGroup, Appearance)   - 
Constructor for class sim.portrayal3d.simple. BranchGroupPortrayal3D 
 Constructs a BranchGroupPortrayal3D with the given scene file loader without transforming it or scaling it, but changing its appearance (unless the appearance is null).
   BranchGroupPortrayal3D(BranchGroup, double, Appearance)   - 
Constructor for class sim.portrayal3d.simple. BranchGroupPortrayal3D 
 Constructs a BranchGroupPortrayal3D with the given scene file loader by scaling it and changing its appearance (unless the appearance is null).
   BranchGroupPortrayal3D(BranchGroup, Transform3D, Appearance)   - 
Constructor for class sim.portrayal3d.simple. BranchGroupPortrayal3D 
 Constructs a BranchGroupPortrayal3D with the given scene file loader by transforming it and changing its appearance (unless the appearance is null).
   bufferedHints   - 
Variable in class sim.display. Display2D.InnerDisplay2D 
 Hints used to draw the buffered image to the screen
   buildAttributes()   - 
Method in class sim.util.media.chart. HistogramSeriesAttributes 
  
   buildAttributes()   - 
Method in class sim.util.media.chart. ScatterPlotSeriesAttributes 
  
   buildAttributes()   - 
Method in class sim.util.media.chart. SeriesAttributes 
 Constructs the widget by adding items to the LabelledList.
   buildAttributes()   - 
Method in class sim.util.media.chart. TimeSeriesAttributes 
  
   buildChart()   - 
Method in class sim.util.media.chart. ChartGenerator 
 Override this to construct the appropriate kind of chart.
   buildChart()   - 
Method in class sim.util.media.chart. HistogramGenerator 
  
   buildChart()   - 
Method in class sim.util.media.chart. ScatterPlotGenerator 
  
   buildChart()   - 
Method in class sim.util.media.chart. TimeSeriesChartGenerator 
  
 
 
    
 C  
 
   CAMERA_ICON   - 
Static variable in class sim.display. Display2D 
  
   CAMERA_ICON_P   - 
Static variable in class sim.display. Display2D 
  
   canvas   - 
Variable in class sim.display3d. Display3D 
 The Java3D canvas holding the universe.
   CapturingCanvas3D   - Class in  sim.display3d  Canvas3D that is synchronized with Display3D and 
 uses postswap to save the contextGraphics into
 an image.   CapturingCanvas3D(GraphicsConfiguration)   - 
Constructor for class sim.display3d. CapturingCanvas3D 
  
   CapturingCanvas3D(GraphicsConfiguration, boolean)   - 
Constructor for class sim.display3d. CapturingCanvas3D 
  
   CausedRuntimeException   - Exception in  sim.util   Deprecated.   Use standard RuntimeExceptions in Java 1.4 now    CausedRuntimeException()   - 
Constructor for exception sim.util. CausedRuntimeException 
  Deprecated.   
   CausedRuntimeException(Throwable)   - 
Constructor for exception sim.util. CausedRuntimeException 
  Deprecated.   
   CausedRuntimeException(Throwable, String)   - 
Constructor for exception sim.util. CausedRuntimeException 
  Deprecated.   
   ceiling()   - 
Method in class sim.field.grid. DoubleGrid2D 
 Sets each value in the grid to ceil(value).
   ceiling()   - 
Method in class sim.field.grid. DoubleGrid3D 
 Sets each value in the grid to ceil(value).
   changeColor(Color)   - 
Method in class sim.util.gui. ColorWell 
  
   chart   - 
Variable in class sim.util.media.chart. ChartGenerator 
 The chart
   ChartGenerator   - Class in  sim.util.media.chart  ChartGenerator is a JPanel which displays a chart using the JFreeChart library.   ChartGenerator()   - 
Constructor for class sim.util.media.chart. ChartGenerator 
 Generates a new ChartGenerator with a blank chart.
   chartHolder   - 
Variable in class sim.util.media.chart. ChartGenerator 
 The JScrollPane which holdw the ChartPanel
   chartPanel   - 
Variable in class sim.util.media.chart. ChartGenerator 
 The panel which holds and draws the chart
   child   - 
Variable in class sim.portrayal.simple. AdjustablePortrayal2D 
  
   child   - 
Variable in class sim.portrayal.simple. CircledPortrayal2D 
  
   child   - 
Variable in class sim.portrayal.simple. LabelledPortrayal2D 
  
   child   - 
Variable in class sim.portrayal.simple. MovablePortrayal2D 
  
   child   - 
Variable in class sim.portrayal.simple. OrientedPortrayal2D 
  
   child   - 
Variable in class sim.portrayal.simple. TrailedPortrayal2D 
 The Child portrayal of this portrayal: a SimplePortrayal2D used solely for determining hit testing.
   child   - 
Variable in class sim.portrayal.simple. TransformedPortrayal2D 
  
   children   - 
Variable in class sim.portrayal.simple. FacetedPortrayal2D 
  
   circle   - 
Static variable in class sim.portrayal.simple. AdjustablePortrayal2D 
  
   CIRCLE_RADIUS   - 
Static variable in class sim.portrayal.simple. AdjustablePortrayal2D 
  
   CircledPortrayal2D   - Class in  sim.portrayal.simple  A wrapper for other Portrayal2Ds which also draws a big circle around them -- useful for
   distinguishing one object from other similar-looking objects.   CircledPortrayal2D(SimplePortrayal2D, double, double, Paint, boolean)   - 
Constructor for class sim.portrayal.simple. CircledPortrayal2D 
 If child is null, then the underlying model object 
        is presumed to be a Portrayal2D and will be used.
   CircledPortrayal2D(SimplePortrayal2D)   - 
Constructor for class sim.portrayal.simple. CircledPortrayal2D 
 Draw a circle of radius scale = 2.0, dr = 0, in blue.
   CircledPortrayal2D(SimplePortrayal2D, Paint, boolean)   - 
Constructor for class sim.portrayal.simple. CircledPortrayal2D 
 Draw a circle of radius or = 2.0, dr = 0.
   CircledPortrayal3D   - Class in  sim.portrayal3d.simple  A wrapper for other Portrayal3Ds which also draws a big translucent sphere around them -- useful for
   distinguishing one object from other similar-looking objects.   CircledPortrayal3D(SimplePortrayal3D)   - 
Constructor for class sim.portrayal3d.simple. CircledPortrayal3D 
  
   CircledPortrayal3D(SimplePortrayal3D, double)   - 
Constructor for class sim.portrayal3d.simple. CircledPortrayal3D 
  
   CircledPortrayal3D(SimplePortrayal3D, double, boolean)   - 
Constructor for class sim.portrayal3d.simple. CircledPortrayal3D 
  
   CircledPortrayal3D(SimplePortrayal3D, Color)   - 
Constructor for class sim.portrayal3d.simple. CircledPortrayal3D 
  
   CircledPortrayal3D(SimplePortrayal3D, Color, double, boolean)   - 
Constructor for class sim.portrayal3d.simple. CircledPortrayal3D 
  
   CircledPortrayal3D(SimplePortrayal3D, Appearance, double, boolean)   - 
Constructor for class sim.portrayal3d.simple. CircledPortrayal3D 
  
   classPortrayals   - 
Variable in class sim.portrayal. FieldPortrayal 
  
   cleaningAsynchronous   - 
Variable in class sim.engine. SimState 
  
   cleanup()   - 
Method in class sim.engine. ParallelSequence 
 Call this just before you get rid of a ParallelSequence: for example, one good place is the stop() method of
        your simulation.
   clear()   - 
Method in class sim.engine. Schedule 
 Empties out the schedule but does not reset the time or steps.
   clear()   - 
Method in class sim.field.continuous. Continuous2D 
  
   clear()   - 
Method in class sim.field.continuous. Continuous3D 
  
   clear()   - 
Method in class sim.field.grid. DenseGrid2D 
 Sets all the locations in the grid to null, and returns in a Bag all stored objects 
        (including duplicates but not null values).
   clear()   - 
Method in class sim.field.grid. ObjectGrid2D 
 Sets all the locations in the grid to null, and returns in a Bag all previously stored objects 
        (including duplicates but not null values).
   clear()   - 
Method in class sim.field.grid. ObjectGrid3D 
 Sets all the locations in the grid to null, and returns in a Bag all stored objects 
        (including duplicates but not null values).
   clear()   - 
Method in class sim.field.network. Network 
 Removes all nodes, deleting all edges from the Field as well.
   clear()   - 
Method in class sim.field. SparseField 
 Deletes everything, returning all the objects as a Bag (which you can freely use and modify).
   clear()   - 
Method in class sim.util. Bag 
 Removes all objects in the Bag.
   clear()   - 
Method in class sim.util. DoubleBag 
 Removes all numbers in the DoubleBag.
   clear()   - 
Method in class sim.util. Heap 
  
   clear()   - 
Method in class sim.util. IntBag 
 Removes all numbers in the IntBag.
   clearBackdrop()   - 
Method in class sim.display3d. Display3D 
 Clears any backdrop presently being used in the scene, turns off the backdrop checkbox, and disables the backdrop checkbox.
   clearPickableFlags(Node)   - 
Static method in class sim.portrayal3d. SimplePortrayal3D 
 Utility method which makes the given Node unpickable.
   clearSelections()   - 
Method in class sim.display. Display2D 
  
   clearSelections()   - 
Method in class sim.display3d. Display3D 
  
   clip   - 
Variable in class sim.portrayal. DrawInfo2D 
  
   clone()   - 
Method in class ec.util. MersenneTwisterFast 
  
   clone()   - 
Method in class sim.util. Bag 
  
   clone()   - 
Method in class sim.util. DoubleBag 
  
   clone()   - 
Method in class sim.util. IntBag 
  
   clone()   - 
Method in class sim.util. MutableDouble 
  
   clone()   - 
Method in class sim.util. MutableDouble2D 
  
   clone()   - 
Method in class sim.util. MutableDouble3D 
  
   clone()   - 
Method in class sim.util. MutableInt2D 
  
   clone()   - 
Method in class sim.util. MutableInt3D 
  
   cloneGraph()   - 
Method in class sim.field.network. Network 
  Deprecated.     
   CollectionProperties   - Class in  sim.util  A simple class for examining the slots of Maps, Collections, Indexed, and arrays as if the slots were Java Bean Properties.   CollectionProperties(Object)   - 
Constructor for class sim.util. CollectionProperties 
 Object can be a Collection, a List, a Map, an Indexed, or an array.
   ColorMap   - Interface in  sim.util.gui  ColorMap is a interface for mapping numerical values to colors.   colors   - 
Variable in class sim.util.gui. SimpleColorMap 
 User-provided color table
   ColorWell   - Class in  sim.util.gui      ColorWell()   - 
Constructor for class sim.util.gui. ColorWell 
  
   ColorWell(Color)   - 
Constructor for class sim.util.gui. ColorWell 
  
   compareTo(Object)   - 
Method in class sim.engine. Schedule.Key 
  
   compareTo(Object)   - 
Method in class sim.field.network. Edge 
  
   completedWrapper(LocationWrapper, PickIntersection, PickResult)   - 
Method in class sim.portrayal3d.continuous. ContinuousPortrayal3D 
  
   completedWrapper(LocationWrapper, PickIntersection, PickResult)   - 
Method in class sim.portrayal3d. FieldPortrayal3D 
 Given the provided PickIntersection and the PickResult it came from, fill in w the location of 
        the picked object, and return it.
   completedWrapper(LocationWrapper, PickIntersection, PickResult)   - 
Method in class sim.portrayal3d.grid. ObjectGridPortrayal3D 
  
   completedWrapper(LocationWrapper, PickIntersection, PickResult)   - 
Method in class sim.portrayal3d.grid. SparseGridPortrayal3D 
  
   completedWrapper(LocationWrapper, PickIntersection, PickResult)   - 
Method in class sim.portrayal3d.grid. ValueGrid2DPortrayal3D 
  
   completedWrapper(LocationWrapper, PickIntersection, PickResult)   - 
Method in class sim.portrayal3d.grid. ValueGridPortrayal3D 
  
   componentType()   - 
Method in class sim.util. Bag 
 Always returns null.
   componentType()   - 
Method in class sim.util. DoubleBag 
  
   componentType()   - 
Method in interface sim.util. Indexed 
 Should return the base component type for this Indexed object, or
        null if the component type should be queried via getValue(index).getClass.getComponentType()
   componentType()   - 
Method in class sim.util. IntBag 
  
   ConePortrayal3D   - Class in  sim.portrayal3d.simple  Portrays objects as a cone of the specified color or appearance (flat opaque white by default)
 which fills the region from (-0.5*scale,-0.5*scale,-0.5*scale) to (0.5*scale,0.5*scale,0.5*scale).   ConePortrayal3D()   - 
Constructor for class sim.portrayal3d.simple. ConePortrayal3D 
 Constructs a ConePortrayal3D with a default (flat opaque white) appearance and a scale of 1.0.
   ConePortrayal3D(double)   - 
Constructor for class sim.portrayal3d.simple. ConePortrayal3D 
 Constructs a ConePortrayal3D with a default (flat opaque white) appearance and the given scale.
   ConePortrayal3D(Color)   - 
Constructor for class sim.portrayal3d.simple. ConePortrayal3D 
 Constructs a ConePortrayal3D with a flat opaque appearance of the given color and a scale of 1.0.
   ConePortrayal3D(Color, double)   - 
Constructor for class sim.portrayal3d.simple. ConePortrayal3D 
 Constructs a ConePortrayal3D with a flat opaque appearance of the given color and the given scale.
   ConePortrayal3D(Image)   - 
Constructor for class sim.portrayal3d.simple. ConePortrayal3D 
 Constructs a ConePortrayal3D with the given (opaque) image and a scale of 1.0.
   ConePortrayal3D(Image, double)   - 
Constructor for class sim.portrayal3d.simple. ConePortrayal3D 
 Constructs a ConePortrayal3D with the given (opaque) image and scale.
   ConePortrayal3D(Appearance, boolean, boolean, double)   - 
Constructor for class sim.portrayal3d.simple. ConePortrayal3D 
 Constructs a ConePortrayal3D with the given appearance and scale, plus whether or not to generate normals or texture coordinates.
   Console   - Class in  sim.display  Console is an elaborate Controller which provides a variety of GUI niceties to control the basics
   of a simulation.   Console(GUIState)   - 
Constructor for class sim.display. Console 
 Creates a Console, using the default initial start behavior (INITIAL_BEHAVIOR_START).
   contains(Object)   - 
Method in class sim.util. Bag 
  
   contains(double)   - 
Method in class sim.util. DoubleBag 
  
   contains(int)   - 
Method in class sim.util. IntBag 
  
   containsAll(Collection)   - 
Method in class sim.util. Bag 
  
   Continuous2D   - Class in  sim.field.continuous  A storage facility for objects located in a continuous 2D environment.   Continuous2D(double, double, double)   - 
Constructor for class sim.field.continuous. Continuous2D 
 Provide expected bounds on the SparseContinuous2D
   Continuous2D(Continuous2D)   - 
Constructor for class sim.field.continuous. Continuous2D 
  
   Continuous3D   - Class in  sim.field.continuous  A storage facility for objects located in a continuous 3D environment.   Continuous3D(double, double, double, double)   - 
Constructor for class sim.field.continuous. Continuous3D 
 Provide expected bounds on the SparseContinuous3D
   Continuous3D(Continuous3D)   - 
Constructor for class sim.field.continuous. Continuous3D 
  
   ContinuousPortrayal2D   - Class in  sim.portrayal.continuous  Portrays Continuous2D fields.   ContinuousPortrayal2D()   - 
Constructor for class sim.portrayal.continuous. ContinuousPortrayal2D 
  
   ContinuousPortrayal3D   - Class in  sim.portrayal3d.continuous  Portrays both Continuous2D and Continuous3D fields.   ContinuousPortrayal3D()   - 
Constructor for class sim.portrayal3d.continuous. ContinuousPortrayal3D 
  
   Controller   - Interface in  sim.display  A Controller is the top-level object responsible for setting up and running the GUIState.   controller   - 
Variable in class sim.display. GUIState 
 The controller for the GUIState.
   controllerUpdate(ControllerEvent)   - 
Method in class sim.util.media. MovieEncoder 
 Controller Listener.
   CPUS   - 
Static variable in class sim.engine. ParallelSequence 
 Indicates that MASON should determine how many threads to use based on the number of CPUs.
   createController()   - 
Method in class sim.display. GUIState 
 Creates and returns a controller ready for the user to manipulate.
   createFrame()   - 
Method in class sim.display. Display2D 
 Creates a frame holding the Display2D.
   createFrame()   - 
Method in class sim.display3d. Display3D 
 Creates a frame holding the Display3D.
   createFrame(Stoppable)   - 
Method in class sim.portrayal. Inspector 
 Creates a scrollable frame surrounding the inspector which calls stop()
        on the underlying stopper when closed.
   createFrame()   - 
Method in class sim.util.media.chart. ChartGenerator 
 Returns a JFrame suitable or housing the ChartGenerator.
   createFrame(Object)   - 
Method in class sim.util.media.chart. ChartGenerator 
  
   createHeap()   - 
Method in class sim.engine. Schedule 
 Returns a Heap to be used by the Schedule.
   createInspectors(Rectangle2D.Double, GUIState)   - 
Method in class sim.display. Display2D 
 Determines the inspectors appropriate for the given selection region (rect), and sends
        them on to the Controller.
   createModel()   - 
Method in class sim.portrayal3d. FieldPortrayal3D 
 Returns a tree structure of the form
        InternalTransformGroup[ model info ].
   createModel()   - 
Method in class sim.portrayal3d.grid. ObjectGridPortrayal3D 
  
   createModel()   - 
Method in class sim.portrayal3d.grid. SparseGrid2DPortrayal3D 
  
   createModel()   - 
Method in class sim.portrayal3d.grid. ValueGrid2DPortrayal3D 
 Format is:
   createModel()   - 
Method in class sim.portrayal3d.grid. ValueGridPortrayal3D 
  
   createModel()   - 
Method in class sim.portrayal3d. SparseFieldPortrayal3D 
  
   createSceneGraph()   - 
Method in class sim.display3d. Display3D 
 Recreates the entire scene graph, including the universe, root, and canvas3d.
   createToolTip()   - 
Method in class sim.display. Display2D.InnerDisplay2D 
  
   createToolTipText(Rectangle2D.Double, GUIState)   - 
Method in class sim.display. Display2D.InnerDisplay2D 
  
   CubePortrayal3D   - Class in  sim.portrayal3d.simple  Portrays objects as a cube of the specified color or appearance (flat opaque white by default)
 which fills the region from (-0.5*scale,-0.5*scale,-0.5*scale) to (0.5*scale,0.5*scale,0.5*scale).   CubePortrayal3D()   - 
Constructor for class sim.portrayal3d.simple. CubePortrayal3D 
 Constructs a CubePortrayal3D with a default (flat opaque white) appearance and a scale of 1.0.
   CubePortrayal3D(double)   - 
Constructor for class sim.portrayal3d.simple. CubePortrayal3D 
 Constructs a CubePortrayal3D with a default (flat opaque white) appearance and the given scale.
   CubePortrayal3D(Color)   - 
Constructor for class sim.portrayal3d.simple. CubePortrayal3D 
 Constructs a CubePortrayal3D with a flat opaque appearance of the given color and a scale of 1.0.
   CubePortrayal3D(Color, double)   - 
Constructor for class sim.portrayal3d.simple. CubePortrayal3D 
 Constructs a CubePortrayal3D with a flat opaque appearance of the given color and the given scale.
   CubePortrayal3D(Image)   - 
Constructor for class sim.portrayal3d.simple. CubePortrayal3D 
 Constructs a CubePortrayal3D with the given (opaque) image and a scale of 1.0.
   CubePortrayal3D(Image, double)   - 
Constructor for class sim.portrayal3d.simple. CubePortrayal3D 
 Constructs a CubePortrayal3D with the given (opaque) image and scale.
   CubePortrayal3D(Appearance, boolean, boolean, double)   - 
Constructor for class sim.portrayal3d.simple. CubePortrayal3D 
 Constructs a CubePortrayal3D with the given appearance and scale, plus whether or not to generate normals or texture coordinates.
   cull(double[], boolean)   - 
Method in interface sim.util.media.chart. DataCuller 
  
   cull(double[], boolean)   - 
Method in class sim.util.media.chart. MinGapDataCuller 
  
   cullToSize(double[], int, IntBag)   - 
Static method in class sim.util.media.chart. MinGapDataCuller 
  
   currentValue   - 
Variable in class sim.util.gui. NumberTextField 
  
   CylinderPortrayal3D   - Class in  sim.portrayal3d.simple  Portrays objects as a cylinder of the specified color or appearance (flat opaque white by default)
 which fills the region from (-0.5*scale,-0.5*scale,-0.5*scale) to (0.5*scale,0.5*scale,0.5*scale).   CylinderPortrayal3D()   - 
Constructor for class sim.portrayal3d.simple. CylinderPortrayal3D 
 Constructs a CylinderPortrayal3D with a default (flat opaque white) appearance and a scale of 1.0.
   CylinderPortrayal3D(double)   - 
Constructor for class sim.portrayal3d.simple. CylinderPortrayal3D 
 Constructs a CylinderPortrayal3D with a default (flat opaque white) appearance and the given scale.
   CylinderPortrayal3D(Color)   - 
Constructor for class sim.portrayal3d.simple. CylinderPortrayal3D 
 Constructs a CylinderPortrayal3D with a flat opaque appearance of the given color and a scale of 1.0.
   CylinderPortrayal3D(Color, double)   - 
Constructor for class sim.portrayal3d.simple. CylinderPortrayal3D 
 Constructs a CylinderPortrayal3D with a flat opaque appearance of the given color and the given scale.
   CylinderPortrayal3D(Image)   - 
Constructor for class sim.portrayal3d.simple. CylinderPortrayal3D 
 Constructs a CylinderPortrayal3D with the given (opaque) image and a scale of 1.0.
   CylinderPortrayal3D(Image, double)   - 
Constructor for class sim.portrayal3d.simple. CylinderPortrayal3D 
 Constructs a CylinderPortrayal3D with the given (opaque) image and scale.
   CylinderPortrayal3D(Appearance, boolean, boolean, double)   - 
Constructor for class sim.portrayal3d.simple. CylinderPortrayal3D 
 Constructs a CylinderPortrayal3D with the given appearance and scale, plus whether or not to generate normals or texture coordinates.
 
 
    
 D  
 
   DataCuller   - Interface in  sim.util.media.chart  This is meant to accomodate an on-line algorithm for keeping a constant number of data points
 from an on-going time series.   dataSinkUpdate(DataSinkEvent)   - 
Method in class sim.util.media. MovieEncoder 
 Event handler for the file writer.
   DEFAULT   - 
Static variable in class sim.portrayal. FieldPortrayal2D 
 Default buffering: let the program decide on its own (typically in a platform-dependent fashion)
   DEFAULT_APPEARANCE   - 
Static variable in class sim.portrayal3d. SimplePortrayal3D 
 Flat white opaque.
   DEFAULT_CIRCLED_APPEARANCE   - 
Static variable in class sim.portrayal3d.simple. CircledPortrayal3D 
  
   DEFAULT_DIVISIONS   - 
Static variable in class sim.portrayal3d.simple. SpherePortrayal3D 
  
   DEFAULT_GUTTER   - 
Static variable in class sim.display. Console 
 When the Console is laid out to the right of some window, the space allocated between it and the window
   DEFAULT_HEIGHT   - 
Static variable in class sim.display. Console 
 Default height of the Console.
   DEFAULT_LABEL_OFFSET   - 
Static variable in class sim.portrayal3d.simple. LabelledPortrayal3D 
  
   DEFAULT_MAX_COLOR   - 
Static variable in class sim.portrayal.simple. TrailedPortrayal2D 
  
   DEFAULT_MAX_PROPERTIES   - 
Static variable in class sim.portrayal. SimpleInspector 
  
   DEFAULT_MAXIMUM_JUMP   - 
Static variable in class sim.portrayal.simple. TrailedPortrayal2D 
  
   DEFAULT_MIN_COLOR   - 
Static variable in class sim.portrayal.simple. TrailedPortrayal2D 
  
   DEFAULT_OFFSET   - 
Static variable in class sim.portrayal.simple. CircledPortrayal2D 
  
   DEFAULT_OFFSET   - 
Static variable in class sim.portrayal.simple. OrientedPortrayal2D 
  
   DEFAULT_OFFSET_X   - 
Static variable in class sim.portrayal.simple. LabelledPortrayal2D 
  
   DEFAULT_OFFSET_Y   - 
Static variable in class sim.portrayal.simple. LabelledPortrayal2D 
  
   DEFAULT_PREFERENCES_KEY   - 
Variable in class sim.display. Display2D 
  
   DEFAULT_PREFERENCES_KEY   - 
Variable in class sim.display3d. Display3D 
  
   DEFAULT_SCALE   - 
Static variable in class sim.portrayal.simple. CircledPortrayal2D 
  
   DEFAULT_SCALE   - 
Static variable in class sim.portrayal.simple. OrientedPortrayal2D 
  
   DEFAULT_SCALE   - 
Static variable in class sim.portrayal3d.simple. CircledPortrayal3D 
  
   DEFAULT_SCALE_X   - 
Static variable in class sim.portrayal.simple. LabelledPortrayal2D 
  
   DEFAULT_SCALE_Y   - 
Static variable in class sim.portrayal.simple. LabelledPortrayal2D 
  
   DEFAULT_WIDTH   - 
Static variable in class sim.display. Console 
 Default width of the Console.
   defaultValue()   - 
Method in interface sim.util.gui. ColorMap 
 Returns  some  level which is valid (that is, validLevel(defaultValue()) should
        always return true).
   defaultValue()   - 
Method in class sim.util.gui. SimpleColorMap 
  
   DenseGrid2D   - Class in  sim.field.grid  A wrapper for 2D arrays of Objects.   DenseGrid2D(int, int)   - 
Constructor for class sim.field.grid. DenseGrid2D 
  
   destroySceneGraph()   - 
Method in class sim.display3d. Display3D 
 Eliminates the existing scene graph.
   detach()   - 
Method in class sim.display3d. SelectionBehavior 
 Detaches the Selection Behavior from the Canvas properly, so leaks don't occur.
   detatchAll()   - 
Method in class sim.display. Display2D 
 Detatches all portrayals from the Display2D.
   detatchAll()   - 
Method in class sim.display3d. Display3D 
 Detatches all portrayals from the Display3D.
   DisclosurePanel   - Class in  sim.util.gui  A panel with a small disclosure triangle which toggles between two subcomponents:
    notionally an "abridged" (short) component and an expanded ("disclosed") component.   DisclosurePanel(String, Component)   - 
Constructor for class sim.util.gui. DisclosurePanel 
  
   DisclosurePanel(String, Component, String)   - 
Constructor for class sim.util.gui. DisclosurePanel 
  
   DisclosurePanel(Component, Component)   - 
Constructor for class sim.util.gui. DisclosurePanel 
  
   DisclosurePanel(Component, Component, String)   - 
Constructor for class sim.util.gui. DisclosurePanel 
  
   discretization   - 
Variable in class sim.field.continuous. Continuous2D 
  
   discretization   - 
Variable in class sim.field.continuous. Continuous3D 
  
   discretize(Double2D)   - 
Method in class sim.field.continuous. Continuous2D 
  
   discretize(Double3D)   - 
Method in class sim.field.continuous. Continuous3D 
  
   display   - 
Variable in class sim.display. Display2D 
 The scroll view which holds the insideDisplay.
   Display2D   - Class in  sim.display  Display2D holds, displays, and manipulates 2D Portrayal objects, allowing the user to scale them,
   scroll them, change how often they're updated, take snapshots, and generate Quicktime movies.   Display2D(double, double, GUIState, long)   - 
Constructor for class sim.display. Display2D 
  Deprecated.     
   Display2D(double, double, GUIState)   - 
Constructor for class sim.display. Display2D 
 Creates a Display2D with the provided width and height for its portrayal region, 
        attached to the provided simulation, and displaying itself with the given interval (which must be > 0).
   Display2D.InnerDisplay2D   - Class in  sim.display  The object which actually does all the drawing.   Display2D.OptionPane   - Class in  sim.display  Option pane   Display3D   - Class in  sim.display3d  Display3D holds, displays, and manipulates 3D Portrayal objects, allowing the user to scale them,
   rotate them, translate them, magnify them, change how often they're updated, take snapshots, and generate Quicktime movies.   Display3D(double, double, GUIState, long)   - 
Constructor for class sim.display3d. Display3D 
  Deprecated.     
   Display3D(double, double, GUIState)   - 
Constructor for class sim.display3d. Display3D 
 Creates a Display3D with the provided width and height for its portrayal region, 
       attached to the provided simulation.
   Display3D.OptionPane3D   - Class in  sim.display3d      disposeFrame()   - 
Method in class sim.portrayal. Inspector 
 Disposes the Inspector's frame if it's not a Controller.
   distance(double, double)   - 
Method in class sim.util. Double2D 
 Returns the distance FROM this Double2D TO the specified point
   distance(Double2D)   - 
Method in class sim.util. Double2D 
 Returns the distance FROM this Double2D TO the specified point.
   distance(Int2D)   - 
Method in class sim.util. Double2D 
 Returns the distance FROM this Double2D TO the specified point.
   distance(MutableInt2D)   - 
Method in class sim.util. Double2D 
 Returns the distance FROM this Double2D TO the specified point.
   distance(Point2D)   - 
Method in class sim.util. Double2D 
 Returns the distance FROM this Double2D TO the specified point.
   distance(double, double, double)   - 
Method in class sim.util. Double3D 
 Returns the distance FROM this Double3D TO the specified point
   distance(Double3D)   - 
Method in class sim.util. Double3D 
 Returns the distance FROM this Double3D TO the specified point.
   distance(Int3D)   - 
Method in class sim.util. Double3D 
 Returns the distance FROM this Double3D TO the specified point.
   distance(MutableInt3D)   - 
Method in class sim.util. Double3D 
 Returns the distance FROM this Double3D TO the specified point.
   distance(double, double)   - 
Method in class sim.util. Int2D 
 Returns the distance FROM this Int2D TO the specified point
   distance(Double2D)   - 
Method in class sim.util. Int2D 
 Returns the distance FROM this Int2D TO the specified point.
   distance(MutableInt2D)   - 
Method in class sim.util. Int2D 
 Returns the distance FROM this Int2D TO the specified point.
   distance(Int2D)   - 
Method in class sim.util. Int2D 
 Returns the distance FROM this Int2D TO the specified point.
   distance(Point2D)   - 
Method in class sim.util. Int2D 
 Returns the distance FROM this Int2D TO the specified point.
   distance(double, double, double)   - 
Method in class sim.util. Int3D 
 Returns the distance FROM this Int3D TO the specified point
   distance(Double3D)   - 
Method in class sim.util. Int3D 
 Returns the distance FROM this Int3D TO the specified point.
   distance(MutableInt3D)   - 
Method in class sim.util. Int3D 
 Returns the distance FROM this Int3D TO the specified point.
   distance(Int3D)   - 
Method in class sim.util. Int3D 
 Returns the distance FROM this Int3D TO the specified point.
   distance(double, double)   - 
Method in class sim.util. MutableDouble2D 
 Returns the distance FROM this MutableDouble2D TO the specified point
   distance(MutableDouble2D)   - 
Method in class sim.util. MutableDouble2D 
 Returns the distance FROM this MutableDouble2D TO the specified point.
   distance(Double2D)   - 
Method in class sim.util. MutableDouble2D 
 Returns the distance FROM this MutableDouble2D TO the specified point.
   distance(Int2D)   - 
Method in class sim.util. MutableDouble2D 
 Returns the distance FROM this MutableDouble2D TO the specified point.
   distance(MutableInt2D)   - 
Method in class sim.util. MutableDouble2D 
 Returns the distance FROM this MutableDouble2D TO the specified point.
   distance(Point2D)   - 
Method in class sim.util. MutableDouble2D 
 Returns the distance FROM this MutableDouble2D TO the specified point.
   distance(double, double, double)   - 
Method in class sim.util. MutableDouble3D 
 Returns the distance FROM this MutableDouble3D TO the specified point
   distance(Double3D)   - 
Method in class sim.util. MutableDouble3D 
 Returns the distance FROM this MutableDouble3D TO the specified point.
   distance(Int3D)   - 
Method in class sim.util. MutableDouble3D 
 Returns the distance FROM this MutableDouble3D TO the specified point.
   distance(MutableInt3D)   - 
Method in class sim.util. MutableDouble3D 
 Returns the distance FROM this MutableDouble3D TO the specified point.
   distance(MutableDouble3D)   - 
Method in class sim.util. MutableDouble3D 
 Returns the distance FROM this MutableDouble3D TO the specified point.
   distance(double, double)   - 
Method in class sim.util. MutableInt2D 
 Returns the distance FROM this MutableInt2D TO the specified point
   distance(Double2D)   - 
Method in class sim.util. MutableInt2D 
 Returns the distance FROM this MutableInt2D TO the specified point.
   distance(MutableInt2D)   - 
Method in class sim.util. MutableInt2D 
 Returns the distance FROM this MutableInt2D TO the specified point.
   distance(Int2D)   - 
Method in class sim.util. MutableInt2D 
 Returns the distance FROM this MutableInt2D TO the specified point.
   distance(Point2D)   - 
Method in class sim.util. MutableInt2D 
 Returns the distance FROM this MutableInt2D TO the specified point.
   distance(double, double, double)   - 
Method in class sim.util. MutableInt3D 
 Returns the distance FROM this Int3D TO the specified point
   distance(Double3D)   - 
Method in class sim.util. MutableInt3D 
 Returns the distance FROM this Int3D TO the specified point.
   distance(MutableInt3D)   - 
Method in class sim.util. MutableInt3D 
 Returns the distance FROM this Int3D TO the specified point.
   distance(Int3D)   - 
Method in class sim.util. MutableInt3D 
 Returns the distance FROM this Int3D TO the specified point.
   distanceSq(double, double)   - 
Method in class sim.util. Double2D 
 Returns the distance FROM this Double2D TO the specified point
   distanceSq(Double2D)   - 
Method in class sim.util. Double2D 
 Returns the distance FROM this Double2D TO the specified point.
   distanceSq(Int2D)   - 
Method in class sim.util. Double2D 
 Returns the distance FROM this Double2D TO the specified point.
   distanceSq(MutableInt2D)   - 
Method in class sim.util. Double2D 
 Returns the distance FROM this Double2D TO the specified point.
   distanceSq(Point2D)   - 
Method in class sim.util. Double2D 
 Returns the distance FROM this Double2D TO the specified point
   distanceSq(double, double, double)   - 
Method in class sim.util. Double3D 
 Returns the squared distance FROM this Double3D TO the specified point
   distanceSq(Double3D)   - 
Method in class sim.util. Double3D 
 Returns the squared distance FROM this Double3D TO the specified point.
   distanceSq(Int3D)   - 
Method in class sim.util. Double3D 
 Returns the squared distance FROM this Double3D TO the specified point.
   distanceSq(MutableInt3D)   - 
Method in class sim.util. Double3D 
 Returns the squared distance FROM this Double3D TO the specified point.
   distanceSq(double, double)   - 
Method in class sim.util. Int2D 
 Returns the squared distance FROM this Int2D TO the specified point
   distanceSq(Double2D)   - 
Method in class sim.util. Int2D 
 Returns the squared distance FROM this Int2D TO the specified point.
   distanceSq(Point2D)   - 
Method in class sim.util. Int2D 
 Returns the squared distance FROM this Int2D TO the specified point
   distanceSq(MutableInt2D)   - 
Method in class sim.util. Int2D 
 Returns the squared distance FROM this Int2D TO the specified point.
   distanceSq(Int2D)   - 
Method in class sim.util. Int2D 
 Returns the squared distance FROM this Int2D TO the specified point.
   distanceSq(double, double, double)   - 
Method in class sim.util. Int3D 
 Returns the squared distance FROM this Int3D TO the specified point
   distanceSq(Double3D)   - 
Method in class sim.util. Int3D 
 Returns the squared distance FROM this Int3D TO the specified point.
   distanceSq(MutableInt3D)   - 
Method in class sim.util. Int3D 
 Returns the squared distance FROM this Int3D TO the specified point.
   distanceSq(Int3D)   - 
Method in class sim.util. Int3D 
 Returns the squared distance FROM this Int3D TO the specified point.
   distanceSq(double, double)   - 
Method in class sim.util. MutableDouble2D 
 Returns the distance FROM this MutableDouble2D TO the specified point
   distanceSq(Double2D)   - 
Method in class sim.util. MutableDouble2D 
 Returns the distance FROM this MutableDouble2D TO the specified point.
   distanceSq(MutableDouble2D)   - 
Method in class sim.util. MutableDouble2D 
 Returns the distance FROM this MutableDouble2D TO the specified point.
   distanceSq(Int2D)   - 
Method in class sim.util. MutableDouble2D 
 Returns the distance FROM this MutableDouble2D TO the specified point.
   distanceSq(MutableInt2D)   - 
Method in class sim.util. MutableDouble2D 
 Returns the distance FROM this MutableDouble2D TO the specified point.
   distanceSq(Point2D)   - 
Method in class sim.util. MutableDouble2D 
 Returns the distance FROM this Point2D TO the specified point
   distanceSq(double, double, double)   - 
Method in class sim.util. MutableDouble3D 
 Returns the squared distance FROM this MutableDouble3D TO the specified point
   distanceSq(Double3D)   - 
Method in class sim.util. MutableDouble3D 
 Returns the squared distance FROM this MutableDouble3D TO the specified point.
   distanceSq(Int3D)   - 
Method in class sim.util. MutableDouble3D 
 Returns the squared distance FROM this MutableDouble3D TO the specified point.
   distanceSq(MutableInt3D)   - 
Method in class sim.util. MutableDouble3D 
 Returns the squared distance FROM this MutableDouble3D TO the specified point.
   distanceSq(MutableDouble3D)   - 
Method in class sim.util. MutableDouble3D 
 Returns the squared distance FROM this MutableDouble3D TO the specified point.
   distanceSq(double, double)   - 
Method in class sim.util. MutableInt2D 
 Returns the squared distance FROM this MutableInt2D TO the specified point
   distanceSq(Double2D)   - 
Method in class sim.util. MutableInt2D 
 Returns the squared distance FROM this MutableInt2D TO the specified point.
   distanceSq(Point2D)   - 
Method in class sim.util. MutableInt2D 
 Returns the squared distance FROM this MutableInt2D TO the specified point
   distanceSq(MutableInt2D)   - 
Method in class sim.util. MutableInt2D 
 Returns the squared distance FROM this MutableInt2D TO the specified point.
   distanceSq(Int2D)   - 
Method in class sim.util. MutableInt2D 
 Returns the squared distance FROM this MutableInt2D TO the specified point.
   distanceSq(double, double, double)   - 
Method in class sim.util. MutableInt3D 
 Returns the squared distance FROM this Int3D TO the specified point
   distanceSq(Double3D)   - 
Method in class sim.util. MutableInt3D 
 Returns the squared distance FROM this Int3D TO the specified point.
   distanceSq(MutableInt3D)   - 
Method in class sim.util. MutableInt3D 
 Returns the squared distance FROM this Int3D TO the specified point.
   distanceSq(Int3D)   - 
Method in class sim.util. MutableInt3D 
 Returns the squared distance FROM this Int3D TO the specified point.
   dlx(int, int)   - 
Method in class sim.field.grid. AbstractGrid2D 
  
   dlx(int, int)   - 
Method in interface sim.field.grid. Grid2D 
 Hex downleft x.
   dlx(int, int)   - 
Method in class sim.field.grid. SparseGrid2D 
  
   dly(int, int)   - 
Method in class sim.field.grid. AbstractGrid2D 
  
   dly(int, int)   - 
Method in interface sim.field.grid. Grid2D 
 Hex downleft y.
   dly(int, int)   - 
Method in class sim.field.grid. SparseGrid2D 
  
   doAbout()   - 
Method in class sim.display. Console 
  
   doChangeCode(Runnable)   - 
Method in class sim.display. Console 
  Deprecated.     
   doChangeCode(Runnable)   - 
Method in interface sim.display. Controller 
  Deprecated.     
   doChangeCode(Runnable)   - 
Method in class sim.display. SimpleController 
  Deprecated.     
   doClose()   - 
Method in class sim.display. Console 
 Closes the Console and shuts down the simulation.
   doClose()   - 
Method in class sim.display. SimpleController 
 Closes the Controller and shuts down the simulation.
   doEnsuredRepaint(Component)   - 
Static method in class sim.util.gui. Utilities 
 Does a repaint that is guaranteed to work (on some systems, plain repaint())
        fails if there's lots of updates going on as is the case in our simulator thread.
   doException(JComponent, Exception)   - 
Method in class sim.display. SimApplet 
  
   doLater(long, Runnable)   - 
Static method in class sim.util.gui. Utilities 
 Schedule something to occur at some specified point in the future in the Swing Event thread.
   doLoop(Class, String[])   - 
Static method in class sim.engine. SimState 
 Calls doLoop(MakesSimState,args), passing in a MakesSimState which creates
        SimStates of the provided Class c, using the constructor new  ( ).
   doLoop(MakesSimState, String[])   - 
Static method in class sim.engine. SimState 
 A convenient top-level loop for the simulation command-line.
   doNew()   - 
Method in class sim.display. Console 
 Pops up a window allowing the user to enter in a class name to start a new simulation.
   doNew()   - 
Method in class sim.display. SimpleController 
 Calls forth the "New Simulation" window.
   DONT_USE_BUFFER   - 
Static variable in class sim.portrayal. FieldPortrayal2D 
 Don't use a buffer
   doOpen()   - 
Method in class sim.display. Console 
 Reverts the current simulation to the simulation stored at a user-specified checkpoint filename.
   doQuit()   - 
Static method in class sim.display. Console 
 Quits the program.
   doSave()   - 
Method in class sim.display. Console 
 Lets the user checkpoint out a simulation to the last checkpoint filename.
   doSaveAs()   - 
Method in class sim.display. Console 
 Lets the user checkpoint out a simulation to a file with a given name.
   dot(Double2D)   - 
Method in class sim.util. Double2D 
 Takes the dot product this Double2D with another
   dot(MutableDouble2D)   - 
Method in class sim.util. MutableDouble2D 
 Returns the dot product of myself against other, that is me DOT other.
   dot(MutableDouble3D)   - 
Method in class sim.util. MutableDouble3D 
 Returns the dot product of myself against other, that is me DOT other.
   Double2D   - Class in  sim.util  Double2D is more or less the same class as java.awt.geom.Point2D.Double, but it is immutable: once the x and y values are set, they cannot be changed (they're final).   Double2D()   - 
Constructor for class sim.util. Double2D 
  
   Double2D(Int2D)   - 
Constructor for class sim.util. Double2D 
  
   Double2D(MutableInt2D)   - 
Constructor for class sim.util. Double2D 
  
   Double2D(MutableDouble2D)   - 
Constructor for class sim.util. Double2D 
  
   Double2D(Point)   - 
Constructor for class sim.util. Double2D 
  
   Double2D(Point2D.Double)   - 
Constructor for class sim.util. Double2D 
  
   Double2D(Point2D.Float)   - 
Constructor for class sim.util. Double2D 
  
   Double2D(Point2D)   - 
Constructor for class sim.util. Double2D 
 Only included for completeness' sakes, in case a new Point2D subclass is created in the future.
   Double2D(double, double)   - 
Constructor for class sim.util. Double2D 
  
   Double3D   - Class in  sim.util  Double3D is more or less the same class as javax.vecmath.Point3d, but it is immutable: once the x and y and z values are set, they cannot be changed (they're final).   Double3D()   - 
Constructor for class sim.util. Double3D 
  
   Double3D(Int2D)   - 
Constructor for class sim.util. Double3D 
 Explicitly assumes the z value is set to 0
   Double3D(Int2D, double)   - 
Constructor for class sim.util. Double3D 
  
   Double3D(Int3D)   - 
Constructor for class sim.util. Double3D 
  
   Double3D(MutableInt2D)   - 
Constructor for class sim.util. Double3D 
 Explicitly assumes the z value is set to 0
   Double3D(MutableInt2D, double)   - 
Constructor for class sim.util. Double3D 
  
   Double3D(MutableInt3D)   - 
Constructor for class sim.util. Double3D 
  
   Double3D(Double2D)   - 
Constructor for class sim.util. Double3D 
 Explicitly assumes the z value is set to 0
   Double3D(Double2D, double)   - 
Constructor for class sim.util. Double3D 
  
   Double3D(Double3D)   - 
Constructor for class sim.util. Double3D 
  
   Double3D(MutableDouble2D)   - 
Constructor for class sim.util. Double3D 
 Explicitly assumes the z value is set to 0
   Double3D(MutableDouble2D, double)   - 
Constructor for class sim.util. Double3D 
  
   Double3D(MutableDouble3D)   - 
Constructor for class sim.util. Double3D 
  
   Double3D(double, double, double)   - 
Constructor for class sim.util. Double3D 
  
   DoubleBag   - Class in  sim.util  Maintains a simple array (objs) of doubles and the number of doubles (numObjs) in the array
    (the array can be bigger than this number).   DoubleBag(int)   - 
Constructor for class sim.util. DoubleBag 
 Creates a DoubleBag with a given initial capacity.
   DoubleBag()   - 
Constructor for class sim.util. DoubleBag 
  
   DoubleBag(DoubleBag)   - 
Constructor for class sim.util. DoubleBag 
 Adds the doubles from the other DoubleBag without copying them.
   DoubleBag(double[])   - 
Constructor for class sim.util. DoubleBag 
 Creates a DoubleBag with the given elements.
   DoubleGrid2D   - Class in  sim.field.grid  A wrapper for 2D arrays of doubles.   DoubleGrid2D(int, int)   - 
Constructor for class sim.field.grid. DoubleGrid2D 
  
   DoubleGrid2D(int, int, double)   - 
Constructor for class sim.field.grid. DoubleGrid2D 
  
   DoubleGrid2D(DoubleGrid2D)   - 
Constructor for class sim.field.grid. DoubleGrid2D 
  
   DoubleGrid3D   - Class in  sim.field.grid  A wrapper for 3D arrays of doubles.   DoubleGrid3D(int, int, int)   - 
Constructor for class sim.field.grid. DoubleGrid3D 
  
   DoubleGrid3D(int, int, int, double)   - 
Constructor for class sim.field.grid. DoubleGrid3D 
  
   DoubleGrid3D(DoubleGrid3D)   - 
Constructor for class sim.field.grid. DoubleGrid3D 
  
   doubleLocationHash   - 
Variable in class sim.field.continuous. Continuous2D 
 Where we store the Double2D values hashed by object
   doubleLocationHash   - 
Variable in class sim.field.continuous. Continuous3D 
 Where we store the Double3D values hashed by object
   doubleValue(Object)   - 
Method in class sim.portrayal.grid. FastHexaObjectGridPortrayal2D 
 Override this as necessary to map the provided object into a double value.
   doubleValue(Object)   - 
Method in class sim.portrayal.grid. FastObjectGridPortrayal2D 
 Override this as necessary to map the provided object into a double value.
   doubleValue(Object)   - 
Method in class sim.portrayal3d.grid. ValueGrid2DPortrayal3D 
  
   doubleValue()   - 
Method in class sim.util. MutableDouble 
  
   doubleValue()   - 
Method in interface sim.util. Valuable 
  
   downx(int, int)   - 
Method in class sim.field.grid. AbstractGrid2D 
  
   downx(int, int)   - 
Method in interface sim.field.grid. Grid2D 
 Hex down x.
   downx(int, int)   - 
Method in class sim.field.grid. SparseGrid2D 
  
   downy(int, int)   - 
Method in class sim.field.grid. AbstractGrid2D 
  
   downy(int, int)   - 
Method in interface sim.field.grid. Grid2D 
 Hex down y.
   downy(int, int)   - 
Method in class sim.field.grid. SparseGrid2D 
  
   draw   - 
Variable in class sim.portrayal. DrawInfo2D 
  
   draw(Object, Graphics2D, DrawInfo2D)   - 
Method in class sim.portrayal. FieldPortrayal2D 
 Draws the field with its origin at [info.draw.x,info.draw.y], relative to the 
        scaled coordinate system defined by [info.draw.width,info.draw.height].
   draw(Object, Graphics2D, DrawInfo2D)   - 
Method in class sim.portrayal.grid. FastHexaObjectGridPortrayal2D 
  
   draw(Object, Graphics2D, DrawInfo2D)   - 
Method in class sim.portrayal.grid. FastObjectGridPortrayal2D 
  
   draw(Object, Graphics2D, DrawInfo2D)   - 
Method in class sim.portrayal.grid. FastValueGridPortrayal2D 
  
   draw(Object, Graphics2D, DrawInfo2D)   - 
Method in class sim.portrayal.network. SimpleEdgePortrayal2D 
  
   draw(Object, Graphics2D, DrawInfo2D)   - 
Method in interface sim.portrayal. Portrayal2D 
 Draw a the given object with an origin at (info.draw.x, info.draw.y),
        and with the coordinate system scaled by so that 1 unit is in the x and
        y directions are equal to info.draw.width and info.draw.height respectively
        in pixels.
   draw(Object, Graphics2D, DrawInfo2D)   - 
Method in class sim.portrayal.simple. AdjustablePortrayal2D 
  
   draw(Object, Graphics2D, DrawInfo2D)   - 
Method in class sim.portrayal.simple. CircledPortrayal2D 
  
   draw(Object, Graphics2D, DrawInfo2D)   - 
Method in class sim.portrayal.simple. FacetedPortrayal2D 
  
   draw(Object, Graphics2D, DrawInfo2D)   - 
Method in class sim.portrayal.simple. ImagePortrayal2D 
  
   draw(Object, Graphics2D, DrawInfo2D)   - 
Method in class sim.portrayal.simple. LabelledPortrayal2D 
  
   draw(Object, Graphics2D, DrawInfo2D)   - 
Method in class sim.portrayal.simple. MovablePortrayal2D 
  
   draw(Object, Graphics2D, DrawInfo2D)   - 
Method in class sim.portrayal.simple. OrientedPortrayal2D 
  
   draw(Object, Graphics2D, DrawInfo2D)   - 
Method in class sim.portrayal.simple. OvalPortrayal2D 
  
   draw(Object, Graphics2D, DrawInfo2D)   - 
Method in class sim.portrayal.simple. RectanglePortrayal2D 
  
   draw(Object, Graphics2D, DrawInfo2D)   - 
Method in class sim.portrayal.simple. ShapePortrayal2D 
  
   draw(Object, Graphics2D, DrawInfo2D)   - 
Method in class sim.portrayal.simple. TrailedPortrayal2D 
  
   draw(Object, Graphics2D, DrawInfo2D)   - 
Method in class sim.portrayal.simple. TransformedPortrayal2D 
  
   draw(Object, Graphics2D, DrawInfo2D)   - 
Method in class sim.portrayal.simple. ValuePortrayal2D 
  
   draw(Object, Graphics2D, DrawInfo2D)   - 
Method in class sim.portrayal. SimplePortrayal2D 
  
   drawFilled   - 
Variable in class sim.portrayal.simple. OrientedPortrayal2D 
  
   DrawInfo2D   - Class in  sim.portrayal  The DrawInfo2D class provides two Rectangles which define a simple drawing situation.   DrawInfo2D(GUIState, FieldPortrayal2D, RectangularShape, RectangularShape)   - 
Constructor for class sim.portrayal. DrawInfo2D 
  
   DrawInfo2D(DrawInfo2D, double, double)   - 
Constructor for class sim.portrayal. DrawInfo2D 
  
   DrawInfo2D(DrawInfo2D)   - 
Constructor for class sim.portrayal. DrawInfo2D 
  
   DrawPolicy   - Interface in  sim.portrayal.grid  Called by Sparse2DPortrayal to determine if all objects
    should be drawn or only one.   drx(int, int)   - 
Method in class sim.field.grid. AbstractGrid2D 
  
   drx(int, int)   - 
Method in interface sim.field.grid. Grid2D 
 Hex downright x.
   drx(int, int)   - 
Method in class sim.field.grid. SparseGrid2D 
  
   dry(int, int)   - 
Method in class sim.field.grid. AbstractGrid2D 
  
   dry(int, int)   - 
Method in interface sim.field.grid. Grid2D 
 Hex downright y.
   dry(int, int)   - 
Method in class sim.field.grid. SparseGrid2D 
  
   dup()   - 
Method in class sim.util. MutableDouble2D 
 Equivalent to  (new MutableDouble2D(d)) , but  (d.dup())  shorter of course, but perhaps not quite as fast.
   dup()   - 
Method in class sim.util. MutableDouble3D 
 Equivalent to  (new MutableDouble3D(d)) , but  (d.dup())  shorter of course, but perhaps not quite as fast.
 
 
    
 E  
 
   ec.util   - package ec.util     Edge   - Class in  sim.field.network  An Edge stores a relationship between two objects in a Network.   Edge(Edge)   - 
Constructor for class sim.field.network. Edge 
  
   Edge(Object, Object, Object)   - 
Constructor for class sim.field.network. Edge 
  
   EdgeDrawInfo2D   - Class in  sim.portrayal.network  An extension of DrawInfo2D for dealing with edges in visualizing network fields.   EdgeDrawInfo2D(GUIState, FieldPortrayal2D, RectangularShape, RectangularShape, Point2D.Double)   - 
Constructor for class sim.portrayal.network. EdgeDrawInfo2D 
  
   EdgeDrawInfo2D(DrawInfo2D, double, double, Point2D.Double)   - 
Constructor for class sim.portrayal.network. EdgeDrawInfo2D 
  
   EdgeDrawInfo2D(DrawInfo2D, Point2D.Double)   - 
Constructor for class sim.portrayal.network. EdgeDrawInfo2D 
  
   EdgeDrawInfo2D(EdgeDrawInfo2D)   - 
Constructor for class sim.portrayal.network. EdgeDrawInfo2D 
  
   elements()   - 
Method in class sim.field.grid. ObjectGrid2D 
 Returns in a Bag all stored objects (including duplicates but not null values).
   elements()   - 
Method in class sim.field.grid. ObjectGrid3D 
 Returns in a Bag all stored objects 
        (including duplicates but not null values).
   ensureFileEndsWith(String, String)   - 
Static method in class sim.util.gui. Utilities 
 Returns a filename guaranteed to end with the given ending.
   EPOCH   - 
Static variable in class sim.engine. Schedule 
 The first possible schedulable time.
   EPOCH_PLUS_EPSILON   - 
Static variable in class sim.engine. Schedule 
 The second possible schedulable time.
   equals(Object)   - 
Method in class sim.engine. Schedule.Key 
  
   equals(Object)   - 
Method in class sim.portrayal. DrawInfo2D 
  
   equals(Object)   - 
Method in class sim.util. Double2D 
  
   equals(Object)   - 
Method in class sim.util. Double3D 
  
   equals(Object)   - 
Method in class sim.util. Int2D 
  
   equals(Object)   - 
Method in class sim.util. Int3D 
  
   equals(Object)   - 
Method in class sim.util. MutableDouble2D 
  
   equals(Object)   - 
Method in class sim.util. MutableDouble3D 
  
   equals(Object)   - 
Method in class sim.util. MutableInt2D 
  
   equals(Object)   - 
Method in class sim.util. MutableInt3D 
  
   exists(Object)   - 
Method in class sim.field. SparseField 
 Returns true if the object is in the field.
   extractMin(Bag)   - 
Method in class sim.util. Heap 
 Removes all key-equal minimum elements and adds them to a Bag, which is then is returned.
   extractMin()   - 
Method in class sim.util. Heap 
 Removes the first minimum element and its key from the heap, and returns the minimum element.
 
 
    
 F  
 
   FacetedPortrayal2D   - Class in  sim.portrayal.simple  A wrapper for multiple Portrayal2Ds which calls on one or the other one according to the
   underlying state of the object, which must be Valuable or a Number of some sort.   FacetedPortrayal2D(SimplePortrayal2D[], boolean)   - 
Constructor for class sim.portrayal.simple. FacetedPortrayal2D 
 If child is null, then the underlying model object 
        is presumed to be a Portrayal2D and will be used.
   FacetedPortrayal2D(SimplePortrayal2D[])   - 
Constructor for class sim.portrayal.simple. FacetedPortrayal2D 
 If child is null, then the underlying model object 
        is presumed to be a Portrayal2D and will be used.
   FastHexaObjectGridPortrayal2D   - Class in  sim.portrayal.grid  A "Fast" version of HexaObjectGridPortrayal2D, which draws objects as rectangles of specified colors,
    rather than using the provided SimplePortrayal2Ds.   FastHexaObjectGridPortrayal2D(boolean)   - 
Constructor for class sim.portrayal.grid. FastHexaObjectGridPortrayal2D 
 If immutableField is true, we presume that the grid doesn't change.
   FastHexaObjectGridPortrayal2D()   - 
Constructor for class sim.portrayal.grid. FastHexaObjectGridPortrayal2D 
 Equivalent to FastHexaObjectGridPortrayal2D(false);
   FastHexaValueGridPortrayal2D   - Class in  sim.portrayal.grid      FastHexaValueGridPortrayal2D(String, boolean)   - 
Constructor for class sim.portrayal.grid. FastHexaValueGridPortrayal2D 
 If immutableField is true, we presume that the grid doesn't change.
   FastHexaValueGridPortrayal2D(String)   - 
Constructor for class sim.portrayal.grid. FastHexaValueGridPortrayal2D 
  
   FastHexaValueGridPortrayal2D(boolean)   - 
Constructor for class sim.portrayal.grid. FastHexaValueGridPortrayal2D 
 If immutableField is true, we presume that the grid doesn't change.
   FastHexaValueGridPortrayal2D()   - 
Constructor for class sim.portrayal.grid. FastHexaValueGridPortrayal2D 
  
   FastObjectGridPortrayal2D   - Class in  sim.portrayal.grid  A "Fast" version of ObjectGridPortrayal2D, which draws objects as rectangles of specified colors,
    rather than using the provided SimplePortrayal2Ds.   FastObjectGridPortrayal2D(boolean)   - 
Constructor for class sim.portrayal.grid. FastObjectGridPortrayal2D 
 If immutableField is true, we presume that the grid doesn't change.
   FastObjectGridPortrayal2D()   - 
Constructor for class sim.portrayal.grid. FastObjectGridPortrayal2D 
 Equivalent to FastObjectGridPortrayal2D(false);
   FastValueGridPortrayal2D   - Class in  sim.portrayal.grid  This class works like a ValueGridPortrayal2D,  except  that it doesn't use an underlying Portrayal for the object
   (instead it always draws a rectangle), and may ignore the getColor() method, so you shouldn't override that method to customize
   the color function in nonlinear ways any more.   FastValueGridPortrayal2D(String, boolean)   - 
Constructor for class sim.portrayal.grid. FastValueGridPortrayal2D 
 If immutableField is true, we presume that the grid doesn't change.
   FastValueGridPortrayal2D(String)   - 
Constructor for class sim.portrayal.grid. FastValueGridPortrayal2D 
  
   FastValueGridPortrayal2D(boolean)   - 
Constructor for class sim.portrayal.grid. FastValueGridPortrayal2D 
 If immutableField is true, we presume that the grid doesn't change.
   FastValueGridPortrayal2D()   - 
Constructor for class sim.portrayal.grid. FastValueGridPortrayal2D 
  
   field   - 
Variable in class sim.field.grid. DenseGrid2D 
  
   field   - 
Variable in class sim.field.grid. DoubleGrid2D 
  
   field   - 
Variable in class sim.field.grid. DoubleGrid3D 
  
   field   - 
Variable in class sim.field.grid. IntGrid2D 
  
   field   - 
Variable in class sim.field.grid. IntGrid3D 
  
   field   - 
Variable in class sim.field.grid. ObjectGrid2D 
  
   field   - 
Variable in class sim.field.grid. ObjectGrid3D 
  
   field   - 
Variable in class sim.portrayal. FieldPortrayal 
  
   fieldPortrayal   - 
Variable in class sim.portrayal. DrawInfo2D 
  
   FieldPortrayal   - Class in  sim.portrayal  A FieldPortrayal is an object which knows how to portray some kind of Field.   FieldPortrayal()   - 
Constructor for class sim.portrayal. FieldPortrayal 
  
   fieldPortrayal   - 
Variable in class sim.portrayal. LocationWrapper 
 The field portrayal depicting this object
   FieldPortrayal2D   - Class in  sim.portrayal  Superclass of all Field Portrayals in 2D.   FieldPortrayal2D()   - 
Constructor for class sim.portrayal. FieldPortrayal2D 
  
   FieldPortrayal3D   - Class in  sim.portrayal3d  Superclass of all Field Portrayals in J3D.   FieldPortrayal3D()   - 
Constructor for class sim.portrayal3d. FieldPortrayal3D 
  
   FieldPortrayal3D(Transform3D)   - 
Constructor for class sim.portrayal3d. FieldPortrayal3D 
  
   fill(Object)   - 
Method in class sim.util. Bag 
 Replaces all elements in the bag with the provided object.
   fill(double)   - 
Method in class sim.util. DoubleBag 
 Replaces all elements in the bag with the provided object.
   fill(int)   - 
Method in class sim.util. IntBag 
 Replaces all elements in the bag with the provided int.
   filled   - 
Variable in class sim.portrayal.simple. OvalPortrayal2D 
  
   filled   - 
Variable in class sim.portrayal.simple. RectanglePortrayal2D 
  
   filled   - 
Variable in class sim.portrayal.simple. ShapePortrayal2D 
  
   FILTER_NONE   - 
Static variable in class sim.util.media. PNGEncoder 
 Constants for filters
   FILTER_SUB   - 
Static variable in class sim.util.media. PNGEncoder 
  
   FILTER_UP   - 
Static variable in class sim.util.media. PNGEncoder 
  
   filterLevel(double)   - 
Method in class sim.util.gui. SimpleColorMap 
  
   finalize()   - 
Method in class sim.display. Display2D 
 Quits the Display2D.
   finalize()   - 
Method in class sim.display3d. Display3D 
 Quits the Display3D.
   finalize()   - 
Method in class sim.engine. AsynchronousSteppable 
  
   finalize()   - 
Method in class sim.engine. ParallelSequence 
  
   finish()   - 
Method in class sim.display. GUIState 
 Called either at the proper or a premature end to the simulation.
   finish()   - 
Method in class sim.engine. SimState 
 Called either at the proper or a premature end to the simulation.
   Fixed2D   - Interface in  sim.portrayal  A Fixed2D object has control over how it is moved by a MovablePortrayal2D object.   floatValue()   - 
Method in class sim.util. MutableDouble 
  
   floor()   - 
Method in class sim.field.grid. DoubleGrid2D 
 Sets each value in the grid to floor(value).
   floor()   - 
Method in class sim.field.grid. DoubleGrid3D 
 Sets each value in the grid to floor(value).
   font   - 
Variable in class sim.portrayal.simple. LabelledPortrayal2D 
 The font of the text.
   FORCE_KEY   - 
Static variable in class sim.util.media.chart. ChartGenerator 
  
   from()   - 
Method in class sim.field.network. Edge 
 Returns the "from" object.
   fromPaint   - 
Variable in class sim.portrayal.network. SimpleEdgePortrayal2D 
  
 
 
    
 G  
 
   generatePDF(Component, File)   - 
Static method in class sim.util.media. PDFEncoder 
  
   generatePDF(JFreeChart, int, int, File)   - 
Static method in class sim.util.media. PDFEncoder 
  
   get(int, int)   - 
Method in class sim.field.grid. DoubleGrid2D 
 Returns the element at location (x,y)
   get(int, int, int)   - 
Method in class sim.field.grid. DoubleGrid3D 
 Returns the element at location (x,y,z)
   get(int, int)   - 
Method in class sim.field.grid. IntGrid2D 
 Returns the element at location (x,y)
   get(int, int, int)   - 
Method in class sim.field.grid. IntGrid3D 
 Returns the element at location (x,y)
   get(int, int)   - 
Method in class sim.field.grid. ObjectGrid2D 
 Returns the element at location (x,y)
   get(int, int, int)   - 
Method in class sim.field.grid. ObjectGrid3D 
  
   get(int)   - 
Method in class sim.util. Bag 
  
   get(int)   - 
Method in class sim.util. DoubleBag 
  
   get(int)   - 
Method in class sim.util. IntBag 
  
   getAbridgedComponent()   - 
Method in class sim.util.gui. DisclosurePanel 
  
   getAdjacencyList(boolean)   - 
Method in class sim.field.network. Network 
 Creates and returns an adjacency list.
   getAdjacencyMatrix()   - 
Method in class sim.field.network. Network 
 Creates and returns a simple adjacency matrix, where only one edge between any two nodes is considered -- if you're
        using a multigraph, use getMultigraphAdjacencyMatrix() instead.
   getAdjustsThickness()   - 
Method in class sim.portrayal.network. SimpleEdgePortrayal2D 
  
   getAllFrames()   - 
Method in class sim.display. Console 
 Returns a list of all displays.
   getAllFrames()   - 
Method in interface sim.display. Controller 
 Returns all registered frames.
   getAllFrames()   - 
Method in class sim.display. SimpleController 
 Returns a list of all displays.
   getAllInspectors()   - 
Method in class sim.display. Console 
 Returns a list of all current inspectors.
   getAllInspectors()   - 
Method in interface sim.display. Controller 
 Returns a list of all current inspectors.
   getAllInspectors()   - 
Method in class sim.display. SimpleController 
 Returns a list of all current inspectors.
   getAllNodes()   - 
Method in class sim.field.network. Network 
 Returns all the objects in the Sparse Field.
   getAllObjects()   - 
Method in class sim.field. SparseField 
 Returns all the objects in the Sparse Field.
   getAlpha(double)   - 
Method in interface sim.util.gui. ColorMap 
 Returns the alpha value for a color for the given level.
   getAlpha(double)   - 
Method in class sim.util.gui. SimpleColorMap 
  
   getAppearance(TransformGroup)   - 
Method in class sim.portrayal3d.simple. PrimitivePortrayal3D 
 Returns an appearance object suitable to set in setAppearance(...).
   getAppPreferences(GUIState, String)   - 
Static method in class sim.display. Prefs 
 Returns app-specific preferences for MASON, with the given additional prefix as a namespace.
   getArrowHead()   - 
Method in class sim.portrayal3d.simple. Arrow 
  
   getArrowTail()   - 
Method in class sim.portrayal3d.simple. Arrow 
  
   getBackdrop()   - 
Method in class sim.display. Display2D 
 Returns the backdrop color or paint.
   getBaseWidth()   - 
Method in class sim.portrayal.network. SimpleEdgePortrayal2D 
  
   getBranchGroupForFile(String)   - 
Static method in class sim.portrayal3d.simple. BranchGroupPortrayal3D 
  
   getBranchGroupForResource(Class, String)   - 
Static method in class sim.portrayal3d.simple. BranchGroupPortrayal3D 
  
   getBranchGroupForURL(URL)   - 
Static method in class sim.portrayal3d.simple. BranchGroupPortrayal3D 
  
   getBuffering()   - 
Method in class sim.portrayal. FieldPortrayal2D 
 Returns whether or not the FieldPortrayal2D will use a buffering "trick" to draw quickly.
   getBuffering()   - 
Method in class sim.portrayal.grid. FastHexaObjectGridPortrayal2D 
  
   getBuffering()   - 
Method in class sim.portrayal.grid. FastObjectGridPortrayal2D 
  
   getCanShowToolTips()   - 
Method in class sim.display3d. ToolTipBehavior 
 Returns whether or not the behavior may show tool tips.
   getCellForIntersection(PickIntersection, Grid2D)   - 
Method in class sim.portrayal3d.grid.quad. MeshPortrayal 
  
   getCellForIntersection(PickIntersection, Grid2D)   - 
Method in class sim.portrayal3d.grid.quad. QuadPortrayal 
  
   getChart()   - 
Method in class sim.util.media.chart. ChartGenerator 
 Returns the underlying chart.
   getChartPanel()   - 
Method in class sim.util.media.chart. ChartGenerator 
 Returns the ChartPanel holding the chart.
   getChild(Object)   - 
Method in class sim.portrayal.simple. AdjustablePortrayal2D 
  
   getChild(Object)   - 
Method in class sim.portrayal.simple. CircledPortrayal2D 
  
   getChild(Object)   - 
Method in class sim.portrayal.simple. LabelledPortrayal2D 
  
   getChild(Object)   - 
Method in class sim.portrayal.simple. MovablePortrayal2D 
  
   getChild(Object)   - 
Method in class sim.portrayal.simple. OrientedPortrayal2D 
  
   getChild(Object)   - 
Method in class sim.portrayal.simple. TransformedPortrayal2D 
  
   getChild(Object)   - 
Method in class sim.portrayal3d.simple. CircledPortrayal3D 
  
   getChild(Object)   - 
Method in class sim.portrayal3d.simple. LabelledPortrayal3D 
  
   getChild(Object)   - 
Method in class sim.portrayal3d.simple. SharedPortrayal3D 
  
   getChild(Object)   - 
Method in class sim.portrayal3d.simple. TransformedPortrayal3D 
  
   getChildIndex(Object, int)   - 
Method in class sim.portrayal.simple. FacetedPortrayal2D 
 Returns the child index to use for the given object.
   getCleaner()   - 
Method in class sim.engine. ParallelSequence 
  
   getClipLocation(DrawInfo2D)   - 
Method in class sim.portrayal. FieldPortrayal2D 
  Deprecated.   use getPositionLocation 
   getColor(double)   - 
Method in interface sim.util.gui. ColorMap 
 Returns a color for the given level
   getColor()   - 
Method in class sim.util.gui. ColorWell 
  
   getColor(double)   - 
Method in class sim.util.gui. SimpleColorMap 
  
   getColorFor(Object)   - 
Method in class sim.portrayal3d.grid. ValueGridPortrayal3D 
 Returns the color presently mapped to the value stored within the given wrapper.
   getCompressionLevel()   - 
Method in class sim.util.media. PNGEncoder 
 Retrieve compression level
   getCurrentDisplay()   - 
Method in class sim.portrayal3d. FieldPortrayal3D 
  
   getCurrentDisplay()   - 
Method in interface sim.portrayal3d. Portrayal3D 
 Returns the current Display3D, or possibly null if it's not been set yet.
   getCurrentDisplay()   - 
Method in class sim.portrayal3d. SimplePortrayal3D 
 If the current display has been set, returns it.
   getCurrentFieldPortrayal()   - 
Method in class sim.portrayal3d. SimplePortrayal3D 
  
   getCurrentGUIState()   - 
Method in class sim.portrayal3d. FieldPortrayal3D 
  
   getCurrentGUIState()   - 
Method in interface sim.portrayal3d. Portrayal3D 
 Returns the current GUIState, or null if no GUIState has been set yet.
   getCurrentGUIState()   - 
Method in class sim.portrayal3d. SimplePortrayal3D 
  
   getDashPattern()   - 
Method in class sim.util.media.chart. TimeSeriesAttributes 
  
   getDataCuller()   - 
Method in class sim.util.media.chart. TimeSeriesChartGenerator 
  
   getDefaultNullPortrayal()   - 
Method in class sim.portrayal. FieldPortrayal 
 Returns a default portrayal for null.
   getDefaultNullPortrayal()   - 
Method in class sim.portrayal.grid. ObjectGridPortrayal2D 
  
   getDefaultPortrayal()   - 
Method in class sim.portrayal.continuous. ContinuousPortrayal2D 
  
   getDefaultPortrayal()   - 
Method in class sim.portrayal. FieldPortrayal 
 Should return a portrayal which can portray any object regardless of
        whether it's valid or not
   getDefaultPortrayal()   - 
Method in class sim.portrayal. FieldPortrayal2D 
  
   getDefaultPortrayal()   - 
Method in class sim.portrayal.grid. ObjectGridPortrayal2D 
  
   getDefaultPortrayal()   - 
Method in class sim.portrayal.grid. SparseGridPortrayal2D 
  
   getDefaultPortrayal()   - 
Method in class sim.portrayal.grid. ValueGridPortrayal2D 
  
   getDefaultPortrayal()   - 
Method in class sim.portrayal.network. NetworkPortrayal2D 
  
   getDefaultPortrayal()   - 
Method in class sim.portrayal3d. FieldPortrayal3D 
  
   getDefaultPortrayal()   - 
Method in class sim.portrayal3d.grid. ValueGrid2DPortrayal3D 
  
   getDefaultPortrayal()   - 
Method in class sim.portrayal3d.grid. ValueGridPortrayal3D 
  
   getDefaultTransform()   - 
Method in class sim.portrayal3d. FieldPortrayal3D 
 Returns the default internal transform suggested for this FieldPortrayal3D.
   getDefaultTransform()   - 
Method in class sim.portrayal3d.simple. TransformedPortrayal3D 
 Returns the default internal transform suggested for this TransformedPortrayal3D.
   getDestroysThreads()   - 
Method in class sim.engine. ParallelSequence 
  
   getDimensions()   - 
Method in class sim.field.continuous. Continuous2D 
  
   getDimensions()   - 
Method in class sim.field.continuous. Continuous3D 
  
   getDimensions()   - 
Method in class sim.field.grid. SparseGrid2D 
  
   getDimensions()   - 
Method in class sim.field.grid. SparseGrid3D 
  
   getDimensions()   - 
Method in interface sim.field. SparseField2D 
 Returns the width and height of the sparse field as a Double2D
   getDimensions()   - 
Method in interface sim.field. SparseField3D 
 Returns the width and height of the sparse field as a Double3D
   getDimensions()   - 
Method in class sim.portrayal.network. SpatialNetwork2D 
  
   getDirected()   - 
Method in class sim.field.network. Edge 
 Returns true if the edge is directed or if we don't know our owner
   getDisclosedComponent()   - 
Method in class sim.util.gui. DisclosurePanel 
  
   getDomain(int)   - 
Method in class sim.util. Properties 
 Returns the domain of the property at the given index.
   getDomain(int)   - 
Method in class sim.util. SimpleProperties 
  
   getDomainAxisLabel()   - 
Method in class sim.util.media.chart. ChartGenerator 
  Deprecated.   Returns the name of the Domain Axis label -- usually this is the X axis. 
   getDrawInfo2D(FieldPortrayal2D, Point2D)   - 
Method in class sim.display. Display2D 
 Constructs a DrawInfo2D for the given portrayal, or null if failed.
   getDrawInfo2D(FieldPortrayal2D, Rectangle2D)   - 
Method in class sim.display. Display2D 
 Constructs a DrawInfo2D for the given portrayal, or null if failed.
   getDrawPolicy()   - 
Method in class sim.portrayal.grid. SparseGridPortrayal2D 
  
   getEdges(Object, Bag)   - 
Method in class sim.field.network. Network 
 Get all the edges that enter or leave a node.
   getEdgesIn(Object)   - 
Method in class sim.field.network. Network 
 Get all edges that enter a node.
   getEdgesOut(Object)   - 
Method in class sim.field.network. Network 
 Get all edges that leave a node.
   getEditedColor()   - 
Method in class sim.util.gui. NumberTextField 
  
   getEditedColor()   - 
Method in class sim.util.gui. PropertyField 
  
   getEncodeAlpha()   - 
Method in class sim.util.media. PNGEncoder 
 Retrieve alpha encoding status.
   getEncodingFormats(float, BufferedImage)   - 
Static method in class sim.util.media. MovieEncoder 
 Returns null and prints an error out to stderr if an error occurred while trying to
        get the formats
   getFame()   - 
Method in class sim.portrayal.continuous. ContinuousPortrayal2D 
  
   getField()   - 
Method in class sim.field.grid. DoubleGrid2D 
  
   getField()   - 
Method in class sim.portrayal. FieldPortrayal 
 Returns the field.
   getField()   - 
Method in class sim.portrayal3d.grid. ValueGrid2DPortrayal3D 
  
   getField()   - 
Method in class sim.portrayal3d.grid. ValueGridPortrayal3D 
  
   getField()   - 
Method in class sim.util.gui. NumberTextField 
  
   getField()   - 
Method in class sim.util.gui. PropertyField 
  
   getFieldPortrayal()   - 
Method in class sim.portrayal. LocationWrapper 
  
   getFillColor()   - 
Method in class sim.util.media.chart. HistogramSeriesAttributes 
  
   getFillOpacity()   - 
Method in class sim.util.media.chart. HistogramSeriesAttributes 
  
   getFilter()   - 
Method in class sim.util.media. PNGEncoder 
 Retrieve filtering scheme
   getFrame()   - 
Method in class sim.display. Display2D 
 Returns the frame holding this Component.
   getFrame()   - 
Method in class sim.display3d. Display3D 
 Returns the frame holding this Component.
   getFrame()   - 
Method in class sim.util.media.chart. ChartGenerator 
 Returns the JFrame which stores the whole chart.
   getFrom()   - 
Method in class sim.field.network. Edge 
 Returns the "from" object.
   getGenerator()   - 
Method in class sim.util.media.chart. SeriesAttributes 
 Returns the ChartGenerator holding the series this SeriesAttributes is responsible for.
   getGlobalAttribute(int)   - 
Method in class sim.util.media.chart. ChartGenerator 
 Returns the global attribute panel of the given index.
   getGlobalAttributeCount()   - 
Method in class sim.util.media.chart. ChartGenerator 
  Deprecated.   Use getNumGlobalAttributes 
   getGlobalPreferences(String)   - 
Static method in class sim.display. Prefs 
 Returns the global preferences for MASON, with the given additional prefix as a namespace.
   getGraphComplement(boolean)   - 
Method in class sim.field.network. Network 
 Complements the graph: same nodes, no edges where they were, edges where they were not.
   getGrowTrailOnlyWhenSelected()   - 
Method in class sim.portrayal.simple. TrailedPortrayal2D 
  Deprecated.   use getOnlyGrowTrailWhenSelected 
   getGUIState()   - 
Method in class sim.portrayal. SimpleInspector 
  
   getHeight()   - 
Method in class sim.field.continuous. Continuous2D 
 Get the height
   getHeight()   - 
Method in class sim.field.continuous. Continuous3D 
 Get the height
   getHeight()   - 
Method in class sim.field.grid. AbstractGrid2D 
  
   getHeight()   - 
Method in class sim.field.grid. AbstractGrid3D 
  
   getHeight()   - 
Method in interface sim.field.grid. Grid2D 
 Returns the width of the field.
   getHeight()   - 
Method in interface sim.field.grid. Grid3D 
 Get the height
   getHeight()   - 
Method in class sim.field.grid. SparseGrid2D 
 Returns the height of the grid
   getHeight()   - 
Method in class sim.field.grid. SparseGrid3D 
 Returns the height of the grid
   getHistogramType()   - 
Method in class sim.util.media.chart. HistogramGenerator 
  
   getImage()   - 
Method in class sim.portrayal3d.grid. ValueGrid2DPortrayal3D 
  
   getIncrementSeedOnPlay()   - 
Method in class sim.display. Console 
  Deprecated.   renamed to getIncrementSeedOnStop 
   getIncrementSeedOnPlay()   - 
Method in class sim.display. SimpleController 
  Deprecated.   renamed to getIncrementSeedOnStop 
   getIncrementSeedOnStop()   - 
Method in class sim.display. Console 
  
   getIncrementSeedOnStop()   - 
Method in class sim.display. SimpleController 
  
   getIndex()   - 
Method in class sim.field. SparseField.LocationAndIndex 
  
   getInfo(Class)   - 
Static method in class sim.display. GUIState 
 Returns either a String or a URL which provides descriptive information about the simulation hosted by
        the given class (which should be a GUIState subclass).
   getInfo()   - 
Static method in class sim.display. GUIState 
 Override this method with a static method of your own 
        in your subclass to provide an object (a URL or a String) describing information about
        your simulation;  if you do not override this method, then the system will
        look for a file called  index.html  located next to your  .class  file
        and use a URL to that file as the information.
   getInfo()   - 
Method in class sim.field.network. Edge 
  
   getInitialValue()   - 
Method in class sim.util.gui. NumberTextField 
  
   getInspector()   - 
Method in class sim.display. GUIState 
 By default returns a non-volatile Inspector which wraps around
        getSimulationInspectedObject(); if getSimulationInspectedObject() returns null, then getInspector()
        will return null also.
   getInspector(LocationWrapper, GUIState)   - 
Method in class sim.portrayal. FieldPortrayal 
  
   getInspector(LocationWrapper, GUIState)   - 
Method in interface sim.portrayal. Portrayal 
 Provide an inspector for an object.
   getInspector(LocationWrapper, GUIState)   - 
Method in class sim.portrayal.simple. AdjustablePortrayal2D 
  
   getInspector(LocationWrapper, GUIState)   - 
Method in class sim.portrayal.simple. CircledPortrayal2D 
  
   getInspector(LocationWrapper, GUIState)   - 
Method in class sim.portrayal.simple. FacetedPortrayal2D 
 If portrayAllChildren, Calls on the first child to return the inspector.
   getInspector(LocationWrapper, GUIState)   - 
Method in class sim.portrayal.simple. LabelledPortrayal2D 
  
   getInspector(LocationWrapper, GUIState)   - 
Method in class sim.portrayal.simple. MovablePortrayal2D 
  
   getInspector(LocationWrapper, GUIState)   - 
Method in class sim.portrayal.simple. OrientedPortrayal2D 
  
   getInspector(LocationWrapper, GUIState)   - 
Method in class sim.portrayal.simple. TrailedPortrayal2D 
  
   getInspector(LocationWrapper, GUIState)   - 
Method in class sim.portrayal.simple. TransformedPortrayal2D 
  
   getInspector(LocationWrapper, GUIState)   - 
Method in class sim.portrayal.simple. ValuePortrayal2D 
  
   getInspector(LocationWrapper, GUIState)   - 
Method in class sim.portrayal. SimplePortrayal2D 
  
   getInspector(LocationWrapper, GUIState)   - 
Method in class sim.portrayal3d.grid.quad. QuadPortrayal 
  
   getInspector(LocationWrapper, GUIState)   - 
Method in class sim.portrayal3d.simple. CircledPortrayal3D 
  
   getInspector(LocationWrapper, GUIState)   - 
Method in class sim.portrayal3d.simple. LabelledPortrayal3D 
  
   getInspector(LocationWrapper, GUIState)   - 
Method in class sim.portrayal3d.simple. SharedPortrayal3D 
 Unlikely to be called, as SharedPortrayal3D is not pickable.
   getInspector(LocationWrapper, GUIState)   - 
Method in class sim.portrayal3d.simple. TransformedPortrayal3D 
  
   getInspector(LocationWrapper, GUIState)   - 
Method in class sim.portrayal3d.simple. ValuePortrayal3D 
  
   getInspector(LocationWrapper, GUIState)   - 
Method in class sim.portrayal3d. SimplePortrayal3D 
  
   getLabel(Edge, EdgeDrawInfo2D)   - 
Method in class sim.portrayal.network. SimpleEdgePortrayal2D 
 Returns a name appropriate for the edge.
   getLabel(Object, DrawInfo2D)   - 
Method in class sim.portrayal.simple. LabelledPortrayal2D 
 Returns a name appropriate for the object.
   getLabel(Object, TransformGroup)   - 
Method in class sim.portrayal3d.simple. LabelledPortrayal3D 
 Returns a name appropriate for the object.
   getLabelScale()   - 
Method in class sim.portrayal3d.simple. LabelledPortrayal3D 
  
   getLabelScaling()   - 
Method in class sim.portrayal.network. SimpleEdgePortrayal2D 
  
   getLabelScaling()   - 
Method in class sim.portrayal.simple. LabelledPortrayal2D 
  
   getLastImage()   - 
Method in class sim.display3d. CapturingCanvas3D 
  
   getLength()   - 
Method in class sim.field.continuous. Continuous3D 
 Get the height
   getLength()   - 
Method in class sim.field.grid. AbstractGrid3D 
  
   getLength()   - 
Method in interface sim.field.grid. Grid3D 
 Get the length
   getLength()   - 
Method in class sim.field.grid. SparseGrid3D 
 Returns the length of the grid
   getLength()   - 
Method in class sim.portrayal.simple. TrailedPortrayal2D 
 Returns the length of the trail in TIME.
   getLocation()   - 
Method in class sim.field. SparseField.LocationAndIndex 
  
   getLocation()   - 
Method in class sim.portrayal. LocationWrapper 
 Override this to provide the current location
   getLocationName()   - 
Method in class sim.portrayal. LocationWrapper 
 Override this to provide the current location's name
   getLocationOfObjectAsVector3d(Object, Vector3d)   - 
Method in class sim.portrayal3d.continuous. ContinuousPortrayal3D 
  
   getLocationOfObjectAsVector3d(Object, Vector3d)   - 
Method in class sim.portrayal3d.grid. SparseGridPortrayal3D 
  
   getLocationOfObjectAsVector3d(Object, Vector3d)   - 
Method in class sim.portrayal3d. SparseFieldPortrayal3D 
 Converts a given location (perhaps a Double3D, Double2D, Int3D, or Int2D) into a Vector3d,
        placing it in the given Vector3d, and returning that Vector3d.
   getLocationPosition(Object, DrawInfo2D)   - 
Method in class sim.portrayal.continuous. ContinuousPortrayal2D 
  
   getLocationPosition(Object, DrawInfo2D)   - 
Method in class sim.portrayal. FieldPortrayal2D 
 Returns the position on-screen of the provided location in the underlying field.
   getLocationPosition(Object, DrawInfo2D)   - 
Method in class sim.portrayal.grid. HexaObjectGridPortrayal2D 
  
   getLocationPosition(Object, DrawInfo2D)   - 
Method in class sim.portrayal.grid. HexaSparseGridPortrayal2D 
  
   getLocationPosition(Object, DrawInfo2D)   - 
Method in class sim.portrayal.grid. HexaValueGridPortrayal2D 
  
   getLocationPosition(Object, DrawInfo2D)   - 
Method in class sim.portrayal.grid. ObjectGridPortrayal2D 
  
   getLocationPosition(Object, DrawInfo2D)   - 
Method in class sim.portrayal.grid. SparseGridPortrayal2D 
  
   getLocationPosition(Object, DrawInfo2D)   - 
Method in class sim.portrayal.grid. ValueGridPortrayal2D 
  
   getMap()   - 
Method in class sim.portrayal.grid. FastHexaObjectGridPortrayal2D 
  
   getMap()   - 
Method in class sim.portrayal.grid. FastObjectGridPortrayal2D 
  
   getMap()   - 
Method in class sim.portrayal.grid. ValueGridPortrayal2D 
  
   getMap()   - 
Method in class sim.portrayal3d.grid.quad. QuadPortrayal 
  
   getMap()   - 
Method in class sim.portrayal3d.grid. ValueGridPortrayal3D 
  
   getMax()   - 
Method in class sim.util. Interval 
  
   getMaximumJump()   - 
Method in class sim.portrayal.simple. TrailedPortrayal2D 
 Returns the maximum percentage of either the width or height of the field that can be 
        jumped between two successive object locations before it's considered to be a huge leap and that segment won't be drawn.
   getMaximumPropertiesForInspector()   - 
Method in class sim.display. GUIState 
  
   getMaxProperties()   - 
Method in class sim.portrayal. SimpleInspector 
  
   getMin()   - 
Method in class sim.util. Interval 
  
   getMinimumSize()   - 
Method in class sim.display. Display2D.InnerDisplay2D 
 Overloaded to return (width * scale, height * scale)
   getMinimumSize()   - 
Method in class sim.util.gui. MiniHistogram 
  
   getMinimumSize()   - 
Method in class sim.util.gui. PropertyField 
  
   getMinKey()   - 
Method in class sim.util. Heap 
 Returns the key value of the current min element.
   getModel(Object, TransformGroup)   - 
Method in class sim.portrayal3d. FieldPortrayal3D 
 Produces the requested model.
   getModel(Object, TransformGroup)   - 
Method in interface sim.portrayal3d. Portrayal3D 
 Provides a TransformGroup which defines the node(s) to place in
 the scenegraph.
   getModel(Object, TransformGroup)   - 
Method in class sim.portrayal3d.simple. AxesPortrayal3D 
  
   getModel(Object, TransformGroup)   - 
Method in class sim.portrayal3d.simple. BranchGroupPortrayal3D 
  
   getModel(Object, TransformGroup)   - 
Method in class sim.portrayal3d.simple. CircledPortrayal3D 
  
   getModel(Object, TransformGroup)   - 
Method in class sim.portrayal3d.simple. CubePortrayal3D 
  
   getModel(Object, TransformGroup)   - 
Method in class sim.portrayal3d.simple. ImagePortrayal3D 
  
   getModel(Object, TransformGroup)   - 
Method in class sim.portrayal3d.simple. LabelledPortrayal3D 
  
   getModel(Object, TransformGroup)   - 
Method in class sim.portrayal3d.simple. LightPortrayal3D 
  
   getModel(Object, TransformGroup)   - 
Method in class sim.portrayal3d.simple. PrimitivePortrayal3D 
 We suggest that if you wish to override this to change the appearance or scale or transform 
        of the underlying model, do the changes first and THEN call super.getModel(obj, j3dModel).
   getModel(Object, TransformGroup)   - 
Method in class sim.portrayal3d.simple. SharedPortrayal3D 
  
   getModel(Object, TransformGroup)   - 
Method in class sim.portrayal3d.simple. TransformedPortrayal3D 
  
   getModel(Object, TransformGroup)   - 
Method in class sim.portrayal3d.simple. ValuePortrayal3D 
  
   getModel(Object, TransformGroup)   - 
Method in class sim.portrayal3d.simple. WireFrameBoxPortrayal3D 
  
   getModel(Object, TransformGroup)   - 
Method in class sim.portrayal3d. SimplePortrayal3D 
  
   getModelInspector()   - 
Method in class sim.display. Console 
 Return the model inspector so simulations can do things like updating the properties.
   getMultigraphAdjacencyMatrix()   - 
Method in class sim.field.network. Network 
 Creates and returns a multigraph adjacency matrix, which includes all edges from a given node to another -- if you know for sure
        that you have a simple graph (no multiple edges between two nodes), use getAdjacencyMatrix instead.
   getName(Class)   - 
Static method in class sim.display. GUIState 
 Call this method to get the simulation name for any class.
   getName()   - 
Static method in class sim.display. GUIState 
 Override this method in your subclass to provide a descriptive 
        name for your simulation;
        otherwise the default will be used: the short classname (that is,
        if your class is foo.bar.Baz, Baz will be used).
   getName(LocationWrapper)   - 
Method in class sim.portrayal. FieldPortrayal 
  
   getName(LocationWrapper)   - 
Method in class sim.portrayal.network. SimpleEdgePortrayal2D 
  
   getName(LocationWrapper)   - 
Method in interface sim.portrayal. Portrayal 
 Returns a static, one-line name for the given object that is useful for a human
        to distinguish it from other objects.
   getName(LocationWrapper)   - 
Method in class sim.portrayal.simple. AdjustablePortrayal2D 
  
   getName(LocationWrapper)   - 
Method in class sim.portrayal.simple. CircledPortrayal2D 
  
   getName(LocationWrapper)   - 
Method in class sim.portrayal.simple. FacetedPortrayal2D 
 If portrayAllChildren, Calls on the first child to return the name.
   getName(LocationWrapper)   - 
Method in class sim.portrayal.simple. LabelledPortrayal2D 
  
   getName(LocationWrapper)   - 
Method in class sim.portrayal.simple. MovablePortrayal2D 
  
   getName(LocationWrapper)   - 
Method in class sim.portrayal.simple. OrientedPortrayal2D 
  
   getName(LocationWrapper)   - 
Method in class sim.portrayal.simple. TrailedPortrayal2D 
  
   getName(LocationWrapper)   - 
Method in class sim.portrayal.simple. TransformedPortrayal2D 
  
   getName(LocationWrapper)   - 
Method in class sim.portrayal.simple. ValuePortrayal2D 
  
   getName()   - 
Method in class sim.portrayal. SimpleInspector 
  
   getName(LocationWrapper)   - 
Method in class sim.portrayal. SimplePortrayal2D 
  
   getName(LocationWrapper)   - 
Method in class sim.portrayal3d.grid.quad. QuadPortrayal 
  
   getName(LocationWrapper)   - 
Method in class sim.portrayal3d.simple. CircledPortrayal3D 
  
   getName(LocationWrapper)   - 
Method in class sim.portrayal3d.simple. LabelledPortrayal3D 
  
   getName(LocationWrapper)   - 
Method in class sim.portrayal3d.simple. SharedPortrayal3D 
 Unlikely to be called, as SharedPortrayal3D is not pickable.
   getName(LocationWrapper)   - 
Method in class sim.portrayal3d.simple. TransformedPortrayal3D 
  
   getName(LocationWrapper)   - 
Method in class sim.portrayal3d.simple. ValuePortrayal3D 
  
   getName(LocationWrapper)   - 
Method in class sim.portrayal3d. SimplePortrayal3D 
  
   getName(int)   - 
Method in class sim.util. CollectionProperties 
  
   getName()   - 
Method in class sim.util.media.chart. SeriesAttributes 
 Returns the name of the series.
   getName(int)   - 
Method in class sim.util. Properties 
 Returns the name of the property at the given index.
   getName(int)   - 
Method in class sim.util. SimpleProperties 
 Returns the name of the given property.
   getNearestNeighbors(Double2D, int, boolean, boolean, boolean, Bag)   - 
Method in class sim.field.continuous. Continuous2D 
 Finds and returns at LEAST the 'atleastThisMany' items closest to a given 'position', plus potentially other items.
   getNeighborsAndCorrespondingPositionsHamiltonianDistance(int, int, int, boolean, Bag, IntBag, IntBag)   - 
Method in class sim.field.grid. SparseGrid2D 
 Gets all neighbors of a location that satisfy abs(x-X) + abs(y-Y)    getNeighborsAndCorrespondingPositionsHexagonalDistance(int, int, int, boolean, Bag, IntBag, IntBag)   - 
Method in class sim.field.grid. SparseGrid2D 
 Gets all neighbors located within the hexagon centered at (X,Y) and 2*dist+1 cells from point to opposite point 
 inclusive.
   getNeighborsAndCorrespondingPositionsMaxDistance(int, int, int, boolean, Bag, IntBag, IntBag)   - 
Method in class sim.field.grid. SparseGrid2D 
 Gets all neighbors of a location that satisfy max( abs(x-X) , abs(y-Y) )    getNeighborsHamiltonianDistance(int, int, int, boolean, IntBag, IntBag)   - 
Method in class sim.field.grid. AbstractGrid2D 
  
   getNeighborsHamiltonianDistance(int, int, int, int, boolean, IntBag, IntBag, IntBag)   - 
Method in class sim.field.grid. AbstractGrid3D 
  
   getNeighborsHamiltonianDistance(int, int, int, boolean, Bag, IntBag, IntBag)   - 
Method in class sim.field.grid. DenseGrid2D 
 Gets all neighbors of a location that satisfy abs(x-X) + abs(y-Y)    getNeighborsHamiltonianDistance(int, int, int, boolean, DoubleBag, IntBag, IntBag)   - 
Method in class sim.field.grid. DoubleGrid2D 
 Gets all neighbors of a location that satisfy abs(x-X) + abs(y-Y)    getNeighborsHamiltonianDistance(int, int, int, int, boolean, DoubleBag, IntBag, IntBag, IntBag)   - 
Method in class sim.field.grid. DoubleGrid3D 
 Gets all neighbors of a location that satisfy abs(x-X) + abs(y-Y) + abs(z-Z)    getNeighborsHamiltonianDistance(int, int, int, boolean, IntBag, IntBag)   - 
Method in interface sim.field.grid. Grid2D 
 Gets all neighbors of a location that satisfy abs(x-X) + abs(y-Y)    getNeighborsHamiltonianDistance(int, int, int, int, boolean, IntBag, IntBag, IntBag)   - 
Method in interface sim.field.grid. Grid3D 
 Gets all neighbors of a location that satisfy abs(x-X) + abs(y-Y) + abs(z-Z)    getNeighborsHamiltonianDistance(int, int, int, boolean, IntBag, IntBag, IntBag)   - 
Method in class sim.field.grid. IntGrid2D 
 Gets all neighbors of a location that satisfy abs(x-X) + abs(y-Y)    getNeighborsHamiltonianDistance(int, int, int, int, boolean, IntBag, IntBag, IntBag, IntBag)   - 
Method in class sim.field.grid. IntGrid3D 
 Gets all neighbors of a location that satisfy abs(x-X) + abs(y-Y) + abs(z-Z)    getNeighborsHamiltonianDistance(int, int, int, boolean, Bag, IntBag, IntBag)   - 
Method in class sim.field.grid. ObjectGrid2D 
 Gets all neighbors of a location that satisfy abs(x-X) + abs(y-Y)    getNeighborsHamiltonianDistance(int, int, int, int, boolean, Bag, IntBag, IntBag, IntBag)   - 
Method in class sim.field.grid. ObjectGrid3D 
 Gets all neighbors of a location that satisfy abs(x-X) + abs(y-Y) + abs(z-Z)    getNeighborsHamiltonianDistance(int, int, int, boolean, IntBag, IntBag)   - 
Method in class sim.field.grid. SparseGrid2D 
  
   getNeighborsHamiltonianDistance(int, int, int, boolean, Bag, IntBag, IntBag)   - 
Method in class sim.field.grid. SparseGrid2D 
 Gets all neighbors of a location that satisfy abs(x-X) + abs(y-Y)    getNeighborsHamiltonianDistance(int, int, int, int, boolean, IntBag, IntBag, IntBag)   - 
Method in class sim.field.grid. SparseGrid3D 
  
   getNeighborsHamiltonianDistance(int, int, int, int, boolean, Bag, IntBag, IntBag, IntBag)   - 
Method in class sim.field.grid. SparseGrid3D 
 Gets all neighbors of a location that satisfy abs(x-X) + abs(y-Y) + abs(z-Z)    getNeighborsHexagonalDistance(int, int, int, boolean, IntBag, IntBag)   - 
Method in class sim.field.grid. AbstractGrid2D 
  
   getNeighborsHexagonalDistance(int, int, int, boolean, Bag, IntBag, IntBag)   - 
Method in class sim.field.grid. DenseGrid2D 
 Gets all neighbors located within the hexagon centered at (X,Y) and 2*dist+1 cells from point to opposite point 
 inclusive.
   getNeighborsHexagonalDistance(int, int, int, boolean, DoubleBag, IntBag, IntBag)   - 
Method in class sim.field.grid. DoubleGrid2D 
 Gets all neighbors located within the hexagon centered at (X,Y) and 2*dist+1 cells from point to opposite point 
 inclusive.
   getNeighborsHexagonalDistance(int, int, int, boolean, IntBag, IntBag)   - 
Method in interface sim.field.grid. Grid2D 
 Gets all neighbors located within the hexagon centered at (X,Y) and 2*dist+1 cells from point to opposite point 
 inclusive.
   getNeighborsHexagonalDistance(int, int, int, boolean, IntBag, IntBag, IntBag)   - 
Method in class sim.field.grid. IntGrid2D 
 Gets all neighbors located within the hexagon centered at (X,Y) and 2*dist+1 cells from point to opposite point 
 inclusive.
   getNeighborsHexagonalDistance(int, int, int, boolean, Bag, IntBag, IntBag)   - 
Method in class sim.field.grid. ObjectGrid2D 
 Gets all neighbors located within the hexagon centered at (X,Y) and 2*dist+1 cells from point to opposite point 
 inclusive.
   getNeighborsHexagonalDistance(int, int, int, boolean, IntBag, IntBag)   - 
Method in class sim.field.grid. SparseGrid2D 
  
   getNeighborsHexagonalDistance(int, int, int, boolean, Bag, IntBag, IntBag)   - 
Method in class sim.field.grid. SparseGrid2D 
 Gets all neighbors located within the hexagon centered at (X,Y) and 2*dist+1 cells from point to opposite point 
 inclusive.
   getNeighborsMaxDistance(int, int, int, boolean, IntBag, IntBag)   - 
Method in class sim.field.grid. AbstractGrid2D 
  
   getNeighborsMaxDistance(int, int, int, int, boolean, IntBag, IntBag, IntBag)   - 
Method in class sim.field.grid. AbstractGrid3D 
  
   getNeighborsMaxDistance(int, int, int, boolean, Bag, IntBag, IntBag)   - 
Method in class sim.field.grid. DenseGrid2D 
 Gets all neighbors of a location that satisfy max( abs(x-X) , abs(y-Y) )    getNeighborsMaxDistance(int, int, int, boolean, DoubleBag, IntBag, IntBag)   - 
Method in class sim.field.grid. DoubleGrid2D 
 Gets all neighbors of a location that satisfy max( abs(x-X) , abs(y-Y) )    getNeighborsMaxDistance(int, int, int, int, boolean, DoubleBag, IntBag, IntBag, IntBag)   - 
Method in class sim.field.grid. DoubleGrid3D 
 Gets all neighbors of a location that satisfy max( abs(x-X) , abs(y-Y), abs(z-Z) )    getNeighborsMaxDistance(int, int, int, boolean, IntBag, IntBag)   - 
Method in interface sim.field.grid. Grid2D 
 Gets all neighbors of a location that satisfy max( abs(x-X) , abs(y-Y) )    getNeighborsMaxDistance(int, int, int, int, boolean, IntBag, IntBag, IntBag)   - 
Method in interface sim.field.grid. Grid3D 
 Gets all neighbors of a location that satisfy max( abs(x-X) , abs(y-Y), abs(z-Z) )    getNeighborsMaxDistance(int, int, int, boolean, IntBag, IntBag, IntBag)   - 
Method in class sim.field.grid. IntGrid2D 
 Gets all neighbors of a location that satisfy max( abs(x-X) , abs(y-Y) )    getNeighborsMaxDistance(int, int, int, int, boolean, IntBag, IntBag, IntBag, IntBag)   - 
Method in class sim.field.grid. IntGrid3D 
 Gets all neighbors of a location that satisfy max( abs(x-X) , abs(y-Y), abs(z-Z) )    getNeighborsMaxDistance(int, int, int, boolean, Bag, IntBag, IntBag)   - 
Method in class sim.field.grid. ObjectGrid2D 
 Gets all neighbors of a location that satisfy max( abs(x-X) , abs(y-Y) )    getNeighborsMaxDistance(int, int, int, int, boolean, Bag, IntBag, IntBag, IntBag)   - 
Method in class sim.field.grid. ObjectGrid3D 
 Gets all neighbors of a location that satisfy max( abs(x-X) , abs(y-Y), abs(z-Z) )    getNeighborsMaxDistance(int, int, int, boolean, IntBag, IntBag)   - 
Method in class sim.field.grid. SparseGrid2D 
  
   getNeighborsMaxDistance(int, int, int, boolean, Bag, IntBag, IntBag)   - 
Method in class sim.field.grid. SparseGrid2D 
 Gets all neighbors of a location that satisfy max( abs(x-X) , abs(y-Y) )    getNeighborsMaxDistance(int, int, int, int, boolean, IntBag, IntBag, IntBag)   - 
Method in class sim.field.grid. SparseGrid3D 
  
   getNeighborsMaxDistance(int, int, int, int, boolean, Bag, IntBag, IntBag, IntBag)   - 
Method in class sim.field.grid. SparseGrid3D 
 Gets all neighbors of a location that satisfy max( abs(x-X) , abs(y-Y), abs(z-Z) )    getNeighborsWithinArc(int, int, double, double, double, IntBag, IntBag)   - 
Method in class sim.field.grid. AbstractGrid2D 
  
   getNeighborsWithinArc(int, int, double, double, double, boolean, IntBag, IntBag)   - 
Method in class sim.field.grid. AbstractGrid2D 
  
   getNodeIndex(Object)   - 
Method in class sim.field.network. Network 
  
   getNumBins()   - 
Method in class sim.util.media.chart. HistogramSeriesAttributes 
  
   getNumGlobalAttributes()   - 
Method in class sim.util.media.chart. ChartGenerator 
 Returns the number of global attribute panels.
   getNumSeriesAttributes()   - 
Method in class sim.util.media.chart. ChartGenerator 
  
   getNumStepsPerStepButtonPress()   - 
Method in class sim.display. Console 
  
   getObject()   - 
Method in class sim.portrayal. LocationWrapper 
 Override this to provide the current object
   getObject()   - 
Method in class sim.portrayal. SimpleInspector 
  
   getObject()   - 
Method in class sim.util. Properties 
 Returns the original object from which the properties are extracted
   getObjectIndex(Object)   - 
Method in class sim.field. SparseField 
 Returns the index of the object in the allObjects Bag, if the object exists, else returns -1.
   getObjectLocation(Object)   - 
Method in class sim.field.continuous. Continuous2D 
  
   getObjectLocation(Object)   - 
Method in class sim.field.continuous. Continuous3D 
  
   getObjectLocation(Object)   - 
Method in class sim.field.grid. SparseGrid2D 
 Returns the object location, or null if there is no such object.
   getObjectLocation(Object)   - 
Method in class sim.field.grid. SparseGrid3D 
 Returns the object location, or null if there is no such object.
   getObjectLocation(Object, GUIState)   - 
Method in class sim.portrayal.continuous. ContinuousPortrayal2D 
  
   getObjectLocation(Object, GUIState)   - 
Method in class sim.portrayal. FieldPortrayal2D 
 Returns the first location in the underlying field of the given object, if such a thing
        is reasonable.
   getObjectLocation(Object, GUIState)   - 
Method in class sim.portrayal.grid. ObjectGridPortrayal2D 
  
   getObjectLocation(Object, GUIState)   - 
Method in class sim.portrayal.grid. SparseGridPortrayal2D 
  
   getObjectLocation(Object)   - 
Method in class sim.portrayal.network. SpatialNetwork2D 
  Deprecated.     
   getObjectLocationAsDouble2D(Object)   - 
Method in class sim.field.continuous. Continuous2D 
 Synonymous with getObjectLocation, which you should generally use instead.
   getObjectLocationAsDouble2D(Object)   - 
Method in class sim.field.grid. SparseGrid2D 
 Returns the object location as a Double2D, or as null if there is no such object.
   getObjectLocationAsDouble2D(Object)   - 
Method in interface sim.field. SparseField2D 
 Returns the location of an object in the sparse field as a Double2D
   getObjectLocationAsDouble3D(Object)   - 
Method in class sim.field.continuous. Continuous3D 
 Returns the object location as a Double3D, or as null if there is no such object.
   getObjectLocationAsDouble3D(Object)   - 
Method in class sim.field.grid. SparseGrid3D 
 Returns the object location as a Double3D, or as null if there is no such object.
   getObjectLocationAsDouble3D(Object)   - 
Method in interface sim.field. SparseField3D 
 Returns the location of an object in the sparse field as a Double3D
   getObjectPosition(Object, DrawInfo2D)   - 
Method in class sim.portrayal. FieldPortrayal2D 
 Returns the position-onscreen of the provided object, assuming that the object exists within the underlying field.
   getObjectsAtDiscretizedLocation(Int2D)   - 
Method in class sim.field.continuous. Continuous2D 
 Returns a bag containing all the objects at a given discretized location, or null when there are no objects at the location.
   getObjectsAtDiscretizedLocation(Int3D)   - 
Method in class sim.field.continuous. Continuous3D 
 Returns a bag containing all the objects at a given discretized location, or null when there are no objects at the location.
   getObjectsAtLocation(Double2D)   - 
Method in class sim.field.continuous. Continuous2D 
 Returns a bag containing all the objects at a given location, 
        or null if there are no such objects or if location is null.
   getObjectsAtLocation(Double3D)   - 
Method in class sim.field.continuous. Continuous3D 
 Returns a bag containing all the objects at a given location, 
        or null if there are no such objects or if location is null.
   getObjectsAtLocation(int, int)   - 
Method in class sim.field.grid. DenseGrid2D 
 Returns a bag containing all the objects at a given location, or null when there are no objects at the location.
   getObjectsAtLocation(Int2D)   - 
Method in class sim.field.grid. DenseGrid2D 
 Returns a bag containing all the objects at a given location, or null when there are no objects at the location.
   getObjectsAtLocation(int, int)   - 
Method in class sim.field.grid. SparseGrid2D 
 Returns a bag containing all the objects at a given location, or null when there are no objects at the location.
   getObjectsAtLocation(int, int, int)   - 
Method in class sim.field.grid. SparseGrid3D 
 Returns a bag containing all the objects at a given location -- which MIGHT be empty or MIGHT be null
        (which should also be interpreted as "empty") when there are no objects at the location.
   getObjectsAtLocation(Object)   - 
Method in class sim.field. SparseField 
 Returns a bag containing all the objects at a given location, or null when there are no objects at the location.
   getObjectsAtLocationOfObject(Object)   - 
Method in class sim.field.continuous. Continuous2D 
 Returns a bag containing all the objects at the exact same location as a given object, including the object itself, 
        or null if the object is not in the Field.
   getObjectsAtLocationOfObject(Object)   - 
Method in class sim.field.continuous. Continuous3D 
 Returns a bag containing all the objects at the exact same location as a given object, including the object itself, 
        or null if the object is not in the Field.
   getObjectsAtLocationOfObject(Object)   - 
Method in class sim.field. SparseField 
 Returns a bag containing all the objects at the same location as a given object, including the object itself, 
        or null if the object is not in the Field.
   getObjectsAtLocations(IntBag, IntBag, Bag)   - 
Method in class sim.field.grid. SparseGrid2D 
 For each   location, puts all such objects into the result bag.
   getObjectsAtLocations(IntBag, IntBag, IntBag, Bag)   - 
Method in class sim.field.grid. SparseGrid3D 
 For each   location, puts all such objects into the result bag.
   getObjectsAtLocations(Bag, Bag)   - 
Method in class sim.field. SparseField 
 For each location, puts all object at that location into the result bag.
   getObjectsExactlyWithinDistance(Double2D, double)   - 
Method in class sim.field.continuous. Continuous2D 
 Returns a Bag containing EXACTLY those objects within a certain distance of a given position, or equal to that distance, measuring
        using a circle of radius 'distance' around the given position.
   getObjectsExactlyWithinDistance(Double2D, double, boolean)   - 
Method in class sim.field.continuous. Continuous2D 
 Returns a Bag containing EXACTLY those objects within a certain distance of a given position, or equal to that distance, measuring
        using a circle of radius 'distance' around the given position.
   getObjectsExactlyWithinDistance(Double2D, double, boolean, boolean, boolean, Bag)   - 
Method in class sim.field.continuous. Continuous2D 
 Returns a Bag containing EXACTLY those objects within a certain distance of a given position.
   getObjectsExactlyWithinDistance(Double3D, double)   - 
Method in class sim.field.continuous. Continuous3D 
 Returns a Bag containing EXACTLY those objects within a certain distance of a given position, or equal to that distance, measuring
        using a circle of radius 'distance' around the given position.
   getObjectsExactlyWithinDistance(Double3D, double, boolean)   - 
Method in class sim.field.continuous. Continuous3D 
 Returns a Bag containing EXACTLY those objects within a certain distance of a given position, or equal to that distance, measuring
        using a circle of radius 'distance' around the given position.
   getObjectsExactlyWithinDistance(Double3D, double, boolean, boolean, boolean, Bag)   - 
Method in class sim.field.continuous. Continuous3D 
 Returns a Bag containing EXACTLY those objects within a certain distance of a given position.
   getObjectsWithinDistance(Double2D, double)   - 
Method in class sim.field.continuous. Continuous2D 
 Returns a bag containing AT LEAST those objects within the bounding box surrounding the
        specified distance of the specified position.
   getObjectsWithinDistance(Double2D, double, boolean)   - 
Method in class sim.field.continuous. Continuous2D 
 Returns a bag containing AT LEAST those objects within the bounding box surrounding the
        specified distance of the specified position.
   getObjectsWithinDistance(Double2D, double, boolean, boolean)   - 
Method in class sim.field.continuous. Continuous2D 
 Returns a bag containing AT LEAST those objects within the bounding box surrounding the
        specified distance of the specified position.
   getObjectsWithinDistance(Double2D, double, boolean, boolean, Bag)   - 
Method in class sim.field.continuous. Continuous2D 
 Puts into the result Bag (and returns it) AT LEAST those objects within the bounding box surrounding the
        specified distance of the specified position.
   getObjectsWithinDistance(Double3D, double)   - 
Method in class sim.field.continuous. Continuous3D 
 Returns a bag containing AT LEAST those objects within the bounding box surrounding the
        specified distance of the specified position.
   getObjectsWithinDistance(Double3D, double, boolean)   - 
Method in class sim.field.continuous. Continuous3D 
 Returns a bag containing AT LEAST those objects within the bounding box surrounding the
        specified distance of the specified position.
   getObjectsWithinDistance(Double3D, double, boolean, boolean)   - 
Method in class sim.field.continuous. Continuous3D 
 Returns a bag containing AT LEAST those objects within the bounding box surrounding the
         specified distance of the specified position.
   getObjectsWithinDistance(Double3D, double, boolean, boolean, Bag)   - 
Method in class sim.field.continuous. Continuous3D 
 Puts into the result Bag (and returns it) AT LEAST those objects within the bounding box surrounding the
        specified distance of the specified position.
   getOnlyCircleWhenSelected()   - 
Method in class sim.portrayal.simple. CircledPortrayal2D 
  
   getOnlyCircleWhenSelected()   - 
Method in class sim.portrayal3d.simple. CircledPortrayal3D 
  
   getOnlyDrawWhenSelected()   - 
Method in class sim.portrayal.simple. OrientedPortrayal2D 
  
   getOnlyGrowTrailWhenSelected()   - 
Method in class sim.portrayal.simple. TrailedPortrayal2D 
 Returns whether or not to grow the trail only after the objet has been selected, and delete it when the object has been deselected.
   getOnlyLabelWhenSelected()   - 
Method in class sim.portrayal.simple. LabelledPortrayal2D 
  
   getOnlyLabelWhenSelected()   - 
Method in class sim.portrayal3d.simple. LabelledPortrayal3D 
  
   getOnlyShowTrailWhenSelected()   - 
Method in class sim.portrayal.simple. TrailedPortrayal2D 
 Returns whether or not to draw the trail only when the object has been selected (or not).
   getOrdering()   - 
Method in class sim.engine. Schedule.Key 
  
   getOtherNode(Object)   - 
Method in class sim.field.network. Edge 
 Returns the alternate to the provided node.
   getPlaySleep()   - 
Method in class sim.display. Console 
 Gets how long we should sleep between each step in the play thread (in milliseconds).
   getPlayState()   - 
Method in class sim.display. Console 
 Gets whether or not the current thread is PS_PLAYING, PS_STOPPED, or PS_PAUSED.
   getPlayState()   - 
Method in class sim.display. SimpleController 
 Gets whether or not the current thread is PS_PLAYING, PS_STOPPED, or PS_PAUSED.
   getPlot()   - 
Method in class sim.util.media.chart. SeriesAttributes 
 Returns the Chart's Plot cast into an XYPlot.
   getPortrayalForAll()   - 
Method in class sim.portrayal. FieldPortrayal 
  
   getPortrayalForNonNull()   - 
Method in class sim.portrayal. FieldPortrayal 
  
   getPortrayalForNull()   - 
Method in class sim.portrayal. FieldPortrayal 
  
   getPortrayalForObject(Object)   - 
Method in class sim.portrayal. FieldPortrayal 
 Returns the appropriate Portrayal.
   getPortrayalForRemainder()   - 
Method in class sim.portrayal. FieldPortrayal 
  
   getPositionLocation(Point2D.Double, DrawInfo2D)   - 
Method in class sim.portrayal.continuous. ContinuousPortrayal2D 
  
   getPositionLocation(Point2D.Double, DrawInfo2D)   - 
Method in class sim.portrayal. FieldPortrayal2D 
 Returns the Location, in the parlance of the underlying Field, of the given position.
   getPositionLocation(Point2D.Double, DrawInfo2D)   - 
Method in class sim.portrayal.grid. HexaObjectGridPortrayal2D 
  
   getPositionLocation(Point2D.Double, DrawInfo2D)   - 
Method in class sim.portrayal.grid. HexaSparseGridPortrayal2D 
  
   getPositionLocation(Point2D.Double, DrawInfo2D)   - 
Method in class sim.portrayal.grid. HexaValueGridPortrayal2D 
  
   getPositionLocation(Point2D.Double, DrawInfo2D)   - 
Method in class sim.portrayal.grid. ObjectGridPortrayal2D 
  
   getPositionLocation(Point2D.Double, DrawInfo2D)   - 
Method in class sim.portrayal.grid. SparseGridPortrayal2D 
  
   getPositionLocation(Point2D.Double, DrawInfo2D)   - 
Method in class sim.portrayal.grid. ValueGridPortrayal2D 
  
   getPositiveWeight(Object, EdgeDrawInfo2D)   - 
Method in class sim.portrayal.network. SimpleEdgePortrayal2D 
 Returns a weight appropriate to scale the edge.
   getPreferencesKey()   - 
Method in class sim.display. Display2D 
  
   getPreferencesKey()   - 
Method in class sim.display3d. Display3D 
  
   getPreferredScrollableViewportSize()   - 
Method in class sim.util.gui. AbstractScrollable 
  
   getPreferredSize()   - 
Method in class sim.display. Display2D.InnerDisplay2D 
 Overloaded to return (width * scale, height * scale)
   getPreferredSize()   - 
Method in class sim.util.gui. MiniHistogram 
  
   getPreferredSize()   - 
Method in class sim.util.gui. PropertyField 
  
   getProperties(Object)   - 
Static method in class sim.util. Properties 
 Returns a Properties object for the given object.
   getProperties(Object, boolean, boolean, boolean)   - 
Static method in class sim.util. Properties 
  Deprecated.   use the full version 
   getProperties(Object, boolean, boolean, boolean, boolean)   - 
Static method in class sim.util. Properties 
 Returns a Properties object for the given object.
   getRangeAxisLabel()   - 
Method in class sim.util.media.chart. ChartGenerator 
  Deprecated.   Returns the name of the Range Axis Label -- usually this is the Y axis. 
   getRawObjectLocation(Object)   - 
Method in class sim.field. SparseField 
 Get the location of the provided object, or null if the object does not exist.
   getRawObjectsAtLocation(Object)   - 
Method in class sim.field. SparseField 
 This method is called by getObjectsAtLocation(location) so you can override getObjectsAtLocation() to
        customize it in certain ways (which is rare).
   getRelativeObjectPosition(Object, Object, DrawInfo2D)   - 
Method in class sim.portrayal.continuous. ContinuousPortrayal2D 
  
   getRelativeObjectPosition(Object, Object, DrawInfo2D)   - 
Method in class sim.portrayal. FieldPortrayal2D 
 Returns the position on-screen of an object at a given location in the field,
        using another object's location and DrawInfo2D to perform the computation.
   getRenderer()   - 
Method in class sim.util.media.chart. SeriesAttributes 
  
   getRequiresConfirmationToStop()   - 
Method in class sim.display. Console 
  
   getRGB(double)   - 
Method in interface sim.util.gui. ColorMap 
 Returns the RGB values, plus alpha, for a color for the given level.
   getRGB(double)   - 
Method in class sim.util.gui. SimpleColorMap 
  
   getScale()   - 
Method in class sim.display. Display2D 
 Returns the scale (the zoom value) of the Display2D
   getScale()   - 
Method in class sim.display3d. Display3D 
 Returns the value of the scale (magnification).
   getScale(DrawInfo2D)   - 
Method in class sim.portrayal.continuous. ContinuousPortrayal2D 
  
   getScale(DrawInfo2D)   - 
Method in class sim.portrayal. FieldPortrayal2D 
 Returns the width and height, in pixels, of 1.0 x 1.0 units in the underlying field.
   getScale(DrawInfo2D)   - 
Method in class sim.portrayal.grid. HexaObjectGridPortrayal2D 
  
   getScale(DrawInfo2D)   - 
Method in class sim.portrayal.grid. HexaSparseGridPortrayal2D 
  
   getScale(DrawInfo2D)   - 
Method in class sim.portrayal.grid. HexaValueGridPortrayal2D 
  
   getScale(DrawInfo2D)   - 
Method in class sim.portrayal.grid. ObjectGridPortrayal2D 
  
   getScale(DrawInfo2D)   - 
Method in class sim.portrayal.grid. SparseGridPortrayal2D 
  
   getScale(DrawInfo2D)   - 
Method in class sim.portrayal.grid. ValueGridPortrayal2D 
  
   getScale()   - 
Method in class sim.portrayal3d.grid. ValueGridPortrayal3D 
  
   getScale2D()   - 
Method in interface sim.portrayal. Scalable2D 
  
   getScaling()   - 
Method in class sim.portrayal.network. SimpleEdgePortrayal2D 
  
   getScrollableBlockIncrement(Rectangle, int, int)   - 
Method in class sim.util.gui. AbstractScrollable 
  
   getScrollableTracksViewportHeight()   - 
Method in class sim.util.gui. AbstractScrollable 
  
   getScrollableTracksViewportWidth()   - 
Method in class sim.util.gui. AbstractScrollable 
  
   getScrollableUnitIncrement(Rectangle, int, int)   - 
Method in class sim.util.gui. AbstractScrollable 
  
   getSelectionBehavior()   - 
Method in class sim.display3d. Display3D 
  
   getSeriesAttribute(int)   - 
Method in class sim.util.media.chart. ChartGenerator 
  
   getSeriesAttributes()   - 
Method in class sim.util.media.chart. ChartGenerator 
  
   getSeriesDataset()   - 
Method in class sim.util.media.chart. ChartGenerator 
  
   getSeriesIndex()   - 
Method in class sim.util.media.chart. SeriesAttributes 
 Returns the index of the series.
   getSeriesName()   - 
Method in class sim.util.media.chart. TimeSeriesAttributes 
  
   getShape()   - 
Method in class sim.portrayal.network. SimpleEdgePortrayal2D 
 Returns the shape of the edge.
   getShape()   - 
Method in class sim.portrayal.simple. OrientedPortrayal2D 
  
   getShape(TransformGroup, int)   - 
Method in class sim.portrayal3d.simple. PrimitivePortrayal3D 
 Returns the shape by the given index.
   getShape(TransformGroup, int)   - 
Method in class sim.portrayal3d.simple. Shape3DPortrayal3D 
  
   getShape(TransformGroup, int)   - 
Method in class sim.portrayal3d.simple. ValuePortrayal3D 
  
   getShape()   - 
Method in class sim.util.media.chart. ScatterPlotSeriesAttributes 
  
   getShapeNum()   - 
Method in class sim.util.media.chart. ScatterPlotSeriesAttributes 
  
   getShouldRepeat()   - 
Method in class sim.display. Console 
 Get whether or not the simualtion should repeat when the stop button is pressed.
   getSimulation()   - 
Method in class sim.display3d. Display3D 
  
   getSimulationInspectedObject()   - 
Method in class sim.display. GUIState 
 Returns an object with various property methods (getFoo(...), isFoo(...), setFoo(...)) whose
        properties will be accessible by the user.
   getSimulationProperties()   - 
Method in class sim.display. GUIState 
 Override this to provide a custom Properties object for your simuation.
   getSorry()   - 
Method in class sim.portrayal.grid. ObjectGridPortrayal2D.Message 
  
   getStatus(LocationWrapper)   - 
Method in class sim.portrayal. FieldPortrayal 
  
   getStatus(LocationWrapper)   - 
Method in interface sim.portrayal. Portrayal 
 Returns a simple, informative one-line description of the current status of the object,
        which may change at any time to reflect how the object is changing.
   getStatus(LocationWrapper)   - 
Method in class sim.portrayal.simple. ValuePortrayal2D 
  
   getStatus(LocationWrapper)   - 
Method in class sim.portrayal. SimplePortrayal2D 
  
   getStatus(LocationWrapper)   - 
Method in class sim.portrayal3d.grid.quad. QuadPortrayal 
  
   getStatus(LocationWrapper)   - 
Method in class sim.portrayal3d. SimplePortrayal3D 
  
   getSteps()   - 
Method in class sim.engine. Schedule 
 Returns the number of steps the Schedule has pulsed so far.
   getStepsPerSecond()   - 
Method in class sim.display. Console 
 Returns the frame rate.
   getStoppable()   - 
Method in class sim.util.media.chart. SeriesAttributes 
  
   getStretch()   - 
Method in class sim.util.media.chart. TimeSeriesAttributes 
  
   getStrokeColor()   - 
Method in class sim.util.media.chart. HistogramSeriesAttributes 
  
   getStrokeColor()   - 
Method in class sim.util.media.chart. TimeSeriesAttributes 
  
   getStrokeOpacity()   - 
Method in class sim.util.media.chart. HistogramSeriesAttributes 
  
   getSymbolColor()   - 
Method in class sim.util.media.chart. ScatterPlotSeriesAttributes 
  
   getSymbolOpacity()   - 
Method in class sim.util.media.chart. ScatterPlotSeriesAttributes 
  
   getTabPane()   - 
Method in class sim.display. Console 
 Simulations can call this to get access to the tabPane -- 
        to add tabbed panes as they like.
   getThickness()   - 
Method in class sim.util.media.chart. HistogramSeriesAttributes 
  
   getThickness()   - 
Method in class sim.util.media.chart. TimeSeriesAttributes 
  
   getThreadPriority()   - 
Method in class sim.display. Console 
  Deprecated.   We may eliminate thread priority as an option 
   getTime()   - 
Method in class sim.engine. Schedule 
 Returns the current timestep
   getTime()   - 
Method in class sim.engine. Schedule.Key 
  
   getTimestamp(String, String)   - 
Method in class sim.engine. Schedule 
 Returns the current time in string format.
   getTimestamp(double, String, String)   - 
Method in class sim.engine. Schedule 
 Returns a given time in string format.
   getTitle()   - 
Method in class sim.portrayal. Inspector 
 Called by the system to come up with an appropriate title for a free-floating inspector window.
   getTitle()   - 
Method in class sim.portrayal. SimpleInspector 
  
   getTitle()   - 
Method in class sim.util.media.chart. ChartGenerator 
 Returns the title of the chart
   getTo()   - 
Method in class sim.field.network. Edge 
 Returns the "to" object.
   getToolTipBehavior()   - 
Method in class sim.display3d. Display3D 
  
   getToolTipText(MouseEvent)   - 
Method in class sim.display. Display2D.InnerDisplay2D 
  
   getTransform()   - 
Method in class sim.display3d. Display3D 
 Returns a copy of the current global model transform.
   getTransform()   - 
Method in class sim.portrayal3d. FieldPortrayal3D 
 Returns a copy of the current internal transform for the FieldPortrayal3D.
   getTransform()   - 
Method in class sim.portrayal3d.simple. TransformedPortrayal3D 
 Returns a copy of the current internal transform for the TransformedPortrayal3D.
   getTransparency()   - 
Method in class sim.portrayal3d.grid. ValueGrid2DPortrayal3D 
  
   getTruncatedName(Class)   - 
Static method in class sim.display. GUIState 
 Returns the short name of the class.
   getType(int)   - 
Method in class sim.util. CollectionProperties 
  
   getType(int)   - 
Method in class sim.util. Properties 
 Returns the Class (or for primitive objects, the primitive TYPE) of the property at the given index.
   getType(int)   - 
Method in class sim.util. SimpleProperties 
 Returns the return type of the property (see the TYPE_...
   getTypeConversion(Class)   - 
Method in class sim.util. Properties 
  
   getUpdateSteppable()   - 
Method in class sim.portrayal. Inspector 
 Called whenever the system needs to get a Steppable which, when stepped, will update the inspector and
       repaint it.
   getValue()   - 
Method in class sim.portrayal.simple. ValuePortrayal2D.DoubleFilter 
  
   getValue()   - 
Method in class sim.portrayal.simple. ValuePortrayal2D.IntFilter 
  
   getValue()   - 
Method in class sim.portrayal3d.grid.quad. QuadPortrayal.DoubleFilter 
  
   getValue()   - 
Method in class sim.portrayal3d.grid.quad. QuadPortrayal.IntFilter 
  
   getValue()   - 
Method in class sim.portrayal3d.grid.quad. QuadPortrayal.ObjectFilter 
  
   getValue()   - 
Method in class sim.portrayal3d.simple. ValuePortrayal3D.DoubleFilter 
  
   getValue()   - 
Method in class sim.portrayal3d.simple. ValuePortrayal3D.IntFilter 
  
   getValue(int)   - 
Method in class sim.util. Bag 
 identical to get(index)
   getValue(int)   - 
Method in class sim.util. CollectionProperties 
  
   getValue(int)   - 
Method in class sim.util. DoubleBag 
  
   getValue()   - 
Method in class sim.util.gui. NumberTextField 
 Returns the most recently set value.
   getValue()   - 
Method in class sim.util.gui. PropertyField 
 Returns the most recently set value.
   getValue(int)   - 
Method in interface sim.util. Indexed 
 Throws an IndexOutOfBoundsException if index is inappropriate.
   getValue(int)   - 
Method in class sim.util. IntBag 
  
   getValue(int)   - 
Method in class sim.util. Properties 
 Returns the value of the property at the given index.
   getValue(int)   - 
Method in class sim.util. SimpleProperties 
 Returns the current value of the property.
   getValueName()   - 
Method in class sim.portrayal.grid. ValueGridPortrayal2D 
  
   getValueName()   - 
Method in class sim.portrayal3d.grid. ValueGrid2DPortrayal3D 
  
   getValueName()   - 
Method in class sim.portrayal3d.grid. ValueGridPortrayal3D 
  
   getWeight()   - 
Method in class sim.field.network. Edge 
 Returns the weight of the edge.
   getWhenShouldEnd()   - 
Method in class sim.display. Console 
 Get when the simulation should end.
   getWhenShouldEndTime()   - 
Method in class sim.display. Console 
 Get when the simulation should end.
   getWhenShouldPause()   - 
Method in class sim.display. Console 
 Get when the simulation should pause.
   getWhenShouldPauseTime()   - 
Method in class sim.display. Console 
 Get when the simulation should pause.
   getWidth()   - 
Method in class sim.field.continuous. Continuous2D 
 Get the width
   getWidth()   - 
Method in class sim.field.continuous. Continuous3D 
 Get the width
   getWidth()   - 
Method in class sim.field.grid. AbstractGrid2D 
  
   getWidth()   - 
Method in class sim.field.grid. AbstractGrid3D 
  
   getWidth()   - 
Method in interface sim.field.grid. Grid2D 
 Returns the width of the field.
   getWidth()   - 
Method in interface sim.field.grid. Grid3D 
 Get the width
   getWidth()   - 
Method in class sim.field.grid. SparseGrid2D 
 Returns the width of the grid
   getWidth()   - 
Method in class sim.field.grid. SparseGrid3D 
 Returns the width of the grid
   getWrapper(Object)   - 
Method in class sim.portrayal.continuous. ContinuousPortrayal2D 
  
   getWrapper(Object, Int2D)   - 
Method in class sim.portrayal.grid. ObjectGridPortrayal2D 
  
   getWrapper(Object)   - 
Method in class sim.portrayal.grid. SparseGridPortrayal2D 
  
   getWrapper(double, Int2D)   - 
Method in class sim.portrayal.grid. ValueGridPortrayal2D 
  
   getWrapper(Edge)   - 
Method in class sim.portrayal.network. NetworkPortrayal2D 
  
   getX()   - 
Method in class sim.util. Double2D 
  
   getX()   - 
Method in class sim.util. Double3D 
  
   getX()   - 
Method in class sim.util. Int2D 
  
   getX()   - 
Method in class sim.util. Int3D 
  
   getX()   - 
Method in class sim.util. MutableDouble2D 
  
   getX()   - 
Method in class sim.util. MutableDouble3D 
  
   getX()   - 
Method in class sim.util. MutableInt2D 
  
   getX()   - 
Method in class sim.util. MutableInt3D 
  
   getXAxisLabel()   - 
Method in class sim.util.media.chart. ChartGenerator 
 Returns the name of the X Axis label.
   getY()   - 
Method in class sim.util. Double2D 
  
   getY()   - 
Method in class sim.util. Double3D 
  
   getY()   - 
Method in class sim.util. Int2D 
  
   getY()   - 
Method in class sim.util. Int3D 
  
   getY()   - 
Method in class sim.util. MutableDouble2D 
  
   getY()   - 
Method in class sim.util. MutableDouble3D 
  
   getY()   - 
Method in class sim.util. MutableInt2D 
  
   getY()   - 
Method in class sim.util. MutableInt3D 
  
   getYAxisLabel()   - 
Method in class sim.util.media.chart. ChartGenerator 
 Returns the name of the Y Axis label.
   getZ()   - 
Method in class sim.util. Double3D 
  
   getZ()   - 
Method in class sim.util. Int3D 
  
   getZ()   - 
Method in class sim.util. MutableDouble3D 
  
   getZ()   - 
Method in class sim.util. MutableInt3D 
  
   getZScale()   - 
Method in class sim.portrayal3d.grid.quad. QuadPortrayal 
  
   globalAttributes   - 
Variable in class sim.util.media.chart. ChartGenerator 
 A holder for global attributes components
   globalModelTransformGroup   - 
Variable in class sim.display3d. Display3D 
 The TransformGroup which holds the switch holding the portrayal's scene graph models.
   Grid2D   - Interface in  sim.field.grid  Define basic neighborhood functions for 2D Grids.   Grid3D   - Interface in  sim.field.grid  Define basic neighborhood functions for 3D Grids.   group   - 
Variable in class sim.portrayal3d.simple. PrimitivePortrayal3D 
 This is cloned to create the model.
   gui   - 
Variable in class sim.portrayal. DrawInfo2D 
  
   guirandom   - 
Variable in class sim.display. GUIState 
 An additional random number generator available for GUI and drawing purposes,
        separate from the one used in the model.
   GUIState   - Class in  sim.display  A wrapper for SimState and Schedule which provides additional functionality for
    GUI objects.   GUIState(SimState)   - 
Constructor for class sim.display. GUIState 
 You may optionally override this constructor to call  super(state)  but you should
        be sure to override the no-argument GUIState() constructor as stipulated.
 
 
    
 H  
 
   halt(boolean)   - 
Method in class sim.engine. AsynchronousSteppable 
 This method should cause the loop created in run(...) to die.
   handleMouseEvent(MouseEvent)   - 
Method in class sim.display. Display2D 
  
   handleMouseEvent(GUIState, Manipulating2D, LocationWrapper, MouseEvent, DrawInfo2D, int)   - 
Method in class sim.portrayal.simple. AdjustablePortrayal2D 
  
   handleMouseEvent(GUIState, Manipulating2D, LocationWrapper, MouseEvent, DrawInfo2D, int)   - 
Method in class sim.portrayal.simple. CircledPortrayal2D 
  
   handleMouseEvent(GUIState, Manipulating2D, LocationWrapper, MouseEvent, DrawInfo2D, int)   - 
Method in class sim.portrayal.simple. LabelledPortrayal2D 
  
   handleMouseEvent(GUIState, Manipulating2D, LocationWrapper, MouseEvent, DrawInfo2D, int)   - 
Method in class sim.portrayal.simple. MovablePortrayal2D 
  
   handleMouseEvent(GUIState, Manipulating2D, LocationWrapper, MouseEvent, DrawInfo2D, int)   - 
Method in class sim.portrayal.simple. OrientedPortrayal2D 
  
   handleMouseEvent(GUIState, Manipulating2D, LocationWrapper, MouseEvent, DrawInfo2D, int)   - 
Method in class sim.portrayal.simple. TrailedPortrayal2D 
  
   handleMouseEvent(GUIState, Manipulating2D, LocationWrapper, MouseEvent, DrawInfo2D, int)   - 
Method in class sim.portrayal. SimplePortrayal2D 
 Optionally handles a mouse event.
   hashCode()   - 
Method in class sim.engine. Schedule.Key 
  
   hashCode()   - 
Method in class sim.util. Double2D 
  
   hashCode()   - 
Method in class sim.util. Double3D 
  
   hashCode()   - 
Method in class sim.util. Int2D 
  
   hashCode()   - 
Method in class sim.util. Int3D 
  
   hashCode()   - 
Method in class sim.util. MutableDouble2D 
  
   hashCode()   - 
Method in class sim.util. MutableDouble3D 
  
   hashCode()   - 
Method in class sim.util. MutableInt2D 
  
   hashCode()   - 
Method in class sim.util. MutableInt3D 
  
   header   - 
Variable in class sim.display. Display2D 
 The component bar at the top of the Display2D.
   header   - 
Variable in class sim.display3d. Display3D 
 The component bar at the top of the Display3D.
   Heap   - Class in  sim.util  Implementations of Heap functions in Java.   Heap()   - 
Constructor for class sim.util. Heap 
  
   Heap(Comparable[], Object[])   - 
Constructor for class sim.util. Heap 
  
   height   - 
Variable in class sim.display. Display2D.InnerDisplay2D 
 The height of the display when the scale is 1.0
   height   - 
Variable in class sim.field.continuous. Continuous2D 
  
   height   - 
Variable in class sim.field.continuous. Continuous3D 
  
   height   - 
Variable in class sim.field.grid. AbstractGrid2D 
  
   height   - 
Variable in class sim.field.grid. AbstractGrid3D 
  
   height   - 
Variable in class sim.field.grid. SparseGrid2D 
  
   height   - 
Variable in class sim.field.grid. SparseGrid3D 
  
   HexagonalPortrayal2D   - Class in  sim.portrayal.simple  A simple portrayal for 2D visualization of hexagons.   HexagonalPortrayal2D()   - 
Constructor for class sim.portrayal.simple. HexagonalPortrayal2D 
  
   HexagonalPortrayal2D(Paint)   - 
Constructor for class sim.portrayal.simple. HexagonalPortrayal2D 
  
   HexagonalPortrayal2D(double)   - 
Constructor for class sim.portrayal.simple. HexagonalPortrayal2D 
  
   HexagonalPortrayal2D(boolean)   - 
Constructor for class sim.portrayal.simple. HexagonalPortrayal2D 
  
   HexagonalPortrayal2D(Paint, double)   - 
Constructor for class sim.portrayal.simple. HexagonalPortrayal2D 
  
   HexagonalPortrayal2D(Paint, boolean)   - 
Constructor for class sim.portrayal.simple. HexagonalPortrayal2D 
  
   HexagonalPortrayal2D(double, boolean)   - 
Constructor for class sim.portrayal.simple. HexagonalPortrayal2D 
  
   HexagonalPortrayal2D(Paint, double, boolean)   - 
Constructor for class sim.portrayal.simple. HexagonalPortrayal2D 
  
   HexaObjectGridPortrayal2D   - Class in  sim.portrayal.grid  Portrayal for hexagonal grids (each cell has six equally-distanced neighbors) containing objects.   HexaObjectGridPortrayal2D()   - 
Constructor for class sim.portrayal.grid. HexaObjectGridPortrayal2D 
  
   HexaSparseGridPortrayal2D   - Class in  sim.portrayal.grid  Portrayal for hexagonal grids (each cell has six equally-distanced neighbors).   HexaSparseGridPortrayal2D()   - 
Constructor for class sim.portrayal.grid. HexaSparseGridPortrayal2D 
  
   HexaSparseGridPortrayal2D(DrawPolicy)   - 
Constructor for class sim.portrayal.grid. HexaSparseGridPortrayal2D 
  Deprecated.   Use setDrawPolicy. 
   HexaValueGridPortrayal2D   - Class in  sim.portrayal.grid  Portrayal for hexagonal grids (each cell has six equally-distanced neighbors) with double-precision real values.   HexaValueGridPortrayal2D()   - 
Constructor for class sim.portrayal.grid. HexaValueGridPortrayal2D 
  
   HexaValueGridPortrayal2D(String)   - 
Constructor for class sim.portrayal.grid. HexaValueGridPortrayal2D 
  
   hideAllFrames()   - 
Method in class sim.display. Console 
 Hides all JFrames registered with the Console.
   HistogramGenerator   - Class in  sim.util.media.chart  TimeSeriesChartGenerator is a ChartGenerator which displays a histogram using the JFreeChart library.   HistogramGenerator()   - 
Constructor for class sim.util.media.chart. HistogramGenerator 
  
   HistogramSeriesAttributes   - Class in  sim.util.media.chart  A SeriesAttributes used for user control of histogram series created with HistogramGenerator.   HistogramSeriesAttributes(ChartGenerator, String, int, double[], int, SeriesChangeListener)   - 
Constructor for class sim.util.media.chart. HistogramSeriesAttributes 
 Produces a HistogramSeriesAttributes object with the given generator, series name, series index,
        and desire to display margin options.
   hitObject(Object, DrawInfo2D)   - 
Method in class sim.portrayal.network. SimpleEdgePortrayal2D 
  
   hitObject(Object, DrawInfo2D)   - 
Method in class sim.portrayal.simple. AdjustablePortrayal2D 
  
   hitObject(Object, DrawInfo2D)   - 
Method in class sim.portrayal.simple. CircledPortrayal2D 
  
   hitObject(Object, DrawInfo2D)   - 
Method in class sim.portrayal.simple. FacetedPortrayal2D 
  
   hitObject(Object, DrawInfo2D)   - 
Method in class sim.portrayal.simple. LabelledPortrayal2D 
  
   hitObject(Object, DrawInfo2D)   - 
Method in class sim.portrayal.simple. MovablePortrayal2D 
  
   hitObject(Object, DrawInfo2D)   - 
Method in class sim.portrayal.simple. OrientedPortrayal2D 
  
   hitObject(Object, DrawInfo2D)   - 
Method in class sim.portrayal.simple. OvalPortrayal2D 
 If drawing area intersects selected area, add last portrayed object to the bag
   hitObject(Object, DrawInfo2D)   - 
Method in class sim.portrayal.simple. RectanglePortrayal2D 
 If drawing area intersects selected area, add last portrayed object to the bag
   hitObject(Object, DrawInfo2D)   - 
Method in class sim.portrayal.simple. ShapePortrayal2D 
  
   hitObject(Object, DrawInfo2D)   - 
Method in class sim.portrayal.simple. TrailedPortrayal2D 
  
   hitObject(Object, DrawInfo2D)   - 
Method in class sim.portrayal.simple. TransformedPortrayal2D 
  
   hitObject(Object, DrawInfo2D)   - 
Method in class sim.portrayal. SimplePortrayal2D 
 Return true if the given object, when drawn, intersects with a provided rectangle, for
        hit testing purposes.
   hitObjects(DrawInfo2D, Bag)   - 
Method in class sim.portrayal. FieldPortrayal2D 
 Adds to the provided Bag LocationWrappers for any objects which
        overlap the provided hit range.
   hitOrDraw(Graphics2D, DrawInfo2D, Bag)   - 
Method in class sim.portrayal.continuous. ContinuousPortrayal2D 
  
   hitOrDraw(Graphics2D, DrawInfo2D, Bag)   - 
Method in class sim.portrayal. FieldPortrayal2D 
 Instead of overriding the draw and hitObjects methods, you can optionally override
        this method to provide  both  the draw(...) and hitObjects(...)
        functionality in a single method, as it's common that these two methods have nearly
        identical code.
   hitOrDraw(Graphics2D, DrawInfo2D, Bag)   - 
Method in class sim.portrayal.grid. FastHexaValueGridPortrayal2D 
  
   hitOrDraw(Graphics2D, DrawInfo2D, Bag)   - 
Method in class sim.portrayal.grid. HexaObjectGridPortrayal2D 
  
   hitOrDraw(Graphics2D, DrawInfo2D, Bag)   - 
Method in class sim.portrayal.grid. HexaSparseGridPortrayal2D 
  
   hitOrDraw(Graphics2D, DrawInfo2D, Bag)   - 
Method in class sim.portrayal.grid. HexaValueGridPortrayal2D 
  
   hitOrDraw(Graphics2D, DrawInfo2D, Bag)   - 
Method in class sim.portrayal.grid. ObjectGridPortrayal2D 
  
   hitOrDraw(Graphics2D, DrawInfo2D, Bag)   - 
Method in class sim.portrayal.grid. SparseGridPortrayal2D 
  
   hitOrDraw(Graphics2D, DrawInfo2D, Bag)   - 
Method in class sim.portrayal.grid. ValueGridPortrayal2D 
  
   hitOrDraw(Graphics2D, DrawInfo2D, Bag)   - 
Method in class sim.portrayal.network. NetworkPortrayal2D 
  
   HTMLBrowser   - Class in  sim.util.gui  HTMLBrowser is a simple web browser which lets the user click on links and which provides
   a Back button when appropriate.   HTMLBrowser(Object)   - 
Constructor for class sim.util.gui. HTMLBrowser 
 Constructs an HTMLBrowser using either an HTML string or a URL
 
 
    
 I  
 
   I_BELLY   - 
Static variable in class sim.util.gui. NumberTextField 
  
   I_BELLY_PRESSED   - 
Static variable in class sim.util.gui. NumberTextField 
  
   I_CLOSE   - 
Static variable in class sim.util.media.chart. SeriesAttributes 
  
   I_CLOSE_PRESSED   - 
Static variable in class sim.util.media.chart. SeriesAttributes 
  
   I_DOWN   - 
Static variable in class sim.util.gui. NumberTextField 
  
   I_DOWN   - 
Static variable in class sim.util.media.chart. SeriesAttributes 
  
   I_DOWN_PRESSED   - 
Static variable in class sim.util.gui. NumberTextField 
  
   I_DOWN_PRESSED   - 
Static variable in class sim.util.media.chart. SeriesAttributes 
  
   I_UP   - 
Static variable in class sim.util.gui. NumberTextField 
  
   I_UP   - 
Static variable in class sim.util.media.chart. SeriesAttributes 
  
   I_UP_PRESSED   - 
Static variable in class sim.util.gui. NumberTextField 
  
   I_UP_PRESSED   - 
Static variable in class sim.util.media.chart. SeriesAttributes 
  
   image   - 
Variable in class sim.portrayal.simple. ImagePortrayal2D 
  
   ImagePortrayal2D   - Class in  sim.portrayal.simple  A simple portrayal for 2D visualization of images.   ImagePortrayal2D(ImageIcon, double)   - 
Constructor for class sim.portrayal.simple. ImagePortrayal2D 
 Creates an ImagePortrayal2D with the image inside the given ImageIcon, and scaled with the given scale.
   ImagePortrayal2D(ImageIcon)   - 
Constructor for class sim.portrayal.simple. ImagePortrayal2D 
 Creates an ImagePortrayal2D with the image inside the given ImageIcon.
   ImagePortrayal2D(Class, String, double)   - 
Constructor for class sim.portrayal.simple. ImagePortrayal2D 
 Creates an ImagePortrayal2D by loading an Image resource using getClass().getResource(), and scaled with the given scale.
   ImagePortrayal2D(Class, String)   - 
Constructor for class sim.portrayal.simple. ImagePortrayal2D 
 Creates an ImagePortrayal2D by loading an Image resource using getClass().getResource()
   ImagePortrayal2D(Image)   - 
Constructor for class sim.portrayal.simple. ImagePortrayal2D 
 Creates an ImagePortrayal2D with the given image.
   ImagePortrayal2D(Image, double)   - 
Constructor for class sim.portrayal.simple. ImagePortrayal2D 
 Creates an ImagePortrayal2D with the given image, and scaled with the given scale..
   ImagePortrayal3D   - Class in  sim.portrayal3d.simple  Portrays objects as a thin flat rectangle displaying a provided Image.   ImagePortrayal3D(Class, String)   - 
Constructor for class sim.portrayal3d.simple. ImagePortrayal3D 
 Constructs a (semi-)transparent, oriented ImagePortrayal3D by loading an Image resource using getClass().getResource()
   ImagePortrayal3D(Class, String, boolean, boolean)   - 
Constructor for class sim.portrayal3d.simple. ImagePortrayal3D 
 Constructs a (semi-)transparent, oriented ImagePortrayal3D by loading an Image resource using getClass().getResource()
   ImagePortrayal3D(ImageIcon)   - 
Constructor for class sim.portrayal3d.simple. ImagePortrayal3D 
 Constructs a (semi-)transparent, oriented ImagePortrayal3D using the provided ImageIcon
   ImagePortrayal3D(ImageIcon, boolean, boolean)   - 
Constructor for class sim.portrayal3d.simple. ImagePortrayal3D 
 Constructs a (semi-)transparent, oriented ImagePortrayal3D using the provided ImageIcon.
   ImagePortrayal3D(Image)   - 
Constructor for class sim.portrayal3d.simple. ImagePortrayal3D 
 Constructs a (semi-)transparent, oriented ImagePortrayal3D
   ImagePortrayal3D(Image, boolean, boolean)   - 
Constructor for class sim.portrayal3d.simple. ImagePortrayal3D 
 Constructs an ImagePortrayal3D
   immutableField   - 
Variable in class sim.portrayal. FieldPortrayal 
  
   in   - 
Variable in class sim.field.network. Network.IndexOutIn 
 Bag containing incoming edges of (entering) the node
   increaseSubsteps(Steppable[])   - 
Method in class sim.display. GUIState 
 Roughly doubles the array size, retaining the existing elements
   index   - 
Variable in class sim.field.network. Network.IndexOutIn 
 Index of the node in the allNodes bag
   Indexed   - Interface in  sim.util  A simple interface (simpler than List) for accessing random-access objects without changing their size.   indexFrom()   - 
Method in class sim.field.network. Edge 
  
   indexOutInHash   - 
Variable in class sim.field.network. Network 
 Hashes Network.IndexOutIn structures by Node.
   indexTo()   - 
Method in class sim.field.network. Edge 
  
   info   - 
Variable in class sim.field.network. Edge 
 Other information (maybe cost) associated with the edge
   inform(String, String, JFrame)   - 
Static method in class sim.util.gui. Utilities 
 Pops up an message dialog box.
   informOfError(Throwable, String, JFrame)   - 
Static method in class sim.util.gui. Utilities 
 Pops up an error dialog box.
   init(Controller)   - 
Method in class sim.display. GUIState 
 Called to initialize (display) windows etc.
   init()   - 
Method in class sim.display. SimApplet 
  
   INITIAL_BAG_SIZE   - 
Static variable in class sim.field.grid. DenseGrid2D 
 The size of an initial bag
   INITIAL_BAG_SIZE   - 
Static variable in class sim.field. SparseField 
 The size of an initial bag
   initialize()   - 
Method in class sim.display3d. ToolTipBehavior 
  
   insideDisplay   - 
Variable in class sim.display. Display2D 
 The 2D display inside the scroll view.
   INSPECT_ICON   - 
Static variable in class sim.portrayal. Inspector 
  
   INSPECT_ICON_P   - 
Static variable in class sim.portrayal. Inspector 
  
   Inspector   - Class in  sim.portrayal  An Inspector is a JPanel containing information about some object,
    and updates its displayed information when updateInspector() is called.   Inspector()   - 
Constructor for class sim.portrayal. Inspector 
  
   Int2D   - Class in  sim.util  Int2D is more or less the same class as java.awt.Point, but it is immutable: once the x and y values are set, they cannot be changed (they're final).   Int2D()   - 
Constructor for class sim.util. Int2D 
  
   Int2D(Point)   - 
Constructor for class sim.util. Int2D 
  
   Int2D(MutableInt2D)   - 
Constructor for class sim.util. Int2D 
  
   Int2D(int, int)   - 
Constructor for class sim.util. Int2D 
  
   Int3D   - Class in  sim.util  Int3D stores three values (x, y, and z) but it is immutable: once the x and y and z values are set, they cannot be changed (they're final).   Int3D()   - 
Constructor for class sim.util. Int3D 
  
   Int3D(int, int, int)   - 
Constructor for class sim.util. Int3D 
  
   Int3D(Int2D)   - 
Constructor for class sim.util. Int3D 
 Explicitly assumes the z value is set to 0
   Int3D(Int2D, int)   - 
Constructor for class sim.util. Int3D 
  
   Int3D(MutableInt2D)   - 
Constructor for class sim.util. Int3D 
  
   Int3D(MutableInt2D, int)   - 
Constructor for class sim.util. Int3D 
  
   IntBag   - Class in  sim.util  Maintains a simple array (objs) of ints and the number of ints (numObjs) in the array
    (the array can be bigger than this number).   IntBag(int)   - 
Constructor for class sim.util. IntBag 
 Creates an IntBag with a given initial capacity.
   IntBag()   - 
Constructor for class sim.util. IntBag 
  
   IntBag(IntBag)   - 
Constructor for class sim.util. IntBag 
 Adds the ints from the other IntBag without copying them.
   IntBag(int[])   - 
Constructor for class sim.util. IntBag 
 Creates an IntBag with the given elements.
   Interval   - Class in  sim.util  Defines an inclusive (closed) interval between two numerical values MIN and MAX.   Interval(long, long)   - 
Constructor for class sim.util. Interval 
  
   Interval(double, double)   - 
Constructor for class sim.util. Interval 
  
   IntGrid2D   - Class in  sim.field.grid  A wrapper for 2D arrays of ints.   IntGrid2D(int, int)   - 
Constructor for class sim.field.grid. IntGrid2D 
  
   IntGrid2D(int, int, int)   - 
Constructor for class sim.field.grid. IntGrid2D 
  
   IntGrid2D(IntGrid2D)   - 
Constructor for class sim.field.grid. IntGrid2D 
  
   IntGrid3D   - Class in  sim.field.grid  A wrapper for 3D arrays of ints.   IntGrid3D(int, int, int)   - 
Constructor for class sim.field.grid. IntGrid3D 
  
   IntGrid3D(int, int, int, int)   - 
Constructor for class sim.field.grid. IntGrid3D 
  
   IntGrid3D(IntGrid3D)   - 
Constructor for class sim.field.grid. IntGrid3D 
  
   intValue()   - 
Method in class sim.util. MutableDouble 
  
   isApplet   - 
Static variable in class sim.display. SimApplet 
  
   isCircleShowing()   - 
Method in class sim.portrayal.simple. CircledPortrayal2D 
  
   isCircleShowing()   - 
Method in class sim.portrayal3d.simple. CircledPortrayal3D 
  
   isClipping()   - 
Method in class sim.display. Display2D 
 Returns true if the Display2D is clipping the drawing area to the user-specified
        height and width
   isComposite(int)   - 
Method in class sim.util. Properties 
 Returns true if the property at the given index is a "Composite" object, meaning it's not a primitive type (double, int, etc.) nor a String.
   isDirected()   - 
Method in class sim.field.network. Network 
  
   isDirtyField()   - 
Method in class sim.portrayal. FieldPortrayal 
  
   isDirtyField()   - 
Method in class sim.portrayal.grid. FastHexaObjectGridPortrayal2D 
  
   isDirtyField()   - 
Method in class sim.portrayal.grid. FastObjectGridPortrayal2D 
  
   isDisclosed()   - 
Method in class sim.util.gui. DisclosurePanel 
  
   isDisplayingToroidally()   - 
Method in class sim.portrayal.continuous. ContinuousPortrayal2D 
 Returns TRUE if the portrayal is displaying objects multiply (in a toroidal fashion)
        if they overlap on the edges of the field.
   isDouble()   - 
Method in class sim.util. Interval 
  
   isDrawFilled()   - 
Method in class sim.portrayal.simple. OrientedPortrayal2D 
  
   isEmpty()   - 
Method in class sim.util. Bag 
  
   isEmpty()   - 
Method in class sim.util. DoubleBag 
  
   isEmpty()   - 
Method in class sim.util. Heap 
  
   isEmpty()   - 
Method in class sim.util. IntBag 
  
   isHidden(int)   - 
Method in class sim.util. Properties 
 Returns true if the class requested that this property be hidden from the user.
   isHidden(int)   - 
Method in class sim.util. SimpleProperties 
  
   isImmutableField()   - 
Method in class sim.portrayal. FieldPortrayal 
 Returns true if the underlying field is assumed to be unchanging -- thus
        there's no reason to update once we're created.
   isInfinite()   - 
Method in class sim.util. MutableDouble 
  
   isInspectorVolatile()   - 
Method in class sim.display. GUIState 
  Deprecated.     
   isLabelShowing()   - 
Method in class sim.portrayal.simple. LabelledPortrayal2D 
  
   isLabelShowing()   - 
Method in class sim.portrayal3d.simple. LabelledPortrayal3D 
  
   isLineShowing()   - 
Method in class sim.portrayal.simple. OrientedPortrayal2D 
  Deprecated.   use isOrientationShowing() 
   isMacOSX   - 
Static variable in class sim.display. Display2D 
 Set to true if we're running on a Mac
   isNaN()   - 
Method in class sim.util. MutableDouble 
  
   isNewMenuAllowed()   - 
Method in class sim.display. Console 
  
   isOrientationHittable()   - 
Method in class sim.portrayal.simple. OrientedPortrayal2D 
 Returns true if the orientation marker can be hit as part of the object.
   isOrientationShowing()   - 
Method in class sim.portrayal.simple. OrientedPortrayal2D 
  
   isReadWrite(int)   - 
Method in class sim.util. CollectionProperties 
  
   isReadWrite(int)   - 
Method in class sim.util. Properties 
 Returns true if the property at the given index is both readable and writable (as opposed to read-only).
   isReadWrite(int)   - 
Method in class sim.util. SimpleProperties 
 Returns whether or not the property can be written as well as read
        Returns false if the index is out of the range [0 ...
   isSealed()   - 
Method in class sim.engine. Schedule 
 Returns whether or not the schedule is sealed (nothing more can be scheduled, even 
        if the schedule isn't at AFTER_SIMULATION yet).
   isSelected(Object)   - 
Method in class sim.portrayal3d. SimplePortrayal3D 
  
   isUsingTriangles()   - 
Method in class sim.portrayal3d.grid. ValueGrid2DPortrayal3D 
  
   isVolatile()   - 
Method in class sim.portrayal. Inspector 
 Returns true (default) if the inspector should be updated every time step.
   isVolatile()   - 
Method in class sim.util. CollectionProperties 
  
   isVolatile()   - 
Method in class sim.util. Properties 
 Returns true if the number or order of properties could change at any time
   isVolatile()   - 
Method in class sim.util. SimpleProperties 
  
   isWindows   - 
Static variable in class sim.display. Display2D 
 Set to true if we're running on Windows
   isXAxisLogScaled()   - 
Method in class sim.util.media.chart. ChartGenerator 
  
   isYAxisLogScaled()   - 
Method in class sim.util.media.chart. ChartGenerator 
  
   iterator()   - 
Method in class sim.field.network. Network 
 Iterates over all objects.
   iterator()   - 
Method in class sim.field. SparseField 
 Iterates over all objects.
   iterator()   - 
Method in class sim.util. Bag 
 NOT fail-fast.
 
 
    
 J  
 
   javaVersion   - 
Static variable in class sim.display. Display2D 
 Set to the version number
   job()   - 
Method in class sim.engine. SimState 
 Returns the job number set by the doLoop(...) facility.
 
 
    
 K  
 
   kill()   - 
Method in class sim.engine. SimState 
 A Steppable on the schedule can call this method to cancel the simulation.
   knob   - 
Static variable in class sim.portrayal.simple. AdjustablePortrayal2D 
  
   KNOB_RADIUS   - 
Static variable in class sim.portrayal.simple. AdjustablePortrayal2D 
  
 
 
    
 L  
 
   label   - 
Variable in class sim.portrayal.simple. LabelledPortrayal2D 
  
   labelFont   - 
Variable in class sim.portrayal.network. SimpleEdgePortrayal2D 
  
   LabelledList   - Class in  sim.util.gui  LabelledList is a JPanel which makes it easy to set up two columns of
    Components, with the right column taking up the extra space if there is any.   LabelledList()   - 
Constructor for class sim.util.gui. LabelledList 
  
   LabelledList(String)   - 
Constructor for class sim.util.gui. LabelledList 
 Creates a Labelled List with a provided border label.
   LabelledPortrayal2D   - Class in  sim.portrayal.simple  A wrapper for other Portrayal2Ds which also draws a textual label.   LabelledPortrayal2D(SimplePortrayal2D, double, double, double, double, Font, int, String, Paint, boolean)   - 
Constructor for class sim.portrayal.simple. LabelledPortrayal2D 
 Draws [x=offsetx, y=offsety] pixels away from the [dx=scalex, dy=scaley] prescaled position of the Portrayal2D, 
        using the SansSerif 10pt font, blue, and left alignment.
   LabelledPortrayal2D(SimplePortrayal2D, String)   - 
Constructor for class sim.portrayal.simple. LabelledPortrayal2D 
 Draws 10 pixels down from the [dx=0, dy=0.5] prescaled position of the Portrayal2D, 
        using the SansSerif 10pt font, blue, and left alignment.
   LabelledPortrayal2D(SimplePortrayal2D, double, String, Paint, boolean)   - 
Constructor for class sim.portrayal.simple. LabelledPortrayal2D 
 Draws 10 pixels down from the [dx=0, dy=scaley] prescaled position of the Portrayal2D, 
        using the SansSerif 10pt font, blue, and left alignment.
   LabelledPortrayal2D(SimplePortrayal2D, String, Paint, boolean)   - 
Constructor for class sim.portrayal.simple. LabelledPortrayal2D 
 Draws 10 pixels down from the [dx=0, dy=0.5] prescaled position of the Portrayal2D, 
        using the SansSerif 10pt font, and left alignment.
   LabelledPortrayal3D   - Class in  sim.portrayal3d.simple  A wrapper for other Portrayal3Ds which also draws a textual label.   LabelledPortrayal3D(SimplePortrayal3D)   - 
Constructor for class sim.portrayal3d.simple. LabelledPortrayal3D 
  
   LabelledPortrayal3D(SimplePortrayal3D, String)   - 
Constructor for class sim.portrayal3d.simple. LabelledPortrayal3D 
  
   LabelledPortrayal3D(SimplePortrayal3D, String, Color, boolean)   - 
Constructor for class sim.portrayal3d.simple. LabelledPortrayal3D 
  
   LabelledPortrayal3D(SimplePortrayal3D, double, Font, String, Color, boolean)   - 
Constructor for class sim.portrayal3d.simple. LabelledPortrayal3D 
  
   LabelledPortrayal3D(SimplePortrayal3D, double, double, double, Font, String, Color, boolean)   - 
Constructor for class sim.portrayal3d.simple. LabelledPortrayal3D 
  
   LabelledPortrayal3D(SimplePortrayal3D, Transform3D, Font, String, Color, boolean)   - 
Constructor for class sim.portrayal3d.simple. LabelledPortrayal3D 
  
   labelPaint   - 
Variable in class sim.portrayal.network. SimpleEdgePortrayal2D 
  
   LARGE_BAG_RATIO   - 
Static variable in class sim.field.grid. DenseGrid2D 
 A bag must be larger than its contents by this ratio to be replaced  replaceLargeBags  is true
   LARGE_BAG_RATIO   - 
Static variable in class sim.field. SparseField 
 A bag must be larger than its contents by this ratio to be replaced  replaceLargeBags  is true
   lastToolTipEvent   - 
Variable in class sim.display. Display2D.InnerDisplay2D 
  
   LAYERS_ICON   - 
Static variable in class sim.display. Display2D 
  
   LAYERS_ICON_P   - 
Static variable in class sim.display. Display2D 
  
   layersbutton   - 
Variable in class sim.display. Display2D 
 The button which pops up the layers menu
   layersbutton   - 
Variable in class sim.display3d. Display3D 
 The button which pops up the layers menu
   length   - 
Variable in class sim.field.continuous. Continuous3D 
  
   length   - 
Variable in class sim.field.grid. AbstractGrid3D 
  
   length   - 
Variable in class sim.field.grid. SparseGrid3D 
  
   length()   - 
Method in class sim.util. Double2D 
 Returns the vector length of the Double2D
   length()   - 
Method in class sim.util. MutableDouble2D 
 Returns the length of the vector.
   length()   - 
Method in class sim.util. MutableDouble3D 
 Returns the length of the vector.
   lengthSq()   - 
Method in class sim.util. Double2D 
 Returns the vector length of the Double2D
   lengthSq()   - 
Method in class sim.util. MutableDouble2D 
 Returns the square of the length of the MutableDouble2D.
   lengthSq()   - 
Method in class sim.util. MutableDouble3D 
 Returns the square of the length of the MutableDouble3D.
   LightPortrayal3D   - Class in  sim.portrayal3d.simple  A simple Portrayal3D which provides ambient, directional, or point light to the scene.   LightPortrayal3D(Color, Double3D)   - 
Constructor for class sim.portrayal3d.simple. LightPortrayal3D 
 Directional Light
   LightPortrayal3D(Color)   - 
Constructor for class sim.portrayal3d.simple. LightPortrayal3D 
 Ambient Light
   LightPortrayal3D(Color, Double3D, float, float, float)   - 
Constructor for class sim.portrayal3d.simple. LightPortrayal3D 
 Point Light.
   LightPortrayal3D(Light)   - 
Constructor for class sim.portrayal3d.simple. LightPortrayal3D 
 Provide your own Light!
   load(SimState)   - 
Method in class sim.display. GUIState 
 Called by the Console when the user is loading in a new state from a checkpoint.
   location   - 
Variable in class sim.portrayal. DrawInfo2D 
  
   location   - 
Variable in class sim.portrayal. LocationWrapper 
 The ORIGINAL location of the object
   locationAndIndexHash   - 
Variable in class sim.field. SparseField 
 LocationAndIndex objects (locations and indexes into the allObjects array) hashed by Object.
   locationBagIterator()   - 
Method in class sim.field. SparseField 
 Iterates [somewhat inefficiently] over all bags of objects grouped by location.
   locationToPass   - 
Variable in class sim.portrayal.grid. ObjectGridPortrayal2D 
  
   locationToPass   - 
Variable in class sim.portrayal.grid. ValueGridPortrayal2D 
  
   LocationWrapper   - Class in  sim.portrayal  A LocationWrapper is used to embody the objects stored in a FieldPortrayal; for
   example, those returned by a hitObjects test on a FieldPortrayal2D.   LocationWrapper(Object, Object, FieldPortrayal)   - 
Constructor for class sim.portrayal. LocationWrapper 
  
   lock   - 
Variable in class sim.engine. Schedule 
  
   longValue()   - 
Method in class sim.util. MutableDouble 
  
   LOWER_PAINT   - 
Static variable in class sim.portrayal.simple. AdjustablePortrayal2D 
  
   LOWER_STROKE   - 
Static variable in class sim.portrayal.simple. AdjustablePortrayal2D 
  
   lowerBound(double)   - 
Method in class sim.field.grid. DoubleGrid2D 
 Thresholds the grid so that values smaller than  toNoLowerThanThisMuch  are changed to  toNoLowerThanThisMuch 
        Returns the modified grid.
   lowerBound(double)   - 
Method in class sim.field.grid. DoubleGrid3D 
 Thresholds the grid so that values smaller than  toNoLowerThanThisMuch  are changed to  toNoLowerThanThisMuch 
        Returns the modified grid.
   lowerBound(int)   - 
Method in class sim.field.grid. IntGrid2D 
 Thresholds the grid so that values smaller than  toNoLowerThanThisMuch  are changed to  toNoLowerThanThisMuch 
        Returns the modified grid.
   lowerBound(int)   - 
Method in class sim.field.grid. IntGrid3D 
 Thresholds the grid so that values smaller than  toNoLowerThanThisMuch  are changed to  toNoLowerThanThisMuch 
        Returns the modified grid.
 
 
    
 M  
 
   main(String[])   - 
Static method in class ec.util. MersenneTwisterFast 
 Tests the code.
   main(String[])   - 
Static method in class sim.display. Console 
 Pops up a window allowing the user to enter in a class name to start a new simulation.
   makeBucketLabels(int, double, double, boolean)   - 
Static method in class sim.util.gui. MiniHistogram 
 Generates a set of  numBuckets  bucket labels appropriate for use in a histogram.
   makeBuckets(double[], int, double, double, boolean)   - 
Static method in class sim.util.gui. MiniHistogram 
 Generates a set of  numBuckets  buckets describing a histogram over the provided values in  vals .
   MakesSimState   - Interface in  sim.engine  An interface for classes capable of creating SimState subclasses.   makeUpdateButton()   - 
Method in class sim.portrayal. Inspector 
 A convenient function to create UpdateButton which you might add to the bottom of the JPanel
        (assuming it still is using BorderLayout).
   manhattanDistance(double, double)   - 
Method in class sim.util. Double2D 
 Returns the manhtattan distance FROM this Double2D TO the specified point
   manhattanDistance(Double2D)   - 
Method in class sim.util. Double2D 
 Returns the manhtattan distance FROM this Double2D TO the specified point
   manhattanDistance(Int2D)   - 
Method in class sim.util. Double2D 
 Returns the manhtattan distance FROM this Double2D TO the specified point
   manhattanDistance(MutableDouble2D)   - 
Method in class sim.util. Double2D 
 Returns the manhtattan distance FROM this Double2D TO the specified point
   manhattanDistance(MutableInt2D)   - 
Method in class sim.util. Double2D 
 Returns the manhtattan distance FROM this Double2D TO the specified point
   manhattanDistance(Point2D)   - 
Method in class sim.util. Double2D 
 Returns the manhtattan distance FROM this Double2D TO the specified point
   manhattanDistance(double, double, double)   - 
Method in class sim.util. Double3D 
 Returns the manhtattan distance FROM this Double3D TO the specified point
   manhattanDistance(Double3D)   - 
Method in class sim.util. Double3D 
 Returns the manhtattan distance FROM this Double3D TO the specified point
   manhattanDistance(Int3D)   - 
Method in class sim.util. Double3D 
 Returns the manhtattan distance FROM this Double3D TO the specified point
   manhattanDistance(MutableDouble3D)   - 
Method in class sim.util. Double3D 
 Returns the manhtattan distance FROM this Double3D TO the specified point
   manhattanDistance(MutableInt3D)   - 
Method in class sim.util. Double3D 
 Returns the manhtattan distance FROM this Double3D TO the specified point
   manhattanDistance(int, int)   - 
Method in class sim.util. Int2D 
 Returns the manhattan distance FROM this Int2D TO the specified point.
   manhattanDistance(MutableInt2D)   - 
Method in class sim.util. Int2D 
 Returns the manhattan distance FROM this Int2D TO the specified point.
   manhattanDistance(Int2D)   - 
Method in class sim.util. Int2D 
 Returns the manhattan distance FROM this Int2D TO the specified point.
   manhattanDistance(Point)   - 
Method in class sim.util. Int2D 
 Returns the manhattan distance FROM this Int2D TO the specified point.
   manhattanDistance(int, int, int)   - 
Method in class sim.util. Int3D 
 Returns the manhattan distance FROM this Int3D TO the specified point.
   manhattanDistance(MutableInt3D)   - 
Method in class sim.util. Int3D 
 Returns the manhattan distance FROM this Int3D TO the specified point.
   manhattanDistance(Int3D)   - 
Method in class sim.util. Int3D 
 Returns the manhattan distance FROM this Int3D TO the specified point.
   manhattanDistance(double, double)   - 
Method in class sim.util. MutableDouble2D 
 Returns the manhtattan distance FROM this MuableDouble2D TO the specified point
   manhattanDistance(Double2D)   - 
Method in class sim.util. MutableDouble2D 
 Returns the manhtattan distance FROM this MuableDouble2D TO the specified point
   manhattanDistance(Int2D)   - 
Method in class sim.util. MutableDouble2D 
 Returns the manhtattan distance FROM this MuableDouble2D TO the specified point
   manhattanDistance(MutableDouble2D)   - 
Method in class sim.util. MutableDouble2D 
 Returns the manhtattan distance FROM this MuableDouble2D TO the specified point
   manhattanDistance(MutableInt2D)   - 
Method in class sim.util. MutableDouble2D 
 Returns the manhtattan distance FROM this MuableDouble2D TO the specified point
   manhattanDistance(Point2D)   - 
Method in class sim.util. MutableDouble2D 
 Returns the manhtattan distance FROM this MuableDouble2D TO the specified point
   manhattanDistance(double, double, double)   - 
Method in class sim.util. MutableDouble3D 
 Returns the manhtattan distance FROM this MutableDouble3D TO the specified point
   manhattanDistance(Double3D)   - 
Method in class sim.util. MutableDouble3D 
 Returns the manhtattan distance FROM this MutableDouble3D TO the specified point
   manhattanDistance(Int3D)   - 
Method in class sim.util. MutableDouble3D 
 Returns the manhtattan distance FROM this MutableDouble3D TO the specified point
   manhattanDistance(MutableDouble3D)   - 
Method in class sim.util. MutableDouble3D 
 Returns the manhtattan distance FROM this MutableDouble3D TO the specified point
   manhattanDistance(MutableInt3D)   - 
Method in class sim.util. MutableDouble3D 
 Returns the manhtattan distance FROM this MutableDouble3D TO the specified point
   manhattanDistance(int, int)   - 
Method in class sim.util. MutableInt2D 
 Returns the manhattan distance FROM this MutableInt2D TO the specified point.
   manhattanDistance(MutableInt2D)   - 
Method in class sim.util. MutableInt2D 
 Returns the manhattan distance FROM this MutableInt2D TO the specified point.
   manhattanDistance(Int2D)   - 
Method in class sim.util. MutableInt2D 
 Returns the manhattan distance FROM this MutableInt2D TO the specified point.
   manhattanDistance(Point)   - 
Method in class sim.util. MutableInt2D 
 Returns the manhattan distance FROM this Int2D TO the specified point.
   manhattanDistance(int, int, int)   - 
Method in class sim.util. MutableInt3D 
 Returns the manhattan distance FROM this Int3D TO the specified point.
   manhattanDistance(MutableInt3D)   - 
Method in class sim.util. MutableInt3D 
 Returns the manhattan distance FROM this Int3D TO the specified point.
   manhattanDistance(Int3D)   - 
Method in class sim.util. MutableInt3D 
 Returns the manhattan distance FROM this Int3D TO the specified point.
   Manipulating2D   - Interface in  sim.display  Manipulating2D is a simple interface for user interface objects which can manipulate
   portrayals in certain ways.   MASON_PREFERENCES   - 
Static variable in class sim.display. Prefs 
  
   max()   - 
Method in class sim.field.grid. DoubleGrid2D 
 Returns the maximum value stored in the grid
   max()   - 
Method in class sim.field.grid. DoubleGrid3D 
 Returns the maximum value stored in the grid
   max()   - 
Method in class sim.field.grid. IntGrid2D 
 Returns the maximum value stored in the grid
   max()   - 
Method in class sim.field.grid. IntGrid3D 
 Returns the maximum value stored in the grid
   maximum(double[])   - 
Static method in class sim.util.gui. MiniHistogram 
 Returns the minimum over the provided vals.
   MAXIMUM_INTEGER   - 
Static variable in class sim.engine. Schedule 
 The last time beyond which the schedule is no longer able to precisely maintain integer values due to loss of precision.
   MAXIMUM_STEPS   - 
Static variable in class sim.display. Console 
 Default maximum number of steps in the step slider
   maximumJump   - 
Variable in class sim.portrayal.simple. TrailedPortrayal2D 
  
   maySetLocation(Object, Object)   - 
Method in interface sim.portrayal. Fixed2D 
 Returns true if the object permits you to change its location in the field.
   mean()   - 
Method in class sim.field.grid. DoubleGrid2D 
 Returns the mean value stored in the grid
   mean()   - 
Method in class sim.field.grid. DoubleGrid3D 
 Returns the mean value stored in the grid
   mean()   - 
Method in class sim.field.grid. IntGrid2D 
 Returns the mean value stored in the grid
   mean()   - 
Method in class sim.field.grid. IntGrid3D 
 Returns the mean value stored in the grid
   MersenneTwisterFast   - Class in  ec.util  MersenneTwister and MersenneTwisterFast   MersenneTwisterFast()   - 
Constructor for class ec.util. MersenneTwisterFast 
 Constructor using the default seed.
   MersenneTwisterFast(long)   - 
Constructor for class ec.util. MersenneTwisterFast 
 Constructor using a given seed.
   MersenneTwisterFast(int[])   - 
Constructor for class ec.util. MersenneTwisterFast 
 Constructor using an array of integers as seed.
   MeshPortrayal   - Class in  sim.portrayal3d.grid.quad  A QuadPortrayal which relates grid locations with  intersections  on a mesh (like positions on
 a Go board, coloring and changing the Z location of the intersections.   MeshPortrayal(ColorMap)   - 
Constructor for class sim.portrayal3d.grid.quad. MeshPortrayal 
  
   MeshPortrayal(ColorMap, double)   - 
Constructor for class sim.portrayal3d.grid.quad. MeshPortrayal 
  
   MethodStep   - Class in  sim.engine  A Steppable which calls an underlying method using Java's reflection system.   MethodStep(Object, String)   - 
Constructor for class sim.engine. MethodStep 
  
   MethodStep(Object, String, boolean)   - 
Constructor for class sim.engine. MethodStep 
  
   min()   - 
Method in class sim.field.grid. DoubleGrid2D 
 Returns the minimum value stored in the grid
   min()   - 
Method in class sim.field.grid. DoubleGrid3D 
 Returns the minimum value stored in the grid
   min()   - 
Method in class sim.field.grid. IntGrid2D 
 Returns the minimum value stored in the grid
   min()   - 
Method in class sim.field.grid. IntGrid3D 
 Returns the minimum value stored in the grid
   MIN_BAG_SIZE   - 
Static variable in class sim.field.grid. DenseGrid2D 
 No bags smaller than this size will be replaced regardless of the setting of  replaceLargeBags 
   MIN_BAG_SIZE   - 
Static variable in class sim.field. SparseField 
 No bags smaller than this size will be replaced regardless of the setting of  replaceLargeBags 
   MinGapDataCuller   - Class in  sim.util.media.chart  This is meant as an on-line algorithm for keeping a constant number of data points
 from an on-going time series.   MinGapDataCuller(int)   - 
Constructor for class sim.util.media.chart. MinGapDataCuller 
  
   MinGapDataCuller(int, int)   - 
Constructor for class sim.util.media.chart. MinGapDataCuller 
  
   MiniHistogram   - Class in  sim.util.gui  A very simple histogram class.   MiniHistogram()   - 
Constructor for class sim.util.gui. MiniHistogram 
  
   MiniHistogram(double[], String[])   - 
Constructor for class sim.util.gui. MiniHistogram 
  
   minimum(double[])   - 
Static method in class sim.util.gui. MiniHistogram 
 Returns the minimum over the provided vals.
   mouseClicked(MouseEvent)   - 
Method in class sim.display3d. SelectionBehavior 
  
   MovablePortrayal2D   - Class in  sim.portrayal.simple  A wrapper for other Portrayal2Ds which makes it possible to drag and move objects with the mouse.   MovablePortrayal2D(SimplePortrayal2D)   - 
Constructor for class sim.portrayal.simple. MovablePortrayal2D 
  
   moveObject(Object, int, int, int, int)   - 
Method in class sim.field.grid. DenseGrid2D 
 If the object is not at [fromX, fromY], then it's simply inserted into [toX, toY], and FALSE is returned.
   moveObject(Object, Int2D, Int2D)   - 
Method in class sim.field.grid. DenseGrid2D 
 If the object is not at FROM, then it's simply inserted into TO, and FALSE is returned.
   moveObjects(int, int, int, int)   - 
Method in class sim.field.grid. DenseGrid2D 
  
   moveObjects(Int2D, Int2D)   - 
Method in class sim.field.grid. DenseGrid2D 
  
   moveSeries(int, boolean)   - 
Method in class sim.util.media.chart. ChartGenerator 
 Override this to move a series relative to other series.
   moveSeries(int, boolean)   - 
Method in class sim.util.media.chart. HistogramGenerator 
  
   moveSeries(int, boolean)   - 
Method in class sim.util.media.chart. ScatterPlotGenerator 
  
   moveSeries(int, boolean)   - 
Method in class sim.util.media.chart. TimeSeriesChartGenerator 
  
   MOVIE_OFF_ICON   - 
Static variable in class sim.display. Display2D 
  
   MOVIE_OFF_ICON_P   - 
Static variable in class sim.display. Display2D 
  
   MOVIE_ON_ICON   - 
Static variable in class sim.display. Display2D 
  
   MOVIE_ON_ICON_P   - 
Static variable in class sim.display. Display2D 
  
   movieButton   - 
Variable in class sim.display. Display2D 
 The button which starts or stops a movie
   movieButton   - 
Variable in class sim.display3d. Display3D 
 The button which starts or stops a movie
   MovieEncoder   - Class in  sim.util.media  Usage of this class depends on the existence of the Java Media Framework (JMF)
 which can be acquired from javasoft.com.   MovieEncoder(float, File, BufferedImage, Format)   - 
Constructor for class sim.util.media. MovieEncoder 
 Creates an object which will write out a move of the specified
        format, and written to the provided file.
   MovieMaker   - Class in  sim.util.gui  A class which gives a GUI front-end to sim.util.media.MovieEncoder.   MovieMaker(Frame)   - 
Constructor for class sim.util.gui. MovieMaker 
  
   multiply(double)   - 
Method in class sim.field.grid. DoubleGrid2D 
 Sets each value in the grid to that value multiplied  byThisMuch 
        Returns the modified grid.
   multiply(IntGrid2D)   - 
Method in class sim.field.grid. DoubleGrid2D 
 Sets the value at each location in the grid to that value multiplied by to the value at the equivalent location in the provided grid.
   multiply(DoubleGrid2D)   - 
Method in class sim.field.grid. DoubleGrid2D 
 Sets the value at each location in the grid to that value multiplied by to the value at the equivalent location in the provided grid.
   multiply(double)   - 
Method in class sim.field.grid. DoubleGrid3D 
 Sets each value in the grid to that value multiplied  byThisMuch 
        Returns the modified grid.
   multiply(IntGrid3D)   - 
Method in class sim.field.grid. DoubleGrid3D 
 Sets the value at each location in the grid to that value multiplied by to the value at the equivalent location in the provided grid.
   multiply(DoubleGrid3D)   - 
Method in class sim.field.grid. DoubleGrid3D 
 Sets the value at each location in the grid to that value multiplied by to the value at the equivalent location in the provided grid.
   multiply(int)   - 
Method in class sim.field.grid. IntGrid2D 
 Sets each value in the grid to that value multiplied  byThisMuch 
        Returns the modified grid.
   multiply(IntGrid2D)   - 
Method in class sim.field.grid. IntGrid2D 
 Sets the value at each location in the grid to that value multiplied by to the value at the equivalent location in the provided grid.
   multiply(int)   - 
Method in class sim.field.grid. IntGrid3D 
 Sets each value in the grid to that value multiplied  byThisMuch 
        Returns the modified grid.
   multiply(IntGrid3D)   - 
Method in class sim.field.grid. IntGrid3D 
 Sets the value at each location in the grid to that value multiplied by to the value at the equivalent location in the provided grid.
   multiply(double)   - 
Method in class sim.util. Double2D 
 Multiplies each element by scalar "val"
   multiply(MutableDouble2D, double)   - 
Method in class sim.util. MutableDouble2D 
 Multiplies other by val, setting me to the result and returning me.
   multiply(Double2D, double)   - 
Method in class sim.util. MutableDouble2D 
 Multiplies other by val, setting me to the result and returning me.
   multiply(MutableDouble3D, double)   - 
Method in class sim.util. MutableDouble3D 
 Multiplies other by val, setting me to the result and returning me.
   multiplyIn(double)   - 
Method in class sim.util. MutableDouble2D 
 Extends my length so that it is multiplied by val, and returns me.
   multiplyIn(double)   - 
Method in class sim.util. MutableDouble3D 
 Extends my length so that it is multiplied by val, and returns me.
   MultiStep   - Class in  sim.engine  MultiStep takes an integer N, a boolean called countdown, and a steppable.   MultiStep(Steppable, int, boolean)   - 
Constructor for class sim.engine. MultiStep 
 If countdown is true, then we call step.step(...) once every N times we're stepped.
   MutableDouble   - Class in  sim.util  MutableDouble simply holds a double value, which can be changed at any time.   MutableDouble()   - 
Constructor for class sim.util. MutableDouble 
  
   MutableDouble(double)   - 
Constructor for class sim.util. MutableDouble 
  
   MutableDouble(MutableDouble)   - 
Constructor for class sim.util. MutableDouble 
  
   MutableDouble2D   - Class in  sim.util  MutableDouble2D is more or less the same class as java.awt.geom.Point2D.Double, except that it is hash-equivalent to Double2D.   MutableDouble2D()   - 
Constructor for class sim.util. MutableDouble2D 
  
   MutableDouble2D(Int2D)   - 
Constructor for class sim.util. MutableDouble2D 
  
   MutableDouble2D(MutableInt2D)   - 
Constructor for class sim.util. MutableDouble2D 
  
   MutableDouble2D(MutableDouble2D)   - 
Constructor for class sim.util. MutableDouble2D 
  
   MutableDouble2D(Double2D)   - 
Constructor for class sim.util. MutableDouble2D 
  
   MutableDouble2D(Point)   - 
Constructor for class sim.util. MutableDouble2D 
  
   MutableDouble2D(Point2D.Double)   - 
Constructor for class sim.util. MutableDouble2D 
  
   MutableDouble2D(Point2D.Float)   - 
Constructor for class sim.util. MutableDouble2D 
  
   MutableDouble2D(Point2D)   - 
Constructor for class sim.util. MutableDouble2D 
 Only included for completeness' sakes, in case a new Point2D subclass is created in the future.
   MutableDouble2D(double, double)   - 
Constructor for class sim.util. MutableDouble2D 
  
   MutableDouble3D   - Class in  sim.util  MutableDouble3D is more or less the same class as javax.vecmath.Point3d, except that it is hash-equivalent to Double3D.   MutableDouble3D()   - 
Constructor for class sim.util. MutableDouble3D 
  
   MutableDouble3D(Int2D)   - 
Constructor for class sim.util. MutableDouble3D 
 Explicitly assumes the z value is set to 0
   MutableDouble3D(Int2D, double)   - 
Constructor for class sim.util. MutableDouble3D 
  
   MutableDouble3D(Int3D)   - 
Constructor for class sim.util. MutableDouble3D 
  
   MutableDouble3D(MutableInt2D)   - 
Constructor for class sim.util. MutableDouble3D 
 Explicitly assumes the z value is set to 0
   MutableDouble3D(MutableInt2D, double)   - 
Constructor for class sim.util. MutableDouble3D 
  
   MutableDouble3D(MutableInt3D)   - 
Constructor for class sim.util. MutableDouble3D 
  
   MutableDouble3D(Double2D)   - 
Constructor for class sim.util. MutableDouble3D 
 Explicitly assumes the z value is set to 0
   MutableDouble3D(Double2D, double)   - 
Constructor for class sim.util. MutableDouble3D 
  
   MutableDouble3D(Double3D)   - 
Constructor for class sim.util. MutableDouble3D 
  
   MutableDouble3D(MutableDouble2D)   - 
Constructor for class sim.util. MutableDouble3D 
 Explicitly assumes the z value is set to 0
   MutableDouble3D(MutableDouble2D, double)   - 
Constructor for class sim.util. MutableDouble3D 
  
   MutableDouble3D(MutableDouble3D)   - 
Constructor for class sim.util. MutableDouble3D 
  
   MutableDouble3D(double, double, double)   - 
Constructor for class sim.util. MutableDouble3D 
  
   MutableInt2D   - Class in  sim.util  MutableInt2D is largely a class identical to java.awt.Point, except that it is hash-equivalent to Int2D.   MutableInt2D()   - 
Constructor for class sim.util. MutableInt2D 
  
   MutableInt2D(Point)   - 
Constructor for class sim.util. MutableInt2D 
  
   MutableInt2D(Int2D)   - 
Constructor for class sim.util. MutableInt2D 
  
   MutableInt2D(int, int)   - 
Constructor for class sim.util. MutableInt2D 
  
   MutableInt3D   - Class in  sim.util  MutableInt3D stores three values (x, y, and z) and is hash-equivalent to Int3D; except MutableInt3D's
   values can be modified and Int3D's values cannot.   MutableInt3D()   - 
Constructor for class sim.util. MutableInt3D 
  
   MutableInt3D(int, int, int)   - 
Constructor for class sim.util. MutableInt3D 
  
   MutableInt3D(Int2D)   - 
Constructor for class sim.util. MutableInt3D 
 Explicitly assumes the z value is set to 0
   MutableInt3D(Int2D, int)   - 
Constructor for class sim.util. MutableInt3D 
  
   MutableInt3D(Int3D)   - 
Constructor for class sim.util. MutableInt3D 
  
   MutableInt3D(MutableInt2D)   - 
Constructor for class sim.util. MutableInt3D 
  
   MutableInt3D(MutableInt2D, int)   - 
Constructor for class sim.util. MutableInt3D 
  
 
 
    
 N  
 
   NAME_INDICATOR   - 
Static variable in class sim.display. Console 
 Used in the 'simulation.classes' file to specify a special name for a simulation.
   nameThread()   - 
Method in class sim.engine. SimState 
 Names the current thread an appropriate name given the SimState
   negate()   - 
Method in class sim.util. Double2D 
 Returns the negation of this Double2D.
   negate()   - 
Method in class sim.util. MutableDouble2D 
 Negates the MutableDouble2D's values and returns it.
   negate()   - 
Method in class sim.util. MutableDouble3D 
 Negates the MutableDouble3D's values
   Network   - Class in  sim.field.network  The Network is a field which stores binary graph and multigraph structures of all kinds, using hash tables to allow
    reasonably rapid dynamic modification.   Network(boolean)   - 
Constructor for class sim.field.network. Network 
 Constructs a directed or undirected graph.
   Network()   - 
Constructor for class sim.field.network. Network 
 Constructs a directed graph
   Network(Network)   - 
Constructor for class sim.field.network. Network 
 Constructs copy of an existing graph.
   Network.IndexOutIn   - Class in  sim.field.network  The structure stored in the indexOutInHash hash table.   Network.IndexOutIn(int, Bag, Bag)   - 
Constructor for class sim.field.network. Network.IndexOutIn 
  
   NetworkPortrayal2D   - Class in  sim.portrayal.network  Portrays network fields.   NetworkPortrayal2D()   - 
Constructor for class sim.portrayal.network. NetworkPortrayal2D 
  
   NEVER_SCALE   - 
Static variable in class sim.portrayal.network. SimpleEdgePortrayal2D 
  
   NEVER_SCALE   - 
Static variable in class sim.portrayal.simple. LabelledPortrayal2D 
  
   newInstance(long, String[])   - 
Method in interface sim.engine. MakesSimState 
 Creates a SimState subclass with the given random number seed
        and command-line arguments passed into main(...).
   newValue(int, int, double)   - 
Method in class sim.portrayal.grid. ValueGridPortrayal2D 
 This method is called by the default inspector to filter new values set by the user.
   newValue(int, int, double)   - 
Method in class sim.portrayal3d.grid. ValueGrid2DPortrayal3D 
 This method is called by the default inspector to filter new values set by the user.
   newValue(int, int, int, double)   - 
Method in class sim.portrayal3d.grid. ValueGridPortrayal3D 
 This method is called by the default inspector to filter new values set by the user.
   newValue(double)   - 
Method in class sim.util.gui. NumberTextField 
 Override this to be informed when a new value has been set.
   newValue(String)   - 
Method in class sim.util.gui. PropertyField 
 Override this to be informed when a new value has been set.
   nextBoolean()   - 
Method in class ec.util. MersenneTwisterFast 
  
   nextBoolean(float)   - 
Method in class ec.util. MersenneTwisterFast 
 This generates a coin flip with a probability  probability 
        of returning true, else returning false.
   nextBoolean(double)   - 
Method in class ec.util. MersenneTwisterFast 
 This generates a coin flip with a probability  probability 
        of returning true, else returning false.
   nextByte()   - 
Method in class ec.util. MersenneTwisterFast 
  
   nextBytes(byte[])   - 
Method in class ec.util. MersenneTwisterFast 
  
   nextChar()   - 
Method in class ec.util. MersenneTwisterFast 
  
   nextDouble()   - 
Method in class ec.util. MersenneTwisterFast 
 Returns a random double in the half-open range from [0.0,1.0).
   nextFloat()   - 
Method in class ec.util. MersenneTwisterFast 
 Returns a random float in the half-open range from [0.0f,1.0f).
   nextGaussian()   - 
Method in class ec.util. MersenneTwisterFast 
  
   nextInt()   - 
Method in class ec.util. MersenneTwisterFast 
  
   nextInt(int)   - 
Method in class ec.util. MersenneTwisterFast 
 Returns an integer drawn uniformly from 0 to n-1.
   nextLong()   - 
Method in class ec.util. MersenneTwisterFast 
  
   nextLong(long)   - 
Method in class ec.util. MersenneTwisterFast 
 Returns a long drawn uniformly from 0 to n-1.
   nextShort()   - 
Method in class ec.util. MersenneTwisterFast 
  
   nodeExists(Object)   - 
Method in class sim.field.network. Network 
  
   normalize()   - 
Method in class sim.util. Double2D 
  
   normalize()   - 
Method in class sim.util. MutableDouble2D 
  
   normalize()   - 
Method in class sim.util. MutableDouble3D 
 Normalizes me (sets my length to 1.0), returning me.
   NumberTextField   - Class in  sim.util.gui  A simple class that lets you specify a label and validate a numerical value.   NumberTextField(double)   - 
Constructor for class sim.util.gui. NumberTextField 
 Creates a NumberTextField which does not display the belly button or arrows.
   NumberTextField(String, double)   - 
Constructor for class sim.util.gui. NumberTextField 
 Creates a NumberTextField which does not display the belly button or arrows.
   NumberTextField(double, boolean)   - 
Constructor for class sim.util.gui. NumberTextField 
 Creates a NumberTextField which (if  doubleEachTime )
        doubles or halves the current value, or (if not  doubleEachTime ) 
        increases or decreases by 1 each time.
   NumberTextField(double, double, double)   - 
Constructor for class sim.util.gui. NumberTextField 
 Creates a NumberTextField according to the provided parameters.
   NumberTextField(String, double, boolean)   - 
Constructor for class sim.util.gui. NumberTextField 
 Creates a NumberTextField with a provided label.
   NumberTextField(String, double, double, double)   - 
Constructor for class sim.util.gui. NumberTextField 
 Creates a NumberTextField with a provided label.
   numObjectsAtLocation(Double2D)   - 
Method in class sim.field.continuous. Continuous2D 
 Returns the number of the objects at a given location, 
        or 0 if there are no such objects or if location is null.
   numObjectsAtLocation(Double3D)   - 
Method in class sim.field.continuous. Continuous3D 
 Returns the number of the objects at a given location, 
        or 0 if there are no such objects or if location is null.
   numObjectsAtLocation(int, int)   - 
Method in class sim.field.grid. DenseGrid2D 
  
   numObjectsAtLocation(Int2D)   - 
Method in class sim.field.grid. DenseGrid2D 
  
   numObjectsAtLocation(int, int)   - 
Method in class sim.field.grid. SparseGrid2D 
 Returns the number of objects stored in the grid at the given location.
   numObjectsAtLocation(int, int, int)   - 
Method in class sim.field.grid. SparseGrid3D 
 Returns the number of objects stored in the grid at the given location.
   numObjectsAtLocation(Object)   - 
Method in class sim.field. SparseField 
 Returns the number of objects at a given location.
   numObjectsAtLocationOfObject(Object)   - 
Method in class sim.field.continuous. Continuous2D 
 Returns the number of objects at the exact same location as a given object, including the object itself, 
        or 0 if the object is not in the Field.
   numObjectsAtLocationOfObject(Object)   - 
Method in class sim.field.continuous. Continuous3D 
 Returns the number of objects at the exact same location as a given object, including the object itself, 
        or 0 if the object is not in the Field.
   numObjectsAtLocationOfObject(Object)   - 
Method in class sim.field. SparseField 
 Returns the number of objects at the same location as a given object, including the object itself, or 0 if the object
        is not in the SparseField.
   numObjs   - 
Variable in class sim.util. Bag 
  
   numObjs   - 
Variable in class sim.util. DoubleBag 
  
   numObjs   - 
Variable in class sim.util. IntBag 
  
   numProperties()   - 
Method in class sim.util. CollectionProperties 
  
   numProperties()   - 
Method in class sim.util. Properties 
 Returns the number of properties discovered in the object.
   numProperties()   - 
Method in class sim.util. SimpleProperties 
 Returns the number of properties discovered
   numShapes()   - 
Method in class sim.portrayal3d.simple. BranchGroupPortrayal3D 
 Unused: returns 0 always.
   numShapes()   - 
Method in class sim.portrayal3d.simple. ConePortrayal3D 
  
   numShapes()   - 
Method in class sim.portrayal3d.simple. CylinderPortrayal3D 
  
   numShapes()   - 
Method in class sim.portrayal3d.simple. PrimitivePortrayal3D 
 Returns the number of shapes handled by this primitive or Shape3D.
   numShapes()   - 
Method in class sim.portrayal3d.simple. Shape3DPortrayal3D 
  
   numShapes()   - 
Method in class sim.portrayal3d.simple. SpherePortrayal3D 
  
 
 
    
 O  
 
   object   - 
Variable in class sim.portrayal. LocationWrapper 
 The ORIGINAL object
   object   - 
Variable in class sim.util. Properties 
  
   ObjectGrid2D   - Class in  sim.field.grid  A wrapper for 2D arrays of Objects.   ObjectGrid2D(int, int)   - 
Constructor for class sim.field.grid. ObjectGrid2D 
  
   ObjectGrid2D(int, int, Object)   - 
Constructor for class sim.field.grid. ObjectGrid2D 
  
   ObjectGrid2D(ObjectGrid2D)   - 
Constructor for class sim.field.grid. ObjectGrid2D 
  
   ObjectGrid3D   - Class in  sim.field.grid  A wrapper for 3D arrays of Objects.   ObjectGrid3D(int, int, int)   - 
Constructor for class sim.field.grid. ObjectGrid3D 
  
   ObjectGrid3D(int, int, int, Object)   - 
Constructor for class sim.field.grid. ObjectGrid3D 
  
   ObjectGrid3D(ObjectGrid3D)   - 
Constructor for class sim.field.grid. ObjectGrid3D 
  
   ObjectGridPortrayal2D   - Class in  sim.portrayal.grid  A portrayal for grids containing objects, such as maybe agents or agent bodies.   ObjectGridPortrayal2D()   - 
Constructor for class sim.portrayal.grid. ObjectGridPortrayal2D 
  
   ObjectGridPortrayal2D.Message   - Class in  sim.portrayal.grid      ObjectGridPortrayal2D.Message(String)   - 
Constructor for class sim.portrayal.grid. ObjectGridPortrayal2D.Message 
  
   ObjectGridPortrayal3D   - Class in  sim.portrayal3d.grid  Portrays ObjectGrid2D and ObjectGrid3D in 3D space.   ObjectGridPortrayal3D()   - 
Constructor for class sim.portrayal3d.grid. ObjectGridPortrayal3D 
  
   objectHash   - 
Variable in class sim.field. SparseField 
 Bags of objects hashed by location.
   objectsHitBy(Rectangle2D.Double)   - 
Method in class sim.display. Display2D 
 Returns LocationWrappers for all the objects which fall within the coordinate rectangle specified by rect.
   objectsHitBy(Point2D)   - 
Method in class sim.display. Display2D 
 Returns LocationWrappers for all the objects which overlap with the point specified by 'point'.
   objectToDraw(Bag, Bag)   - 
Method in interface sim.portrayal.grid. DrawPolicy 
 Specifies objects which should be drawn at a given location, and which objects should not.
   objs   - 
Variable in class sim.util. Bag 
  
   objs   - 
Variable in class sim.util. DoubleBag 
  
   objs   - 
Variable in class sim.util. IntBag 
  
   offset   - 
Variable in class sim.portrayal.simple. OrientedPortrayal2D 
 The post-scaling length offset
   offset   - 
Variable in class sim.portrayal.simple. OvalPortrayal2D 
  
   offsetx   - 
Variable in class sim.portrayal.simple. LabelledPortrayal2D 
 The post-scaling offset from the object's origin.
   offsety   - 
Variable in class sim.portrayal.simple. LabelledPortrayal2D 
 The post-scaling offset from the object's origin.
   ONLY_INDICATOR   - 
Static variable in class sim.display. Console 
 Used in the 'simulation.classes' file to indicate that nonstandard classes may not be accessed.
   onlyLabelWhenSelected   - 
Variable in class sim.portrayal.simple. LabelledPortrayal2D 
  
   optionButton   - 
Variable in class sim.display. Display2D 
 The button which pops up the option pane
   optionButton   - 
Variable in class sim.display3d. Display3D 
 The button which pops up the option pane
   optionPane   - 
Variable in class sim.display. Display2D 
 Our option pane
   optionPane   - 
Variable in class sim.display3d. Display3D 
  
   OPTIONS_ICON   - 
Static variable in class sim.display. Display2D 
  
   OPTIONS_ICON_P   - 
Static variable in class sim.display. Display2D 
  
   Orientable2D   - Interface in  sim.portrayal  An Orientable2D object can have its orientation changed in radians.   orientation2D()   - 
Method in interface sim.portrayal. Oriented2D 
  
   Oriented2D   - Interface in  sim.portrayal  An Oriented2D object provides an orientation in radians.   OrientedPortrayal2D   - Class in  sim.portrayal.simple  A wrapper for other Portrayal2Ds which provides some kind of pointing object (typically a line)
   along the object's specified orientation angle.   OrientedPortrayal2D(SimplePortrayal2D, int, double, Paint, int)   - 
Constructor for class sim.portrayal.simple. OrientedPortrayal2D 
  
   OrientedPortrayal2D(SimplePortrayal2D, int, double, Paint)   - 
Constructor for class sim.portrayal.simple. OrientedPortrayal2D 
 If child is null, then the underlying model object 
        is presumed to be a Portrayal2D and will be used.
   OrientedPortrayal2D(SimplePortrayal2D)   - 
Constructor for class sim.portrayal.simple. OrientedPortrayal2D 
 Draw a line of length scale = 0.5, offset = 0, in red.
   OrientedPortrayal2D(SimplePortrayal2D, int, double)   - 
Constructor for class sim.portrayal.simple. OrientedPortrayal2D 
 Draw a line of the given length in red.
   OrientedPortrayal2D(SimplePortrayal2D, Paint)   - 
Constructor for class sim.portrayal.simple. OrientedPortrayal2D 
 Draw a line of length scale = 0.5, offset = 0.
   out   - 
Variable in class sim.field.network. Network.IndexOutIn 
 Bag containing outgoing edges of (leaving) the node
   OvalPortrayal2D   - Class in  sim.portrayal.simple  A simple portrayal for 2D visualization of ovals.   OvalPortrayal2D()   - 
Constructor for class sim.portrayal.simple. OvalPortrayal2D 
  
   OvalPortrayal2D(Paint)   - 
Constructor for class sim.portrayal.simple. OvalPortrayal2D 
  
   OvalPortrayal2D(double)   - 
Constructor for class sim.portrayal.simple. OvalPortrayal2D 
  
   OvalPortrayal2D(Paint, double)   - 
Constructor for class sim.portrayal.simple. OvalPortrayal2D 
  
   OvalPortrayal2D(Paint, boolean)   - 
Constructor for class sim.portrayal.simple. OvalPortrayal2D 
  
   OvalPortrayal2D(double, boolean)   - 
Constructor for class sim.portrayal.simple. OvalPortrayal2D 
  
   OvalPortrayal2D(Paint, double, boolean)   - 
Constructor for class sim.portrayal.simple. OvalPortrayal2D 
  
   owner()   - 
Method in class sim.field.network. Edge 
 Returns the "owner" field.
 
 
    
 P  
 
   paint(Graphics, boolean, boolean)   - 
Method in class sim.display. Display2D.InnerDisplay2D 
 Paints an image to the screen either buffered or unbuffered.
   paint   - 
Variable in class sim.portrayal.simple. LabelledPortrayal2D 
 The Paint or Color of the text
   paint   - 
Variable in class sim.portrayal.simple. OrientedPortrayal2D 
 The Paint or Color of the line
   paint   - 
Variable in class sim.portrayal.simple. OvalPortrayal2D 
  
   paint   - 
Variable in class sim.portrayal.simple. RectanglePortrayal2D 
  
   paint   - 
Variable in class sim.portrayal.simple. ShapePortrayal2D 
  
   paintComponent(Graphics)   - 
Method in class sim.display. Display2D.InnerDisplay2D 
 Swing's equivalent of paint(Graphics g).
   paintComponent(Graphics, boolean)   - 
Method in class sim.display. Display2D.InnerDisplay2D 
  Deprecated.   use paintComponent() or paint(...) 
   paintComponent(Graphics)   - 
Method in class sim.display3d. Display3D 
 Updates the scene graph
   paintComponent(Graphics)   - 
Method in class sim.util.gui. ColorWell 
  
   paintComponent(Graphics)   - 
Method in class sim.util.gui. MiniHistogram 
  
   paintToMovie(Graphics)   - 
Method in class sim.display. Display2D.InnerDisplay2D 
 Paints a movie, by drawing to a buffer, then
            encoding the buffer to disk, then optionally 
            writing the buffer to the provided Graphics2D.
   ParallelSequence   - Class in  sim.engine  Spawns all the sequence elements in parallel on separate threads.   ParallelSequence(Steppable[], int)   - 
Constructor for class sim.engine. ParallelSequence 
 Creates a ParallelSequence with the specified number of threads, or if threads==ParallelSequence.CPUS, then the number of threads is determined
        at runtime based on the number of CPUs or cores on the system.
   ParallelSequence(Steppable[])   - 
Constructor for class sim.engine. ParallelSequence 
 Creates a ParallelSequence with one thread per steppable.
   pause()   - 
Method in class sim.engine. AsynchronousSteppable 
 Requests that the AsynchronousSteppable shut down its thread (temporarily) and blocks until this occurs.
   PDFEncoder   - Class in  sim.util.media      PDFEncoder()   - 
Constructor for class sim.util.media. PDFEncoder 
  
   performSelection(LocationWrapper)   - 
Method in class sim.display. Display2D 
 Selects the following object, deselecting other objects if so asked.
   performSelection(Bag)   - 
Method in class sim.display. Display2D 
  
   performSelection(Rectangle2D.Double)   - 
Method in class sim.display. Display2D 
  
   performSelection(LocationWrapper)   - 
Method in interface sim.display. Manipulating2D 
 Selects the following object, deselecting other selected objects.
   performSelection(LocationWrapper)   - 
Method in class sim.display3d. Display3D 
  
   performSelection(Bag)   - 
Method in class sim.display3d. Display3D 
  
   perpDot(Double2D)   - 
Method in class sim.util. Double2D 
 2D version of the cross product.
   perpDot(MutableDouble2D)   - 
Method in class sim.util. MutableDouble2D 
 2D version of the cross product: returns the dot product of me rotated 90 degrees dotted
        against the other vector.
   pngEncode()   - 
Method in class sim.util.media. PNGEncoder 
 Creates an array of bytes that is the PNG equivalent of the current image, specifying whether to encode alpha or not.
   PNGEncoder   - Class in  sim.util.media  PNGEncoder takes a Java Image object and creates a byte string which can be saved as a PNG file.   PNGEncoder()   - 
Constructor for class sim.util.media. PNGEncoder 
 Class constructor
   PNGEncoder(Image)   - 
Constructor for class sim.util.media. PNGEncoder 
 Class constructor specifying Image to encode, with no alpha channel encoding.
   PNGEncoder(Image, boolean)   - 
Constructor for class sim.util.media. PNGEncoder 
 Class constructor specifying Image to encode, and whether to encode alpha.
   PNGEncoder(Image, boolean, int)   - 
Constructor for class sim.util.media. PNGEncoder 
 Class constructor specifying Image to encode, whether to encode alpha, and filter to use.
   PNGEncoder(Image, boolean, int, int)   - 
Constructor for class sim.util.media. PNGEncoder 
 Class constructor specifying Image source to encode, whether to encode alpha, filter to use, and compression level.
   policy   - 
Variable in class sim.portrayal.grid. SparseGridPortrayal2D 
  
   polygonAttributes()   - 
Method in class sim.portrayal3d. FieldPortrayal3D 
  
   polygonAttributes()   - 
Method in class sim.portrayal3d.grid. ValueGrid2DPortrayal3D 
  
   polygonAttributes()   - 
Method in class sim.portrayal3d.grid. ValueGridPortrayal3D 
  
   polygonAttributes()   - 
Method in interface sim.portrayal3d. Portrayal3D 
 Provides a PolygonAttributes which can be modified to change
 the underlying model's attributes (culling, vertex versus point versus fill).
   polygonAttributes()   - 
Method in class sim.portrayal3d.simple. CircledPortrayal3D 
  
   polygonAttributes()   - 
Method in class sim.portrayal3d.simple. LabelledPortrayal3D 
  
   polygonAttributes()   - 
Method in class sim.portrayal3d.simple. SharedPortrayal3D 
  
   polygonAttributes()   - 
Method in class sim.portrayal3d.simple. TransformedPortrayal3D 
  
   polygonAttributes()   - 
Method in class sim.portrayal3d.simple. ValuePortrayal3D 
  
   polygonAttributes()   - 
Method in class sim.portrayal3d. SimplePortrayal3D 
  
   pop()   - 
Method in class sim.util. Bag 
 Returns null if the Bag is empty, else removes and returns the topmost object.
   pop()   - 
Method in class sim.util. DoubleBag 
 Returns 0 if the DoubleBag is empty, else removes and returns the topmost double.
   pop()   - 
Method in class sim.util. IntBag 
 Returns 0 if the IntBag is empty, else removes and returns the topmost int.
   popup   - 
Variable in class sim.display. Display2D 
 The popup layers menu
   popup   - 
Variable in class sim.display3d. Display3D 
 The popup layers menu
   Portrayal   - Interface in  sim.portrayal  A common interface for portrayal classes.   Portrayal2D   - Interface in  sim.portrayal  The basic 2D portrayal interface.   Portrayal3D   - Interface in  sim.portrayal3d  The top-level definition of Portrayals which portray underlying models using, er,
 Java3D models.   portrayalForAll   - 
Variable in class sim.portrayal. FieldPortrayal 
  
   portrayalForNonNull   - 
Variable in class sim.portrayal. FieldPortrayal 
  
   portrayalForNull   - 
Variable in class sim.portrayal. FieldPortrayal 
  
   portrayalForRemainder   - 
Variable in class sim.portrayal. FieldPortrayal 
  
   portrayals   - 
Variable in class sim.portrayal. FieldPortrayal 
  
   possiblyCull()   - 
Method in class sim.util.media.chart. TimeSeriesAttributes 
  
   postCheckpoint()   - 
Method in class sim.engine. SimState 
 Called just after the SimState was checkpointed (serialized out to a file to be
        unserialized and fired up at a future time).
   postRender()   - 
Method in class sim.display3d. CapturingCanvas3D 
  
   postSwap()   - 
Method in class sim.display3d. CapturingCanvas3D 
  
   preCheckpoint()   - 
Method in class sim.engine. SimState 
 Called just before the SimState is being checkpointed (serialized out to a file to be
        unserialized and fired up at a future time).
   precise   - 
Variable in class sim.display. Display2D 
  
   precise   - 
Variable in class sim.portrayal. DrawInfo2D 
  
   Prefs   - Class in  sim.display  A simple cover class for Java's preferences system.   Prefs()   - 
Constructor for class sim.display. Prefs 
  
   pressPause()   - 
Method in class sim.display. Console 
 Called when the user presses the pause button.
   pressPause()   - 
Method in class sim.display. SimpleController 
 Called when the user presses the pause button.
   pressPlay()   - 
Method in class sim.display. Console 
 Called when the user presses the play button.
   pressPlay()   - 
Method in class sim.display. SimpleController 
 Called when the user presses the play button.
   pressStop()   - 
Method in class sim.display. Console 
 Called when the user presses the stop button.
   pressStop()   - 
Method in class sim.display. SimpleController 
 Called when the user presses the stop button.
   PrimitivePortrayal3D   - Class in  sim.portrayal3d.simple  An abstract superclass for portrayals involving Shape3D or various Primitive (sphere, cone, etc.) objects.   PrimitivePortrayal3D()   - 
Constructor for class sim.portrayal3d.simple. PrimitivePortrayal3D 
  
   printStackTrace(PrintStream)   - 
Method in exception sim.util. CausedRuntimeException 
  Deprecated.   
   printStackTrace(PrintWriter)   - 
Method in exception sim.util. CausedRuntimeException 
  Deprecated.   
   printStackTrace()   - 
Method in exception sim.util. CausedRuntimeException 
  Deprecated.   
   processStimulus(Enumeration)   - 
Method in class sim.display3d. SelectionBehavior 
  
   processStimulus(Enumeration)   - 
Method in class sim.display3d. ToolTipBehavior 
 Disregard all stimuli other than ...
   Propertied   - Interface in  sim.util  A Propertied object is one which provides its own Properties rather than
   letting SimpleProperties scan the object statically.   properties()   - 
Method in interface sim.util. Propertied 
 Returns my own Properties.
   Properties   - Class in  sim.util  The abstract superclass of Property inspectors.   Properties()   - 
Constructor for class sim.util. Properties 
  
   propertiesProxy()   - 
Method in interface sim.util. Proxiable 
 Returns the proxy object to query for Properties instead of me.
   PropertyField   - Class in  sim.util.gui  A simple class designed to allow the user to modify a property in the form of a string, number, boolean value, or option.   PropertyField()   - 
Constructor for class sim.util.gui. PropertyField 
 Constructs a PropertyField as just a writeable, empty text field.
   PropertyField(String)   - 
Constructor for class sim.util.gui. PropertyField 
 Constructs a PropertyField as a writeable text field with the provided initial value.
   PropertyField(String, boolean)   - 
Constructor for class sim.util.gui. PropertyField 
 Constructs a PropertyField as a text field with the provided initial value, either writeable or not.
   PropertyField(String, String)   - 
Constructor for class sim.util.gui. PropertyField 
 Constructs a labelled PropertyField as a writeable text field with the provided initial value.
   PropertyField(String, String, boolean)   - 
Constructor for class sim.util.gui. PropertyField 
 Constructs a labelled PropertyField as a text field with the provided initial value, either writeable or not.
   PropertyField(String, String, boolean, Object, int)   - 
Constructor for class sim.util.gui. PropertyField 
 Constructs a PropertyField with an optional label, an initial value, a "writeable" flag, an optional domain
        (for the slider and list options), and a display form (checkboxes, view buttons, text fields, sliders, or lists).
   Proxiable   - Interface in  sim.util  A proxiable object is one which provides SimpleProperties with a proxy to
   stand in for it; that is, to have the proxy's properties inspected instead
   of the object itself.   PS_PAUSED   - 
Static variable in class sim.display. Console 
 The play thread is presently paused.
   PS_PAUSED   - 
Static variable in class sim.display. SimpleController 
 The play thread is presently paused.
   PS_PLAYING   - 
Static variable in class sim.display. Console 
 The play thread is presently playing.
   PS_PLAYING   - 
Static variable in class sim.display. SimpleController 
 The play thread is presently playing.
   PS_STOPPED   - 
Static variable in class sim.display. Console 
 The play thread is presently stopped.
   PS_STOPPED   - 
Static variable in class sim.display. SimpleController 
 The play thread is presently stopped.
   push(Object)   - 
Method in class sim.util. Bag 
 Synonym for add(obj) -- stylistically, you should add instead unless you
        want to think of the Bag as a stack.
   push(double)   - 
Method in class sim.util. DoubleBag 
 Synonym for add(obj) -- try to use add instead unless you
        want to think of the DoubleBag as a stack.
   push(int)   - 
Method in class sim.util. IntBag 
 Synonym for add(obj) -- try to use add instead unless you
        want to think of the IntBag as a stack.
 
 
    
 Q  
 
   QuadPortrayal   - Class in  sim.portrayal3d.grid.quad  A QuadPortrayal is the abstract superclass of objects which describe how rectangles in a
 in a ValueGrid2DPortrayal3D are associated with the underlying ValueGrid2D.   QuadPortrayal(ColorMap, double)   - 
Constructor for class sim.portrayal3d.grid.quad. QuadPortrayal 
  
   QuadPortrayal.DoubleFilter   - Class in  sim.portrayal3d.grid.quad      QuadPortrayal.DoubleFilter(LocationWrapper)   - 
Constructor for class sim.portrayal3d.grid.quad. QuadPortrayal.DoubleFilter 
  
   QuadPortrayal.Filter   - Class in  sim.portrayal3d.grid.quad      QuadPortrayal.Filter(LocationWrapper)   - 
Constructor for class sim.portrayal3d.grid.quad. QuadPortrayal.Filter 
  
   QuadPortrayal.IntFilter   - Class in  sim.portrayal3d.grid.quad      QuadPortrayal.IntFilter(LocationWrapper)   - 
Constructor for class sim.portrayal3d.grid.quad. QuadPortrayal.IntFilter 
  
   QuadPortrayal.ObjectFilter   - Class in  sim.portrayal3d.grid.quad      QuadPortrayal.ObjectFilter(LocationWrapper)   - 
Constructor for class sim.portrayal3d.grid.quad. QuadPortrayal.ObjectFilter 
  
   quit()   - 
Method in class sim.display. Display2D 
 Quits the Display2D.
   quit()   - 
Method in class sim.display. GUIState 
 Called by the Console when the user is quitting the SimState.
   quit()   - 
Method in class sim.display3d. Display3D 
 Quits the Display3D.
   quit()   - 
Method in class sim.util.media.chart. ChartGenerator 
 Prepares the chart to be garbage collected.
 
 
    
 R  
 
   random   - 
Variable in class sim.engine. SimState 
 The SimState's random number generator
   RandomSequence   - Class in  sim.engine  RandomSequence is a Sequence which executes its Steppable objects in random order each time.   RandomSequence(Steppable[])   - 
Constructor for class sim.engine. RandomSequence 
 Does not synchronize before using the random number generator
   RandomSequence(Steppable[], boolean)   - 
Constructor for class sim.engine. RandomSequence 
 Synchronizes on the random number generator only if shouldSynchronize is true
   RateAdjuster   - Class in  sim.display  A simple class which maintains a frame rate fixed to no more than a given number of ticks per second.   RateAdjuster(double)   - 
Constructor for class sim.display. RateAdjuster 
  
   readFromCheckpoint(File)   - 
Static method in class sim.engine. SimState 
 Creates a SimState from checkpoint.
   readFromCheckpoint(InputStream)   - 
Static method in class sim.engine. SimState 
 Creates and returns a new SimState object read in from the provided stream.
   readNewStateFromCheckpoint(File)   - 
Method in class sim.display. GUIState 
 Loads a new SimState from the provided file.
   readState(DataInputStream)   - 
Method in class ec.util. MersenneTwisterFast 
 Reads the entire state of the MersenneTwister RNG from the stream
   rebuildGraphicsDefinitions()   - 
Method in class sim.util.media.chart. HistogramSeriesAttributes 
  
   rebuildGraphicsDefinitions()   - 
Method in class sim.util.media.chart. ScatterPlotSeriesAttributes 
  
   rebuildGraphicsDefinitions()   - 
Method in class sim.util.media.chart. SeriesAttributes 
 Updates features of the series to reflect the current widget settings as specified by the user.
   rebuildGraphicsDefinitions()   - 
Method in class sim.util.media.chart. TimeSeriesAttributes 
  
   RectanglePortrayal2D   - Class in  sim.portrayal.simple  A simple portrayal for 2D visualization of rectangles.   RectanglePortrayal2D()   - 
Constructor for class sim.portrayal.simple. RectanglePortrayal2D 
  
   RectanglePortrayal2D(Paint)   - 
Constructor for class sim.portrayal.simple. RectanglePortrayal2D 
  
   RectanglePortrayal2D(double)   - 
Constructor for class sim.portrayal.simple. RectanglePortrayal2D 
  
   RectanglePortrayal2D(Paint, double)   - 
Constructor for class sim.portrayal.simple. RectanglePortrayal2D 
  
   RectanglePortrayal2D(Paint, boolean)   - 
Constructor for class sim.portrayal.simple. RectanglePortrayal2D 
  
   RectanglePortrayal2D(double, boolean)   - 
Constructor for class sim.portrayal.simple. RectanglePortrayal2D 
  
   RectanglePortrayal2D(Paint, double, boolean)   - 
Constructor for class sim.portrayal.simple. RectanglePortrayal2D 
  
   REDRAW_OPTIONS   - 
Static variable in class sim.display. Display2D 
  
   refresh()   - 
Method in class sim.display. Console 
  
   refresh()   - 
Method in interface sim.display. Controller 
 Lazily updates and redraws all the displays and inspectors.
   refresh()   - 
Method in class sim.display. SimpleController 
  
   REFRESH_ICON   - 
Static variable in class sim.display. Display2D 
  
   REFRESH_ICON_P   - 
Static variable in class sim.display. Display2D 
  
   refreshbutton   - 
Variable in class sim.display. Display2D 
 The button which pops up the refresh menu
   refreshbutton   - 
Variable in class sim.display3d. Display3D 
 The button which pops up the refresh menu
   refreshPopup   - 
Variable in class sim.display. Display2D 
 The refresh menu
   refreshPopup   - 
Variable in class sim.display3d. Display3D 
 The field for scaling values
   registerFrame(JFrame)   - 
Method in class sim.display. Console 
 Simulations can call this to add a frame to be listed in the "Display list" of the console
   registerFrame(JFrame)   - 
Method in interface sim.display. Controller 
 Simulations can call this to add a frame to be listed in the "Display list" of the Controller.
   registerFrame(JFrame)   - 
Method in class sim.display. SimpleController 
 Simulations can call this to add a frame to be listed in the "Display list" of the console
   registerInspector(Inspector, Stoppable)   - 
Method in class sim.display. Console 
 Registers an inspector to be Stopped if necessary in the future.
   registerInspector(Inspector, Stoppable)   - 
Method in interface sim.display. Controller 
 Registers an inspector to be refreshed as appropriate and stopped when the model is restarted.
   registerInspector(Inspector, Stoppable)   - 
Method in class sim.display. SimpleController 
 Registers an inspector to be Stopped if necessary in the future.
   remove(Object)   - 
Method in class sim.field.continuous. Continuous2D 
  
   remove(Object)   - 
Method in class sim.field.continuous. Continuous3D 
  
   remove(Object)   - 
Method in class sim.field. SparseField 
 Removes an object if it exists.
   remove(Object)   - 
Method in class sim.util. Bag 
 Removes the object, moving the topmost object into its position.
   remove(int)   - 
Method in class sim.util. Bag 
 Removes the object at the given index, moving the topmost object into its position.
   remove(int)   - 
Method in class sim.util. DoubleBag 
 Removes the double at the given index, moving the topmost double into its position.
   remove(int)   - 
Method in class sim.util. IntBag 
 Removes the int at the given index, moving the topmost int into its position.
   removeAll(Collection)   - 
Method in class sim.util. Bag 
  
   removeAllInspectors(boolean)   - 
Method in class sim.display. Console 
 Stops and removes all inspectors.
   removeAllInspectors(boolean)   - 
Method in class sim.display. SimpleController 
 Stops and removes all inspectors.
   removeAllNodes()   - 
Method in class sim.field.network. Network 
 Synonym for clear(), here only for backward-compatibility.
   removeAllSeries()   - 
Method in class sim.util.media.chart. ChartGenerator 
 Deletes all series from the chart.
   removeAppPreferences(GUIState, String)   - 
Static method in class sim.display. Prefs 
 Deletes certain app-specific preferences for MASON, with the given additional prefix as a namespace.
   removeEdge(Edge)   - 
Method in class sim.field.network. Network 
 Removes an edge and returns it.
   removeEmptyBags   - 
Variable in class sim.field.grid. DenseGrid2D 
 Should we remove bags in the field if they have been emptied, and let them GC, or should
        we keep them around?
   removeEmptyBags   - 
Variable in class sim.field. SparseField 
 Should we remove bags in the field if they have been emptied, and let them GC, or should
        we keep them around?   This doesn't include the allObjects bag.
   removeFromAsynchronousRegistry(AsynchronousSteppable)   - 
Method in class sim.engine. SimState 
 Unregisters an AsynchronousSteppable from the asynchronous registry.
   removeGlobalAttribute(int)   - 
Method in class sim.util.media.chart. ChartGenerator 
 Remooves the global attribute at the given index and returns it.
   removeGlobalPreferences(String)   - 
Static method in class sim.display. Prefs 
 Deletes certain global preferences for MASON, with the given additional prefix as a namespace.
   removeListeners()   - 
Method in class sim.display. Display2D.InnerDisplay2D 
  Deprecated.   Use Display2D.removeListeners instead. 
   removeListeners()   - 
Method in class sim.display. Display2D 
 Removes all mouse listeners, mouse motion listeners, and Key listeners from this component.
   removeMultiply(Object)   - 
Method in class sim.util. Bag 
 Removes multiple instantiations of an object
   removeNode(Object)   - 
Method in class sim.field.network. Network 
 Removes a node, deleting all incoming and outgoing edges from the Field as well.
   removeNondestructively(int)   - 
Method in class sim.util. Bag 
 Removes the object at the given index, shifting the other objects down.
   removeNondestructively(int)   - 
Method in class sim.util. DoubleBag 
 Removes the double at the given index, shifting the other doubles down.
   removeNondestructively(int)   - 
Method in class sim.util. IntBag 
 Removes the int at the given index, shifting the other ints down.
   removeObjectAtLocation(Object, int, int)   - 
Method in class sim.field.grid. DenseGrid2D 
  
   removeObjectAtLocation(Object, Int2D)   - 
Method in class sim.field.grid. DenseGrid2D 
  
   removeObjectMultiplyAtLocation(Object, int, int)   - 
Method in class sim.field.grid. DenseGrid2D 
  
   removeObjectMultiplyAtLocation(Object, Int2D)   - 
Method in class sim.field.grid. DenseGrid2D 
  
   removeObjectsAtLocation(Double2D)   - 
Method in class sim.field.continuous. Continuous2D 
 Removes objects at exactly the given location, and returns a bag of them, or null of no objects are at that location.
   removeObjectsAtLocation(Double3D)   - 
Method in class sim.field.continuous. Continuous3D 
 Removes objects at exactly the given location, and returns a bag of them, or null of no objects are at that location.
   removeObjectsAtLocation(int, int)   - 
Method in class sim.field.grid. DenseGrid2D 
 Removes all the objects stored at the given location and returns them as a Bag (which you are free to modify).
   removeObjectsAtLocation(Int2D)   - 
Method in class sim.field.grid. DenseGrid2D 
 Removes all the objects stored at the given location and returns them as a Bag (which you are free to modify).
   removeObjectsAtLocation(int, int)   - 
Method in class sim.field.grid. SparseGrid2D 
 Removes all the objects stored at the given location and returns them as a Bag (which you are free to modify).
   removeObjectsAtLocation(int, int, int)   - 
Method in class sim.field.grid. SparseGrid3D 
 Removes all the objects stored at the given location and returns them as a Bag (which you are free to modify).
   removeObjectsAtLocation(Object)   - 
Method in class sim.field. SparseField 
 Removes objects at the given location, and returns a bag of them, or null of no objects are at that location.
   removeSeries(int)   - 
Method in class sim.util.media.chart. ChartGenerator 
 Override this to remove a series from the chart.
   removeSeries(int)   - 
Method in class sim.util.media.chart. HistogramGenerator 
  
   removeSeries(int)   - 
Method in class sim.util.media.chart. ScatterPlotGenerator 
  
   removeSeries(int)   - 
Method in class sim.util.media.chart. TimeSeriesChartGenerator 
  
   replaceLargeBags   - 
Variable in class sim.field.grid. DenseGrid2D 
 When a bag drops to one quarter capacity, should we replace it with a new bag?
   replaceLargeBags   - 
Variable in class sim.field. SparseField 
 When a bag drops to one quarter capacity, should we replace it with a new bag?  This doesn't include the allObjects bag.
   REPLACEMENT_BAG_RATIO   - 
Static variable in class sim.field.grid. DenseGrid2D 
 A bag to be replaced will be shrunk to this ratio if  replaceLargeBags  is true
   REPLACEMENT_BAG_RATIO   - 
Static variable in class sim.field. SparseField 
 A bag to be replaced will be shrunk to this ratio if  replaceLargeBags  is true
   reset()   - 
Method in class sim.display. Display2D 
 Resets the Display2D so it reschedules itself and clears out all selections.
   reset()   - 
Method in class sim.display3d. Display3D 
 Resets the Display3D so it reschedules itself.
   reset()   - 
Method in class sim.engine. Schedule 
 Empties out the schedule and resets it to a pristine state BEFORE_SIMULATION, with steps = 0.
   reset(boolean)   - 
Method in class sim.field.network. Network 
 Resets the network, clearing it of nodes and edges.
   reset()   - 
Method in class sim.portrayal. FieldPortrayal 
  Deprecated.   Use setDirtyField(false); 
   reset()   - 
Method in class sim.portrayal.grid. FastHexaObjectGridPortrayal2D 
  Deprecated.   Resets the underlying FastHexaValueGridPortrayal2D. 
   reset()   - 
Method in class sim.portrayal.grid. FastObjectGridPortrayal2D 
  Deprecated.   Use setDirtyField(false);
        Resets the underlying FastValueGridPortrayal2D. 
   resetCountdown()   - 
Method in class sim.engine. MultiStep 
 If we're counting down, then this resets the countdown.
   resetQueues()   - 
Method in class sim.display. GUIState 
 Don't call this unless you know what you're doing.
   resetTransform()   - 
Method in class sim.display3d. Display3D 
 Resets the global model transform to its default value (identity).
   resetTransform()   - 
Method in class sim.portrayal3d. FieldPortrayal3D 
 Resets the internal transform to the value of getDefaultTransform()
   resetTransform()   - 
Method in class sim.portrayal3d.simple. TransformedPortrayal3D 
 Resets the internal transform to the value of getDefaultTransform()
   resize(int)   - 
Method in class sim.util. Bag 
 Resizes the internal array to at least the requested size.
   resize(double)   - 
Method in class sim.util. Double2D 
 Scales the vector to length "dist"
   resize(int)   - 
Method in class sim.util. DoubleBag 
  
   resize(int)   - 
Method in class sim.util. IntBag 
  
   resize(double)   - 
Method in class sim.util. MutableDouble2D 
 Sets my length, which should be >= 0.
   resize(double)   - 
Method in class sim.util. MutableDouble3D 
 Sets my length, which should be >= 0.
   resume()   - 
Method in class sim.engine. AsynchronousSteppable 
 Fires up the AsynchronousSteppable after a pause().
   retainAll(Collection)   - 
Method in class sim.util. Bag 
  
   reverse()   - 
Method in class sim.util. Bag 
 Reverses order of the elements in the Bag
   reverse()   - 
Method in class sim.util. DoubleBag 
 Reverses order of the elements in the DoubleBag
   reverse()   - 
Method in class sim.util. IntBag 
 Reverses order of the elements in the IntBag
   reverseAllEdges()   - 
Method in class sim.field.network. Network 
 This reverse the direction of all edges in the graph.
   reviseColor(Color, double)   - 
Method in class sim.util.media.chart. SeriesAttributes 
 Given an opaque color and a desired opacity (from 0.0 to 1.0), returns a new color of the same tint but with
        the given opacity.
   reviseStopper(Stoppable)   - 
Method in class sim.portrayal. Inspector 
 Gives the user a chance to wrap the Inspector's stopper in a larger stopper,
        which will then get registered with the Inspector; this larger stopper is
        also what is passed into Inspector.createFrame(...).
   rint()   - 
Method in class sim.field.grid. DoubleGrid2D 
 Sets each value in the grid to rint(value).
   rint()   - 
Method in class sim.field.grid. DoubleGrid3D 
 Sets each value in the grid to rint(value).
   root   - 
Variable in class sim.display3d. Display3D 
 The root scene graph node in the Java3D universe.
   rotate(double)   - 
Method in class sim.util. Double2D 
 Rotates the Double2D by theta radians
   rotate(double)   - 
Method in class sim.util. MutableDouble2D 
 Rotates me by theta radians, returning me.
   rotateX(double)   - 
Method in class sim.display3d. Display3D 
 Modifies the global model transform by rotating along the current X axis the provided number of degrees.
   rotateX(double)   - 
Method in class sim.portrayal3d. FieldPortrayal3D 
 Modifies the internal transform by rotating along the current X axis the provided number of degrees.
   rotateX(double)   - 
Method in class sim.portrayal3d.simple. TransformedPortrayal3D 
 Modifies the internal transform by rotating along the current X axis the provided number of degrees.
   rotateY(double)   - 
Method in class sim.display3d. Display3D 
 Modifies the global model transform by rotating along the current Y axis the provided number of degrees.
   rotateY(double)   - 
Method in class sim.portrayal3d. FieldPortrayal3D 
 Modifies the internal transform by rotating along the current Y axis the provided number of degrees.
   rotateY(double)   - 
Method in class sim.portrayal3d.simple. TransformedPortrayal3D 
 Modifies the internal transform by rotating along the current Y axis the provided number of degrees.
   rotateZ(double)   - 
Method in class sim.display3d. Display3D 
 Modifies the global model transform by rotating along the current Z axis the provided number of degrees.
   rotateZ(double)   - 
Method in class sim.portrayal3d. FieldPortrayal3D 
 Modifies the internal transform by rotating along the current Z axis the provided number of degrees.
   rotateZ(double)   - 
Method in class sim.portrayal3d.simple. TransformedPortrayal3D 
 Modifies the internal transform by rotating along the current Z axis the provided number of degrees.
   run(boolean)   - 
Method in class sim.engine. AsynchronousSteppable 
 This method should enter the parallel thread's loop.
 
 
    
 S  
 
   save(Preferences)   - 
Static method in class sim.display. Prefs 
 Saves a given Preferences.
   savePreferences(Preferences)   - 
Method in class sim.display3d. Display3D.OptionPane3D 
 Saves the Option Pane Preferences to a given Preferences Node
   Scalable2D   - Interface in  sim.portrayal  A Scalable2D object provides a scale, and can have the same changed.   scale(double)   - 
Method in class sim.display3d. Display3D 
 Modifies the global model transform by uniformly scaling it in all directions by the provided amount.
   scale(double, double, double)   - 
Method in class sim.display3d. Display3D 
 Modifies the internal transform by scaling it in a nonuniform fashion.
   scale   - 
Variable in class sim.portrayal.simple. OrientedPortrayal2D 
 The pre-scaling length
   scale   - 
Variable in class sim.portrayal.simple. OvalPortrayal2D 
  
   scale   - 
Variable in class sim.portrayal.simple. RectanglePortrayal2D 
  
   scale   - 
Variable in class sim.portrayal.simple. ShapePortrayal2D 
  
   scale(double)   - 
Method in class sim.portrayal3d. FieldPortrayal3D 
 Modifies the internal transform by uniformly scaling it in all directions by the provided amount.
   scale(double, double, double)   - 
Method in class sim.portrayal3d. FieldPortrayal3D 
 Modifies the internal transform by scaling it in a nonuniform fashion.
   scale(double)   - 
Method in class sim.portrayal3d.simple. TransformedPortrayal3D 
 Modifies the internal transform by uniformly scaling it in all directions by the provided amount.
   scale(double, double, double)   - 
Method in class sim.portrayal3d.simple. TransformedPortrayal3D 
 Modifies the internal transform by scaling it in a nonuniform fashion.
   SCALE_WHEN_SMALLER   - 
Static variable in class sim.portrayal.network. SimpleEdgePortrayal2D 
  
   SCALE_WHEN_SMALLER   - 
Static variable in class sim.portrayal.simple. LabelledPortrayal2D 
  
   scaleField   - 
Variable in class sim.display. Display2D 
 The field for scaling values
   scaleField   - 
Variable in class sim.display3d. Display3D 
 The button which starts or stops a movie
   scalex   - 
Variable in class sim.portrayal.simple. LabelledPortrayal2D 
 The pre-scaling offset from the object's origin.
   scaley   - 
Variable in class sim.portrayal.simple. LabelledPortrayal2D 
 The pre-scaling offset from the object's origin.
   ScatterPlotGenerator   - Class in  sim.util.media.chart      ScatterPlotGenerator()   - 
Constructor for class sim.util.media.chart. ScatterPlotGenerator 
  
   ScatterPlotSeriesAttributes   - Class in  sim.util.media.chart      ScatterPlotSeriesAttributes(ChartGenerator, String, int, double[][], SeriesChangeListener)   - 
Constructor for class sim.util.media.chart. ScatterPlotSeriesAttributes 
 Produces a ScatterPlotSeriesAttributes object with the given generator, series name, series index,
        and desire to display margin options.
   sceneGraphCreated()   - 
Method in class sim.display3d. Display3D 
 A hook for people who want to modify the scene graph just after it's been created (when the
        user pressed the start button usually; or if the user dynamicallly attaches or detaches
        portrayals) but before the root has been attached to the universe and before the
        canvas has started rendering.
   Schedule   - Class in  sim.engine  Schedule defines a threadsafe scheduling queue in which events can be scheduled to occur
   at future time.   Schedule()   - 
Constructor for class sim.engine. Schedule 
 Creates a Schedule.
   schedule   - 
Variable in class sim.engine. SimState 
 SimState's schedule
   Schedule.Key   - Class in  sim.engine  Timestamps stored as keys in the heap.   Schedule.Key(double, int)   - 
Constructor for class sim.engine. Schedule.Key 
  
   scheduleAtEnd(Steppable)   - 
Method in class sim.display. GUIState 
 Schedules an item to occur when the user stops the simulator (when the stop() method is executed), when it stops on its own accord, or when the user has load()ed another simulation to replace it.
   scheduleAtExtreme(Steppable, boolean)   - 
Method in class sim.display. GUIState 
  Deprecated.   use scheduleAtStart and scheduleAtEnd instead 
   scheduleAtStart(Steppable)   - 
Method in class sim.display. GUIState 
 Schedules an item to occur when the user starts the simulator (when the start() method is executed) or loads one (when load() is executed).
   scheduleComplete()   - 
Method in class sim.engine. Schedule 
 Returns true if the schedule has nothing left to do.
   scheduleImmediate(boolean, Steppable)   - 
Method in class sim.display. GUIState 
  Deprecated.   use scheduleImmediatelyBefore and scheduleImmediatelyAfter instead 
   scheduleImmediatelyAfter(Steppable)   - 
Method in class sim.display. GUIState 
 Schedules an item to occur (in no particular order) immediately after
        the schedule is stepped on the next time step (not including blank steps).
   scheduleImmediatelyBefore(Steppable)   - 
Method in class sim.display. GUIState 
 Schedules an item to occur (in no particular order) immediately before 
        the schedule is stepped on the next time step (not including blank steps).
   scheduleImmediateRepeat(boolean, Steppable)   - 
Method in class sim.display. GUIState 
  Deprecated.   use scheduleRepeatingImmediatelyBefore and scheduleRepeatingImmediatelyAfter instead 
   scheduleOnce(Steppable)   - 
Method in class sim.engine. Schedule 
 Schedules the event to occur at getTime() + 1.0, 0 ordering.
   scheduleOnce(Steppable, int)   - 
Method in class sim.engine. Schedule 
 Schedules the event to occur at getTime() + 1.0, and in the ordering provided.
   scheduleOnce(double, Steppable)   - 
Method in class sim.engine. Schedule 
 Schedules the event to occur at the provided time, 0 ordering.
   scheduleOnce(double, int, Steppable)   - 
Method in class sim.engine. Schedule 
 Schedules the event to occur at the provided time, and in the ordering provided.
   scheduleOnce(Schedule.Key, Steppable)   - 
Method in class sim.engine. Schedule 
 Schedules an item.
   scheduleOnceIn(double, Steppable)   - 
Method in class sim.engine. Schedule 
 Schedules the event to occur at getTime() + delta, 0 ordering.
   scheduleOnceIn(double, Steppable, int)   - 
Method in class sim.engine. Schedule 
 Schedules the event to occur at getTime() + delta, and in the ordering provided.
   scheduleRepeating(Steppable)   - 
Method in class sim.engine. Schedule 
 Schedules the event to recur at an interval of 1.0 starting at getTime() + 1.0, and at 0 ordering.
   scheduleRepeating(Steppable, double)   - 
Method in class sim.engine. Schedule 
 Schedules the event to recur at the specified interval starting at getTime() + interval, and at 0 ordering.
   scheduleRepeating(Steppable, int, double)   - 
Method in class sim.engine. Schedule 
 Schedules the event to recur at the specified interval starting at getTime() + interval, and at the provided ordering.
   scheduleRepeating(double, Steppable)   - 
Method in class sim.engine. Schedule 
 Schedules the event to recur at the specified interval starting at the provided time, and at 0 ordering.
   scheduleRepeating(double, Steppable, double)   - 
Method in class sim.engine. Schedule 
 Schedules the event to recur at the specified interval starting at the provided time, 
        in ordering 0.
   scheduleRepeating(double, int, Steppable)   - 
Method in class sim.engine. Schedule 
 Schedules the event to recur at an interval of 1.0 starting at the provided time, 
        and in the ordering provided.
   scheduleRepeating(double, int, Steppable, double)   - 
Method in class sim.engine. Schedule 
 Schedules the event to recur at the specified interval starting at the provided time, 
        and in the ordering provided.
   scheduleRepeatingImmediatelyAfter(Steppable)   - 
Method in class sim.display. GUIState 
 Schedules an item to occur (in no particular order) immediately after
        all future steps the Schedule takes (not including blank steps).
   scheduleRepeatingImmediatelyBefore(Steppable)   - 
Method in class sim.display. GUIState 
 Schedules an item to occur (in no particular order) immediately before
        all future steps the Schedule takes (not including blank steps).
   seal()   - 
Method in class sim.engine. Schedule 
 Seals the schedule: after a schedule is sealed, no further Steppables may be scheduled on it.
   secondPoint   - 
Variable in class sim.portrayal.network. EdgeDrawInfo2D 
 A pre-scaled point to draw to.
   seed()   - 
Method in class sim.engine. SimState 
 Returns the seed set by the doLoop(...) facility and by the constructor.
   selected   - 
Variable in class sim.portrayal. DrawInfo2D 
  
   SelectionBehavior   - Class in  sim.display3d  A behavior added to Display3Ds which enables Portrayal3Ds to be selected (via Java3D picking).   SelectionBehavior(Canvas3D, BranchGroup, Bounds, GUIState)   - 
Constructor for class sim.display3d. SelectionBehavior 
  
   Sequence   - Class in  sim.engine  Sequence is a Steppable which, on being stepped, in turn stepps several other
   Steppable objects in turn.   Sequence(Steppable[])   - 
Constructor for class sim.engine. Sequence 
 Assumes all the steps are filled.
   seriesAttributes   - 
Variable in class sim.util.media.chart. ChartGenerator 
 A holder for series attributes components
   SeriesAttributes   - Class in  sim.util.media.chart  The superclass for the series-attributes widgets used by subclasses of ChartGenerator to let the user
    control individual series' features.   SeriesAttributes(ChartGenerator, String, int, SeriesChangeListener)   - 
Constructor for class sim.util.media.chart. SeriesAttributes 
 Builds a SeriesAttributes with the provided generator, name for the series, and index for the series.
   set(int, int, double)   - 
Method in class sim.field.grid. DoubleGrid2D 
 Sets location (x,y) to val
   set(int, int, int, double)   - 
Method in class sim.field.grid. DoubleGrid3D 
 Sets location (x,y,z) to val
   set(int, int, int)   - 
Method in class sim.field.grid. IntGrid2D 
 Sets location (x,y) to val
   set(int, int, int, int)   - 
Method in class sim.field.grid. IntGrid3D 
 Sets location (x,y) to val
   set(int, int, Object)   - 
Method in class sim.field.grid. ObjectGrid2D 
 Sets location (x,y) to val
   set(int, int, int, Object)   - 
Method in class sim.field.grid. ObjectGrid3D 
  
   set(int, Object)   - 
Method in class sim.util. Bag 
  
   set(int, double)   - 
Method in class sim.util. DoubleBag 
  
   set(int, int)   - 
Method in class sim.util. IntBag 
  
   setAbridgedComponent(Component)   - 
Method in class sim.util.gui. DisclosurePanel 
  
   setAdjustsThickness(boolean)   - 
Method in class sim.portrayal.network. SimpleEdgePortrayal2D 
  
   setAppearance(TransformGroup, Appearance)   - 
Method in class sim.portrayal3d.simple. PrimitivePortrayal3D 
 Sets the Appearance of the portrayal.
   setAppearanceFlags(Appearance)   - 
Static method in class sim.portrayal3d. SimplePortrayal3D 
 Sets a variety of flags on an Appearance so that its features can be modified
        when the scene is live.
   setAuxiliaryField(SparseField2D)   - 
Method in class sim.portrayal.network. SpatialNetwork2D 
  
   setAuxillaryField(SparseField2D)   - 
Method in class sim.portrayal.network. SpatialNetwork2D 
  
   setBackdrop(Paint)   - 
Method in class sim.display. Display2D 
 Specify the backdrop color or other paint.
   setBackdrop(Appearance)   - 
Method in class sim.display3d. Display3D 
 Sets a general appearance for a spherical backdrop, turns on the backdrop checkbox,  and enables the backdrop checkbox.
   setBackdrop(Color)   - 
Method in class sim.display3d. Display3D 
 Sets the color for a flat backdrop, turns on the backdrop checkbox,  and enables the backdrop checkbox.
   setBackdrop(Image, boolean)   - 
Method in class sim.display3d. Display3D 
 Sets the image for a backdrop (spherical or flat), turns on the backdrop checkbox,  and enables the backdrop checkbox
   setBaseWidth(double)   - 
Method in class sim.portrayal.network. SimpleEdgePortrayal2D 
 Sets the width of the base of the triangle used in drawing the directed edge -- by default, this is 0 (a simple line is drawn).
   setBucketLabels(String[])   - 
Method in class sim.util.gui. MiniHistogram 
 Sets labels for the buckets provided in setBuckets.
   setBuckets(double[])   - 
Method in class sim.util.gui. MiniHistogram 
 Sets the displayed bucket array.
   setBucketsAndLabels(double[], String[])   - 
Method in class sim.util.gui. MiniHistogram 
  
   setBuffering(int)   - 
Method in class sim.portrayal. FieldPortrayal2D 
 Sets whether or not the FieldPortrayal2D will use a buffering "trick" to draw quickly.
   setBuffering(int)   - 
Method in class sim.portrayal.grid. FastHexaObjectGridPortrayal2D 
  
   setBuffering(int)   - 
Method in class sim.portrayal.grid. FastObjectGridPortrayal2D 
  
   setCanShowToolTips(boolean)   - 
Method in class sim.display3d. ToolTipBehavior 
 Sets whether or not the behavior may show tool tips.
   setCircleShowing(boolean)   - 
Method in class sim.portrayal.simple. CircledPortrayal2D 
  
   setCircleShowing(boolean)   - 
Method in class sim.portrayal3d.simple. CircledPortrayal3D 
  
   setClipping(boolean)   - 
Method in class sim.display. Display2D 
 Sets the Display2D to clip or to not clip to the user-specified height and width when drawing
   setColor(Color)   - 
Method in class sim.util.gui. ColorWell 
  
   setColorTable(Color[])   - 
Method in class sim.util.gui. SimpleColorMap 
 Specifies that if a value (cast into an int) in the IntGrid2D or DoubleGrid2D falls in the range 0 ...
   setCompressionLevel(int)   - 
Method in class sim.util.media. PNGEncoder 
 Set the compression level to use
   setCurrentDisplay(Display3D)   - 
Method in class sim.portrayal3d. FieldPortrayal3D 
  
   setCurrentDisplay(Display3D)   - 
Method in interface sim.portrayal3d. Portrayal3D 
 Sets the current Display3D.
   setCurrentDisplay(Display3D)   - 
Method in class sim.portrayal3d.simple. CircledPortrayal3D 
 Sets the current display both here and in the child.
   setCurrentDisplay(Display3D)   - 
Method in class sim.portrayal3d.simple. LabelledPortrayal3D 
 Sets the current display both here and in the child.
   setCurrentDisplay(Display3D)   - 
Method in class sim.portrayal3d.simple. SharedPortrayal3D 
 Sets the current display both here and in the child.
   setCurrentDisplay(Display3D)   - 
Method in class sim.portrayal3d.simple. TransformedPortrayal3D 
 Sets the current display both here and in the child.
   setCurrentDisplay(Display3D)   - 
Method in class sim.portrayal3d. SimplePortrayal3D 
  
   setCurrentFieldPortrayal(FieldPortrayal3D)   - 
Method in class sim.portrayal3d.simple. CircledPortrayal3D 
 Sets the current field portrayal both here and in the child.
   setCurrentFieldPortrayal(FieldPortrayal3D)   - 
Method in class sim.portrayal3d.simple. LabelledPortrayal3D 
 Sets the current field portrayal both here and in the child.
   setCurrentFieldPortrayal(FieldPortrayal3D)   - 
Method in class sim.portrayal3d.simple. SharedPortrayal3D 
 Sets the current field portrayal both here and in the child.
   setCurrentFieldPortrayal(FieldPortrayal3D)   - 
Method in class sim.portrayal3d.simple. TransformedPortrayal3D 
 Sets the current field portrayal both here and in the child.
   setCurrentFieldPortrayal(FieldPortrayal3D)   - 
Method in class sim.portrayal3d. SimplePortrayal3D 
  
   setDashPattern(int)   - 
Method in class sim.util.media.chart. TimeSeriesAttributes 
  
   setData(ValueGridCellInfo, float[], float[], int, int, int)   - 
Method in class sim.portrayal3d.grid.quad. MeshPortrayal 
  
   setData(ValueGridCellInfo, float[], float[], int, int, int)   - 
Method in class sim.portrayal3d.grid.quad. QuadPortrayal 
  
   setData(ValueGridCellInfo, float[], float[], int, int, int)   - 
Method in class sim.portrayal3d.grid.quad. TilePortrayal 
  
   setDataCuller(DataCuller)   - 
Method in class sim.util.media.chart. TimeSeriesChartGenerator 
  
   setDestroysThreads(boolean)   - 
Method in class sim.engine. ParallelSequence 
  
   setDirtyField(boolean)   - 
Method in class sim.portrayal. FieldPortrayal 
  
   setDirtyField(boolean)   - 
Method in class sim.portrayal.grid. FastHexaObjectGridPortrayal2D 
  
   setDirtyField(boolean)   - 
Method in class sim.portrayal.grid. FastObjectGridPortrayal2D 
  
   setDisclosed(boolean)   - 
Method in class sim.util.gui. DisclosurePanel 
  
   setDisclosedComponent(Component)   - 
Method in class sim.util.gui. DisclosurePanel 
  
   setDisplayingToroidally(boolean)   - 
Method in class sim.portrayal.continuous. ContinuousPortrayal2D 
 Set this to TRUE to cause the portrayal to display objects multiply (in a toroidal fashion)
        if they overlap on the edges of the field.
   setDomainAxisLabel(String)   - 
Method in class sim.util.media.chart. ChartGenerator 
  Deprecated.   Sets the name of the Domain Axis label  -- usually this is the X axis. 
   setDomainAxisRange(double, double)   - 
Method in class sim.util.media.chart. ChartGenerator 
  Deprecated.     
   setDrawFilled(boolean)   - 
Method in class sim.portrayal.simple. OrientedPortrayal2D 
  
   setDrawPolicy(DrawPolicy)   - 
Method in class sim.portrayal.grid. SparseGridPortrayal2D 
  
   setEditedColor(Color)   - 
Method in class sim.util.gui. NumberTextField 
  
   setEditedColor(Color)   - 
Method in class sim.util.gui. PropertyField 
  
   setEnabled(boolean)   - 
Method in class sim.util.gui. NumberTextField 
  
   setEnabled(boolean)   - 
Method in class sim.util.gui. PropertyField 
  
   setEncodeAlpha(boolean)   - 
Method in class sim.util.media. PNGEncoder 
 Set the alpha encoding on or off.
   setField(Object)   - 
Method in class sim.portrayal.continuous. ContinuousPortrayal2D 
  
   setField(Object)   - 
Method in class sim.portrayal. FieldPortrayal 
 Sets the field, and sets the dirtyField flag to true.
   setField(Object)   - 
Method in class sim.portrayal.grid. FastHexaObjectGridPortrayal2D 
  
   setField(Object)   - 
Method in class sim.portrayal.grid. FastObjectGridPortrayal2D 
  
   setField(Object)   - 
Method in class sim.portrayal.grid. ObjectGridPortrayal2D 
  
   setField(Object)   - 
Method in class sim.portrayal.grid. SparseGridPortrayal2D 
  
   setField(Object)   - 
Method in class sim.portrayal.grid. ValueGridPortrayal2D 
  
   setField(Object)   - 
Method in class sim.portrayal.network. NetworkPortrayal2D 
  
   setField(Object)   - 
Method in class sim.portrayal3d.continuous. ContinuousPortrayal3D 
  
   setField(Object)   - 
Method in class sim.portrayal3d.grid. ObjectGridPortrayal3D 
  
   setField(Object)   - 
Method in class sim.portrayal3d.grid. SparseGrid2DPortrayal3D 
  
   setField(Object)   - 
Method in class sim.portrayal3d.grid. SparseGridPortrayal3D 
  
   setField(Object)   - 
Method in class sim.portrayal3d.grid. ValueGrid2DPortrayal3D 
  
   setField(Object)   - 
Method in class sim.portrayal3d.grid. ValueGridPortrayal3D 
  
   setFillColor(Color)   - 
Method in class sim.util.media.chart. HistogramSeriesAttributes 
  
   setFillOpacity(double)   - 
Method in class sim.util.media.chart. HistogramSeriesAttributes 
  
   setFilter(int)   - 
Method in class sim.util.media. PNGEncoder 
 Set the filter to use
   setFrame(Paint)   - 
Method in class sim.portrayal.continuous. ContinuousPortrayal2D 
 If you provide a Paint, a thin frame of this paint will be drawn around the (0,0,width,height) space of
        the field.
   setGrowTrailOnlyWhenSelected(boolean)   - 
Method in class sim.portrayal.simple. TrailedPortrayal2D 
  Deprecated.   use setOnlyGrowTrailWhenSelected 
   setHistogramType(HistogramType)   - 
Method in class sim.util.media.chart. HistogramGenerator 
  
   setImage(Image)   - 
Method in class sim.portrayal3d.grid. ValueGrid2DPortrayal3D 
 Set the appearance to a fully opaque image.
   setImage(Image)   - 
Method in class sim.util.media. PNGEncoder 
 Set the image to be encoded
   setImmutableField(boolean)   - 
Method in class sim.portrayal. FieldPortrayal 
 Specifies that the underlying field is (or is not) to be assumed unchanging --
        thus there's no reason to update once we're created.
   setImmutableField(boolean)   - 
Method in class sim.portrayal.grid. FastHexaObjectGridPortrayal2D 
  
   setImmutableField(boolean)   - 
Method in class sim.portrayal.grid. FastObjectGridPortrayal2D 
  
   setIncrementSeedOnPlay(boolean)   - 
Method in class sim.display. Console 
  Deprecated.   renamed to setIncrementSeedOnStop 
   setIncrementSeedOnPlay(boolean)   - 
Method in class sim.display. SimpleController 
  Deprecated.   renamed to setIncrementSeedOnStop 
   setIncrementSeedOnStop(boolean)   - 
Method in class sim.display. Console 
  
   setIncrementSeedOnStop(boolean)   - 
Method in class sim.display. SimpleController 
  
   setInfo(Object)   - 
Method in class sim.field.network. Edge 
  
   setInitialValue(double)   - 
Method in class sim.util.gui. NumberTextField 
  
   setInspectors(Bag, Bag)   - 
Method in class sim.display. Console 
 Adds new inspectors to the Console's list, given the provided inspectors, their portrayals, and appropriate names for them.
   setInspectors(Bag, Bag)   - 
Method in interface sim.display. Controller 
 Replaces current inspectors with the ones provided.
   setInspectors(Bag, Bag)   - 
Method in class sim.display. SimpleController 
 Adds new inspectors to the Console's list, given the provided inspectors, their portrayals, and appropriate names for them.
   setJob(long)   - 
Method in class sim.engine. SimState 
  
   setLabelScale(double)   - 
Method in class sim.portrayal3d.simple. LabelledPortrayal3D 
  
   setLabelScaling(int)   - 
Method in class sim.portrayal.network. SimpleEdgePortrayal2D 
  
   setLabelScaling(int)   - 
Method in class sim.portrayal.simple. LabelledPortrayal2D 
  
   setLabelShowing(boolean)   - 
Method in class sim.portrayal.simple. LabelledPortrayal2D 
  
   setLabelShowing(boolean)   - 
Method in class sim.portrayal3d.simple. LabelledPortrayal3D 
  
   setLength(double)   - 
Method in class sim.portrayal.simple. TrailedPortrayal2D 
 Sets the length of the trail in TIME.
   setLength(double)   - 
Method in class sim.util. MutableDouble2D 
  Deprecated.   use resize instead [renaming] 
   setLength(double)   - 
Method in class sim.util. MutableDouble3D 
  Deprecated.   use resize instead [renaming] 
   setLevels(double, double, Color, Color)   - 
Method in class sim.util.gui. SimpleColorMap 
 Sets the color levels for the ValueGridPortrayal2D values for use by the default getColor(...)
        method.
   setLineShowing(boolean)   - 
Method in class sim.portrayal.simple. OrientedPortrayal2D 
  Deprecated.   use setOrientationShowing() 
   setLocation(int, int)   - 
Method in class sim.util. MutableInt2D 
  Deprecated.   use setTo 
   setLocation(Point)   - 
Method in class sim.util. MutableInt2D 
  Deprecated.   use setTo 
   setLocation(Int2D)   - 
Method in class sim.util. MutableInt2D 
  Deprecated.   use setTo 
   setLocation(MutableInt2D)   - 
Method in class sim.util. MutableInt2D 
  Deprecated.   use setTo 
   setLocation(int, int, int)   - 
Method in class sim.util. MutableInt3D 
  Deprecated.   use setTo 
   setLocation(Int3D)   - 
Method in class sim.util. MutableInt3D 
  Deprecated.   use setTo 
   setLocation(MutableInt3D)   - 
Method in class sim.util. MutableInt3D 
  Deprecated.   use setTo 
   setMap(ColorMap)   - 
Method in class sim.portrayal.grid. FastHexaObjectGridPortrayal2D 
  
   setMap(ColorMap)   - 
Method in class sim.portrayal.grid. FastObjectGridPortrayal2D 
  
   setMap(ColorMap)   - 
Method in class sim.portrayal.grid. ValueGridPortrayal2D 
  
   setMap(ColorMap)   - 
Method in class sim.portrayal3d.grid.quad. QuadPortrayal 
  
   setMap(ColorMap)   - 
Method in class sim.portrayal3d.grid. ValueGridPortrayal3D 
  
   setMaximumJump(double)   - 
Method in class sim.portrayal.simple. TrailedPortrayal2D 
 Sets the maximum percentage of either the width or height of the field that can be 
        jumped between two successive object locations before it's considered to be a huge leap and that segment won't be drawn.
   setName(String)   - 
Method in class sim.util.media.chart. HistogramSeriesAttributes 
 It's very expensive to call this function (O(n)) because JFreeChart has no way of changing the
        name of a histogram dataset series, and so we must rebuild all of it from scratch.
   setName(String)   - 
Method in class sim.util.media.chart. ScatterPlotSeriesAttributes 
  
   setName(String)   - 
Method in class sim.util.media.chart. SeriesAttributes 
 Sets the name of the series.
   setName(String)   - 
Method in class sim.util.media.chart. TimeSeriesAttributes 
  
   setNewMenuAllowed(boolean)   - 
Method in class sim.display. Console 
  
   setNumBins(int)   - 
Method in class sim.util.media.chart. HistogramSeriesAttributes 
  
   setNumStepsPerStepButtonPress(int)   - 
Method in class sim.display. Console 
 Sets the number of steps per stepp button press.
   setObjectLocation(Object, Double2D)   - 
Method in class sim.field.continuous. Continuous2D 
  
   setObjectLocation(Object, Double3D)   - 
Method in class sim.field.continuous. Continuous3D 
  
   setObjectLocation(Object, int, int)   - 
Method in class sim.field.grid. SparseGrid2D 
 Changes the location of an object, or adds if it doesn't exist yet.
   setObjectLocation(Object, Int2D)   - 
Method in class sim.field.grid. SparseGrid2D 
 Changes the location of an object, or adds if it doesn't exist yet.
   setObjectLocation(Object, int, int, int)   - 
Method in class sim.field.grid. SparseGrid3D 
 Changes the location of an object, or adds if it doesn't exist yet.
   setObjectLocation(Object, Int3D)   - 
Method in class sim.field.grid. SparseGrid3D 
 Changes the location of an object, or adds if it doesn't exist yet.
   setObjectLocation(Object, Object)   - 
Method in class sim.field. SparseField 
 Changes the location of an object, or adds if it doesn't exist yet.
   setObjectPosition(Object, Point2D.Double, DrawInfo2D)   - 
Method in class sim.portrayal.continuous. ContinuousPortrayal2D 
  
   setObjectPosition(Object, Point2D.Double, DrawInfo2D)   - 
Method in class sim.portrayal. FieldPortrayal2D 
 Moves (or tries to move) the object to an internal location equivalent to the given position on-screen of the
        provided object, assuming that the object exists within the underlying field and that this
        location is acceptable.
   setObjectPosition(Object, Point2D.Double, DrawInfo2D)   - 
Method in class sim.portrayal.grid. HexaSparseGridPortrayal2D 
  
   setObjectPosition(Object, Point2D.Double, DrawInfo2D)   - 
Method in class sim.portrayal.grid. SparseGridPortrayal2D 
  
   setOnlyCircleWhenSelected(boolean)   - 
Method in class sim.portrayal.simple. CircledPortrayal2D 
  
   setOnlyCircleWhenSelected(boolean)   - 
Method in class sim.portrayal3d.simple. CircledPortrayal3D 
  
   setOnlyDrawWhenSelected(boolean)   - 
Method in class sim.portrayal.simple. OrientedPortrayal2D 
  
   setOnlyGrowTrailWhenSelected(boolean)   - 
Method in class sim.portrayal.simple. TrailedPortrayal2D 
 Set this to grow the trail only after the objet has been selected, and delete it when the object has been deselected.
   setOnlyLabelWhenSelected(boolean)   - 
Method in class sim.portrayal.simple. LabelledPortrayal2D 
  
   setOnlyLabelWhenSelected(boolean)   - 
Method in class sim.portrayal3d.simple. LabelledPortrayal3D 
  
   setOnlyShowTrailWhenSelected(boolean)   - 
Method in class sim.portrayal.simple. TrailedPortrayal2D 
 Set this to draw the trail only when the object has been selected (or not).
   setOrientation2D(double)   - 
Method in interface sim.portrayal. Orientable2D 
  
   setOrientationHittable(boolean)   - 
Method in class sim.portrayal.simple. OrientedPortrayal2D 
 Sets whether or not the orientation marker can be hit as part of the object.
   setOrientationShowing(boolean)   - 
Method in class sim.portrayal.simple. OrientedPortrayal2D 
  
   setParent(ValueGridPortrayal2D)   - 
Method in class sim.portrayal.simple. ValuePortrayal2D 
  Deprecated.   does nothing now 
   setPickable(boolean)   - 
Method in class sim.portrayal3d.simple. PrimitivePortrayal3D 
 Sets objects as pickable or not.
   setPickableFlags(Shape3D)   - 
Static method in class sim.portrayal3d. SimplePortrayal3D 
 Utility method which prepares the given Shape3D to be pickable (for selection and inspection).
   setPickableFlags(Geometry)   - 
Static method in class sim.portrayal3d. SimplePortrayal3D 
 Utility method which prepares the given Geometry to be pickable (for selection and inspection).
   setPlaySleep(long)   - 
Method in class sim.display. Console 
 Sets (in milliseconds) how long we should sleep between each step in the play thread.
   setPortrayalForAll(Portrayal)   - 
Method in class sim.portrayal. FieldPortrayal 
 Set the portrayal to null to remove it.
   setPortrayalForClass(Class, Portrayal)   - 
Method in class sim.portrayal. FieldPortrayal 
 Sets a portrayal for a class -- objects must be of EXACTLY this class (not subclasses)
        to respond to this.
   setPortrayalForNonNull(Portrayal)   - 
Method in class sim.portrayal. FieldPortrayal 
 Set the portrayal to null to remove it.
   setPortrayalForNull(Portrayal)   - 
Method in class sim.portrayal. FieldPortrayal 
 Set the portrayal to null to remove it.
   setPortrayalForObject(Object, Portrayal)   - 
Method in class sim.portrayal. FieldPortrayal 
 Sets a portrayal for a class -- objects must be equal(...) to the provided object here
        to respond to this.
   setPortrayalForRemainder(Portrayal)   - 
Method in class sim.portrayal. FieldPortrayal 
 Set the portrayal to null to remove it.
   setPreferencesKey(String)   - 
Method in class sim.display. Display2D 
 If you have more than one Display2D in your simulation and you want them to have
        different preferences, set each to a different key value.
   setPreferencesKey(String)   - 
Method in class sim.display3d. Display3D 
 If you have more than one Display3D in your simulation and you want them to have
        different preferences, set each to a different key value.
   setRangeAxisLabel(String)   - 
Method in class sim.util.media.chart. ChartGenerator 
  Deprecated.   Sets the name of the Range Axis label -- usually this is the Y axis. 
   setRangeAxisRange(double, double)   - 
Method in class sim.util.media.chart. ChartGenerator 
  Deprecated.     
   setRequiresConfirmationToStop(boolean)   - 
Method in class sim.display. Console 
  
   setScale(double)   - 
Method in class sim.display. Display2D 
 Sets the scale (the zoom value) of the Display2D
   setScale(double)   - 
Method in class sim.display3d. Display3D 
 Changes the value of the scale (magnification).
   setScale(double)   - 
Method in class sim.portrayal3d.grid. ValueGridPortrayal3D 
  
   setScale(TransformGroup, double)   - 
Method in class sim.portrayal3d.simple. PrimitivePortrayal3D 
 Sets the Transform3D of the portrayal to a given scaling value, if there *is* a transform (in some cases, such as ValuePortrayal3D, there won't be any).
   setScale2D(double)   - 
Method in interface sim.portrayal. Scalable2D 
  
   setScaling(int)   - 
Method in class sim.portrayal.network. SimpleEdgePortrayal2D 
  
   setSeed(long)   - 
Method in class ec.util. MersenneTwisterFast 
 Initalize the pseudo random number generator.
   setSeed(int[])   - 
Method in class ec.util. MersenneTwisterFast 
 Sets the seed of the MersenneTwister using an array of integers.
   setSeed(long)   - 
Method in class sim.engine. SimState 
  
   setSelected(LocationWrapper, boolean)   - 
Method in class sim.portrayal.continuous. ContinuousPortrayal2D 
  
   setSelected(LocationWrapper, boolean)   - 
Method in class sim.portrayal. FieldPortrayal 
 Selects or deselects all of the provided objects.
   setSelected(Bag, boolean)   - 
Method in class sim.portrayal. FieldPortrayal 
  
   setSelected(LocationWrapper, boolean)   - 
Method in class sim.portrayal.grid. ObjectGridPortrayal2D 
  
   setSelected(LocationWrapper, boolean)   - 
Method in class sim.portrayal.grid. SparseGridPortrayal2D 
  
   setSelected(LocationWrapper, boolean)   - 
Method in interface sim.portrayal. Portrayal 
 Change the portrayal state to reflect the fact that you've 
        been selected or not selected.
   setSelected(LocationWrapper, boolean)   - 
Method in class sim.portrayal.simple. AdjustablePortrayal2D 
  
   setSelected(LocationWrapper, boolean)   - 
Method in class sim.portrayal.simple. CircledPortrayal2D 
  
   setSelected(LocationWrapper, boolean)   - 
Method in class sim.portrayal.simple. FacetedPortrayal2D 
 If portrayAllChildren, Returns true if any ONE of the children returns true.
   setSelected(LocationWrapper, boolean)   - 
Method in class sim.portrayal.simple. LabelledPortrayal2D 
  
   setSelected(LocationWrapper, boolean)   - 
Method in class sim.portrayal.simple. MovablePortrayal2D 
  
   setSelected(LocationWrapper, boolean)   - 
Method in class sim.portrayal.simple. OrientedPortrayal2D 
  
   setSelected(LocationWrapper, boolean)   - 
Method in class sim.portrayal.simple. TrailedPortrayal2D 
  
   setSelected(LocationWrapper, boolean)   - 
Method in class sim.portrayal.simple. TransformedPortrayal2D 
  
   setSelected(LocationWrapper, boolean)   - 
Method in class sim.portrayal. SimplePortrayal2D 
  
   setSelected(LocationWrapper, boolean)   - 
Method in class sim.portrayal3d.grid.quad. QuadPortrayal 
  
   setSelected(LocationWrapper, boolean)   - 
Method in class sim.portrayal3d.simple. CircledPortrayal3D 
  
   setSelected(LocationWrapper, boolean)   - 
Method in class sim.portrayal3d.simple. LabelledPortrayal3D 
  
   setSelected(LocationWrapper, boolean)   - 
Method in class sim.portrayal3d.simple. SharedPortrayal3D 
 Unlikely to be called, as SharedPortrayal3D is not pickable.
   setSelected(LocationWrapper, boolean)   - 
Method in class sim.portrayal3d.simple. TransformedPortrayal3D 
  
   setSelected(LocationWrapper, boolean)   - 
Method in class sim.portrayal3d. SimplePortrayal3D 
 If the object is selected, adds it to a hash table of selected objects for which
        this SimplePortrayal3D's isSelected() method will return TRUE.
   setSelectsAll(boolean, boolean)   - 
Method in class sim.display3d. Display3D 
 Sets whether mouse-clicking results in selecting all picked elements (true) or just the closest one (false).
   setSelectsAll(boolean, boolean)   - 
Method in class sim.display3d. SelectionBehavior 
 Sets whether mouse-clicking results in selecting all picked elements (true) or just the closest one (false).
   setSeriesAttributes(SeriesAttributes[])   - 
Method in class sim.util.media.chart. ChartGenerator 
  
   setSeriesDataset(XYDataset)   - 
Method in class sim.util.media.chart. ChartGenerator 
  
   setSeriesIndex(int)   - 
Method in class sim.util.media.chart. SeriesAttributes 
 Sets the index of the series -- Do NOT call this if you want to move the series order; instead call moveSeries
        in the Generator.
   setShape(int)   - 
Method in class sim.portrayal.network. SimpleEdgePortrayal2D 
 Sets the shape of the edge.
   setShape(int)   - 
Method in class sim.portrayal.simple. OrientedPortrayal2D 
  
   setShape3DFlags(Shape3D)   - 
Static method in class sim.portrayal3d.simple. PrimitivePortrayal3D 
 Sets common Shape3D flags to make its appearance and geometry easy to modify.
   setShapeNum(int)   - 
Method in class sim.util.media.chart. ScatterPlotSeriesAttributes 
  
   setShouldRepeat(boolean)   - 
Method in class sim.display. Console 
 Set whether or not the simualtion should repeat when the stop button is pressed.
   setShowsAmbientLight(boolean)   - 
Method in class sim.display3d. Display3D 
  
   setShowsAxes(boolean)   - 
Method in class sim.display3d. Display3D 
  
   setShowsBackdrop(boolean)   - 
Method in class sim.display3d. Display3D 
  
   setShowsSpotlight(boolean)   - 
Method in class sim.display3d. Display3D 
  
   setStoppable(Stoppable)   - 
Method in class sim.engine. WeakStep 
  
   setStoppable(SeriesChangeListener)   - 
Method in class sim.util.media.chart. SeriesAttributes 
  
   setStretch(double)   - 
Method in class sim.util.media.chart. TimeSeriesAttributes 
  
   setStroke(Stroke)   - 
Method in class sim.portrayal.simple. ShapePortrayal2D 
  
   setStrokeColor(Color)   - 
Method in class sim.util.media.chart. HistogramSeriesAttributes 
  
   setStrokeColor(Color)   - 
Method in class sim.util.media.chart. TimeSeriesAttributes 
  
   setStrokeOpacity(double)   - 
Method in class sim.util.media.chart. HistogramSeriesAttributes 
  
   setSymbolColor(Color)   - 
Method in class sim.util.media.chart. ScatterPlotSeriesAttributes 
  
   setSymbolOpacity(double)   - 
Method in class sim.util.media.chart. ScatterPlotSeriesAttributes 
  
   setText(Object)   - 
Method in class sim.util.gui. HTMLBrowser 
  
   setThickness(double)   - 
Method in class sim.util.media.chart. HistogramSeriesAttributes 
  
   setThickness(double)   - 
Method in class sim.util.media.chart. TimeSeriesAttributes 
  
   setThreadPriority(int)   - 
Method in class sim.display. Console 
  Deprecated.   We may eliminate thread priority as an option 
   setTitle(String)   - 
Method in class sim.util.media.chart. ChartGenerator 
 Sets the title of the chart (and the window frame).
   setTo(double)   - 
Method in class sim.field.grid. DoubleGrid2D 
 Sets all the locations in the grid the provided element
   setTo(DoubleGrid2D)   - 
Method in class sim.field.grid. DoubleGrid2D 
 Changes the dimensions of the grid to be the same as the one provided, then
        sets all the locations in the grid to the elements at the quivalent locations in the
        provided grid.
   setTo(double)   - 
Method in class sim.field.grid. DoubleGrid3D 
 Sets all the locations in the grid the provided element
   setTo(DoubleGrid3D)   - 
Method in class sim.field.grid. DoubleGrid3D 
 Changes the dimensions of the grid to be the same as the one provided, then
        sets all the locations in the grid to the elements at the quivalent locations in the
        provided grid.
   setTo(int)   - 
Method in class sim.field.grid. IntGrid2D 
 Sets all the locations in the grid the provided element
   setTo(IntGrid2D)   - 
Method in class sim.field.grid. IntGrid2D 
 Changes the dimensions of the grid to be the same as the one provided, then
        sets all the locations in the grid to the elements at the quivalent locations in the
        provided grid.
   setTo(int)   - 
Method in class sim.field.grid. IntGrid3D 
 Sets all the locations in the grid the provided element
   setTo(IntGrid3D)   - 
Method in class sim.field.grid. IntGrid3D 
 Changes the dimensions of the grid to be the same as the one provided, then
        sets all the locations in the grid to the elements at the quivalent locations in the
        provided grid.
   setTo(Object)   - 
Method in class sim.field.grid. ObjectGrid2D 
 Sets all the locations in the grid the provided element
   setTo(ObjectGrid2D)   - 
Method in class sim.field.grid. ObjectGrid2D 
 Changes the dimensions of the grid to be the same as the one provided, then
        sets all the locations in the grid to the elements at the quivalent locations in the
        provided grid.
   setTo(Object)   - 
Method in class sim.field.grid. ObjectGrid3D 
  
   setTo(ObjectGrid3D)   - 
Method in class sim.field.grid. ObjectGrid3D 
  
   setTo(double, double)   - 
Method in class sim.util. MutableDouble2D 
  
   setTo(Int2D)   - 
Method in class sim.util. MutableDouble2D 
  
   setTo(Double2D)   - 
Method in class sim.util. MutableDouble2D 
  
   setTo(MutableInt2D)   - 
Method in class sim.util. MutableDouble2D 
  
   setTo(MutableDouble2D)   - 
Method in class sim.util. MutableDouble2D 
  
   setTo(double, double, double)   - 
Method in class sim.util. MutableDouble3D 
  
   setTo(Int3D)   - 
Method in class sim.util. MutableDouble3D 
  
   setTo(MutableInt3D)   - 
Method in class sim.util. MutableDouble3D 
  
   setTo(Double3D)   - 
Method in class sim.util. MutableDouble3D 
  
   setTo(MutableDouble3D)   - 
Method in class sim.util. MutableDouble3D 
  
   setTo(int, int)   - 
Method in class sim.util. MutableInt2D 
  
   setTo(Point)   - 
Method in class sim.util. MutableInt2D 
  
   setTo(Int2D)   - 
Method in class sim.util. MutableInt2D 
  
   setTo(MutableInt2D)   - 
Method in class sim.util. MutableInt2D 
  
   setTo(int, int, int)   - 
Method in class sim.util. MutableInt3D 
  
   setTo(Int3D)   - 
Method in class sim.util. MutableInt3D 
  
   setTo(MutableInt3D)   - 
Method in class sim.util. MutableInt3D 
  
   setTolerance(double)   - 
Method in class sim.display3d. SelectionBehavior 
  
   setToMinus(MutableDouble2D)   - 
Method in class sim.util. MutableDouble2D 
 Sets the values to the negation of the values in the provided MutableDouble2D
   setToMinus(MutableDouble3D)   - 
Method in class sim.util. MutableDouble3D 
 Sets the values to the negation of the values in the provided MutableDouble3D
   setToolTipText(String)   - 
Method in class sim.util.gui. NumberTextField 
  
   setToolTipText(String)   - 
Method in class sim.util.gui. PropertyField 
  
   setTransform(Transform3D)   - 
Method in class sim.display3d. Display3D 
 Sets the Display3D's global model transform.
   setTransform(Transform3D)   - 
Method in class sim.portrayal3d. FieldPortrayal3D 
 Sets the FieldPortrayal3D's internal Transform.
   setTransform(TransformGroup, Transform3D)   - 
Method in class sim.portrayal3d.simple. PrimitivePortrayal3D 
 Sets the Transform3D of the portrayal, if there is one (in some cases, such as ValuePortrayal3D, there won't be any).
   setTransform(Transform3D)   - 
Method in class sim.portrayal3d.simple. TransformedPortrayal3D 
 Sets the TransformedPortrayal3D's internal Transform.
   setTransform(TransformGroup, Transform3D)   - 
Method in class sim.portrayal3d.simple. ValuePortrayal3D 
 Returns false and does not set the transform (there's nothing to set).
   setTransparency(double)   - 
Method in class sim.portrayal3d.grid. ValueGrid2DPortrayal3D 
 Sets non-image transparency: 1.0 is fully opaque, 0.0 is fully transparent.
   setupApplet(Class)   - 
Method in class sim.display. SimApplet 
  
   setupHints(boolean, boolean, boolean)   - 
Method in class sim.display. Display2D.InnerDisplay2D 
 The default method for setting up the given hints.
   setUsingTriangles(boolean)   - 
Method in class sim.portrayal3d.grid. ValueGrid2DPortrayal3D 
  
   setValue(double)   - 
Method in class sim.portrayal.simple. ValuePortrayal2D.DoubleFilter 
  
   setValue(int)   - 
Method in class sim.portrayal.simple. ValuePortrayal2D.IntFilter 
  
   setValue(double)   - 
Method in class sim.portrayal3d.grid.quad. QuadPortrayal.DoubleFilter 
  
   setValue(int)   - 
Method in class sim.portrayal3d.grid.quad. QuadPortrayal.IntFilter 
  
   setValue(double)   - 
Method in class sim.portrayal3d.simple. ValuePortrayal3D.DoubleFilter 
  
   setValue(int)   - 
Method in class sim.portrayal3d.simple. ValuePortrayal3D.IntFilter 
  
   setValue(int, Object)   - 
Method in class sim.util. Bag 
 identical to set(index, element)
   setValue(int, Object)   - 
Method in class sim.util. DoubleBag 
  
   setValue(double)   - 
Method in class sim.util.gui. NumberTextField 
 Sets the value without filtering first.
   setValue(String)   - 
Method in class sim.util.gui. PropertyField 
 Sets the value, not filtering it through newValue(val) first.
   setValue(int, Object)   - 
Method in interface sim.util. Indexed 
 Throws an IndexOutOfBoundsException if index is inappropriate, and IllegalArgumentException
        if the value is inappropriate.
   setValue(int, Object)   - 
Method in class sim.util. IntBag 
  
   setValue(int, Object)   - 
Method in class sim.util. Properties 
 Sets the current value of the property.
   setValue(int, String)   - 
Method in class sim.util. Properties 
 Sets the current value of the property to the value parsed from the given string.
   setValueName(String)   - 
Method in class sim.portrayal.grid. ValueGridPortrayal2D 
  
   setValueName(String)   - 
Method in class sim.portrayal3d.grid. ValueGrid2DPortrayal3D 
  
   setValueName(String)   - 
Method in class sim.portrayal3d.grid. ValueGridPortrayal3D 
  
   setVolatile(boolean)   - 
Method in class sim.portrayal. Inspector 
 Set to true (default) if the inspector should be updated every time step.
   setVolatile(boolean)   - 
Method in class sim.portrayal. SimpleInspector 
  
   setWhenShouldEnd(long)   - 
Method in class sim.display. Console 
 Set when the simulation should end.
   setWhenShouldEndTime(double)   - 
Method in class sim.display. Console 
 Set when the simulation should end.
   setWhenShouldPause(long)   - 
Method in class sim.display. Console 
 Sets when the simulation should pause.
   setWhenShouldPauseTime(double)   - 
Method in class sim.display. Console 
 Sets when the simulation should pause.
   setX(double)   - 
Method in class sim.util. MutableDouble2D 
  
   setX(double)   - 
Method in class sim.util. MutableDouble3D 
  
   setX(int)   - 
Method in class sim.util. MutableInt2D 
  
   setX(int)   - 
Method in class sim.util. MutableInt3D 
  
   setXAxisLabel(String)   - 
Method in class sim.util.media.chart. ChartGenerator 
 Sets the name of the X Axis label.
   setXAxisLogScaled(boolean)   - 
Method in class sim.util.media.chart. ChartGenerator 
  
   setXAxisRange(double, double)   - 
Method in class sim.util.media.chart. ChartGenerator 
  
   setY(double)   - 
Method in class sim.util. MutableDouble2D 
  
   setY(double)   - 
Method in class sim.util. MutableDouble3D 
  
   setY(int)   - 
Method in class sim.util. MutableInt2D 
  
   setY(int)   - 
Method in class sim.util. MutableInt3D 
  
   setYAxisLabel(String)   - 
Method in class sim.util.media.chart. ChartGenerator 
 Sets the name of the Y Axis label.
   setYAxisLogScaled(boolean)   - 
Method in class sim.util.media.chart. ChartGenerator 
  
   setYAxisRange(double, double)   - 
Method in class sim.util.media.chart. ChartGenerator 
  
   setZ(double)   - 
Method in class sim.util. MutableDouble3D 
  
   setZ(int)   - 
Method in class sim.util. MutableInt3D 
  
   setZScale(double)   - 
Method in class sim.portrayal3d.grid.quad. QuadPortrayal 
  
   shape   - 
Variable in class sim.portrayal.network. SimpleEdgePortrayal2D 
  
   shape   - 
Variable in class sim.portrayal.simple. ShapePortrayal2D 
  
   Shape3DPortrayal3D   - Class in  sim.portrayal3d.simple  A simple portrayal for displaying Shape3D objects.   Shape3DPortrayal3D(Shape3D)   - 
Constructor for class sim.portrayal3d.simple. Shape3DPortrayal3D 
 Constructs a Shape3DPortrayal3D with the given shape and a default (flat opaque white) appearance.
   Shape3DPortrayal3D(Shape3D, Color)   - 
Constructor for class sim.portrayal3d.simple. Shape3DPortrayal3D 
 Constructs a Shape3DPortrayal3D  with the given shape and a flat opaque appearance of the given color.
   Shape3DPortrayal3D(Shape3D, Image)   - 
Constructor for class sim.portrayal3d.simple. Shape3DPortrayal3D 
 Constructs a Shape3DPortrayal3D with the given shape and (opaque) image.
   Shape3DPortrayal3D(Shape3D, Appearance)   - 
Constructor for class sim.portrayal3d.simple. Shape3DPortrayal3D 
 Constructs a Shape3DPortrayal3D with the given shape and appearance.
   Shape3DPortrayal3D(Geometry)   - 
Constructor for class sim.portrayal3d.simple. Shape3DPortrayal3D 
 Constructs a Shape3DPortrayal3D with the given geometry and a default (flat opaque white) appearance.
   Shape3DPortrayal3D(Geometry, Color)   - 
Constructor for class sim.portrayal3d.simple. Shape3DPortrayal3D 
 Constructs a Shape3DPortrayal3D  with the given geometry and a flat opaque appearance of the given color.
   Shape3DPortrayal3D(Geometry, Image)   - 
Constructor for class sim.portrayal3d.simple. Shape3DPortrayal3D 
 Constructs a Shape3DPortrayal3D with the given geometry and (opaque) image.
   Shape3DPortrayal3D(Geometry, Appearance)   - 
Constructor for class sim.portrayal3d.simple. Shape3DPortrayal3D 
 Constructs a Shape3DPortrayal3D with the given geometry and appearance.
   SHAPE_COMPASS   - 
Static variable in class sim.portrayal.simple. OrientedPortrayal2D 
  
   SHAPE_CUBE   - 
Static variable in class sim.portrayal3d.simple. ValuePortrayal3D 
  
   SHAPE_KITE   - 
Static variable in class sim.portrayal.simple. OrientedPortrayal2D 
  
   SHAPE_LINE   - 
Static variable in class sim.portrayal.network. SimpleEdgePortrayal2D 
  
   SHAPE_LINE   - 
Static variable in class sim.portrayal.simple. OrientedPortrayal2D 
  
   SHAPE_SQUARE   - 
Static variable in class sim.portrayal3d.simple. ValuePortrayal3D 
  
   SHAPE_TRIANGLE   - 
Static variable in class sim.portrayal.network. SimpleEdgePortrayal2D 
  
   ShapePortrayal2D   - Class in  sim.portrayal.simple  A simple portrayal for 2D visualization of java.awt.Shapes and java.awt.Polygons.   ShapePortrayal2D(double[], double[])   - 
Constructor for class sim.portrayal.simple. ShapePortrayal2D 
  
   ShapePortrayal2D(double[], double[], Paint)   - 
Constructor for class sim.portrayal.simple. ShapePortrayal2D 
  
   ShapePortrayal2D(double[], double[], double)   - 
Constructor for class sim.portrayal.simple. ShapePortrayal2D 
  
   ShapePortrayal2D(double[], double[], Paint, double)   - 
Constructor for class sim.portrayal.simple. ShapePortrayal2D 
  
   ShapePortrayal2D(double[], double[], boolean)   - 
Constructor for class sim.portrayal.simple. ShapePortrayal2D 
  
   ShapePortrayal2D(double[], double[], Paint, boolean)   - 
Constructor for class sim.portrayal.simple. ShapePortrayal2D 
  
   ShapePortrayal2D(double[], double[], double, boolean)   - 
Constructor for class sim.portrayal.simple. ShapePortrayal2D 
  
   ShapePortrayal2D(double[], double[], Paint, double, boolean)   - 
Constructor for class sim.portrayal.simple. ShapePortrayal2D 
  
   ShapePortrayal2D(Shape)   - 
Constructor for class sim.portrayal.simple. ShapePortrayal2D 
  
   ShapePortrayal2D(Shape, Paint)   - 
Constructor for class sim.portrayal.simple. ShapePortrayal2D 
  
   ShapePortrayal2D(Shape, double)   - 
Constructor for class sim.portrayal.simple. ShapePortrayal2D 
  
   ShapePortrayal2D(Shape, Paint, double)   - 
Constructor for class sim.portrayal.simple. ShapePortrayal2D 
  
   ShapePortrayal2D(Shape, boolean)   - 
Constructor for class sim.portrayal.simple. ShapePortrayal2D 
  
   ShapePortrayal2D(Shape, Paint, boolean)   - 
Constructor for class sim.portrayal.simple. ShapePortrayal2D 
  
   ShapePortrayal2D(Shape, double, boolean)   - 
Constructor for class sim.portrayal.simple. ShapePortrayal2D 
  
   ShapePortrayal2D(Shape, Paint, double, boolean)   - 
Constructor for class sim.portrayal.simple. ShapePortrayal2D 
  
   SharedPortrayal3D   - Class in  sim.portrayal3d.simple      SharedPortrayal3D(SimplePortrayal3D)   - 
Constructor for class sim.portrayal3d.simple. SharedPortrayal3D 
  
   shouldUpdate()   - 
Method in class sim.display. Display2D 
 Returns whether it's time to update.
   shouldUpdate()   - 
Method in class sim.display3d. Display3D 
 Returns whether it's time to update.
   SHOW_CHECKBOX   - 
Static variable in class sim.util.gui. PropertyField 
  
   SHOW_LIST   - 
Static variable in class sim.util.gui. PropertyField 
  
   SHOW_SLIDER   - 
Static variable in class sim.util.gui. PropertyField 
  
   SHOW_TEXTFIELD   - 
Static variable in class sim.util.gui. PropertyField 
  
   SHOW_VIEWBUTTON   - 
Static variable in class sim.util.gui. PropertyField 
  
   showAllFrames()   - 
Method in class sim.display. Console 
 Shows and brings to front all JFrames registered with the Console.
   shrink(int)   - 
Method in class sim.util. Bag 
 Resizes the objs array to max(numObjs, desiredLength), unless that value is greater than or equal to objs.length,
        in which case no resizing is done (this operation only shrinks -- use resize() instead).
   shrink(int)   - 
Method in class sim.util. DoubleBag 
 Resizes the objs array to max(numObjs, desiredLength), unless that value is greater than or equal to objs.length,
        in which case no resizing is done (this operation only shrinks -- use resize() instead).
   shrink(int)   - 
Method in class sim.util. IntBag 
 Resizes the objs array to max(numObjs, desiredLength), unless that value is greater than or equal to objs.length,
        in which case no resizing is done (this operation only shrinks -- use resize() instead).
   shuffle(Random)   - 
Method in class sim.util. Bag 
 Shuffles (randomizes the order of) the Bag
   shuffle(MersenneTwisterFast)   - 
Method in class sim.util. Bag 
 Shuffles (randomizes the order of) the Bag
   shuffle(Random)   - 
Method in class sim.util. DoubleBag 
 Shuffles (randomizes the order of) the DoubleBag
   shuffle(MersenneTwisterFast)   - 
Method in class sim.util. DoubleBag 
 Shuffles (randomizes the order of) the DoubleBag
   shuffle(Random)   - 
Method in class sim.util. IntBag 
 Shuffles (randomizes the order of) the IntBag
   shuffle(MersenneTwisterFast)   - 
Method in class sim.util. IntBag 
 Shuffles (randomizes the order of) the IntBag
   sim.display   - package sim.display     sim.display3d   - package sim.display3d     sim.engine   - package sim.engine     sim.field   - package sim.field     sim.field.continuous   - package sim.field.continuous     sim.field.grid   - package sim.field.grid     sim.field.network   - package sim.field.network     sim.portrayal   - package sim.portrayal     sim.portrayal.continuous   - package sim.portrayal.continuous     sim.portrayal.grid   - package sim.portrayal.grid     sim.portrayal.network   - package sim.portrayal.network     sim.portrayal.simple   - package sim.portrayal.simple     sim.portrayal3d   - package sim.portrayal3d     sim.portrayal3d.continuous   - package sim.portrayal3d.continuous     sim.portrayal3d.grid   - package sim.portrayal3d.grid     sim.portrayal3d.grid.quad   - package sim.portrayal3d.grid.quad     sim.portrayal3d.simple   - package sim.portrayal3d.simple     sim.util   - package sim.util     sim.util.gui   - package sim.util.gui     sim.util.media   - package sim.util.media     sim.util.media.chart   - package sim.util.media.chart     SimApplet   - Class in  sim.display  A simple class for creating Applets out of your simulations.   SimApplet()   - 
Constructor for class sim.display. SimApplet 
  
   SimpleColorMap   - Class in  sim.util.gui  Maps numerical levels to colors using either a lookup table, color interpolation, or both.   SimpleColorMap()   - 
Constructor for class sim.util.gui. SimpleColorMap 
 Constructs a ColorMap that gradiates from 0.0 -> black to 1.0 -> white.
   SimpleColorMap(double, double, Color, Color)   - 
Constructor for class sim.util.gui. SimpleColorMap 
 Constructs a ColorMap that gradiates from minLevel -> minColor to maxLevel -> maxColor.
   SimpleColorMap(Color[])   - 
Constructor for class sim.util.gui. SimpleColorMap 
 Given an array of size n, constructs a ColorMap that maps integers from 0 to n-1 to the colors in the array.
   SimpleColorMap(Color[], double, double, Color, Color)   - 
Constructor for class sim.util.gui. SimpleColorMap 
 Given an array of size n, constructs a ColorMap that maps integers from 0 to n-1 to the colors in the array,
        and gradiates from minLevel -> minColor to maxLevel -> maxColor for certain other values.
   SimpleController   - Class in  sim.display      SimpleController(GUIState)   - 
Constructor for class sim.display. SimpleController 
  
   SimpleController(GUIState, boolean)   - 
Constructor for class sim.display. SimpleController 
  
   SimpleEdgePortrayal2D   - Class in  sim.portrayal.network      SimpleEdgePortrayal2D()   - 
Constructor for class sim.portrayal.network. SimpleEdgePortrayal2D 
 Draws a single-color, undirected black line (or triangle) with no label.
   SimpleEdgePortrayal2D(Paint, Paint)   - 
Constructor for class sim.portrayal.network. SimpleEdgePortrayal2D 
 One single color line will be drawn, and if labelPaint is null, no label is drawn.
   SimpleEdgePortrayal2D(Paint, Paint, Paint)   - 
Constructor for class sim.portrayal.network. SimpleEdgePortrayal2D 
 If fromPaint == toPaint, one single color line will be drawn, and if labelPaint is null, no label is drawn.
   SimpleEdgePortrayal2D(Paint, Paint, Paint, Font)   - 
Constructor for class sim.portrayal.network. SimpleEdgePortrayal2D 
 If fromPaint == toPaint, one single color line will be drawn, and if labelPaint is null, no label is drawn.
   SimpleInspector   - Class in  sim.portrayal  A simple inspector class that looks at the "getX" and "setX" method of the object to be investigates
   and creates a user-friendly graphical interface with read only and read/write components of the object.   SimpleInspector(Properties, GUIState, String, int)   - 
Constructor for class sim.portrayal. SimpleInspector 
  
   SimpleInspector(Properties, GUIState, String)   - 
Constructor for class sim.portrayal. SimpleInspector 
  
   SimpleInspector(Object, GUIState)   - 
Constructor for class sim.portrayal. SimpleInspector 
  
   SimpleInspector(Object, GUIState, String)   - 
Constructor for class sim.portrayal. SimpleInspector 
  
   SimpleInspector(Object, GUIState, String, int)   - 
Constructor for class sim.portrayal. SimpleInspector 
  
   SimplePortrayal2D   - Class in  sim.portrayal  The superclass of all 2D Simple Portrayals.   SimplePortrayal2D()   - 
Constructor for class sim.portrayal. SimplePortrayal2D 
  
   SimplePortrayal3D   - Class in  sim.portrayal3d  The superclass of all 3D Simple Portrayals which by default adds nothing to the 3D
    scene.   SimplePortrayal3D()   - 
Constructor for class sim.portrayal3d. SimplePortrayal3D 
  
   SimpleProperties   - Class in  sim.util  A very simple class for getting and setting object properties.   SimpleProperties(Object)   - 
Constructor for class sim.util. SimpleProperties 
 Gathers all properties for the object, including ones defined in superclasses.
   SimpleProperties(Object, boolean, boolean)   - 
Constructor for class sim.util. SimpleProperties 
  Deprecated.   Use the full form 
   SimpleProperties(Object, boolean, boolean, boolean)   - 
Constructor for class sim.util. SimpleProperties 
 Gathers all properties for the object, possibly including ones defined in superclasses.
   SimState   - Class in  sim.engine  SimState represents the simulation proper.   SimState(long)   - 
Constructor for class sim.engine. SimState 
 Creates a SimState with a new random number generator initialized to the given seed,
        plus a new, empty schedule.
   SimState(MersenneTwisterFast, Schedule)   - 
Constructor for class sim.engine. SimState 
 Creates a SimState with the given random number generator and schedule, and
        sets the seed to a bogus value (0).
   SimState(long, Schedule)   - 
Constructor for class sim.engine. SimState 
 Creates a SimState with the schedule, creating a new random number generator.
   SimState(MersenneTwisterFast)   - 
Constructor for class sim.engine. SimState 
 Creates a SimState with a new schedule, the provided random number generator,
        and a bogus seed (0).
   simulationClass()   - 
Method in interface sim.engine. MakesSimState 
 Returns the class of the SimState subclass that will be generated.
   size()   - 
Method in class sim.field. SparseField 
 Returns the number of elements in the field
   size()   - 
Method in class sim.util. Bag 
  
   size()   - 
Method in class sim.util. DoubleBag 
  
   size()   - 
Method in interface sim.util. Indexed 
  
   size()   - 
Method in class sim.util. IntBag 
  
   skipBox   - 
Variable in class sim.display. Display2D 
 The combo box for skipping frames
   skipBox   - 
Variable in class sim.display3d. Display3D 
 The combo box for skipping frames
   skipField   - 
Variable in class sim.display. Display2D 
 The field for skipping frames
   skipField   - 
Variable in class sim.display3d. Display3D 
 The field for skipping frames
   skipFrame   - 
Variable in class sim.display. Display2D 
 The frame which holds the skip controls
   skipFrame   - 
Variable in class sim.display3d. Display3D 
 The frame which holds the skip controls
   SLOP   - 
Static variable in class sim.portrayal.simple. AdjustablePortrayal2D 
  
   snapshotButton   - 
Variable in class sim.display. Display2D 
 The button which snaps a screenshot
   snapshotButton   - 
Variable in class sim.display3d. Display3D 
 The button which snaps a screenshot
   sort(Comparator)   - 
Method in class sim.util. Bag 
 Sorts the bag according to the provided comparator
   sort()   - 
Method in class sim.util. Bag 
 Sorts the bag under the assumption that all objects stored within are Comparable.
   sort()   - 
Method in class sim.util. DoubleBag 
 Sorts the doubles into ascending numerical order.
   sort()   - 
Method in class sim.util. IntBag 
 Sorts the ints into ascending numerical order.
   SparseField   - Class in  sim.field  While it has no abstract members, SparseField is explicitly an abstract superclass of various sparse
    field objects.   SparseField()   - 
Constructor for class sim.field. SparseField 
  
   SparseField(SparseField)   - 
Constructor for class sim.field. SparseField 
  
   SparseField.LocationAndIndex   - Class in  sim.field  Objects stored in SparseField's locationAndIndexHash table.   SparseField.LocationAndIndex(Object, int)   - 
Constructor for class sim.field. SparseField.LocationAndIndex 
  
   SparseField2D   - Interface in  sim.field      SparseField3D   - Interface in  sim.field      SparseFieldPortrayal3D   - Class in  sim.portrayal3d  An abstract superclass for all FieldPortrayal3Ds which display SparseFields.   SparseFieldPortrayal3D()   - 
Constructor for class sim.portrayal3d. SparseFieldPortrayal3D 
  
   SparseGrid2D   - Class in  sim.field.grid  A storage facility for sparse objects in discrete 2D space, using HashMaps.   SparseGrid2D(int, int)   - 
Constructor for class sim.field.grid. SparseGrid2D 
  
   SparseGrid2D(SparseGrid2D)   - 
Constructor for class sim.field.grid. SparseGrid2D 
  
   SparseGrid2DPortrayal3D   - Class in  sim.portrayal3d.grid  Displays objects in a SparseGrid2D along the XY grid in a special way.   SparseGrid2DPortrayal3D(double)   - 
Constructor for class sim.portrayal3d.grid. SparseGrid2DPortrayal3D 
 Creates a SparseGrid2DPortrayal3D with the provided scale
   SparseGrid2DPortrayal3D()   - 
Constructor for class sim.portrayal3d.grid. SparseGrid2DPortrayal3D 
 Creates a SparseGrid2DPortrayal3D with scale = 1.0
   SparseGrid3D   - Class in  sim.field.grid  A storage facility for sparse objects in discrete 3D space, using HashMaps.   SparseGrid3D(int, int, int)   - 
Constructor for class sim.field.grid. SparseGrid3D 
  
   SparseGrid3D(SparseGrid3D)   - 
Constructor for class sim.field.grid. SparseGrid3D 
  
   SparseGridPortrayal2D   - Class in  sim.portrayal.grid  Can be used to draw both continuous and discrete sparse fields.   SparseGridPortrayal2D()   - 
Constructor for class sim.portrayal.grid. SparseGridPortrayal2D 
  
   SparseGridPortrayal2D(DrawPolicy)   - 
Constructor for class sim.portrayal.grid. SparseGridPortrayal2D 
  Deprecated.   Use setDrawPolicy. 
   SparseGridPortrayal3D   - Class in  sim.portrayal3d.grid  Portrays both SparseGrid2D and SparseGrid3D fields.   SparseGridPortrayal3D()   - 
Constructor for class sim.portrayal3d.grid. SparseGridPortrayal3D 
  
   SpatialNetwork2D   - Class in  sim.portrayal.network  A wrapper used by NetworkPortrayal2D to hold a Network and EITHER a Continuous2D OR a SparseGrid2D (or some other SparseField2D).   SpatialNetwork2D(SparseField2D, Network)   - 
Constructor for class sim.portrayal.network. SpatialNetwork2D 
  
   SpherePortrayal3D   - Class in  sim.portrayal3d.simple  Portrays objects as a sphere of the specified color or appearance (flat opaque white by default)
 which fills the region from (-0.5*scale,-0.5*scale,-0.5*scale) to (0.5*scale,0.5*scale,0.5*scale).   SpherePortrayal3D()   - 
Constructor for class sim.portrayal3d.simple. SpherePortrayal3D 
 Constructs a SpherePortrayal3D with a default (flat opaque white) appearance and a scale of 1.0.
   SpherePortrayal3D(double)   - 
Constructor for class sim.portrayal3d.simple. SpherePortrayal3D 
 Constructs a SpherePortrayal3D with a default (flat opaque white) appearance and the given scale.
   SpherePortrayal3D(Color)   - 
Constructor for class sim.portrayal3d.simple. SpherePortrayal3D 
 Constructs a SpherePortrayal3D with a flat opaque appearance of the given color and a scale of 1.0.
   SpherePortrayal3D(Color, double)   - 
Constructor for class sim.portrayal3d.simple. SpherePortrayal3D 
 Constructs a SpherePortrayal3D with a flat opaque appearance of the given color and the given scale.
   SpherePortrayal3D(Color, double, int)   - 
Constructor for class sim.portrayal3d.simple. SpherePortrayal3D 
 Constructs a SpherePortrayal3D with a flat opaque appearance of the given color, scale, and divisions.
   SpherePortrayal3D(Image)   - 
Constructor for class sim.portrayal3d.simple. SpherePortrayal3D 
 Constructs a SpherePortrayal3D with the given (opaque) image and a scale of 1.0.
   SpherePortrayal3D(Image, double)   - 
Constructor for class sim.portrayal3d.simple. SpherePortrayal3D 
 Constructs a SpherePortrayal3D with the given (opaque) image and scale.
   SpherePortrayal3D(Image, double, int)   - 
Constructor for class sim.portrayal3d.simple. SpherePortrayal3D 
 Constructs a SpherePortrayal3D with the given (opaque) image, scale, and divisions.
   SpherePortrayal3D(Appearance, boolean, boolean, double)   - 
Constructor for class sim.portrayal3d.simple. SpherePortrayal3D 
 Constructs a SpherePortrayal3D with the given appearance, and scale, plus whether or not to generate normals or texture coordinates.
   SpherePortrayal3D(Appearance, boolean, boolean, double, int)   - 
Constructor for class sim.portrayal3d.simple. SpherePortrayal3D 
 Constructs a SpherePortrayal3D with the given appearance, divisions, and scale, plus whether or not to generate normals or texture coordinates.
   split(String)   - 
Static method in class sim.util.gui. WordWrap 
 A useful auxillary method: once you're word-wrapped your text, you can use this to break it into
        multiple strings at the \n position.
   start()   - 
Method in class sim.display. GUIState 
  
   start()   - 
Method in class sim.engine. SimState 
 Called immediately prior to starting the simulation, or in-between
        simulation runs.
   start(BufferedImage)   - 
Method in class sim.util.gui. MovieMaker 
 Create a dialog box allowing the user to specify where to save the file, and in what format and frame rate (default = 10 frames per second), and set up the movie encoding process ready to go, using typicalImage as an example image (for size purposes).
   start(BufferedImage, float)   - 
Method in class sim.util.gui. MovieMaker 
 Create a dialog box allowing the user to specify where to save the file, and in what format and frame rate (default provided), and set up the movie encoding process ready to go, using typicalImage as an example image (for size purposes).
   startMovie()   - 
Method in class sim.display. Display2D 
 Starts a Quicktime movie on the given Display2D.
   startMovie()   - 
Method in class sim.display3d. Display3D 
 Starts a Quicktime movie on the given Display3D.
   startMovie()   - 
Method in class sim.util.media.chart. ChartGenerator 
 Starts a Quicktime movie on the given ChartGenerator.
   state   - 
Variable in class sim.display. GUIState 
 The underlying SimState
   state   - 
Variable in class sim.engine. AsynchronousSteppable 
  
   stateEquals(Object)   - 
Method in class ec.util. MersenneTwisterFast 
  
   step(SimState)   - 
Method in class sim.display. Display2D 
 Steps the Display2D in the GUIState schedule.
   step()   - 
Method in class sim.display. GUIState 
 Returns FALSE if nothing was stepped -- the schedule is exhausted or time has run out.
   step(SimState)   - 
Method in class sim.display. RateAdjuster 
  
   step(SimState)   - 
Method in class sim.display3d. Display3D 
 Steps the Display3D in the GUIState schedule.
   step(SimState)   - 
Method in class sim.engine. AsynchronousSteppable 
 Fires up the AsynchronousSteppable and registers it with the SimState.
   step(SimState)   - 
Method in class sim.engine. MethodStep 
  
   step(SimState)   - 
Method in class sim.engine. MultiStep 
  
   step(SimState)   - 
Method in class sim.engine. ParallelSequence 
  
   step(SimState)   - 
Method in class sim.engine. RandomSequence 
  
   step(SimState)   - 
Method in class sim.engine. Schedule 
 Steps the schedule, gathering and ordering all the items to step on the next time step (skipping
        blank time steps), and then stepping all of them in the decided order.
   step(SimState)   - 
Method in class sim.engine. Sequence 
  
   step(SimState)   - 
Method in interface sim.engine. Steppable 
  
   step   - 
Variable in class sim.engine. TentativeStep 
  
   step(SimState)   - 
Method in class sim.engine. TentativeStep 
  
   step(SimState)   - 
Method in class sim.engine. WeakStep 
  
   Steppable   - Interface in  sim.engine  Something that can be stepped   steps   - 
Variable in class sim.engine. Sequence 
  
   stop()   - 
Method in class sim.engine. AsynchronousSteppable 
 Requests that the AsynchronousSteppable shut down its thread, and blocks until this occurs.
   stop()   - 
Method in interface sim.engine. Stoppable 
  
   stop()   - 
Method in class sim.engine. TentativeStep 
  
   stop()   - 
Method in class sim.util.gui. MovieMaker 
 End the movie stream, finish up writing to disk, and clean up.
   stop()   - 
Method in class sim.util.media. MovieEncoder 
 Stops the writer and finishes uprocessor.
   stopAllInspectors(boolean)   - 
Method in class sim.display. Console 
 Stops all inspectors.
   stopAllInspectors(boolean)   - 
Method in class sim.display. SimpleController 
 Stops all inspectors.
   stopCapturing()   - 
Method in class sim.display3d. CapturingCanvas3D 
  
   stopMovie()   - 
Method in class sim.display. Display2D 
 Stops a Quicktime movie and cleans up, flushing the remaining frames out to disk.
   stopMovie()   - 
Method in class sim.display3d. Display3D 
 Stops a Quicktime movie and cleans up, flushing the remaining frames out to disk.
   stopMovie()   - 
Method in class sim.util.media.chart. ChartGenerator 
 Stops a Quicktime movie and cleans up, flushing the remaining frames out to disk.
   Stoppable   - Interface in  sim.engine  Stoppable objects can be prevented from being stepped any further by calling their stop() method.   stopper()   - 
Method in class sim.engine. AsynchronousSteppable 
  Deprecated.   Will be deleted in the future. 
   storage   - 
Variable in class sim.display. GUIState 
  
   stroke   - 
Variable in class sim.portrayal.simple. ShapePortrayal2D 
  
   stx(double)   - 
Method in class sim.field.continuous. Continuous2D 
 Simple [and fast] toroidal x.
   stx(double)   - 
Method in class sim.field.continuous. Continuous3D 
 Simple [and fast] toroidal x.
   stx(int)   - 
Method in class sim.field.grid. AbstractGrid2D 
  
   stx(int)   - 
Method in class sim.field.grid. AbstractGrid3D 
  
   stx(int)   - 
Method in interface sim.field.grid. Grid2D 
 Simple [and fast] toroidal x.
   stx(int)   - 
Method in interface sim.field.grid. Grid3D 
 Simple [and fast] toroidal x.
   stx(int)   - 
Method in class sim.field.grid. SparseGrid2D 
  
   stx(int)   - 
Method in class sim.field.grid. SparseGrid3D 
  
   sty(double)   - 
Method in class sim.field.continuous. Continuous2D 
 Simple [and fast] toroidal y.
   sty(double)   - 
Method in class sim.field.continuous. Continuous3D 
 Simple [and fast] toroidal y.
   sty(int)   - 
Method in class sim.field.grid. AbstractGrid2D 
  
   sty(int)   - 
Method in class sim.field.grid. AbstractGrid3D 
  
   sty(int)   - 
Method in interface sim.field.grid. Grid2D 
 Simple [and fast] toroidal y.
   sty(int)   - 
Method in interface sim.field.grid. Grid3D 
 Simple [and fast] toroidal y.
   sty(int)   - 
Method in class sim.field.grid. SparseGrid2D 
  
   sty(int)   - 
Method in class sim.field.grid. SparseGrid3D 
  
   stz(double)   - 
Method in class sim.field.continuous. Continuous3D 
 Simple [and fast] toroidal z.
   stz(int)   - 
Method in class sim.field.grid. AbstractGrid3D 
  
   stz(int, int)   - 
Method in class sim.field.grid. AbstractGrid3D 
  
   stz(int)   - 
Method in interface sim.field.grid. Grid3D 
 Simple [and fast] toroidal z.
   stz(int)   - 
Method in class sim.field.grid. SparseGrid3D 
  
   stz(int, int)   - 
Method in class sim.field.grid. SparseGrid3D 
  
   submit()   - 
Method in class sim.util.gui. NumberTextField 
  
   submit()   - 
Method in class sim.util.gui. PropertyField 
 Commits to the current setting of the propertyField, filtering it through newValue.
   subtract(Double2D)   - 
Method in class sim.util. Double2D 
 Subtracts Double2D "other" from current Double2D using 
 vector subtraction
   subtract(MutableDouble2D, MutableDouble2D)   - 
Method in class sim.util. MutableDouble2D 
 Subtracts other2 from other1, setting me to the result and returning me.
   subtract(Double2D, MutableDouble2D)   - 
Method in class sim.util. MutableDouble2D 
 Subtracts other2 from other1, setting me to the result and returning me.
   subtract(MutableDouble2D, Double2D)   - 
Method in class sim.util. MutableDouble2D 
 Subtracts other2 from other1, setting me to the result and returning me.
   subtract(MutableDouble3D, MutableDouble3D)   - 
Method in class sim.util. MutableDouble3D 
 Subtracts other2 from other1, setting me to the result and returning me.
   subtractIn(Double2D)   - 
Method in class sim.util. MutableDouble2D 
 Sets me to me minus other, returning me.
   subtractIn(MutableDouble2D)   - 
Method in class sim.util. MutableDouble2D 
 Sets me to me minus other, returning me.
   subtractIn(Double3D)   - 
Method in class sim.util. MutableDouble3D 
 Sets me to me minus other, returning me.
   subtractIn(MutableDouble3D)   - 
Method in class sim.util. MutableDouble3D 
 Sets me to me minus other, returning me.
 
 
    
 T  
 
   takeSnapshot(File, int)   - 
Method in class sim.display. Display2D 
  
   takeSnapshot()   - 
Method in class sim.display. Display2D 
  
   takeSnapshot(File)   - 
Method in class sim.display3d. Display3D 
 Takes a snapshot of the Display3D's currently displayed simulation.
   takeSnapshot()   - 
Method in class sim.display3d. Display3D 
 Takes a snapshot of the Display3D's currently displayed simulation.
   tds(Double2D, Double2D)   - 
Method in class sim.field.continuous. Continuous2D 
 Minimum Toroidal Distance Squared between two points.
   tds(Double3D, Double3D)   - 
Method in class sim.field.continuous. Continuous3D 
 Minimum Toroidal Distance Squared between two points.
   tdx(double, double)   - 
Method in class sim.field.continuous. Continuous2D 
 Minimum toroidal distance between two values in the X dimension.
   tdx(double, double)   - 
Method in class sim.field.continuous. Continuous3D 
 Minimum toroidal distance between two values in the X dimension.
   tdy(double, double)   - 
Method in class sim.field.continuous. Continuous2D 
 Minimum toroidal distance between two values in the Y dimension.
   tdy(double, double)   - 
Method in class sim.field.continuous. Continuous3D 
 Minimum toroidal distance between two values in the Y dimension.
   tdz(double, double)   - 
Method in class sim.field.continuous. Continuous3D 
 Minimum toroidal distance between two values in the Z dimension.
   TentativeStep   - Class in  sim.engine  A Steppable wrapper which can be stopped.   TentativeStep(Steppable)   - 
Constructor for class sim.engine. TentativeStep 
  
   throwIndexOutOfBoundsException(int)   - 
Method in class sim.util. Bag 
  
   throwIndexOutOfBoundsException(int)   - 
Method in class sim.util. DoubleBag 
  
   throwIndexOutOfBoundsException(int)   - 
Method in class sim.util. IntBag 
  
   TilePortrayal   - Class in  sim.portrayal3d.grid.quad  A QuadPortrayal which describes locations as the center of a square in a grid (like tiles on the floor,
 each tile corresponding to a location on the grid).   TilePortrayal(ColorMap)   - 
Constructor for class sim.portrayal3d.grid.quad. TilePortrayal 
  
   TilePortrayal(ColorMap, double)   - 
Constructor for class sim.portrayal3d.grid.quad. TilePortrayal 
  
   time()   - 
Method in class sim.engine. Schedule 
  Deprecated.   use getTime() 
   TimeSeriesAttributes   - Class in  sim.util.media.chart  A SeriesAttributes used for user control pf time series created with TimeSeriesCharGenerator.   TimeSeriesAttributes(ChartGenerator, XYSeries, int, SeriesChangeListener)   - 
Constructor for class sim.util.media.chart. TimeSeriesAttributes 
 Builds a TimeSeriesAttributes with the given generator, series, and index for the series.
   TimeSeriesChartGenerator   - Class in  sim.util.media.chart  TimeSeriesChartGenerator is a ChartGenerator which displays a time-series chart using the JFreeChart library.   TimeSeriesChartGenerator()   - 
Constructor for class sim.util.media.chart. TimeSeriesChartGenerator 
  
   tmpLocalT   - 
Variable in class sim.portrayal3d.grid. ValueGrid2DPortrayal3D 
 tmp Transform3D 
 it is reused, since the TGs are copying it internally
   tmpVect   - 
Variable in class sim.portrayal3d.grid. ValueGrid2DPortrayal3D 
 tmp Vector3d
   to()   - 
Method in class sim.field.network. Edge 
 Returns the "to" object.
   toArray()   - 
Method in class sim.field.grid. DoubleGrid2D 
 Flattens the grid to a one-dimensional array, storing the elements in row-major order,including duplicates and null values.
   toArray()   - 
Method in class sim.field.grid. DoubleGrid3D 
 Flattens the grid to a one-dimensional array, storing the elements in row-major order,including duplicates and null values.
   toArray()   - 
Method in class sim.field.grid. IntGrid2D 
 Flattens the grid to a one-dimensional array, storing the elements in row-major order,including duplicates and null values.
   toArray()   - 
Method in class sim.field.grid. IntGrid3D 
 Flattens the grid to a one-dimensional array, storing the elements in row-major order,including duplicates and null values.
   toArray()   - 
Method in class sim.field.grid. ObjectGrid2D 
 Flattens the grid to a one-dimensional array, storing the elements in row-major order,including duplicates and null values.
   toArray()   - 
Method in class sim.field.grid. ObjectGrid3D 
 Flattens the grid to a one-dimensional array, storing the elements in row-major order,including duplicates and null values.
   toArray()   - 
Method in class sim.util. Bag 
  
   toArray(Object[])   - 
Method in class sim.util. Bag 
  
   toArray()   - 
Method in class sim.util. DoubleBag 
  
   toArray()   - 
Method in class sim.util. IntBag 
  
   toCoordinates()   - 
Method in class sim.util. Double2D 
  
   toCoordinates()   - 
Method in class sim.util. Double3D 
  
   toCoordinates()   - 
Method in class sim.util. Int2D 
  
   toCoordinates()   - 
Method in class sim.util. Int3D 
  
   toCoordinates()   - 
Method in class sim.util. MutableDouble2D 
  
   toCoordinates()   - 
Method in class sim.util. MutableDouble3D 
  
   toCoordinates()   - 
Method in class sim.util. MutableInt2D 
  
   toCoordinates()   - 
Method in class sim.util. MutableInt3D 
  
   toDouble()   - 
Method in class sim.util. MutableDouble 
  
   toDoubleArray()   - 
Method in class sim.util. DoubleBag 
  
   toDoubleArray()   - 
Method in class sim.util. IntBag 
  
   toHTML(String)   - 
Static method in class sim.util.gui. WordWrap 
 A useful auxillary method: once you've word-wrapped your text, you can use this to convert it into
        'HTML' style, where  <  is converted into  &lt; ,  &  is converted into
         &amp; , and  \n  or  \r  are converted into  <br> .
   toIntegerArray()   - 
Method in class sim.util. IntBag 
  
   toLongArray()   - 
Method in class sim.util. IntBag 
  
   ToolTipBehavior   - Class in  sim.display3d  A behavior similar to SelectionBehavior, except you don't have to
 double-click and the resulting info is presented in a tool-tip, not the
 console
 
 I'm using Swing's default (ToolTipManager) initialDelay constant, but I did
 not implement DismissDelay or ReshowDelay   ToolTipBehavior(Canvas3D, BranchGroup, Bounds, GUIState)   - 
Constructor for class sim.display3d. ToolTipBehavior 
  
   tooManyPoints(int)   - 
Method in interface sim.util.media.chart. DataCuller 
  
   tooManyPoints(int)   - 
Method in class sim.util.media.chart. MinGapDataCuller 
  
   top()   - 
Method in class sim.util. Bag 
 Returns null if the Bag is empty, else returns the topmost object.
   top()   - 
Method in class sim.util. DoubleBag 
 Returns 0 if the DoubleBag is empty, else returns the topmost double.
   top()   - 
Method in class sim.util. IntBag 
 Returns 0 if the IntBag is empty, else returns the topmost int.
   toPaint   - 
Variable in class sim.portrayal.network. SimpleEdgePortrayal2D 
  
   toPoint()   - 
Method in class sim.util. Int2D 
  
   toPoint()   - 
Method in class sim.util. MutableInt2D 
  
   toPoint2D()   - 
Method in class sim.util. Double2D 
  
   toPoint2D()   - 
Method in class sim.util. Int2D 
  
   toPoint2D()   - 
Method in class sim.util. MutableDouble2D 
  
   toPoint2D()   - 
Method in class sim.util. MutableInt2D 
  
   toString()   - 
Method in class sim.field.network. Edge 
  
   toString()   - 
Method in class sim.portrayal. DrawInfo2D 
  
   toString()   - 
Method in class sim.portrayal.network. EdgeDrawInfo2D 
  
   toString()   - 
Method in class sim.portrayal.simple. ValuePortrayal2D.Filter 
  
   toString()   - 
Method in class sim.portrayal3d.simple. ValuePortrayal3D.Filter 
  
   toString()   - 
Method in class sim.util. Double2D 
  
   toString()   - 
Method in class sim.util. Double3D 
  
   toString()   - 
Method in class sim.util. Int2D 
  
   toString()   - 
Method in class sim.util. Int3D 
  
   toString()   - 
Method in class sim.util. MutableDouble 
  
   toString()   - 
Method in class sim.util. MutableDouble2D 
  
   toString()   - 
Method in class sim.util. MutableDouble3D 
  
   toString()   - 
Method in class sim.util. MutableInt2D 
  
   toString()   - 
Method in class sim.util. MutableInt3D 
  
   trail   - 
Variable in class sim.portrayal.simple. TrailedPortrayal2D 
 The SimplePortrayal2D used to draw line segments in the trail.
   TrailedPortrayal2D   - Class in  sim.portrayal.simple  TrailedPortrayal2D is a special SimplePortrayal wrapper which enables you to draw "trails" or
   "mouse tails" that drag behind objects and show where the've recently been.   TrailedPortrayal2D(GUIState, SimplePortrayal2D, FieldPortrayal2D, SimplePortrayal2D, double)   - 
Constructor for class sim.portrayal.simple. TrailedPortrayal2D 
 Creates a TrailedPortrayal2D for a given child portrayal, field portrayal for the trail, trail portrayal, and length in time.
   TrailedPortrayal2D(GUIState, SimplePortrayal2D, FieldPortrayal2D, double, Color, Color)   - 
Constructor for class sim.portrayal.simple. TrailedPortrayal2D 
 Creates a TrailedPortrayal2D for a given child portrayal, field portrayal for the trail, length in time, using a default trail portrayal going from minColor to maxColor through time.
   TrailedPortrayal2D(GUIState, SimplePortrayal2D, FieldPortrayal2D, double)   - 
Constructor for class sim.portrayal.simple. TrailedPortrayal2D 
 Creates a TrailedPortrayal2D for a given child portrayal, field portrayal for the trail, length in time, using a default trail portrayal with default settings.
   TrailedPortrayal2D.TrailDrawInfo2D   - Class in  sim.portrayal.simple  A special version of DrawInfo2D which adds additional information useful for drawing your own trails.   TrailedPortrayal2D.TrailDrawInfo2D(GUIState, FieldPortrayal2D, RectangularShape, RectangularShape, Point2D.Double)   - 
Constructor for class sim.portrayal.simple. TrailedPortrayal2D.TrailDrawInfo2D 
  
   TrailedPortrayal2D.TrailDrawInfo2D(DrawInfo2D, double, double, Point2D.Double)   - 
Constructor for class sim.portrayal.simple. TrailedPortrayal2D.TrailDrawInfo2D 
  
   TrailedPortrayal2D.TrailDrawInfo2D(DrawInfo2D, Point2D.Double)   - 
Constructor for class sim.portrayal.simple. TrailedPortrayal2D.TrailDrawInfo2D 
  
   TrailedPortrayal2D.TrailDrawInfo2D(EdgeDrawInfo2D)   - 
Constructor for class sim.portrayal.simple. TrailedPortrayal2D.TrailDrawInfo2D 
  
   transform(Transform3D)   - 
Method in class sim.display3d. Display3D 
 Changes the global model transform of the FieldPortrayal3D by 
        appending to it the provided transform operation.
   transform   - 
Variable in class sim.portrayal.simple. TransformedPortrayal2D 
  
   transform(Transform3D)   - 
Method in class sim.portrayal3d. FieldPortrayal3D 
 Changes the internal transform of the FieldPortrayal3D by 
        appending to it the provided transform operation.
   transform(Transform3D)   - 
Method in class sim.portrayal3d.simple. TransformedPortrayal3D 
 Changes the internal transform of the TransformedPortrayal3D by 
        appending to it the provided transform operation.
   TransformedPortrayal2D   - Class in  sim.portrayal.simple  A wrapper for other Portrayal2Ds which transforms the graphics space before drawing them.   TransformedPortrayal2D(SimplePortrayal2D, AffineTransform)   - 
Constructor for class sim.portrayal.simple. TransformedPortrayal2D 
  
   TransformedPortrayal3D   - Class in  sim.portrayal3d.simple  A wrapper for other Portrayal3Ds which transforms them with an underlying Transform3D: meaning
   that you can rotate them, translate them, scale them, etc.   TransformedPortrayal3D(SimplePortrayal3D, Transform3D)   - 
Constructor for class sim.portrayal3d.simple. TransformedPortrayal3D 
  
   TransformedPortrayal3D(SimplePortrayal3D)   - 
Constructor for class sim.portrayal3d.simple. TransformedPortrayal3D 
  
   translate(double, double, double)   - 
Method in class sim.display3d. Display3D 
 Modifies the global model transform by translating in the provided x, y, and z amounts.
   translate(double, double, double)   - 
Method in class sim.portrayal3d. FieldPortrayal3D 
 Modifies the internal transform by translating in the provided x, y, and z amounts.
   translate(double, double, double)   - 
Method in class sim.portrayal3d.simple. TransformedPortrayal3D 
 Modifies the internal transform by translating in the provided x, y, and z amounts.
   trb(int, int)   - 
Method in class sim.field.grid. AbstractGrid2D 
  
   trb(int, int)   - 
Method in interface sim.field.grid. Grid2D 
 Horizontal edge is on the bottom for triangle.
   trb(int, int)   - 
Method in class sim.field.grid. SparseGrid2D 
  
   trt(int, int)   - 
Method in class sim.field.grid. AbstractGrid2D 
  
   trt(int, int)   - 
Method in interface sim.field.grid. Grid2D 
 Horizontal edge is on the top for triangle.
   trt(int, int)   - 
Method in class sim.field.grid. SparseGrid2D 
  
   truncate()   - 
Method in class sim.field.grid. DoubleGrid2D 
 Eliminates the decimal portion of each value in the grid (rounds towards zero).
   truncate()   - 
Method in class sim.field.grid. DoubleGrid3D 
 Eliminates the decimal portion of each value in the grid (rounds towards zero).
   tv(Double2D, Double2D)   - 
Method in class sim.field.continuous. Continuous2D 
 Minimum Toroidal difference vector between two points.
   tv(Double3D, Double3D)   - 
Method in class sim.field.continuous. Continuous3D 
 Minimum Toroidal difference vector between two points.
   tx(double)   - 
Method in class sim.field.continuous. Continuous2D 
 Toroidal x
   tx(double)   - 
Method in class sim.field.continuous. Continuous3D 
 Toroidal x
   tx(int)   - 
Method in class sim.field.grid. AbstractGrid2D 
  
   tx(int)   - 
Method in class sim.field.grid. AbstractGrid3D 
  
   tx(int)   - 
Method in interface sim.field.grid. Grid2D 
 Toroidal x.
   tx(int)   - 
Method in interface sim.field.grid. Grid3D 
 Toroidal x.
   tx(int)   - 
Method in class sim.field.grid. SparseGrid2D 
  
   tx(int)   - 
Method in class sim.field.grid. SparseGrid3D 
  
   ty(double)   - 
Method in class sim.field.continuous. Continuous2D 
 Toroidal y
   ty(double)   - 
Method in class sim.field.continuous. Continuous3D 
 Toroidal y
   ty(int)   - 
Method in class sim.field.grid. AbstractGrid2D 
  
   ty(int)   - 
Method in class sim.field.grid. AbstractGrid3D 
  
   ty(int)   - 
Method in interface sim.field.grid. Grid2D 
 Toroidal y.
   ty(int)   - 
Method in interface sim.field.grid. Grid3D 
 Toroidal y.
   ty(int)   - 
Method in class sim.field.grid. SparseGrid2D 
  
   ty(int)   - 
Method in class sim.field.grid. SparseGrid3D 
  
   TYPE_HIT_OBJECT   - 
Static variable in class sim.portrayal. SimplePortrayal2D 
  
   TYPE_PDF   - 
Static variable in class sim.display. Display2D 
  
   TYPE_PNG   - 
Static variable in class sim.display. Display2D 
  
   TYPE_SELECTED_OBJECT   - 
Static variable in class sim.portrayal. SimplePortrayal2D 
  
   typeToName(Class)   - 
Method in class sim.util. Properties 
  
   tz(double)   - 
Method in class sim.field.continuous. Continuous3D 
 Toroidal z
   tz(int)   - 
Method in class sim.field.grid. AbstractGrid3D 
  
   tz(int)   - 
Method in interface sim.field.grid. Grid3D 
 Toroidal z.
   tz(int)   - 
Method in class sim.field.grid. SparseGrid3D 
  
 
 
    
 U  
 
   ulx(int, int)   - 
Method in class sim.field.grid. AbstractGrid2D 
  
   ulx(int, int)   - 
Method in interface sim.field.grid. Grid2D 
 Hex upleft x.
   ulx(int, int)   - 
Method in class sim.field.grid. SparseGrid2D 
  
   uly(int, int)   - 
Method in class sim.field.grid. AbstractGrid2D 
  
   uly(int, int)   - 
Method in interface sim.field.grid. Grid2D 
 Hex upleft y.
   uly(int, int)   - 
Method in class sim.field.grid. SparseGrid2D 
  
   unbufferedHints   - 
Variable in class sim.display. Display2D.InnerDisplay2D 
 Hints used to draw objects to the screen or to a buffer
   universe   - 
Variable in class sim.display3d. Display3D 
 The Java3D universe.
   unregisterAllFrames()   - 
Method in class sim.display. Console 
 Simulations can call this to clear out the "Display list" of the console
   unregisterAllFrames()   - 
Method in interface sim.display. Controller 
 Simulations can call this to clear out the "Display list" of the Controller.
   unregisterAllFrames()   - 
Method in class sim.display. SimpleController 
 Simulations can call this to clear out the "Display list" of the console
   unregisterFrame(JFrame)   - 
Method in class sim.display. Console 
 Simulations can call this to remove a frame from the "Display list" of the console
   unregisterFrame(JFrame)   - 
Method in interface sim.display. Controller 
 Simulations can call this to remove a frame from the "Display list" of the Controller.
   unregisterFrame(JFrame)   - 
Method in class sim.display. SimpleController 
 Simulations can call this to remove a frame from the "Display list" of the console
   update()   - 
Method in class sim.util.gui. NumberTextField 
  
   update()   - 
Method in class sim.util.gui. PropertyField 
 Reverts the property field to its previous string value WITHOUT calling newValue()
   update(long, boolean)   - 
Method in class sim.util.media.chart. ChartGenerator 
 Key must be 0 or higher.
   update()   - 
Method in class sim.util.media.chart. ChartGenerator 
 Override this to update the chart to reflect new data.
   update()   - 
Method in class sim.util.media.chart. HistogramGenerator 
  
   update()   - 
Method in class sim.util.media.chart. ScatterPlotGenerator 
  
   UPDATE_ICON   - 
Static variable in class sim.portrayal. Inspector 
  
   UPDATE_ICON_P   - 
Static variable in class sim.portrayal. Inspector 
  
   UPDATE_RULE_ALWAYS   - 
Static variable in class sim.display. Display2D 
  
   UPDATE_RULE_INTERNAL_TIME   - 
Static variable in class sim.display. Display2D 
  
   UPDATE_RULE_NEVER   - 
Static variable in class sim.display. Display2D 
  
   UPDATE_RULE_STEPS   - 
Static variable in class sim.display. Display2D 
 Used internally and by Display3D to indicate
   UPDATE_RULE_WALLCLOCK_TIME   - 
Static variable in class sim.display. Display2D 
  
   updateButtonPressed()   - 
Method in class sim.portrayal. Inspector 
  
   updateChartLater(long)   - 
Method in class sim.util.media.chart. ChartGenerator 
 Posts a request to update the chart on the Swing event queue to happen next time repaints etc.
   updateChartWithin(long, long)   - 
Method in class sim.util.media.chart. ChartGenerator 
 Updates the inspector asynchronously sometime before the given milliseconds have transpired.
   updateEdge(Edge, Object, Object, Object)   - 
Method in class sim.field.network. Network 
 Removes the given edge, then changes its from, to, and info values to the provided ones,
        then adds the edge to the network again.
   updateInspector()   - 
Method in class sim.portrayal. Inspector 
 Called by the system to inform the Inspector that it needs to update itself to reflect any
        changed in the underlying data.
   updateInspector()   - 
Method in class sim.portrayal. SimpleInspector 
  
   updateModel(TransformGroup)   - 
Method in class sim.portrayal3d. FieldPortrayal3D 
 Returns a tree structure of the form
        InternalTransformGroup[ model info ].
   updateModel(TransformGroup)   - 
Method in class sim.portrayal3d.grid. ObjectGridPortrayal3D 
  
   updateModel(TransformGroup)   - 
Method in class sim.portrayal3d.grid. SparseGrid2DPortrayal3D 
  
   updateModel(TransformGroup)   - 
Method in class sim.portrayal3d.grid. ValueGrid2DPortrayal3D 
  
   updateModel(TransformGroup)   - 
Method in class sim.portrayal3d.grid. ValueGridPortrayal3D 
  
   updateModel(TransformGroup)   - 
Method in class sim.portrayal3d. SparseFieldPortrayal3D 
  
   updateScene(int, int)   - 
Method in class sim.display3d. ToolTipBehavior 
  
   updateSceneGraph(boolean)   - 
Method in class sim.display3d. Display3D 
 Updates the scene graph to reflect changes in the simulation.
   updateSeries(int, double[])   - 
Method in class sim.util.media.chart. HistogramGenerator 
  
   updateSeries(int, double[][])   - 
Method in class sim.util.media.chart. ScatterPlotGenerator 
  
   updateToolTips()   - 
Method in class sim.display. Display2D.InnerDisplay2D 
  
   UPPER_PAINT   - 
Static variable in class sim.portrayal.simple. AdjustablePortrayal2D 
  
   UPPER_STROKE   - 
Static variable in class sim.portrayal.simple. AdjustablePortrayal2D 
  
   upperBound(double)   - 
Method in class sim.field.grid. DoubleGrid2D 
 Thresholds the grid so that values greater to  toNoMoreThanThisMuch  are changed to  toNoMoreThanThisMuch .
   upperBound(double)   - 
Method in class sim.field.grid. DoubleGrid3D 
 Thresholds the grid so that values greater to  toNoMoreThanThisMuch  are changed to  toNoMoreThanThisMuch .
   upperBound(int)   - 
Method in class sim.field.grid. IntGrid2D 
 Thresholds the grid so that values greater to  toNoMoreThanThisMuch  are changed to  toNoMoreThanThisMuch .
   upperBound(int)   - 
Method in class sim.field.grid. IntGrid3D 
 Thresholds the grid so that values greater to  toNoMoreThanThisMuch  are changed to  toNoMoreThanThisMuch .
   upx(int, int)   - 
Method in class sim.field.grid. AbstractGrid2D 
  
   upx(int, int)   - 
Method in interface sim.field.grid. Grid2D 
 Hex up x.
   upx(int, int)   - 
Method in class sim.field.grid. SparseGrid2D 
  
   upy(int, int)   - 
Method in class sim.field.grid. AbstractGrid2D 
  
   upy(int, int)   - 
Method in interface sim.field.grid. Grid2D 
 Hex up y.
   upy(int, int)   - 
Method in class sim.field.grid. SparseGrid2D 
  
   urx(int, int)   - 
Method in class sim.field.grid. AbstractGrid2D 
  
   urx(int, int)   - 
Method in interface sim.field.grid. Grid2D 
 Hex upright x.
   urx(int, int)   - 
Method in class sim.field.grid. SparseGrid2D 
  
   ury(int, int)   - 
Method in class sim.field.grid. AbstractGrid2D 
  
   ury(int, int)   - 
Method in interface sim.field.grid. Grid2D 
 Hex upright y.
   ury(int, int)   - 
Method in class sim.field.grid. SparseGrid2D 
  
   USE_BUFFER   - 
Static variable in class sim.portrayal. FieldPortrayal2D 
 Use a buffer
   Utilities   - Class in  sim.util.gui  Various static utility methods.   Utilities()   - 
Constructor for class sim.util.gui. Utilities 
  
 
 
    
 V  
 
   val   - 
Variable in class sim.util. MutableDouble 
  
   validLevel(double)   - 
Method in interface sim.util.gui. ColorMap 
 Returns true if a level is "valid" (it provides a meaningful color)
   validLevel(double)   - 
Method in class sim.util.gui. SimpleColorMap 
  
   validSimState(SimState)   - 
Method in class sim.display. GUIState 
 This method should be set to return TRUE if state can be validly used -- mostly likely
        all you need to check is that it's the right class for this simulation.
   Valuable   - Interface in  sim.util  Having a value.   value   - 
Variable in class sim.portrayal.simple. TrailedPortrayal2D.TrailDrawInfo2D 
 A value from 1.0 to 0.0 indicating how far "back in time" this segment is supposed to be.
   value()   - 
Method in class sim.portrayal3d.grid.quad. ValueGridCellInfo 
  
   ValueGrid2DPortrayal3D   - Class in  sim.portrayal3d.grid  Displays ValueGrid2Ds values along the XY grid using a surface.   ValueGrid2DPortrayal3D(String, Image)   - 
Constructor for class sim.portrayal3d.grid. ValueGrid2DPortrayal3D 
 Use a fully opaque image as the appearance.
   ValueGrid2DPortrayal3D(String, double)   - 
Constructor for class sim.portrayal3d.grid. ValueGrid2DPortrayal3D 
 Be somewhat transparent (1.0 is fully opaque, 0.0f is fully transparent).
   ValueGrid2DPortrayal3D(String)   - 
Constructor for class sim.portrayal3d.grid. ValueGrid2DPortrayal3D 
 Be completely opaque.
   ValueGrid2DPortrayal3D()   - 
Constructor for class sim.portrayal3d.grid. ValueGrid2DPortrayal3D 
 Be completely opaque, with a value name of "Value".
   ValueGridCellInfo   - Class in  sim.portrayal3d.grid.quad  Used by ValueGrid2DPortrayal3D to send needed value information to
 underlying QuadPortrayals.   ValueGridCellInfo(ValueGrid2DPortrayal3D, Grid2D)   - 
Constructor for class sim.portrayal3d.grid.quad. ValueGridCellInfo 
  
   ValueGridPortrayal2D   - Class in  sim.portrayal.grid  This class is capable of portraying the DoubleGrid2D and IntGrid2D fields (and  only  those two fields -- or subclasses).   ValueGridPortrayal2D()   - 
Constructor for class sim.portrayal.grid. ValueGridPortrayal2D 
  
   ValueGridPortrayal2D(String)   - 
Constructor for class sim.portrayal.grid. ValueGridPortrayal2D 
  
   ValueGridPortrayal3D   - Class in  sim.portrayal3d.grid      ValueGridPortrayal3D()   - 
Constructor for class sim.portrayal3d.grid. ValueGridPortrayal3D 
  
   ValueGridPortrayal3D(String)   - 
Constructor for class sim.portrayal3d.grid. ValueGridPortrayal3D 
  
   ValueGridPortrayal3D(double)   - 
Constructor for class sim.portrayal3d.grid. ValueGridPortrayal3D 
  
   ValueGridPortrayal3D(String, double)   - 
Constructor for class sim.portrayal3d.grid. ValueGridPortrayal3D 
  
   ValuePortrayal2D   - Class in  sim.portrayal.simple  The ValuePortrayal2D is the default portrayal for ValueGridPortrayal2Ds.   ValuePortrayal2D()   - 
Constructor for class sim.portrayal.simple. ValuePortrayal2D 
  
   ValuePortrayal2D(ValueGridPortrayal2D)   - 
Constructor for class sim.portrayal.simple. ValuePortrayal2D 
  Deprecated.     
   ValuePortrayal2D.DoubleFilter   - Class in  sim.portrayal.simple      ValuePortrayal2D.DoubleFilter(LocationWrapper)   - 
Constructor for class sim.portrayal.simple. ValuePortrayal2D.DoubleFilter 
  
   ValuePortrayal2D.Filter   - Class in  sim.portrayal.simple      ValuePortrayal2D.Filter(LocationWrapper)   - 
Constructor for class sim.portrayal.simple. ValuePortrayal2D.Filter 
  
   ValuePortrayal2D.IntFilter   - Class in  sim.portrayal.simple      ValuePortrayal2D.IntFilter(LocationWrapper)   - 
Constructor for class sim.portrayal.simple. ValuePortrayal2D.IntFilter 
  
   ValuePortrayal3D   - Class in  sim.portrayal3d.simple  ValuePortrayal3D defines a cube or square whose color and transparency can be changed, 
    and is really intended solely for use in ValueGridPortrayal3D.   ValuePortrayal3D()   - 
Constructor for class sim.portrayal3d.simple. ValuePortrayal3D 
 Creates a ValuePortrayal3D with a cube shape.
   ValuePortrayal3D(int)   - 
Constructor for class sim.portrayal3d.simple. ValuePortrayal3D 
 Creates a ValuePortrayal3D with a cube (SHAPE_CUBE) or square (SHAPE_SQUARE) shape.
   ValuePortrayal3D.DoubleFilter   - Class in  sim.portrayal3d.simple      ValuePortrayal3D.DoubleFilter(LocationWrapper)   - 
Constructor for class sim.portrayal3d.simple. ValuePortrayal3D.DoubleFilter 
  
   ValuePortrayal3D.Filter   - Class in  sim.portrayal3d.simple      ValuePortrayal3D.Filter(LocationWrapper)   - 
Constructor for class sim.portrayal3d.simple. ValuePortrayal3D.Filter 
  
   ValuePortrayal3D.IntFilter   - Class in  sim.portrayal3d.simple      ValuePortrayal3D.IntFilter(LocationWrapper)   - 
Constructor for class sim.portrayal3d.simple. ValuePortrayal3D.IntFilter 
  
   valueToPass   - 
Variable in class sim.portrayal.grid. ValueGridPortrayal2D 
  
   version()   - 
Static method in class sim.engine. SimState 
 Returns MASON's Version
   viewProperty()   - 
Method in class sim.util.gui. PropertyField 
 Override this to be informed when a property is to be viewed in its
        own inspector because the user pressed the "view" button.
   viewRoot   - 
Variable in class sim.display3d. Display3D 
 An additional root scene graph node which is attached to the viewing transform of the universe, and thus
        stays in the same location regardless of the placement of the camera.
 
 
    
 W  
 
   WeakStep   - Class in  sim.engine  WeakStep is a wrapper for steppable objects in the situation where we only want
   the schedule to tenuously hold onto the steppable object -- that is, if everyone
   else has forgotten about the object, the schedule should as well.   WeakStep(Steppable)   - 
Constructor for class sim.engine. WeakStep 
  
   width   - 
Variable in class sim.display. Display2D.InnerDisplay2D 
 The width of the display when the scale is 1.0
   width   - 
Variable in class sim.field.continuous. Continuous2D 
  
   width   - 
Variable in class sim.field.continuous. Continuous3D 
  
   width   - 
Variable in class sim.field.grid. AbstractGrid2D 
  
   width   - 
Variable in class sim.field.grid. AbstractGrid3D 
  
   width   - 
Variable in class sim.field.grid. SparseGrid2D 
  
   width   - 
Variable in class sim.field.grid. SparseGrid3D 
  
   WireFrameBoxPortrayal3D   - Class in  sim.portrayal3d.simple  A SimplePortrayal3D which draws an arbitrary wireframe box.   WireFrameBoxPortrayal3D()   - 
Constructor for class sim.portrayal3d.simple. WireFrameBoxPortrayal3D 
 Draws a white wireframe box from (-0.5,-0.5,-0.5) to (0.5,0.5,0.5)
   WireFrameBoxPortrayal3D(double, double, double, double, double, double)   - 
Constructor for class sim.portrayal3d.simple. WireFrameBoxPortrayal3D 
 Draws a white wireframe box from (x,y,z) to (x2,y2,z2)
   WireFrameBoxPortrayal3D(double, double, double, double, double, double, Color)   - 
Constructor for class sim.portrayal3d.simple. WireFrameBoxPortrayal3D 
 Draws a wireframe box from (x,y,z) to (x2,y2,z2) in the specified color.
   WireFrameBoxPortrayal3D(double, double, double, double, double, double, Appearance)   - 
Constructor for class sim.portrayal3d.simple. WireFrameBoxPortrayal3D 
 Draws a wireframe box from (x,y,z) to (x2,y2,z2) in the specified appearance.
   WordWrap   - Class in  sim.util.gui  WordWrap is a simple word-wrapping class which provides word-wrap either to columns of raw text; or to some number
    of pixels (given a font).   WordWrap()   - 
Constructor for class sim.util.gui. WordWrap 
  
   wrap(String, int)   - 
Static method in class sim.util.gui. WordWrap 
 Wraps a string to a given number of columns.
   wrap(String, int, FontMetrics)   - 
Static method in class sim.util.gui. WordWrap 
 Wraps a string to a given number of pixels in width, given a font whose metrics are provided as well.
   wrapModelForNewObject(Object, Transform3D)   - 
Method in class sim.portrayal3d. SparseFieldPortrayal3D 
 This function is called from createModel for each object in the 
 field and from the updateModel part of getModel for the
 new objects.
   writeState(DataOutputStream)   - 
Method in class ec.util. MersenneTwisterFast 
 Writes the entire state of the MersenneTwister RNG to the stream
   writeToCheckpoint(OutputStream)   - 
Method in class sim.engine. SimState 
 Serializes out the SimState, and the entire simulation state (not including the graphical interfaces)
        to the provided stream.
   writeToCheckpoint(File)   - 
Method in class sim.engine. SimState 
 Writes the state to a checkpoint and returns the state.
 
 
    
 X  
 
   x   - 
Variable in class sim.portrayal3d.grid.quad. ValueGridCellInfo 
  
   x   - 
Variable in class sim.util. Double2D 
  
   x   - 
Variable in class sim.util. Double3D 
  
   x   - 
Variable in class sim.util. Int2D 
  
   x   - 
Variable in class sim.util. Int3D 
  
   x   - 
Variable in class sim.util. MutableDouble2D 
  
   x   - 
Variable in class sim.util. MutableDouble3D 
  
   x   - 
Variable in class sim.util. MutableInt2D 
  
   x   - 
Variable in class sim.util. MutableInt3D 
  
   xOffset   - 
Variable in class sim.display. Display2D.InnerDisplay2D 
 x offset
 
 
    
 Y  
 
   y   - 
Variable in class sim.portrayal3d.grid.quad. ValueGridCellInfo 
  
   y   - 
Variable in class sim.util. Double2D 
  
   y   - 
Variable in class sim.util. Double3D 
  
   y   - 
Variable in class sim.util. Int2D 
  
   y   - 
Variable in class sim.util. Int3D 
  
   y   - 
Variable in class sim.util. MutableDouble2D 
  
   y   - 
Variable in class sim.util. MutableDouble3D 
  
   y   - 
Variable in class sim.util. MutableInt2D 
  
   y   - 
Variable in class sim.util. MutableInt3D 
  
   yOffset   - 
Variable in class sim.display. Display2D.InnerDisplay2D 
 y offset
 
 
    
 Z  
 
   z   - 
Variable in class sim.util. Double3D 
  
   z   - 
Variable in class sim.util. Int3D 
  
   z   - 
Variable in class sim.util. MutableDouble3D 
  
   z   - 
Variable in class sim.util. MutableInt3D 
  
   zero()   - 
Method in class sim.util. MutableDouble2D 
 Sets the values to 0 and returns it.
   zero()   - 
Method in class sim.util. MutableDouble3D 
 Sets the values to 0.
   zScale   - 
Variable in class sim.portrayal3d.grid. SparseGrid2DPortrayal3D 
  
 
 
    
 _  
 
   _setValue(int, Object)   - 
Method in class sim.util. CollectionProperties 
  
   _setValue(int, Object)   - 
Method in class sim.util. Properties 
  
   _setValue(int, Object)   - 
Method in class sim.util. SimpleProperties 
  
 
 
 A   B   C   D   E   F   G   H   I   J   K   L   M   N   O   P   Q   R   S   T   U   V   W   X   Y   Z   _  

 
   
  
 
 
 
   
 
   
          Overview     
        Package   
        Class   
          Tree     
          Deprecated     
       Index    
          Help     
   
 
 
  
 
 
 

 
  
 PREV 
 NEXT  
  
    FRAMES     
   NO FRAMES     
  
    All Classes  ');
  }
  //-->
 
 
    All Classes  
 


  
 
 
  
 

 

 
 
