# Supplementary material for: Out of the net: An agent-based model to study human movements influence on local-scale malaria transmission
Source: PLoS One. 2018 Mar 6;13(3):e0193493. doi: 10.1371/journal.pone.0193493 (PMC5839546; doi:10.1371/journal.pone.0193493)
Supplement: S2 File — (ZIP) [file pone.0193493.s002.zip › S2/docs/classdocs/overview-frame.html]

Overview List


|  |
| --- |

|  |
| --- |
| All Classes Packages   ec.util   sim.display   sim.display3d   sim.engine   sim.field   sim.field.continuous   sim.field.grid   sim.field.network   sim.portrayal   sim.portrayal.continuous   sim.portrayal.grid   sim.portrayal.network   sim.portrayal.simple   sim.portrayal3d   sim.portrayal3d.continuous   sim.portrayal3d.grid   sim.portrayal3d.grid.quad   sim.portrayal3d.simple   sim.util   sim.util.gui   sim.util.media   sim.util.media.chart |
