# Supplementary material for: Out of the net: An agent-based model to study human movements influence on local-scale malaria transmission
Source: PLoS One. 2018 Mar 6;13(3):e0193493. doi: 10.1371/journal.pone.0193493 (PMC5839546; doi:10.1371/journal.pone.0193493)
Supplement: S2 File — (ZIP) [file pone.0193493.s002.zip › S2/docs/classdocs/overview-summary.html]

Overview


---


|  |  |  |  |  |  |  |  |  |  |
| --- | --- | --- | --- | --- | --- | --- | --- | --- | --- |
| |  |  |  |  |  |  |  | | --- | --- | --- | --- | --- | --- | --- | | **Overview** | Package | Class | **Tree** | **Deprecated** | **Index** | **Help** | | |  |
| PREV   NEXT | **FRAMES**    **NO FRAMES**     **All Classes** |


---

| **Packages** | |
| --- | --- |
| **ec.util** |  |
| **sim.display** |  |
| **sim.display3d** |  |
| **sim.engine** |  |
| **sim.field** |  |
| **sim.field.continuous** |  |
| **sim.field.grid** |  |
| **sim.field.network** |  |
| **sim.portrayal** |  |
| **sim.portrayal.continuous** |  |
| **sim.portrayal.grid** |  |
| **sim.portrayal.network** |  |
| **sim.portrayal.simple** |  |
| **sim.portrayal3d** |  |
| **sim.portrayal3d.continuous** |  |
| **sim.portrayal3d.grid** |  |
| **sim.portrayal3d.grid.quad** |  |
| **sim.portrayal3d.simple** |  |
| **sim.util** |  |
| **sim.util.gui** |  |
| **sim.util.media** |  |
| **sim.util.media.chart** |  |

---


|  |  |  |  |  |  |  |  |  |  |
| --- | --- | --- | --- | --- | --- | --- | --- | --- | --- |
| |  |  |  |  |  |  |  | | --- | --- | --- | --- | --- | --- | --- | | **Overview** | Package | Class | **Tree** | **Deprecated** | **Index** | **Help** | | |  |
| PREV   NEXT | **FRAMES**    **NO FRAMES**     **All Classes** |


---
