# Supplementary material for: Out of the net: An agent-based model to study human movements influence on local-scale malaria transmission
Source: PLoS One. 2018 Mar 6;13(3):e0193493. doi: 10.1371/journal.pone.0193493 (PMC5839546; doi:10.1371/journal.pone.0193493)
Supplement: S2 File — (ZIP) [file pone.0193493.s002.zip › S2/docs/classdocs/overview-tree.html]

Class Hierarchy


---


|  |  |  |  |  |  |  |  |  |  |
| --- | --- | --- | --- | --- | --- | --- | --- | --- | --- |
| |  |  |  |  |  |  |  | | --- | --- | --- | --- | --- | --- | --- | | **Overview** | Package | Class | **Tree** | **Deprecated** | **Index** | **Help** | | |  |
| PREV   NEXT | **FRAMES**    **NO FRAMES**     **All Classes** |


---


## Hierarchy For All Packages

**Package Hierarchies:**: ec.util, sim.display, sim.display3d, sim.engine, sim.field, sim.field.continuous, sim.field.grid, sim.field.network, sim.portrayal, sim.portrayal.continuous, sim.portrayal.grid, sim.portrayal.network, sim.portrayal.simple, sim.portrayal3d, sim.portrayal3d.continuous, sim.portrayal3d.grid, sim.portrayal3d.grid.quad, sim.portrayal3d.simple, sim.util, sim.util.gui, sim.util.media, sim.util.media.chart

---

## Class Hierarchy

- java.lang.Object
  - sim.field.grid.**AbstractGrid2D** (implements sim.field.grid.Grid2D)
    - sim.field.grid.**DenseGrid2D**- sim.field.grid.**DoubleGrid2D**- sim.field.grid.**IntGrid2D**- sim.field.grid.**ObjectGrid2D**- sim.field.grid.**AbstractGrid3D** (implements sim.field.grid.Grid3D)
      - sim.field.grid.**DoubleGrid3D**- sim.field.grid.**IntGrid3D**- sim.field.grid.**ObjectGrid3D**- sim.engine.**AsynchronousSteppable** (implements sim.engine.Stoppable)- sim.util.**Bag** (implements java.lang.Cloneable, java.util.Collection<E>, sim.util.Indexed, java.io.Serializable)- java.awt.Component (implements java.awt.image.ImageObserver, java.awt.MenuContainer, java.io.Serializable)
            - java.awt.Canvas (implements javax.accessibility.Accessible)
              - javax.media.j3d.Canvas3D
                - sim.display3d.**CapturingCanvas3D**- java.awt.Container
                - javax.swing.JComponent (implements java.io.Serializable)
                  - sim.display.**Display2D** (implements sim.display.Manipulating2D, sim.engine.Steppable)- sim.display.**Display2D.InnerDisplay2D**- javax.swing.JPanel (implements javax.accessibility.Accessible)
                        - sim.util.gui.**AbstractScrollable** (implements javax.swing.Scrollable)- sim.util.media.chart.**ChartGenerator**
                            - sim.util.media.chart.**HistogramGenerator**- sim.util.media.chart.**ScatterPlotGenerator**- sim.util.media.chart.**TimeSeriesChartGenerator**- sim.util.gui.**ColorWell**- sim.util.gui.**DisclosurePanel**- sim.display3d.**Display3D** (implements sim.engine.Steppable)- sim.util.gui.**HTMLBrowser**- sim.portrayal.**Inspector**
                                      - sim.portrayal.**SimpleInspector**- sim.util.gui.**LabelledList**
                                        - sim.util.media.chart.**SeriesAttributes**
                                          - sim.util.media.chart.**HistogramSeriesAttributes**- sim.util.media.chart.**ScatterPlotSeriesAttributes**- sim.util.media.chart.**TimeSeriesAttributes**- sim.util.gui.**MiniHistogram**- sim.util.gui.**NumberTextField**- sim.util.gui.**PropertyField**- java.awt.Panel (implements javax.accessibility.Accessible)
                    - java.applet.Applet
                      - sim.display.**SimApplet**- java.awt.Window (implements javax.accessibility.Accessible)
                      - java.awt.Frame (implements java.awt.MenuContainer)
                        - javax.swing.JFrame (implements javax.accessibility.Accessible, javax.swing.RootPaneContainer, javax.swing.WindowConstants)
                          - sim.display.**Console** (implements sim.display.Controller)- sim.display.**Display2D.OptionPane**- sim.display3d.**Display3D.OptionPane3D**- sim.util.**Double2D** (implements java.io.Serializable)- sim.util.**Double3D** (implements java.io.Serializable)- sim.util.**DoubleBag** (implements java.lang.Cloneable, sim.util.Indexed, java.io.Serializable)- sim.portrayal.**DrawInfo2D**
                    - sim.portrayal.network.**EdgeDrawInfo2D**
                      - sim.portrayal.simple.**TrailedPortrayal2D.TrailDrawInfo2D**- sim.field.network.**Edge** (implements java.lang.Comparable<T>, java.io.Serializable)- sim.portrayal.**FieldPortrayal**
                        - sim.portrayal.**FieldPortrayal2D** (implements sim.portrayal.Portrayal2D)
                          - sim.portrayal.continuous.**ContinuousPortrayal2D**- sim.portrayal.network.**NetworkPortrayal2D**- sim.portrayal.grid.**ObjectGridPortrayal2D**
                                - sim.portrayal.grid.**FastObjectGridPortrayal2D**- sim.portrayal.grid.**HexaObjectGridPortrayal2D**
                                    - sim.portrayal.grid.**FastHexaObjectGridPortrayal2D**- sim.portrayal.grid.**SparseGridPortrayal2D**
                                  - sim.portrayal.grid.**HexaSparseGridPortrayal2D**- sim.portrayal.grid.**ValueGridPortrayal2D**
                                    - sim.portrayal.grid.**FastValueGridPortrayal2D**- sim.portrayal.grid.**HexaValueGridPortrayal2D**
                                        - sim.portrayal.grid.**FastHexaValueGridPortrayal2D**- sim.portrayal3d.**FieldPortrayal3D** (implements sim.portrayal3d.Portrayal3D)
                            - sim.portrayal3d.grid.**ObjectGridPortrayal3D**- sim.portrayal3d.**SparseFieldPortrayal3D**
                                - sim.portrayal3d.continuous.**ContinuousPortrayal3D**- sim.portrayal3d.grid.**SparseGridPortrayal3D**
                                    - sim.portrayal3d.grid.**SparseGrid2DPortrayal3D**- sim.portrayal3d.grid.**ValueGrid2DPortrayal3D**- sim.portrayal3d.grid.**ValueGridPortrayal3D**- sim.display.**GUIState**- sim.util.**Heap** (implements java.io.Serializable)- sim.util.**Int2D** (implements java.io.Serializable)- sim.util.**Int3D** (implements java.io.Serializable)- sim.util.**IntBag** (implements java.lang.Cloneable, sim.util.Indexed, java.io.Serializable)- sim.util.**Interval**- sim.portrayal.**LocationWrapper**- ec.util.**MersenneTwisterFast** (implements java.lang.Cloneable, java.io.Serializable)- sim.engine.**MethodStep** (implements sim.engine.Steppable)- sim.util.media.chart.**MinGapDataCuller** (implements sim.util.media.chart.DataCuller)- sim.util.media.**MovieEncoder** (implements java.io.Serializable)- sim.util.gui.**MovieMaker**- sim.engine.**MultiStep** (implements sim.engine.Steppable)- sim.util.**MutableDouble2D** (implements java.lang.Cloneable, java.io.Serializable)- sim.util.**MutableDouble3D** (implements java.lang.Cloneable, java.io.Serializable)- sim.util.**MutableInt2D** (implements java.lang.Cloneable, java.io.Serializable)- sim.util.**MutableInt3D** (implements java.lang.Cloneable, java.io.Serializable)- sim.field.network.**Network** (implements java.io.Serializable)- sim.field.network.**Network.IndexOutIn** (implements java.io.Serializable)- java.lang.Number (implements java.io.Serializable)
                                                                - sim.util.**MutableDouble** (implements java.lang.Cloneable, sim.util.Valuable)- sim.portrayal.grid.**ObjectGridPortrayal2D.Message**- sim.util.media.**PDFEncoder**- sim.util.media.**PNGEncoder**- sim.display.**Prefs**- sim.util.**Properties** (implements java.io.Serializable)
                                                                          - sim.util.**CollectionProperties**- sim.util.**SimpleProperties** (implements java.io.Serializable)- sim.portrayal3d.grid.quad.**QuadPortrayal** (implements sim.portrayal.Portrayal)
                                                                            - sim.portrayal3d.grid.quad.**MeshPortrayal**- sim.portrayal3d.grid.quad.**TilePortrayal**- sim.portrayal3d.grid.quad.**QuadPortrayal.Filter**
                                                                              - sim.portrayal3d.grid.quad.**QuadPortrayal.DoubleFilter**- sim.portrayal3d.grid.quad.**QuadPortrayal.IntFilter**- sim.portrayal3d.grid.quad.**QuadPortrayal.ObjectFilter**- sim.display.**RateAdjuster** (implements sim.engine.Steppable)- javax.media.j3d.SceneGraphObject
                                                                                  - javax.media.j3d.Node
                                                                                    - javax.media.j3d.Group
                                                                                      - javax.media.j3d.TransformGroup
                                                                                        - sim.portrayal3d.simple.**Arrow**- javax.media.j3d.Leaf
                                                                                        - javax.media.j3d.Behavior
                                                                                          - com.sun.j3d.utils.behaviors.mouse.MouseBehavior (implements java.awt.event.MouseListener, java.awt.event.MouseMotionListener)
                                                                                            - sim.display3d.**SelectionBehavior**- com.sun.j3d.utils.picking.behaviors.PickMouseBehavior
                                                                                              - sim.display3d.**ToolTipBehavior**- sim.engine.**Schedule** (implements java.io.Serializable)- sim.engine.**Schedule.Key** (implements java.lang.Comparable<T>, java.io.Serializable)- sim.engine.**Sequence** (implements sim.engine.Steppable)
                                                                                        - sim.engine.**ParallelSequence**- sim.engine.**RandomSequence**- sim.util.gui.**SimpleColorMap** (implements sim.util.gui.ColorMap)- sim.display.**SimpleController** (implements sim.display.Controller)- sim.portrayal.**SimplePortrayal2D** (implements sim.portrayal.Portrayal2D)
                                                                                              - sim.portrayal.simple.**AdjustablePortrayal2D**- sim.portrayal.simple.**FacetedPortrayal2D**- sim.portrayal.simple.**LabelledPortrayal2D**- sim.portrayal.simple.**MovablePortrayal2D**- sim.portrayal.simple.**OrientedPortrayal2D**- sim.portrayal.simple.**OvalPortrayal2D**
                                                                                                          - sim.portrayal.simple.**CircledPortrayal2D**- sim.portrayal.simple.**RectanglePortrayal2D**
                                                                                                            - sim.portrayal.simple.**ImagePortrayal2D**- sim.portrayal.simple.**ValuePortrayal2D**- sim.portrayal.simple.**ShapePortrayal2D**
                                                                                                              - sim.portrayal.simple.**HexagonalPortrayal2D**- sim.portrayal.network.**SimpleEdgePortrayal2D**- sim.portrayal.simple.**TrailedPortrayal2D**- sim.portrayal.simple.**TransformedPortrayal2D**- sim.portrayal3d.**SimplePortrayal3D** (implements sim.portrayal3d.Portrayal3D)
                                                                                                - sim.portrayal3d.simple.**AxesPortrayal3D**- sim.portrayal3d.simple.**CircledPortrayal3D**- sim.portrayal3d.simple.**CubePortrayal3D**- sim.portrayal3d.simple.**ImagePortrayal3D**- sim.portrayal3d.simple.**LabelledPortrayal3D**- sim.portrayal3d.simple.**LightPortrayal3D**- sim.portrayal3d.simple.**PrimitivePortrayal3D**
                                                                                                              - sim.portrayal3d.simple.**BranchGroupPortrayal3D**- sim.portrayal3d.simple.**ConePortrayal3D**- sim.portrayal3d.simple.**CylinderPortrayal3D**- sim.portrayal3d.simple.**Shape3DPortrayal3D**
                                                                                                                      - sim.portrayal3d.simple.**ValuePortrayal3D**- sim.portrayal3d.simple.**SpherePortrayal3D**- sim.portrayal3d.simple.**SharedPortrayal3D**- sim.portrayal3d.simple.**TransformedPortrayal3D**- sim.portrayal3d.simple.**WireFrameBoxPortrayal3D**- sim.engine.**SimState** (implements java.io.Serializable)- sim.field.**SparseField** (implements java.io.Serializable)
                                                                                                    - sim.field.continuous.**Continuous2D** (implements sim.field.SparseField2D)- sim.field.continuous.**Continuous3D** (implements sim.field.SparseField3D)- sim.field.grid.**SparseGrid2D** (implements sim.field.grid.Grid2D, sim.field.SparseField2D)- sim.field.grid.**SparseGrid3D** (implements sim.field.SparseField3D)- sim.field.**SparseField.LocationAndIndex** (implements java.io.Serializable)- sim.portrayal.network.**SpatialNetwork2D**- sim.engine.**TentativeStep** (implements sim.engine.Steppable, sim.engine.Stoppable)- java.lang.Throwable (implements java.io.Serializable)
                                                                                                            - java.lang.Exception
                                                                                                              - java.lang.RuntimeException
                                                                                                                - sim.util.**CausedRuntimeException**- sim.util.gui.**Utilities**- sim.portrayal3d.grid.quad.**ValueGridCellInfo**- sim.portrayal.simple.**ValuePortrayal2D.Filter**
                                                                                                                  - sim.portrayal.simple.**ValuePortrayal2D.DoubleFilter**- sim.portrayal.simple.**ValuePortrayal2D.IntFilter**- sim.portrayal3d.simple.**ValuePortrayal3D.Filter**
                                                                                                                    - sim.portrayal3d.simple.**ValuePortrayal3D.DoubleFilter**- sim.portrayal3d.simple.**ValuePortrayal3D.IntFilter**- sim.engine.**WeakStep** (implements sim.engine.Steppable)- sim.util.gui.**WordWrap** (implements java.io.Serializable)

## Interface Hierarchy

- sim.util.gui.**ColorMap**- sim.display.**Controller**- sim.util.media.chart.**DataCuller**- sim.portrayal.grid.**DrawPolicy**- sim.portrayal.**Fixed2D**- sim.util.**Indexed**- sim.engine.**MakesSimState**- sim.display.**Manipulating2D**- sim.portrayal.**Oriented2D**
                  - sim.portrayal.**Orientable2D**- sim.util.**Propertied**- sim.util.**Proxiable**- sim.portrayal.**Scalable2D**- java.io.Serializable
                          - sim.field.grid.**Grid2D**- sim.field.grid.**Grid3D**- sim.portrayal.**Portrayal**
                                - sim.portrayal.**Portrayal2D**- sim.portrayal3d.**Portrayal3D**- sim.engine.**Steppable**- sim.engine.**Stoppable**- sim.field.**SparseField2D**- sim.field.**SparseField3D**- sim.util.**Valuable**

---


|  |  |  |  |  |  |  |  |  |  |
| --- | --- | --- | --- | --- | --- | --- | --- | --- | --- |
| |  |  |  |  |  |  |  | | --- | --- | --- | --- | --- | --- | --- | | **Overview** | Package | Class | **Tree** | **Deprecated** | **Index** | **Help** | | |  |
| PREV   NEXT | **FRAMES**    **NO FRAMES**     **All Classes** |


---
