# Supplementary material for: Out of the net: An agent-based model to study human movements influence on local-scale malaria transmission
Source: PLoS One. 2018 Mar 6;13(3):e0193493. doi: 10.1371/journal.pone.0193493 (PMC5839546; doi:10.1371/journal.pone.0193493)
Supplement: S2 File — (ZIP) [file pone.0193493.s002.zip › S2/docs/classdocs/serialized-form.html]

Serialized Form


---


|  |  |  |  |  |  |  |  |  |  |
| --- | --- | --- | --- | --- | --- | --- | --- | --- | --- |
| |  |  |  |  |  |  |  | | --- | --- | --- | --- | --- | --- | --- | | **Overview** | Package | Class | **Tree** | **Deprecated** | **Index** | **Help** | | |  |
| PREV   NEXT | **FRAMES**    **NO FRAMES**     **All Classes** |


---


# Serialized Form


---

| **Package** **ec.util** |
| --- |

| **Class ec.util.MersenneTwisterFast extends java.lang.Object implements Serializable** | |
| --- | --- |

**serialVersionUID:**-8219700664442619525L

| **Serialized Fields** |
| --- |

### mt

```
int[] mt
```

---

### mti

```
int mti
```

---

### mag01

```
int[] mag01
```

---

### \_\_nextNextGaussian

```
double __nextNextGaussian
```

---

### \_\_haveNextNextGaussian

```
boolean __haveNextNextGaussian
```

---

| **Package** **sim.display** |
| --- |

| **Class sim.display.Console extends javax.swing.JFrame implements Serializable** | |
| --- | --- |

| **Serialized Fields** |
| --- |

### simulation

```
GUIState simulation
```

:   Our simulation

---

### newMenuAllowed

```
boolean newMenuAllowed
```

:   Do we only allow the user to choose the 'New Simulation' menu?

---

### preferredInspectorIndex

```
int preferredInspectorIndex
```

:   Keep track of the last inspector selected so it stays selected after user picks a different area.

---

### infoPanel

```
javax.swing.JComponent infoPanel
```

:   The HTML display's container panel

---

### time

```
javax.swing.JLabel time
```

:   The current time

---

### slider

```
javax.swing.JSlider slider
```

:   The frame rate

---

### sliderText

```
javax.swing.JLabel sliderText
```

:   The associated text with the speed of play slider

---

### stepSlider

```
javax.swing.JSlider stepSlider
```

:   The slider which controls the number of steps per press of the step-button

---

### stepSliderText

```
javax.swing.JLabel stepSliderText
```

:   The associated text for number of steps per press of the step-button

---

### repeatButton

```
javax.swing.JCheckBox repeatButton
```

:   The checkbox which states whether or not we should give way just a little bit

---

### stopButton

```
javax.swing.JButton stopButton
```

:   The stop button

---

### playButton

```
javax.swing.JButton playButton
```

:   The play button

---

### pauseButton

```
javax.swing.JButton pauseButton
```

:   The pause button

---

### tabPane

```
javax.swing.JTabbedPane tabPane
```

:   The top-level tabbed view

---

### frameListDisplay

```
javax.swing.JList frameListDisplay
```

:   The list of frames shown in the "Displays" tab

---

### frameList

```
java.util.Vector<E> frameList
```

:   The actual list of frames used in frameListDisplay

---

### endField

```
PropertyField endField
```

:   Where the user can enter in a step count to stop at

---

### pauseField

```
PropertyField pauseField
```

:   Where the user can enter in a step count to pause at

---

### timeEndField

```
PropertyField timeEndField
```

:   Where the user can enter in a time to stop at

---

### timePauseField

```
PropertyField timePauseField
```

:   Where the user can enter in a time to pause at

---

### randomField

```
PropertyField randomField
```

:   Where the user can enter a new random number seed

---

### menuBar

```
javax.swing.JMenuBar menuBar
```

:   The Console's menu bar

---

### newMenu

```
javax.swing.JMenuItem newMenu
```

:   The 'New Simulation' menu.

---

### innerInspectorPanel

```
javax.swing.JSplitPane innerInspectorPanel
```

:   The split pane shown under the "Inspectors" tab, holding the list of
    inspectors at top, and specific inspectors at bottom

---

### inspectorPanel

```
javax.swing.JPanel inspectorPanel
```

:   An outer panel which holds the innerInspectorPanel, plus associated buttons

---

### incrementSeedOnStop

```
javax.swing.JCheckBox incrementSeedOnStop
```

:   The checkbox for whether or not the random seed should be incremented each play-button press

---

### inspectorList

```
javax.swing.JList inspectorList
```

:   The list of inspectors at the top of the split pane

---

### inspectorSwitcher

```
javax.swing.JPanel inspectorSwitcher
```

:   Holds the inspectors shown at the bottom of the split pane (if any)

---

### inspectorCardLayout

```
java.awt.CardLayout inspectorCardLayout
```

:   The card layout which enables inspectorSwitcher to show various inspectors

---

### detatchButton

```
javax.swing.JButton detatchButton
```

:   The button for detatching inspectors

---

### removeButton

```
javax.swing.JButton removeButton
```

:   The button for emptying the inspector list

---

### modelInspector

```
Inspector modelInspector
```

:   The global model inspector, if any

---

### modelInspectorScrollPane

```
javax.swing.JScrollPane modelInspectorScrollPane
```

:   The JScrollPane which holds the global model inspector, if any

---

### buttonBox

```
javax.swing.Box buttonBox
```

:   The box which holds the play/stop/pause buttons, and the time and rate fields.

---

### timeBox

```
javax.swing.JComboBox timeBox
```

:   The combo box which specifies what's displayed in the time field

---

### randomSeed

```
long randomSeed
```

:   Random number generator seed

---

### numStepsPerStepButtonPress

```
int numStepsPerStepButtonPress
```

:   how many steps we should take on one press of the "step" button. As this is only relevant
    when there is NO underlying play thread (stepping happens inside the event loop, with the
    play thread killed), it can be safely set, but only do so from the event loop.

---

### shouldRepeat

```
boolean shouldRepeat
```

:   Should the simulation repeat when the stop button is pressed?

---

### threadPriority

```
int threadPriority
```

:   What should the simulation thread priority be? Don't play with this.

---

### whenShouldEnd

```
long whenShouldEnd
```

:   When should the simulation end? Don't play with this.

---

### whenShouldPause

```
long whenShouldPause
```

:   When should the simulation pause? Don't play with this.

---

### whenShouldEndTime

```
double whenShouldEndTime
```

:   When should the simulation end? Don't play with this.

---

### whenShouldPauseTime

```
double whenShouldPauseTime
```

:   When should the simulation pause? Don't play with this.

---

### playSleep

```
long playSleep
```

:   Milliseconds of how long we should sleep between each step. Don't play with this.

---

### playThread

```
java.lang.Thread playThread
```

:   The thread that actually goes through the steps

---

### playThreadLock

```
java.lang.Object playThreadLock
```

:   A general lock used by a number of short methods which need to "synchronize on the play thread"
    even if it's changing to another thread. To do this, we use this official 'play thread lock'

---

### threadShouldStop

```
boolean threadShouldStop
```

:   Whether the thread should stop. Don't play with this.

---

### playState

```
int playState
```

:   The current state of the simulation: playing, stopped, or paused. Don't play with this.

---

### isClosing

```
boolean isClosing
```

:   Private internal flag which indicates if the program is already in the process of quitting.

---

### isClosingLock

```
java.lang.Object isClosingLock
```

:   Private lock used by doClose() to avoid synchronizing on Console.

---

### simulationFile

```
java.io.File simulationFile
```

:   The last filename the user requested. Used to open file dialogs intelligently

---

### requiresConfirmationToStop

```
boolean requiresConfirmationToStop
```

---

### lastTime

```
double lastTime
```

:   The last value the time was set to.

---

### lastRate

```
double lastRate
```

:   The last value the frame rate was set to.

---

### lastSteps

```
long lastSteps
```

---

### showing

```
int showing
```

---

### rateFormat

```
java.text.NumberFormat rateFormat
```

:   How the frame rate should look

---

### lastText

```
java.lang.String lastText
```

---

### blocker

```
java.lang.Runnable blocker
```

:   Used to block until a repaint is handled -- see spawnPlayThread() below

---

### inspectorNames

```
java.util.Vector<E> inspectorNames
```

:   Holds the names for each inspector presently in the inspectorSwitcher

---

### inspectorStoppables

```
java.util.Vector<E> inspectorStoppables
```

:   Holds the Stoppable objects for each inspector presently in the inspectorSwitcher.

---

### inspectorToolbars

```
java.util.Vector<E> inspectorToolbars
```

:   Holds the toolbars wrapping each inspector presently in the inspectorSwitcher.

---

### allInspectors

```
java.util.WeakHashMap<K,V> allInspectors
```

:   Weakly maps inspectors to their stoppables for all inspectors that might possibly be around.
    Cleaned out when the user presses play.
    As inspectors are closed or eliminated, they may disappear from this WeakHashMap and be garbage collected.

| **Class sim.display.Display2D extends javax.swing.JComponent implements Serializable** | |
| --- | --- |

| **Serialized Fields** |
| --- |

### precise

```
boolean precise
```

---

### DEFAULT\_PREFERENCES\_KEY

```
java.lang.String DEFAULT_PREFERENCES_KEY
```

---

### preferencesKey

```
java.lang.String preferencesKey
```

---

### useTooltips

```
boolean useTooltips
```

:   Use tool tips?

---

### lastEncodedSteps

```
long lastEncodedSteps
```

:   The last steps for a frame that was painted to the screen. Keeping this
    variable around enables our movie maker to ensure that it doesn't write
    a frame twice to its movie stream.

---

### movieMaker

```
MovieMaker movieMaker
```

:   Our movie maker, if one is running, else null.

---

### insideDisplay

```
Display2D.InnerDisplay2D insideDisplay
```

:   The 2D display inside the scroll view. Does the actual drawing of the simulation.

---

### optionPane

```
Display2D.OptionPane optionPane
```

:   Our option pane

---

### portrayals

```
java.util.ArrayList<E> portrayals
```

:   The list of portrayals the insideDisplay draws. Each element in this list is a Portrayal2DHolder.

---

### display

```
javax.swing.JScrollPane display
```

:   The scroll view which holds the insideDisplay.

---

### port

```
javax.swing.JViewport port
```

:   The scroll view's viewport.

---

### stopper

```
Stoppable stopper
```

:   The stoppable for the repeat object which redraws the Display2D in the schedule.

---

### simulation

```
GUIState simulation
```

:   The simulation proper.

---

### header

```
javax.swing.Box header
```

:   The component bar at the top of the Display2D.

---

### popup

```
javax.swing.JPopupMenu popup
```

:   The popup layers menu

---

### layersbutton

```
javax.swing.JToggleButton layersbutton
```

:   The button which pops up the layers menu

---

### refreshPopup

```
javax.swing.JPopupMenu refreshPopup
```

:   The refresh menu

---

### refreshbutton

```
javax.swing.JToggleButton refreshbutton
```

:   The button which pops up the refresh menu

---

### movieButton

```
javax.swing.JButton movieButton
```

:   The button which starts or stops a movie

---

### snapshotButton

```
javax.swing.JButton snapshotButton
```

:   The button which snaps a screenshot

---

### optionButton

```
javax.swing.JButton optionButton
```

:   The button which pops up the option pane

---

### scaleField

```
NumberTextField scaleField
```

:   The field for scaling values

---

### skipField

```
NumberTextField skipField
```

:   The field for skipping frames

---

### skipBox

```
javax.swing.JComboBox skipBox
```

:   The combo box for skipping frames

---

### skipFrame

```
javax.swing.JFrame skipFrame
```

:   The frame which holds the skip controls

---

### scale

```
double scale
```

:   Scale (zoom value). 1.0 is 1:1. 2.0 is zoomed in 2 times. Etc.

---

### scaleLock

```
java.lang.Object scaleLock
```

---

### clipping

```
boolean clipping
```

:   Whether or not we're clipping

---

### backdrop

```
java.awt.Paint backdrop
```

:   Backdrop color or other paint. This is the color/paint that the simulation is whitewashed with prior to
    the portrayals redrawing themselves. This differs from the scroll view's BACKGROUND
    color, which is the color of any area that the simulation doesn't draw on.

---

### selectedWrappers

```
java.util.ArrayList<E> selectedWrappers
```

---

### sacrificialObj

```
java.lang.Object sacrificialObj
```

:   Takes a snapshot of the Display2D's currently displayed simulation.
    Ought only be done from the main event loop.

---

### updateRule

```
int updateRule
```

---

### stepInterval

```
long stepInterval
```

---

### timeInterval

```
double timeInterval
```

---

### wallInterval

```
long wallInterval
```

---

### lastStep

```
long lastStep
```

---

### lastTime

```
double lastTime
```

---

### lastWall

```
long lastWall
```

| **Class sim.display.Display2D.InnerDisplay2D extends javax.swing.JComponent implements Serializable** | |
| --- | --- |

| **Serialized Fields** |
| --- |

### buffer

```
java.awt.image.BufferedImage buffer
```

:   Image buffer for doing buffered draws, mostly for screenshots etc.

---

### width

```
double width
```

:   The width of the display when the scale is 1.0

---

### height

```
double height
```

:   The height of the display when the scale is 1.0

---

### xOffset

```
double xOffset
```

:   x offset

---

### yOffset

```
double yOffset
```

:   y offset

---

### unbufferedHints

```
java.awt.RenderingHints unbufferedHints
```

:   Hints used to draw objects to the screen or to a buffer

---

### bufferedHints

```
java.awt.RenderingHints bufferedHints
```

:   Hints used to draw the buffered image to the screen

---

### toolTip

```
java.lang.ref.WeakReference<T> toolTip
```

---

### lastToolTipEvent

```
java.awt.event.MouseEvent lastToolTipEvent
```

---

### lastToolTipText

```
java.lang.String lastToolTipText
```

---

### paintLock

```
boolean paintLock
```

---

### viewRect

```
java.awt.Rectangle viewRect
```

---

### viewRectLock

```
java.lang.Object viewRectLock
```

:   Lock for the viewRect above. Don't want to lock on the Display2D itself.

| **Class sim.display.Display2D.OptionPane extends javax.swing.JFrame implements Serializable** | |
| --- | --- |

| **Serialized Fields** |
| --- |

### buffering

```
int buffering
```

---

### useNoBuffer

```
javax.swing.JRadioButton useNoBuffer
```

---

### useBuffer

```
javax.swing.JRadioButton useBuffer
```

---

### useDefault

```
javax.swing.JRadioButton useDefault
```

---

### usageGroup

```
javax.swing.ButtonGroup usageGroup
```

---

### antialias

```
javax.swing.JCheckBox antialias
```

---

### alphaInterpolation

```
javax.swing.JCheckBox alphaInterpolation
```

---

### interpolation

```
javax.swing.JCheckBox interpolation
```

---

### tooltips

```
javax.swing.JCheckBox tooltips
```

---

### systemPreferences

```
javax.swing.JButton systemPreferences
```

---

### appPreferences

```
javax.swing.JButton appPreferences
```

---

### xOffsetField

```
NumberTextField xOffsetField
```

---

### yOffsetField

```
NumberTextField yOffsetField
```

---

### listener

```
java.awt.event.ActionListener listener
```

| **Class sim.display.RateAdjuster extends java.lang.Object implements Serializable** | |
| --- | --- |

| **Serialized Fields** |
| --- |

### initialTime

```
long initialTime
```

---

### totalTics

```
long totalTics
```

---

### started

```
boolean started
```

---

### rate

```
double rate
```

| **Class sim.display.SimApplet extends java.applet.Applet implements Serializable** | |
| --- | --- |

---

| **Package** **sim.display3d** |
| --- |

| **Class sim.display3d.CapturingCanvas3D extends javax.media.j3d.Canvas3D implements Serializable** | |
| --- | --- |

| **Serialized Fields** |
| --- |

### writeBuffer\_

```
boolean writeBuffer_
```

---

### keepOnWriting\_

```
boolean keepOnWriting_
```

---

### buffer\_

```
java.awt.image.BufferedImage buffer_
```

---

### x

```
int x
```

---

### y

```
int y
```

---

### width

```
int width
```

---

### height

```
int height
```

| **Class sim.display3d.Display3D extends javax.swing.JPanel implements Serializable** | |
| --- | --- |

| **Serialized Fields** |
| --- |

### DEFAULT\_PREFERENCES\_KEY

```
java.lang.String DEFAULT_PREFERENCES_KEY
```

---

### preferencesKey

```
java.lang.String preferencesKey
```

---

### portrayals

```
java.util.ArrayList<E> portrayals
```

---

### stopper

```
Stoppable stopper
```

---

### simulation

```
GUIState simulation
```

---

### header

```
javax.swing.Box header
```

:   The component bar at the top of the Display3D.

---

### movieButton

```
javax.swing.JButton movieButton
```

:   The button which starts or stops a movie

---

### snapshotButton

```
javax.swing.JButton snapshotButton
```

:   The button which snaps a screenshot

---

### optionButton

```
javax.swing.JButton optionButton
```

:   The button which pops up the option pane

---

### refreshPopup

```
javax.swing.JPopupMenu refreshPopup
```

:   The field for scaling values

---

### refreshbutton

```
javax.swing.JToggleButton refreshbutton
```

:   The button which pops up the refresh menu

---

### scaleField

```
NumberTextField scaleField
```

:   The button which starts or stops a movie

---

### skipField

```
NumberTextField skipField
```

:   The field for skipping frames

---

### skipBox

```
javax.swing.JComboBox skipBox
```

:   The combo box for skipping frames

---

### skipFrame

```
javax.swing.JFrame skipFrame
```

:   The frame which holds the skip controls

---

### canvas

```
CapturingCanvas3D canvas
```

:   The Java3D canvas holding the universe. A good time to fool around with this is
    in the sceneGraphCreated() hook.

---

### universe

```
com.sun.j3d.utils.universe.SimpleUniverse universe
```

:   The Java3D universe. Created (and recreated) by createSceneGraph. A good time to fool around with this is
    in the sceneGraphCreated() hook.

---

### root

```
javax.media.j3d.BranchGroup root
```

:   The root scene graph node in the Java3D universe. Created (and recreated) by createSceneGraph.
    This is a good place to hang things you don't want auto-rotated nor transformed by the Display3D. Hang things off of here
    in the sceneGraphCreated() hook.

---

### viewRoot

```
javax.media.j3d.BranchGroup viewRoot
```

:   An additional root scene graph node which is attached to the viewing transform of the universe, and thus
    stays in the same location regardless of the placement of the camera.

---

### portrayalSwitch

```
javax.media.j3d.Switch portrayalSwitch
```

---

### portrayalSwitchMask

```
java.util.BitSet portrayalSwitchMask
```

---

### auxillarySwitch

```
javax.media.j3d.Switch auxillarySwitch
```

---

### auxillarySwitchMask

```
java.util.BitSet auxillarySwitchMask
```

---

### lightSwitch

```
javax.media.j3d.Switch lightSwitch
```

:   Holds two lights located at the camera: in slot 0, a white PointLight, and in slot 1, a white AmbientLight.
    You may change these lights to different colored lights, but please keep them PointLights and AmbientLights
    respectively. These lights are turned on and off by the Options pane.

---

### lightSwitchMask

```
java.util.BitSet lightSwitchMask
```

---

### movieMaker

```
MovieMaker movieMaker
```

---

### popup

```
javax.swing.JPopupMenu popup
```

:   The popup layers menu

---

### layersbutton

```
javax.swing.JToggleButton layersbutton
```

:   The button which pops up the layers menu

---

### subgraphCount

```
int subgraphCount
```

---

### dirty

```
boolean dirty
```

---

### backdropAppearance

```
javax.media.j3d.Appearance backdropAppearance
```

---

### backdropImage

```
java.awt.Image backdropImage
```

---

### backdropColor

```
java.awt.Color backdropColor
```

---

### bogusMover

```
javax.media.j3d.PointArray bogusMover
```

---

### globalModelTransformGroup

```
javax.media.j3d.TransformGroup globalModelTransformGroup
```

:   The TransformGroup which holds the switch holding the portrayal's scene graph models.
    A good time to fool around with this is in the sceneGraphCreated() hook.
    This is a good place to hang stuff which you want to get rotated AND transformed along with the scene graph.

---

### toolTipBehavior

```
ToolTipBehavior toolTipBehavior
```

---

### usingToolTips

```
boolean usingToolTips
```

---

### scale

```
double scale
```

---

### scaleLock

```
java.lang.Object scaleLock
```

---

### autoSpin

```
javax.media.j3d.RotationInterpolator autoSpin
```

---

### autoSpinBackground

```
javax.media.j3d.RotationInterpolator autoSpinBackground
```

---

### autoSpinTransformGroup

```
javax.media.j3d.TransformGroup autoSpinTransformGroup
```

:   The TransformGroup which used to spin the underlying model.
    This is a good place to hang stuff which you want to get rotated along with the models in the scene graph,
    but DON'T want transformed along with the scene graph.

---

### autoSpinBackgroundTransformGroup

```
javax.media.j3d.TransformGroup autoSpinBackgroundTransformGroup
```

---

### mOrbitBehavior

```
com.sun.j3d.utils.behaviors.vp.OrbitBehavior mOrbitBehavior
```

---

### mSelectBehavior

```
SelectionBehavior mSelectBehavior
```

---

### selectionAll

```
boolean selectionAll
```

---

### inspectionAll

```
boolean inspectionAll
```

---

### updateRule

```
int updateRule
```

---

### stepInterval

```
long stepInterval
```

---

### timeInterval

```
double timeInterval
```

---

### wallInterval

```
long wallInterval
```

---

### lastStep

```
long lastStep
```

---

### lastTime

```
double lastTime
```

---

### lastWall

```
long lastWall
```

---

### orbitRotateXCheckBox

```
javax.swing.JCheckBox orbitRotateXCheckBox
```

---

### orbitRotateYCheckBox

```
javax.swing.JCheckBox orbitRotateYCheckBox
```

---

### orbitTranslateXCheckBox

```
javax.swing.JCheckBox orbitTranslateXCheckBox
```

---

### orbitTranslateYCheckBox

```
javax.swing.JCheckBox orbitTranslateYCheckBox
```

---

### orbitZoomCheckBox

```
javax.swing.JCheckBox orbitZoomCheckBox
```

---

### selectBehCheckBox

```
javax.swing.JCheckBox selectBehCheckBox
```

---

### polyPoint

```
javax.swing.JRadioButton polyPoint
```

---

### polyLine

```
javax.swing.JRadioButton polyLine
```

---

### polyFill

```
javax.swing.JRadioButton polyFill
```

---

### polyCullNone

```
javax.swing.JRadioButton polyCullNone
```

---

### polyCullFront

```
javax.swing.JRadioButton polyCullFront
```

---

### polyCullBack

```
javax.swing.JRadioButton polyCullBack
```

---

### showAxesCheckBox

```
javax.swing.JCheckBox showAxesCheckBox
```

---

### showBackgroundCheckBox

```
javax.swing.JCheckBox showBackgroundCheckBox
```

---

### tooltips

```
javax.swing.JCheckBox tooltips
```

---

### showSpotlightCheckBox

```
javax.swing.JCheckBox showSpotlightCheckBox
```

---

### showAmbientLightCheckBox

```
javax.swing.JCheckBox showAmbientLightCheckBox
```

---

### rotAxis\_X

```
NumberTextField rotAxis_X
```

---

### rotAxis\_Y

```
NumberTextField rotAxis_Y
```

---

### rotAxis\_Z

```
NumberTextField rotAxis_Z
```

---

### spinDuration

```
NumberTextField spinDuration
```

---

### rasterizationMode

```
int rasterizationMode
```

:   Sets the rasterization mode for configurable polygon portrayals.
    Mode can be PolygonAttributes.POLYGON\_FILL, PolygonAttributes.POLYGON\_LINE,
    or PolygonAttributes.POLYGON\_POINT.

---

### cullingMode

```
int cullingMode
```

:   Sets the rasterization mode for configurable polygon portrayals.
    Mode can be PolygonAttributes.CULL\_BACK, PolygonAttributes.CULL\_FRONT,
    or PolygonAttributes.CULL\_NONE.

---

### selectedWrappers

```
java.util.ArrayList<E> selectedWrappers
```

---

### systemPreferences

```
javax.swing.JButton systemPreferences
```

---

### appPreferences

```
javax.swing.JButton appPreferences
```

---

### optionPane

```
Display3D.OptionPane3D optionPane
```

| **Class sim.display3d.Display3D.OptionPane3D extends javax.swing.JFrame implements Serializable** | |
| --- | --- |

---

| **Package** **sim.engine** |
| --- |

| **Class sim.engine.AsynchronousSteppable extends java.lang.Object implements Serializable** | |
| --- | --- |

| **Serialization Methods** |
| --- |

### readObject

```
private void readObject(java.io.ObjectInputStream p)
                 throws java.io.IOException,
                        java.lang.ClassNotFoundException
```

:   **Throws:**: `java.io.IOException`: `java.lang.ClassNotFoundException`

---


### writeObject

```
private void writeObject(java.io.ObjectOutputStream p)
                  throws java.io.IOException
```

:   **Throws:**: `java.io.IOException`

| **Serialized Fields** |
| --- |

### thread

```
java.lang.Thread thread
```

---

### running

```
boolean running
```

---

### paused

```
boolean paused
```

---

### state

```
SimState state
```

| **Class sim.engine.MethodStep extends java.lang.Object implements Serializable** | |
| --- | --- |

| **Serialized Fields** |
| --- |

### method

```
java.lang.reflect.Method method
```

---

### object

```
java.lang.Object object
```

---

### passInSimState

```
boolean passInSimState
```

| **Class sim.engine.MultiStep extends java.lang.Object implements Serializable** | |
| --- | --- |

| **Serialized Fields** |
| --- |

### current

```
int current
```

---

### countdown

```
boolean countdown
```

---

### n

```
int n
```

---

### step

```
Steppable step
```

| **Class sim.engine.ParallelSequence extends Sequence implements Serializable** | |
| --- | --- |

**serialVersionUID:**2731888904476273479L

| **Serialization Methods** |
| --- |

### readObject

```
private void readObject(java.io.ObjectInputStream p)
                 throws java.io.IOException,
                        java.lang.ClassNotFoundException
```

:   **Throws:**: `java.io.IOException`: `java.lang.ClassNotFoundException`

---


### writeObject

```
private void writeObject(java.io.ObjectOutputStream p)
                  throws java.io.IOException
```

:   **Throws:**: `java.io.IOException`

| **Serialized Fields** |
| --- |

### semaphore

```
sim.engine.ParallelSequence.Semaphore semaphore
```

---

### workers

```
sim.engine.ParallelSequence.Worker[] workers
```

---

### threads

```
java.lang.Thread[] threads
```

---

### pleaseDie

```
boolean pleaseDie
```

---

### operating

```
boolean operating
```

---

### destroysThreads

```
boolean destroysThreads
```

| **Class sim.engine.RandomSequence extends Sequence implements Serializable** | |
| --- | --- |

| **Serialized Fields** |
| --- |

### shouldSynchronize

```
boolean shouldSynchronize
```

| **Class sim.engine.Schedule extends java.lang.Object implements Serializable** | |
| --- | --- |

| **Serialized Fields** |
| --- |

### shuffling

```
boolean shuffling
```

---

### queue

```
Heap queue
```

---

### time

```
double time
```

---

### steps

```
long steps
```

---

### sealed

```
boolean sealed
```

---

### lock

```
java.lang.Object lock
```

---

### currentSteps

```
Bag currentSteps
```

---

### substeps

```
Bag substeps
```

---

### inStep

```
boolean inStep
```

| **Class sim.engine.Schedule.Key extends java.lang.Object implements Serializable** | |
| --- | --- |

| **Serialized Fields** |
| --- |

### time

```
double time
```

---

### ordering

```
int ordering
```

| **Class sim.engine.Sequence extends java.lang.Object implements Serializable** | |
| --- | --- |

| **Serialized Fields** |
| --- |

### steps

```
Steppable[] steps
```

| **Class sim.engine.SimState extends java.lang.Object implements Serializable** | |
| --- | --- |

| **Serialized Fields** |
| --- |

### random

```
MersenneTwisterFast random
```

:   The SimState's random number generator

---

### schedule

```
Schedule schedule
```

:   SimState's schedule

---

### asynchronous

```
java.util.HashSet<E> asynchronous
```

---

### asynchronousLock

```
java.lang.Object asynchronousLock
```

---

### cleaningAsynchronous

```
boolean cleaningAsynchronous
```

---

### job

```
long job
```

---

### seed

```
long seed
```

| **Class sim.engine.TentativeStep extends java.lang.Object implements Serializable** | |
| --- | --- |

| **Serialized Fields** |
| --- |

### step

```
Steppable step
```

| **Class sim.engine.WeakStep extends java.lang.Object implements Serializable** | |
| --- | --- |

| **Serialization Methods** |
| --- |

### readObject

```
private void readObject(java.io.ObjectInputStream p)
                 throws java.io.IOException,
                        java.lang.ClassNotFoundException
```

:   **Throws:**: `java.io.IOException`: `java.lang.ClassNotFoundException`

---


### writeObject

```
private void writeObject(java.io.ObjectOutputStream p)
                  throws java.io.IOException
```

:   **Throws:**: `java.io.IOException`

| **Serialized Fields** |
| --- |

### weakStep

```
java.lang.ref.WeakReference<T> weakStep
```

---

### stop

```
Stoppable stop
```

---

| **Package** **sim.field** |
| --- |

| **Class sim.field.SparseField extends java.lang.Object implements Serializable** | |
| --- | --- |

| **Serialized Fields** |
| --- |

### removeEmptyBags

```
boolean removeEmptyBags
```

:   Should we remove bags in the field if they have been emptied, and let them GC, or should
    we keep them around? This doesn't include the allObjects bag.

---

### replaceLargeBags

```
boolean replaceLargeBags
```

:   When a bag drops to one quarter capacity, should we replace it with a new bag? This doesn't include the allObjects bag.

---

### locationAndIndexHash

```
java.util.HashMap<K,V> locationAndIndexHash
```

:   LocationAndIndex objects (locations and indexes into the allObjects array) hashed by Object. Ideally you would
    store only immutable or hash-by-pointer objects, el se they'll get lost in the HashMap.

---

### objectHash

```
java.util.HashMap<K,V> objectHash
```

:   Bags of objects hashed by location. Do not rely on these bags always being the same objects.

---

### allObjects

```
Bag allObjects
```

:   All the objects in the sparse field. For fast scans. Do not rely on this bag always being the same object.

| **Class sim.field.SparseField.LocationAndIndex extends java.lang.Object implements Serializable** | |
| --- | --- |

| **Serialized Fields** |
| --- |

### location

```
java.lang.Object location
```

---

### index

```
int index
```

---

### otherObjectsAtLocation

```
Bag otherObjectsAtLocation
```

---

| **Package** **sim.field.continuous** |
| --- |

| **Class sim.field.continuous.Continuous2D extends SparseField implements Serializable** | |
| --- | --- |

| **Serialized Fields** |
| --- |

### doubleLocationHash

```
java.util.HashMap<K,V> doubleLocationHash
```

:   Where we store the Double2D values hashed by object

---

### width

```
double width
```

---

### height

```
double height
```

---

### discretization

```
double discretization
```

---

### speedyMutableInt2D

```
MutableInt2D speedyMutableInt2D
```

| **Class sim.field.continuous.Continuous3D extends SparseField implements Serializable** | |
| --- | --- |

| **Serialized Fields** |
| --- |

### doubleLocationHash

```
java.util.HashMap<K,V> doubleLocationHash
```

:   Where we store the Double3D values hashed by object

---

### width

```
double width
```

---

### height

```
double height
```

---

### length

```
double length
```

---

### discretization

```
double discretization
```

---

### speedyMutableInt3D

```
MutableInt3D speedyMutableInt3D
```

---

| **Package** **sim.field.grid** |
| --- |

| **Class sim.field.grid.AbstractGrid2D extends java.lang.Object implements Serializable** | |
| --- | --- |

| **Serialized Fields** |
| --- |

### width

```
int width
```

---

### height

```
int height
```

| **Class sim.field.grid.AbstractGrid3D extends java.lang.Object implements Serializable** | |
| --- | --- |

| **Serialized Fields** |
| --- |

### width

```
int width
```

---

### height

```
int height
```

---

### length

```
int length
```

| **Class sim.field.grid.DenseGrid2D extends AbstractGrid2D implements Serializable** | |
| --- | --- |

| **Serialized Fields** |
| --- |

### removeEmptyBags

```
boolean removeEmptyBags
```

:   Should we remove bags in the field if they have been emptied, and let them GC, or should
    we keep them around?

---

### replaceLargeBags

```
boolean replaceLargeBags
```

:   When a bag drops to one quarter capacity, should we replace it with a new bag?

---

### field

```
Bag[][] field
```

| **Class sim.field.grid.DoubleGrid2D extends AbstractGrid2D implements Serializable** | |
| --- | --- |

| **Serialized Fields** |
| --- |

### field

```
double[][] field
```

| **Class sim.field.grid.DoubleGrid3D extends AbstractGrid3D implements Serializable** | |
| --- | --- |

| **Serialized Fields** |
| --- |

### field

```
double[][][] field
```

| **Class sim.field.grid.IntGrid2D extends AbstractGrid2D implements Serializable** | |
| --- | --- |

| **Serialized Fields** |
| --- |

### field

```
int[][] field
```

| **Class sim.field.grid.IntGrid3D extends AbstractGrid3D implements Serializable** | |
| --- | --- |

| **Serialized Fields** |
| --- |

### field

```
int[][][] field
```

| **Class sim.field.grid.ObjectGrid2D extends AbstractGrid2D implements Serializable** | |
| --- | --- |

| **Serialized Fields** |
| --- |

### field

```
java.lang.Object[][] field
```

| **Class sim.field.grid.ObjectGrid3D extends AbstractGrid3D implements Serializable** | |
| --- | --- |

| **Serialized Fields** |
| --- |

### field

```
java.lang.Object[][][] field
```

| **Class sim.field.grid.SparseGrid2D extends SparseField implements Serializable** | |
| --- | --- |

| **Serialized Fields** |
| --- |

### width

```
int width
```

---

### height

```
int height
```

---

### speedyMutableInt2D

```
MutableInt2D speedyMutableInt2D
```

| **Class sim.field.grid.SparseGrid3D extends SparseField implements Serializable** | |
| --- | --- |

| **Serialized Fields** |
| --- |

### width

```
int width
```

---

### height

```
int height
```

---

### length

```
int length
```

---

### speedyMutableInt3D

```
MutableInt3D speedyMutableInt3D
```

---

| **Package** **sim.field.network** |
| --- |

| **Class sim.field.network.Edge extends java.lang.Object implements Serializable** | |
| --- | --- |

| **Serialized Fields** |
| --- |

### owner

```
Network owner
```

---

### from

```
java.lang.Object from
```

:   The node from where the edge leaves

---

### to

```
java.lang.Object to
```

:   The node where the edge enters

---

### info

```
java.lang.Object info
```

:   Other information (maybe cost) associated with the edge

---

### indexFrom

```
int indexFrom
```

---

### indexTo

```
int indexTo
```

| **Class sim.field.network.Network extends java.lang.Object implements Serializable** | |
| --- | --- |

| **Serialized Fields** |
| --- |

### directed

```
boolean directed
```

---

### indexOutInHash

```
java.util.HashMap<K,V> indexOutInHash
```

:   Hashes Network.IndexOutIn structures by Node. These structures
    contain the incoming edges of the Node, its outgoing edges, and the index of
    the Node in the allNodes bag.

---

### allNodes

```
Bag allNodes
```

:   All the objects in the sparse field. For fast scans. Do not rely on this bag always being the same object.

---

### emptyBag

```
Bag emptyBag
```

| **Class sim.field.network.Network.IndexOutIn extends java.lang.Object implements Serializable** | |
| --- | --- |

| **Serialized Fields** |
| --- |

### index

```
int index
```

:   Index of the node in the allNodes bag

---

### out

```
Bag out
```

:   Bag containing outgoing edges of (leaving) the node

---

### in

```
Bag in
```

:   Bag containing incoming edges of (entering) the node

---

| **Package** **sim.portrayal** |
| --- |

| **Class sim.portrayal.FieldPortrayal2D extends FieldPortrayal implements Serializable** | |
| --- | --- |

| **Serialized Fields** |
| --- |

### simple

```
SimplePortrayal2D simple
```

---

### buffering

```
int buffering
```

---

### bufferingLock

```
java.lang.Object bufferingLock
```

| **Class sim.portrayal.Inspector extends javax.swing.JPanel implements Serializable** | |
| --- | --- |

| **Serialized Fields** |
| --- |

### \_volatile

```
boolean _volatile
```

| **Class sim.portrayal.SimpleInspector extends Inspector implements Serializable** | |
| --- | --- |

| **Serialized Fields** |
| --- |

### maxProperties

```
int maxProperties
```

---

### state

```
GUIState state
```

:   The GUIState of the simulation

---

### object

```
java.lang.Object object
```

:   The object being inspected

---

### propertyList

```
LabelledList propertyList
```

:   The property list displayed -- this may change at any time

---

### properties

```
Properties properties
```

:   The generated object properties -- this may change at any time

---

### members

```
PropertyField[] members
```

:   Each of the property fields in the property list, not all of which may exist at any time.

---

### name

```
java.lang.String name
```

:   The displayed name of the inspector

---

### start

```
int start
```

:   The current index of the topmost element

---

### count

```
int count
```

:   The number of items presently in the propertyList

---

### header

```
javax.swing.JPanel header
```

---

### numElements

```
javax.swing.JLabel numElements
```

---

### startField

```
javax.swing.Box startField
```

---

### updateButton

```
javax.swing.JButton updateButton
```

| **Class sim.portrayal.SimplePortrayal2D extends java.lang.Object implements Serializable** | |
| --- | --- |

---

| **Package** **sim.portrayal.continuous** |
| --- |

| **Class sim.portrayal.continuous.ContinuousPortrayal2D extends FieldPortrayal2D implements Serializable** | |
| --- | --- |

| **Serialized Fields** |
| --- |

### defaultPortrayal

```
SimplePortrayal2D defaultPortrayal
```

---

### frame

```
java.awt.Paint frame
```

---

### displayingToroidally

```
boolean displayingToroidally
```

---

### selectedWrappers

```
java.util.HashMap<K,V> selectedWrappers
```

---

| **Package** **sim.portrayal.grid** |
| --- |

| **Class sim.portrayal.grid.FastHexaObjectGridPortrayal2D extends HexaObjectGridPortrayal2D implements Serializable** | |
| --- | --- |

| **Serialized Fields** |
| --- |

### valueGridPortrayal

```
FastHexaValueGridPortrayal2D valueGridPortrayal
```

---

### grid

```
DoubleGrid2D grid
```

| **Class sim.portrayal.grid.FastHexaValueGridPortrayal2D extends HexaValueGridPortrayal2D implements Serializable** | |
| --- | --- |

| **Serialized Fields** |
| --- |

### buffer

```
java.awt.image.BufferedImage buffer
```

---

### raster

```
java.awt.image.WritableRaster raster
```

---

### dbuffer

```
java.awt.image.DataBufferInt dbuffer
```

---

### valueToPass

```
MutableDouble valueToPass
```

| **Class sim.portrayal.grid.FastObjectGridPortrayal2D extends ObjectGridPortrayal2D implements Serializable** | |
| --- | --- |

| **Serialized Fields** |
| --- |

### valueGridPortrayal

```
FastValueGridPortrayal2D valueGridPortrayal
```

---

### grid

```
DoubleGrid2D grid
```

| **Class sim.portrayal.grid.FastValueGridPortrayal2D extends ValueGridPortrayal2D implements Serializable** | |
| --- | --- |

| **Serialized Fields** |
| --- |

### buffer

```
java.awt.image.BufferedImage buffer
```

---

### raster

```
java.awt.image.WritableRaster raster
```

---

### data

```
int[] data
```

| **Class sim.portrayal.grid.HexaObjectGridPortrayal2D extends ObjectGridPortrayal2D implements Serializable** | |
| --- | --- |

| **Serialized Fields** |
| --- |

### xPoints

```
int[] xPoints
```

---

### yPoints

```
int[] yPoints
```

---

### xyC

```
double[] xyC
```

---

### xyC\_ul

```
double[] xyC_ul
```

---

### xyC\_up

```
double[] xyC_up
```

---

### xyC\_ur

```
double[] xyC_ur
```

| **Class sim.portrayal.grid.HexaSparseGridPortrayal2D extends SparseGridPortrayal2D implements Serializable** | |
| --- | --- |

| **Serialized Fields** |
| --- |

### xPoints

```
int[] xPoints
```

---

### yPoints

```
int[] yPoints
```

---

### xyC

```
double[] xyC
```

---

### xyC\_ul

```
double[] xyC_ul
```

---

### xyC\_up

```
double[] xyC_up
```

---

### xyC\_ur

```
double[] xyC_ur
```

| **Class sim.portrayal.grid.HexaValueGridPortrayal2D extends ValueGridPortrayal2D implements Serializable** | |
| --- | --- |

| **Serialized Fields** |
| --- |

### xPoints

```
int[] xPoints
```

---

### yPoints

```
int[] yPoints
```

---

### xPointsf

```
float[] xPointsf
```

---

### yPointsf

```
float[] yPointsf
```

---

### xyC

```
double[] xyC
```

---

### xyC\_ul

```
double[] xyC_ul
```

---

### xyC\_up

```
double[] xyC_up
```

---

### xyC\_ur

```
double[] xyC_ur
```

---

### generalPath

```
java.awt.geom.GeneralPath generalPath
```

| **Class sim.portrayal.grid.ObjectGridPortrayal2D extends FieldPortrayal2D implements Serializable** | |
| --- | --- |

| **Serialized Fields** |
| --- |

### defaultPortrayal

```
SimplePortrayal2D defaultPortrayal
```

---

### defaultNullPortrayal

```
SimplePortrayal2D defaultNullPortrayal
```

---

### locationToPass

```
MutableInt2D locationToPass
```

---

### SEARCH\_DISTANCE

```
int SEARCH_DISTANCE
```

---

### xPos

```
IntBag xPos
```

---

### yPos

```
IntBag yPos
```

---

### unknown

```
ObjectGridPortrayal2D.Message unknown
```

---

### selectedWrapper

```
LocationWrapper selectedWrapper
```

---

### selectedWrappers

```
java.util.HashMap<K,V> selectedWrappers
```

| **Class sim.portrayal.grid.SparseGridPortrayal2D extends FieldPortrayal2D implements Serializable** | |
| --- | --- |

| **Serialized Fields** |
| --- |

### policy

```
DrawPolicy policy
```

---

### defaultPortrayal

```
SimplePortrayal2D defaultPortrayal
```

---

### selectedWrappers

```
java.util.HashMap<K,V> selectedWrappers
```

| **Class sim.portrayal.grid.ValueGridPortrayal2D extends FieldPortrayal2D implements Serializable** | |
| --- | --- |

| **Serialized Fields** |
| --- |

### map

```
ColorMap map
```

---

### defaultPortrayal

```
SimplePortrayal2D defaultPortrayal
```

---

### valueName

```
java.lang.String valueName
```

---

### valueToPass

```
MutableDouble valueToPass
```

---

### locationToPass

```
MutableInt2D locationToPass
```

---

| **Package** **sim.portrayal.network** |
| --- |

| **Class sim.portrayal.network.NetworkPortrayal2D extends FieldPortrayal2D implements Serializable** | |
| --- | --- |

| **Serialized Fields** |
| --- |

### defaultPortrayal

```
SimpleEdgePortrayal2D defaultPortrayal
```

| **Class sim.portrayal.network.SimpleEdgePortrayal2D extends SimplePortrayal2D implements Serializable** | |
| --- | --- |

| **Serialized Fields** |
| --- |

### fromPaint

```
java.awt.Paint fromPaint
```

---

### toPaint

```
java.awt.Paint toPaint
```

---

### labelPaint

```
java.awt.Paint labelPaint
```

---

### labelFont

```
java.awt.Font labelFont
```

---

### scaledFont

```
java.awt.Font scaledFont
```

---

### labelScaling

```
int labelScaling
```

---

### scaling

```
int scaling
```

---

### baseWidth

```
double baseWidth
```

---

### shape

```
int shape
```

---

### adjustsThickness

```
boolean adjustsThickness
```

---

### preciseLine

```
java.awt.geom.Line2D.Double preciseLine
```

---

### precisePoly

```
java.awt.geom.GeneralPath precisePoly
```

---

### xPoints

```
int[] xPoints
```

---

### yPoints

```
int[] yPoints
```

---

| **Package** **sim.portrayal.simple** |
| --- |

| **Class sim.portrayal.simple.AdjustablePortrayal2D extends SimplePortrayal2D implements Serializable** | |
| --- | --- |

| **Serialized Fields** |
| --- |

### child

```
SimplePortrayal2D child
```

---

### adjusting

```
boolean adjusting
```

---

### adjustingObject

```
java.lang.Object adjustingObject
```

---

### adjustingInitialScale

```
double adjustingInitialScale
```

---

### adjustingInitialPosition

```
java.awt.geom.Point2D.Double adjustingInitialPosition
```

| **Class sim.portrayal.simple.CircledPortrayal2D extends OvalPortrayal2D implements Serializable** | |
| --- | --- |

| **Serialized Fields** |
| --- |

### child

```
SimplePortrayal2D child
```

---

### showCircle

```
boolean showCircle
```

:   Overrides all drawing.

---

### onlyCircleWhenSelected

```
boolean onlyCircleWhenSelected
```

| **Class sim.portrayal.simple.FacetedPortrayal2D extends SimplePortrayal2D implements Serializable** | |
| --- | --- |

| **Serialized Fields** |
| --- |

### children

```
SimplePortrayal2D[] children
```

---

### portrayAllChildren

```
boolean portrayAllChildren
```

---

### errorThrown

```
boolean errorThrown
```

| **Class sim.portrayal.simple.HexagonalPortrayal2D extends ShapePortrayal2D implements Serializable** | |
| --- | --- |

| **Class sim.portrayal.simple.ImagePortrayal2D extends RectanglePortrayal2D implements Serializable** | |
| --- | --- |

| **Serialized Fields** |
| --- |

### image

```
java.awt.Image image
```

---

### preciseTransform

```
java.awt.geom.AffineTransform preciseTransform
```

| **Class sim.portrayal.simple.LabelledPortrayal2D extends SimplePortrayal2D implements Serializable** | |
| --- | --- |

| **Serialized Fields** |
| --- |

### scalex

```
double scalex
```

:   The pre-scaling offset from the object's origin.

---

### scaley

```
double scaley
```

:   The pre-scaling offset from the object's origin.

---

### offsetx

```
double offsetx
```

:   The post-scaling offset from the object's origin.

---

### offsety

```
double offsety
```

:   The post-scaling offset from the object's origin.

---

### align

```
int align
```

:   One of ALIGN\_CENTER, ALIGN\_LEFT, or ALIGN\_RIGHT

---

### font

```
java.awt.Font font
```

:   The font of the text.

---

### paint

```
java.awt.Paint paint
```

:   The Paint or Color of the text

---

### label

```
java.lang.String label
```

---

### child

```
SimplePortrayal2D child
```

---

### showLabel

```
boolean showLabel
```

:   Overrides all drawing.

---

### onlyLabelWhenSelected

```
boolean onlyLabelWhenSelected
```

---

### scaledFont

```
java.awt.Font scaledFont
```

---

### labelScaling

```
int labelScaling
```

| **Class sim.portrayal.simple.MovablePortrayal2D extends SimplePortrayal2D implements Serializable** | |
| --- | --- |

| **Serialized Fields** |
| --- |

### child

```
SimplePortrayal2D child
```

---

### originalMousePosition

```
java.awt.geom.Point2D originalMousePosition
```

---

### originalObjectPosition

```
java.awt.geom.Point2D originalObjectPosition
```

| **Class sim.portrayal.simple.OrientedPortrayal2D extends SimplePortrayal2D implements Serializable** | |
| --- | --- |

| **Serialized Fields** |
| --- |

### shape

```
int shape
```

:   The type of the oriented shape

---

### scale

```
double scale
```

:   The pre-scaling length

---

### offset

```
int offset
```

:   The post-scaling length offset

---

### paint

```
java.awt.Paint paint
```

:   The Paint or Color of the line

---

### child

```
SimplePortrayal2D child
```

---

### showOrientation

```
boolean showOrientation
```

:   Overrides all drawing.

---

### drawFilled

```
boolean drawFilled
```

---

### path

```
java.awt.Shape path
```

---

### onlyDrawWhenSelected

```
boolean onlyDrawWhenSelected
```

---

### simplePolygonX

```
int[] simplePolygonX
```

---

### simplePolygonY

```
int[] simplePolygonY
```

---

### simplePolygonXd

```
double[] simplePolygonXd
```

---

### simplePolygonYd

```
double[] simplePolygonYd
```

---

### lastLength

```
double lastLength
```

---

### transform

```
java.awt.geom.AffineTransform transform
```

---

### stroke

```
java.awt.Stroke stroke
```

---

### orientationHittable

```
boolean orientationHittable
```

| **Class sim.portrayal.simple.OvalPortrayal2D extends SimplePortrayal2D implements Serializable** | |
| --- | --- |

| **Serialized Fields** |
| --- |

### paint

```
java.awt.Paint paint
```

---

### scale

```
double scale
```

---

### filled

```
boolean filled
```

---

### offset

```
double offset
```

| **Class sim.portrayal.simple.RectanglePortrayal2D extends SimplePortrayal2D implements Serializable** | |
| --- | --- |

| **Serialized Fields** |
| --- |

### paint

```
java.awt.Paint paint
```

---

### scale

```
double scale
```

---

### filled

```
boolean filled
```

| **Class sim.portrayal.simple.ShapePortrayal2D extends SimplePortrayal2D implements Serializable** | |
| --- | --- |

| **Serialized Fields** |
| --- |

### paint

```
java.awt.Paint paint
```

---

### scale

```
double scale
```

---

### shape

```
java.awt.Shape shape
```

---

### stroke

```
java.awt.Stroke stroke
```

---

### filled

```
boolean filled
```

---

### transform

```
java.awt.geom.AffineTransform transform
```

---

### xPoints

```
double[] xPoints
```

---

### yPoints

```
double[] yPoints
```

---

### scaledXPoints

```
double[] scaledXPoints
```

---

### scaledYPoints

```
double[] scaledYPoints
```

---

### translatedXPoints

```
int[] translatedXPoints
```

---

### translatedYPoints

```
int[] translatedYPoints
```

---

### scaling

```
double scaling
```

---

### bufferedWidth

```
double bufferedWidth
```

---

### bufferedHeight

```
double bufferedHeight
```

---

### bufferedShape

```
java.awt.Shape bufferedShape
```

| **Class sim.portrayal.simple.TrailedPortrayal2D extends SimplePortrayal2D implements Serializable** | |
| --- | --- |

| **Serialized Fields** |
| --- |

### isSelected

```
boolean isSelected
```

---

### onlyGrowTrailWhenSelected

```
boolean onlyGrowTrailWhenSelected
```

---

### onlyShowTrailWhenSelected

```
boolean onlyShowTrailWhenSelected
```

---

### places

```
java.util.LinkedList<E> places
```

---

### defaultMap

```
SimpleColorMap defaultMap
```

---

### child

```
SimplePortrayal2D child
```

:   The Child portrayal of this portrayal: a SimplePortrayal2D used solely for determining hit testing.

---

### trail

```
SimplePortrayal2D trail
```

:   The SimplePortrayal2D used to draw line segments in the trail.

---

### length

```
double length
```

---

### state

```
GUIState state
```

---

### fieldPortrayal

```
FieldPortrayal2D fieldPortrayal
```

---

### maximumJump

```
double maximumJump
```

---

### lastObj

```
java.lang.Object lastObj
```

---

### selectedObj

```
java.lang.Object selectedObj
```

---

### locked

```
boolean locked
```

| **Class sim.portrayal.simple.TransformedPortrayal2D extends SimplePortrayal2D implements Serializable** | |
| --- | --- |

| **Serialized Fields** |
| --- |

### child

```
SimplePortrayal2D child
```

---

### transform

```
java.awt.geom.AffineTransform transform
```

| **Class sim.portrayal.simple.ValuePortrayal2D extends RectanglePortrayal2D implements Serializable** | |
| --- | --- |

| **Serialized Fields** |
| --- |

### level

```
double level
```

---

### isTransparent

```
boolean isTransparent
```

---

| **Package** **sim.portrayal3d** |
| --- |

| **Class sim.portrayal3d.FieldPortrayal3D extends FieldPortrayal implements Serializable** | |
| --- | --- |

| **Serialized Fields** |
| --- |

### internalTransform

```
javax.media.j3d.Transform3D internalTransform
```

---

### updateInternalTransform

```
boolean updateInternalTransform
```

---

### display

```
Display3D display
```

---

### defaultPortrayal

```
SimplePortrayal3D defaultPortrayal
```

:   White sphere as default portrayal
    for objects that do not have any other specified to them
    Note that it is not final, so it can be replaced.

| **Class sim.portrayal3d.SimplePortrayal3D extends java.lang.Object implements Serializable** | |
| --- | --- |

| **Serialized Fields** |
| --- |

### fieldPortrayal

```
FieldPortrayal3D fieldPortrayal
```

---

### display

```
Display3D display
```

---

### selectedObjects

```
java.util.HashMap<K,V> selectedObjects
```

| **Class sim.portrayal3d.SparseFieldPortrayal3D extends FieldPortrayal3D implements Serializable** | |
| --- | --- |

---

| **Package** **sim.portrayal3d.continuous** |
| --- |

| **Class sim.portrayal3d.continuous.ContinuousPortrayal3D extends SparseFieldPortrayal3D implements Serializable** | |
| --- | --- |

---

| **Package** **sim.portrayal3d.grid** |
| --- |

| **Class sim.portrayal3d.grid.ObjectGridPortrayal3D extends FieldPortrayal3D implements Serializable** | |
| --- | --- |

| **Serialized Fields** |
| --- |

### SEARCH\_DISTANCE

```
int SEARCH_DISTANCE
```

---

### bagsize

```
int bagsize
```

---

### xPos

```
IntBag xPos
```

---

### yPos

```
IntBag yPos
```

---

### zPos

```
IntBag zPos
```

---

### unknown

```
ObjectGridPortrayal2D.Message unknown
```

| **Class sim.portrayal3d.grid.SparseGrid2DPortrayal3D extends SparseGridPortrayal3D implements Serializable** | |
| --- | --- |

| **Serialized Fields** |
| --- |

### zScale

```
double zScale
```

| **Class sim.portrayal3d.grid.SparseGridPortrayal3D extends SparseFieldPortrayal3D implements Serializable** | |
| --- | --- |

| **Class sim.portrayal3d.grid.ValueGrid2DPortrayal3D extends FieldPortrayal3D implements Serializable** | |
| --- | --- |

| **Serialized Fields** |
| --- |

### image

```
java.awt.Image image
```

---

### transparency

```
double transparency
```

:   Non-image transparency: 1.0f is fully opaque, 0.0f is fully transparent.

---

### mPolyAttributes

```
javax.media.j3d.PolygonAttributes mPolyAttributes
```

---

### useTriangles

```
boolean useTriangles
```

---

### valueName

```
java.lang.String valueName
```

---

### defaultPortrayal

```
QuadPortrayal defaultPortrayal
```

---

### coords

```
float[] coords
```

---

### colors

```
float[] colors
```

---

### resetField

```
boolean resetField
```

---

### tmpVect

```
javax.vecmath.Vector3d tmpVect
```

:   tmp Vector3d

---

### tmpLocalT

```
javax.media.j3d.Transform3D tmpLocalT
```

:   tmp Transform3D
    it is reused, since the TGs are copying it internally

---

### tmpGCI

```
ValueGridCellInfo tmpGCI
```

:   allocated in portray, and heavily reused in create/update model
    to avoid "new"s

| **Class sim.portrayal3d.grid.ValueGridPortrayal3D extends FieldPortrayal3D implements Serializable** | |
| --- | --- |

| **Serialized Fields** |
| --- |

### valueName

```
java.lang.String valueName
```

---

### scale

```
double scale
```

---

### map

```
ColorMap map
```

---

### width

```
int width
```

---

### height

```
int height
```

---

### length

```
int length
```

---

### valueToPass

```
MutableDouble valueToPass
```

---

### dirtyScale

```
boolean dirtyScale
```

---

### defaultPortrayal

```
ValuePortrayal3D defaultPortrayal
```

---

| **Package** **sim.portrayal3d.grid.quad** |
| --- |

| **Class sim.portrayal3d.grid.quad.MeshPortrayal extends QuadPortrayal implements Serializable** | |
| --- | --- |

| **Serialized Fields** |
| --- |

### tmpCoords

```
float[] tmpCoords
```

---

### tmpColor

```
float[] tmpColor
```

| **Class sim.portrayal3d.grid.quad.QuadPortrayal extends java.lang.Object implements Serializable** | |
| --- | --- |

| **Serialized Fields** |
| --- |

### zScale

```
double zScale
```

:   How much we move the quad up or down for a given value.

---

### colorDispenser

```
ColorMap colorDispenser
```

:   Our color map for values

| **Class sim.portrayal3d.grid.quad.TilePortrayal extends QuadPortrayal implements Serializable** | |
| --- | --- |

| **Serialized Fields** |
| --- |

### tmpCoords

```
float[] tmpCoords
```

---

### tmpColor

```
float[] tmpColor
```

---

| **Package** **sim.portrayal3d.simple** |
| --- |

| **Class sim.portrayal3d.simple.AxesPortrayal3D extends SimplePortrayal3D implements Serializable** | |
| --- | --- |

| **Serialized Fields** |
| --- |

### arrowRadius

```
double arrowRadius
```

---

### mLetters

```
boolean mLetters
```

| **Class sim.portrayal3d.simple.BranchGroupPortrayal3D extends PrimitivePortrayal3D implements Serializable** | |
| --- | --- |

| **Class sim.portrayal3d.simple.CircledPortrayal3D extends SimplePortrayal3D implements Serializable** | |
| --- | --- |

| **Serialized Fields** |
| --- |

### scale

```
double scale
```

---

### appearance

```
javax.media.j3d.Appearance appearance
```

---

### child

```
SimplePortrayal3D child
```

---

### showCircle

```
boolean showCircle
```

:   Overrides all drawing.

---

### onlyCircleWhenSelected

```
boolean onlyCircleWhenSelected
```

| **Class sim.portrayal3d.simple.ConePortrayal3D extends PrimitivePortrayal3D implements Serializable** | |
| --- | --- |

| **Class sim.portrayal3d.simple.CubePortrayal3D extends SimplePortrayal3D implements Serializable** | |
| --- | --- |

| **Serialized Fields** |
| --- |

### scale

```
double scale
```

---

### appearance

```
javax.media.j3d.Appearance appearance
```

---

### generateNormals

```
boolean generateNormals
```

---

### generateTextureCoordinates

```
boolean generateTextureCoordinates
```

---

### scaledVerts

```
float[] scaledVerts
```

| **Class sim.portrayal3d.simple.CylinderPortrayal3D extends PrimitivePortrayal3D implements Serializable** | |
| --- | --- |

| **Class sim.portrayal3d.simple.ImagePortrayal3D extends SimplePortrayal3D implements Serializable** | |
| --- | --- |

| **Serialized Fields** |
| --- |

### shape

```
javax.media.j3d.Shape3D shape
```

| **Class sim.portrayal3d.simple.LabelledPortrayal3D extends SimplePortrayal3D implements Serializable** | |
| --- | --- |

| **Serialized Fields** |
| --- |

### labelScale

```
double labelScale
```

---

### color

```
java.awt.Color color
```

---

### offset

```
javax.media.j3d.Transform3D offset
```

---

### font

```
java.awt.Font font
```

---

### font3D

```
javax.media.j3d.Font3D font3D
```

---

### child

```
SimplePortrayal3D child
```

---

### label

```
java.lang.String label
```

---

### showLabel

```
boolean showLabel
```

:   Overrides all drawing.

---

### onlyLabelWhenSelected

```
boolean onlyLabelWhenSelected
```

| **Class sim.portrayal3d.simple.LightPortrayal3D extends SimplePortrayal3D implements Serializable** | |
| --- | --- |

| **Serialized Fields** |
| --- |

### light

```
javax.media.j3d.Light light
```

| **Class sim.portrayal3d.simple.PrimitivePortrayal3D extends SimplePortrayal3D implements Serializable** | |
| --- | --- |

| **Serialized Fields** |
| --- |

### transform

```
javax.media.j3d.Transform3D transform
```

---

### appearance

```
javax.media.j3d.Appearance appearance
```

---

### group

```
javax.media.j3d.Node group
```

:   This is cloned to create the model. Typically this group holds a single element,
    either a Shape3D object or a Primitive of some sort. The model, which is also a
    TransformGroup, will then hold onto this object (or more properly, a clone). Note
    that the outer model TransformGroup (called j3dModel throughout this code) is not owned
    by us once we create it. So if we want to rotate or scale the Shape3D or Primitive,
    we do it by transforming 'group' instead upon creation.

---

### pickable

```
boolean pickable
```

| **Class sim.portrayal3d.simple.Shape3DPortrayal3D extends PrimitivePortrayal3D implements Serializable** | |
| --- | --- |

| **Class sim.portrayal3d.simple.SharedPortrayal3D extends SimplePortrayal3D implements Serializable** | |
| --- | --- |

| **Serialized Fields** |
| --- |

### child

```
SimplePortrayal3D child
```

---

### group

```
javax.media.j3d.SharedGroup group
```

| **Class sim.portrayal3d.simple.SpherePortrayal3D extends PrimitivePortrayal3D implements Serializable** | |
| --- | --- |

| **Class sim.portrayal3d.simple.TransformedPortrayal3D extends SimplePortrayal3D implements Serializable** | |
| --- | --- |

| **Serialized Fields** |
| --- |

### child

```
SimplePortrayal3D child
```

---

### internalTransform

```
javax.media.j3d.Transform3D internalTransform
```

---

### updateInternalTransform

```
boolean updateInternalTransform
```

| **Class sim.portrayal3d.simple.ValuePortrayal3D extends Shape3DPortrayal3D implements Serializable** | |
| --- | --- |

| **Serialized Fields** |
| --- |

### mPolyAttributes

```
javax.media.j3d.PolygonAttributes mPolyAttributes
```

| **Class sim.portrayal3d.simple.WireFrameBoxPortrayal3D extends SimplePortrayal3D implements Serializable** | |
| --- | --- |

| **Serialized Fields** |
| --- |

### appearance

```
javax.media.j3d.Appearance appearance
```

---

### scaledVerts

```
float[] scaledVerts
```

---

| **Package** **sim.util** |
| --- |

| **Class sim.util.Bag extends java.lang.Object implements Serializable** | |
| --- | --- |

| **Serialized Fields** |
| --- |

### objs

```
java.lang.Object[] objs
```

---

### numObjs

```
int numObjs
```

| **Class sim.util.CausedRuntimeException extends java.lang.RuntimeException implements Serializable** | |
| --- | --- |

| **Serialized Fields** |
| --- |

### target

```
java.lang.Throwable target
```

:   **Deprecated.**

---

### message

```
java.lang.String message
```

:   **Deprecated.**

| **Class sim.util.CollectionProperties extends Properties implements Serializable** | |
| --- | --- |

| **Serialized Fields** |
| --- |

### collection

```
java.util.Collection<E> collection
```

---

### map

```
java.util.Map<K,V> map
```

---

### indexed

```
Indexed indexed
```

---

### isVolatile

```
boolean isVolatile
```

| **Class sim.util.Double2D extends java.lang.Object implements Serializable** | |
| --- | --- |

| **Serialized Fields** |
| --- |

### x

```
double x
```

---

### y

```
double y
```

| **Class sim.util.Double3D extends java.lang.Object implements Serializable** | |
| --- | --- |

| **Serialized Fields** |
| --- |

### x

```
double x
```

---

### y

```
double y
```

---

### z

```
double z
```

| **Class sim.util.DoubleBag extends java.lang.Object implements Serializable** | |
| --- | --- |

| **Serialized Fields** |
| --- |

### objs

```
double[] objs
```

---

### numObjs

```
int numObjs
```

| **Class sim.util.Heap extends java.lang.Object implements Serializable** | |
| --- | --- |

| **Serialized Fields** |
| --- |

### keys

```
java.lang.Comparable<T>[] keys
```

---

### objects

```
java.lang.Object[] objects
```

---

### numElem

```
int numElem
```

| **Class sim.util.Int2D extends java.lang.Object implements Serializable** | |
| --- | --- |

| **Serialized Fields** |
| --- |

### x

```
int x
```

---

### y

```
int y
```

| **Class sim.util.Int3D extends java.lang.Object implements Serializable** | |
| --- | --- |

| **Serialized Fields** |
| --- |

### x

```
int x
```

---

### y

```
int y
```

---

### z

```
int z
```

| **Class sim.util.IntBag extends java.lang.Object implements Serializable** | |
| --- | --- |

| **Serialized Fields** |
| --- |

### objs

```
int[] objs
```

---

### numObjs

```
int numObjs
```

| **Class sim.util.MutableDouble extends java.lang.Number implements Serializable** | |
| --- | --- |

| **Serialized Fields** |
| --- |

### val

```
double val
```

| **Class sim.util.MutableDouble2D extends java.lang.Object implements Serializable** | |
| --- | --- |

| **Serialized Fields** |
| --- |

### x

```
double x
```

---

### y

```
double y
```

| **Class sim.util.MutableDouble3D extends java.lang.Object implements Serializable** | |
| --- | --- |

| **Serialized Fields** |
| --- |

### x

```
double x
```

---

### y

```
double y
```

---

### z

```
double z
```

| **Class sim.util.MutableInt2D extends java.lang.Object implements Serializable** | |
| --- | --- |

| **Serialized Fields** |
| --- |

### x

```
int x
```

---

### y

```
int y
```

| **Class sim.util.MutableInt3D extends java.lang.Object implements Serializable** | |
| --- | --- |

| **Serialized Fields** |
| --- |

### x

```
int x
```

---

### y

```
int y
```

---

### z

```
int z
```

| **Class sim.util.Properties extends java.lang.Object implements Serializable** | |
| --- | --- |

| **Serialized Fields** |
| --- |

### object

```
java.lang.Object object
```

| **Class sim.util.SimpleProperties extends Properties implements Serializable** | |
| --- | --- |

| **Serialized Fields** |
| --- |

### getMethods

```
java.util.ArrayList<E> getMethods
```

---

### setMethods

```
java.util.ArrayList<E> setMethods
```

---

### domMethods

```
java.util.ArrayList<E> domMethods
```

---

### hideMethods

```
java.util.ArrayList<E> hideMethods
```

---

### auxillary

```
Properties auxillary
```

---

| **Package** **sim.util.gui** |
| --- |

| **Class sim.util.gui.AbstractScrollable extends javax.swing.JPanel implements Serializable** | |
| --- | --- |

| **Class sim.util.gui.ColorWell extends javax.swing.JPanel implements Serializable** | |
| --- | --- |

| **Serialized Fields** |
| --- |

### color

```
java.awt.Color color
```

| **Class sim.util.gui.DisclosurePanel extends javax.swing.JPanel implements Serializable** | |
| --- | --- |

| **Serialized Fields** |
| --- |

### disclosureToggle

```
javax.swing.JToggleButton disclosureToggle
```

---

### abridgedComponent

```
java.awt.Component abridgedComponent
```

---

### disclosedComponent

```
java.awt.Component disclosedComponent
```

---

### disclosed

```
boolean disclosed
```

| **Class sim.util.gui.HTMLBrowser extends javax.swing.JPanel implements Serializable** | |
| --- | --- |

| **Serialized Fields** |
| --- |

### stack

```
java.util.Stack<E> stack
```

---

### infoPane

```
javax.swing.JEditorPane infoPane
```

---

### scroll

```
javax.swing.JScrollPane scroll
```

| **Class sim.util.gui.LabelledList extends javax.swing.JPanel implements Serializable** | |
| --- | --- |

| **Serialized Fields** |
| --- |

### consolePanel

```
javax.swing.JPanel consolePanel
```

---

### gridbag

```
java.awt.GridBagLayout gridbag
```

---

### gbc

```
java.awt.GridBagConstraints gbc
```

---

### y

```
int y
```

| **Class sim.util.gui.MiniHistogram extends javax.swing.JComponent implements Serializable** | |
| --- | --- |

| **Serialized Fields** |
| --- |

### buckets

```
double[] buckets
```

---

### labels

```
java.lang.String[] labels
```

---

### motionAdapter

```
java.awt.event.MouseMotionAdapter motionAdapter
```

---

### adapter

```
java.awt.event.MouseAdapter adapter
```

| **Class sim.util.gui.NumberTextField extends javax.swing.JComponent implements Serializable** | |
| --- | --- |

| **Serialized Fields** |
| --- |

### valField

```
javax.swing.JTextField valField
```

---

### downButton

```
javax.swing.JButton downButton
```

---

### upButton

```
javax.swing.JButton upButton
```

---

### bellyButton

```
javax.swing.JButton bellyButton
```

---

### fieldLabel

```
javax.swing.JLabel fieldLabel
```

---

### initialValue

```
double initialValue
```

---

### multiply

```
double multiply
```

---

### add

```
double add
```

---

### currentValue

```
double currentValue
```

---

### defaultColor

```
java.awt.Color defaultColor
```

---

### editedColor

```
java.awt.Color editedColor
```

---

### edited

```
boolean edited
```

---

### listener

```
java.awt.event.KeyListener listener
```

---

### focusAdapter

```
java.awt.event.FocusAdapter focusAdapter
```

| **Class sim.util.gui.PropertyField extends javax.swing.JComponent implements Serializable** | |
| --- | --- |

| **Serialized Fields** |
| --- |

### list

```
javax.swing.JComboBox list
```

---

### valField

```
javax.swing.JTextField valField
```

---

### checkField

```
javax.swing.JCheckBox checkField
```

---

### viewButton

```
javax.swing.JButton viewButton
```

---

### viewLabel

```
javax.swing.JLabel viewLabel
```

---

### optionalLabel

```
javax.swing.JLabel optionalLabel
```

---

### slider

```
javax.swing.JSlider slider
```

---

### sliderFormatter

```
java.text.DecimalFormat sliderFormatter
```

---

### valFieldBorder

```
javax.swing.border.Border valFieldBorder
```

---

### emptyBorder

```
javax.swing.border.Border emptyBorder
```

---

### currentValue

```
java.lang.String currentValue
```

---

### isReadWrite

```
boolean isReadWrite
```

---

### domain

```
java.lang.Object domain
```

---

### displayState

```
int displayState
```

---

### defaultColor

```
java.awt.Color defaultColor
```

---

### editedColor

```
java.awt.Color editedColor
```

---

### edited

```
boolean edited
```

---

### listener

```
java.awt.event.KeyListener listener
```

---

### checkListener

```
java.awt.event.ActionListener checkListener
```

---

### viewButtonListener

```
java.awt.event.ActionListener viewButtonListener
```

---

### focusAdapter

```
java.awt.event.FocusAdapter focusAdapter
```

---

### sliding

```
boolean sliding
```

---

### sliderListener

```
javax.swing.event.ChangeListener sliderListener
```

---

### listListener

```
java.awt.event.ActionListener listListener
```

---

### settingList

```
boolean settingList
```

| **Class sim.util.gui.WordWrap extends java.lang.Object implements Serializable** | |
| --- | --- |

---

| **Package** **sim.util.media** |
| --- |

| **Class sim.util.media.MovieEncoder extends java.lang.Object implements Serializable** | |
| --- | --- |

| **Serialized Fields** |
| --- |

### started

```
boolean started
```

---

### stopped

```
boolean stopped
```

---

### width

```
int width
```

---

### height

```
int height
```

---

### type

```
int type
```

---

### frameRate

```
float frameRate
```

---

### processor

```
Processor processor
```

---

### source

```
sim.util.media.MovieEncoderDataSource source
```

---

### sink

```
DataSink sink
```

---

### file

```
java.io.File file
```

---

### encodeFormat

```
Format encodeFormat
```

---

### waitSync

```
java.lang.Object waitSync
```

---

### stateTransitionOK

```
boolean stateTransitionOK
```

---

### waitFileSync

```
java.lang.Object waitFileSync
```

---

### fileDone

```
boolean fileDone
```

---

### fileSuccess

```
boolean fileSuccess
```

---

| **Package** **sim.util.media.chart** |
| --- |

| **Class sim.util.media.chart.ChartGenerator extends javax.swing.JPanel implements Serializable** | |
| --- | --- |

| **Serialized Fields** |
| --- |

### globalAttributes

```
javax.swing.Box globalAttributes
```

:   A holder for global attributes components

---

### seriesAttributes

```
javax.swing.Box seriesAttributes
```

:   A holder for series attributes components

---

### chart

```
JFreeChart chart
```

:   The chart

---

### chartPanel

```
ChartPanel chartPanel
```

:   The panel which holds and draws the chart

---

### chartHolder

```
javax.swing.JScrollPane chartHolder
```

:   The JScrollPane which holdw the ChartPanel

---

### frame

```
javax.swing.JFrame frame
```

---

### titleField

```
PropertyField titleField
```

:   The global attributes chart title field.

---

### xLabel

```
PropertyField xLabel
```

:   The global attributes domain axis field.

---

### yLabel

```
PropertyField yLabel
```

:   The global attributes range axis field.

---

### yLog

```
javax.swing.JCheckBox yLog
```

:   The global attributes logarithmic range axis check box.

---

### xLog

```
javax.swing.JCheckBox xLog
```

:   The global attributes logarithmic domain axis check box.

---

### movieButton

```
javax.swing.JButton movieButton
```

---

### buffer

```
java.awt.image.BufferedImage buffer
```

---

### movieMaker

```
MovieMaker movieMaker
```

---

### oldKey

```
long oldKey
```

---

### timer

```
java.lang.Thread timer
```

| **Class sim.util.media.chart.HistogramGenerator extends ChartGenerator implements Serializable** | |
| --- | --- |

| **Serialized Fields** |
| --- |

### histogramType

```
HistogramType histogramType
```

| **Class sim.util.media.chart.HistogramSeriesAttributes extends SeriesAttributes implements Serializable** | |
| --- | --- |

| **Serialized Fields** |
| --- |

### values

```
double[] values
```

---

### thickness

```
float thickness
```

:   Border thickness

---

### thicknessField

```
NumberTextField thicknessField
```

---

### fillColor

```
java.awt.Color fillColor
```

:   The color of the histogram bar.

---

### fillColorWell

```
ColorWell fillColorWell
```

---

### strokeColor

```
java.awt.Color strokeColor
```

:   The color of the histogram bar border.

---

### strokeColorWell

```
ColorWell strokeColorWell
```

---

### fillOpacity

```
double fillOpacity
```

:   The opacity of the histogram bar. Sadly this must be separate than the color because
    Sun doesn't have a proper color selector.

---

### fillOpacityField

```
NumberTextField fillOpacityField
```

---

### lineOpacity

```
double lineOpacity
```

:   The opacity of the histogram bar border. Sadly this must be separate than the color because
    Sun doesn't have a proper color selector.

---

### lineOpacityField

```
NumberTextField lineOpacityField
```

---

### numBinsField

```
NumberTextField numBinsField
```

---

### numBins

```
int numBins
```

| **Class sim.util.media.chart.ScatterPlotGenerator extends ChartGenerator implements Serializable** | |
| --- | --- |

| **Class sim.util.media.chart.ScatterPlotSeriesAttributes extends SeriesAttributes implements Serializable** | |
| --- | --- |

| **Serialized Fields** |
| --- |

### values

```
double[][] values
```

---

### color

```
java.awt.Color color
```

---

### colorWell

```
ColorWell colorWell
```

---

### opacity

```
double opacity
```

---

### opacityField

```
NumberTextField opacityField
```

---

### shapeNum

```
int shapeNum
```

---

### shape

```
java.awt.Shape shape
```

---

### shapeList

```
javax.swing.JComboBox shapeList
```

| **Class sim.util.media.chart.SeriesAttributes extends LabelledList implements Serializable** | |
| --- | --- |

| **Serialized Fields** |
| --- |

### stoppable

```
SeriesChangeListener stoppable
```

---

### seriesIndex

```
int seriesIndex
```

:   The index of the series that this SeriesAttributes is responsible for.

---

### generator

```
ChartGenerator generator
```

:   The ChartGenerator which holds the series that this SeriesAttributes is responsible for.

---

### manipulators

```
javax.swing.Box manipulators
```

| **Class sim.util.media.chart.TimeSeriesAttributes extends SeriesAttributes implements Serializable** | |
| --- | --- |

| **Serialized Fields** |
| --- |

### stretch

```
float stretch
```

:   How much we should stretch the dashPatterns listed above. 1.0 is normal.

---

### stretchField

```
NumberTextField stretchField
```

---

### thickness

```
float thickness
```

:   Line thickness.

---

### thicknessField

```
NumberTextField thicknessField
```

---

### dashPattern

```
float[] dashPattern
```

:   Line dash pattern (one of the dashPatterns above).

---

### dashPatternList

```
javax.swing.JComboBox dashPatternList
```

---

### strokeColor

```
java.awt.Color strokeColor
```

:   Line color.

---

### strokeColorWell

```
ColorWell strokeColorWell
```

---

### series

```
XYSeries series
```

:   The time series in question.

| **Class sim.util.media.chart.TimeSeriesChartGenerator extends ChartGenerator implements Serializable** | |
| --- | --- |

| **Serialized Fields** |
| --- |

### useCullingCheckBox

```
javax.swing.JCheckBox useCullingCheckBox
```

---

### maxPointsPerSeriesTextField

```
NumberTextField maxPointsPerSeriesTextField
```

---

### dataCuller

```
DataCuller dataCuller
```

---


|  |  |  |  |  |  |  |  |  |  |
| --- | --- | --- | --- | --- | --- | --- | --- | --- | --- |
| |  |  |  |  |  |  |  | | --- | --- | --- | --- | --- | --- | --- | | **Overview** | Package | Class | **Tree** | **Deprecated** | **Index** | **Help** | | |  |
| PREV   NEXT | **FRAMES**    **NO FRAMES**     **All Classes** |


---
