# Supplementary material for: Out of the net: An agent-based model to study human movements influence on local-scale malaria transmission
Source: PLoS One. 2018 Mar 6;13(3):e0193493. doi: 10.1371/journal.pone.0193493 (PMC5839546; doi:10.1371/journal.pone.0193493)
Supplement: S2 File — (ZIP) [file pone.0193493.s002.zip › S2/docs/howto.html]

How-Tos


# How-Tos

These are short examples of how to do things in MASON you may have been wondering about. For more involved examples of extending MASON to use parameterization, etc., see the online Web Tutorials.

- How to Schedule Multiple Methods to be Called on the Same Agent- How to Create a Field Portrayal Inspector- How to Draw Directly on the Display2D (or)  
      How to Create a Background Image for the Display2D- How to Make an Applet- How to Parallelize a MASON application- How to Schedule Things Weakly- How to Delete Things from the Schedule- How to Delete Things from a Field- How to Add a Slider or Pop-up Menu to an Inspected Property- How to Draw Faster- How to Scale or Translate a 2D Field Portrayal's depiction on-screen- How to Create a Plug-In Inspector for a Single Property- How to Create an Inspector Holding Other Inspectors- How to Add a Chart Programmatically- How to Hide the Console- How to Show that an Object Has Been "Selected" by the Mouse- How to Move an Object in 2D by Dragging It with the Mouse- How to Change the Look of a Provided SimplePortrayal2D Dynamically Based on the Object Status- How to Draw "Trails" or "Mouse Tails" Dragging Behind Objects

## How to Schedule Multiple Methods to be Called on the Same Agent

People have on occasion been mystified by the fact that MASON's Steppable class only has the single class step(*SimState*). If an agent subclasses from Steppable, how can we schedule two different methods in the agent?

Tutorial 5 showed an example of how to do this. But before we go into it in more depth, it's worth explaining some history. The original Swarm multiagent simulation system was written not in Java but in Objective-C, a preprocessor language for C which added Smalltalk-like messagepassing and a simple object-oriented model. Strangely, Swarm wasn't targeted for the only major system which ever ran Objective-C, namely NeXTSTEP (later MacOS X).

Objective-C allowed you to send *messages* to *objects*. This was essentially the same thing as calling method on objects in Java, except that in Objective-C, you could make up the message at run-time and send it to the object. If the object had a method of that name, it'd get called. If it didn't, then if the object had a method called forward(*message*), it'd be called. If there was no method called forward, a runtime error would be issued.

This scheme allowed you to schedule events on Swarm's schedule by just providing a message (essentially a string representing the method name) and an object to send the message to. But in Java you can't make up the method calls at runtime except through reflection. The compiler requires you to specify the method at compile time for efficiency's sake.

As it attempted to reimplement Swarm in Java, RePast's approach was to copy Swarm's scheduler, including replicating the message-passing notion. Thus users can schedule a method, by string name, in combination with an object to call the method on. Using reflection, the method is looked up at runtime and called. There are several problems with this. First, depending on the virtual machine used, this can be fairly inefficient. Second and more importantly, it allows for runtime errors when the method doesn't exist. This can be profoundly confusing to a Java coder. And third, it's very much against Java style: it violates all sorts of contracts and guarantees.

Violating Java style is *perfectly acceptable* if the occasion calls for it. However as it turns out there is a "Java" way to schedule methods to be called: using an anonymous class. Let's say your object myObject has the methods foo(), bar(), and baz() and you wish them all to be scheduled for the next time step. You can do this:

```
final MyObjectClass obj = myObject;
schedule.scheduleOnce(new Steppable() { public void step(SimState state) { obj.foo(); } });
schedule.scheduleOnce(new Steppable() { public void step(SimState state) { obj.bar(); } });
schedule.scheduleOnce(new Steppable() { public void step(SimState state) { obj.baz(); } });
```

You only need to make obj final if myObject is a local variable. If it's an instance variable, you can just say myObject.foo() and skip the obj = ... line entirely.

If your method foo takes a SimState, you could have written:

```
final MyObjectClass obj = myObject;
schedule.scheduleOnce(new Steppable() { public void step(SimState state) { obj.foo(state); } });
```

You could even create an event-creating method (note that thingToPrint is final):

```
public boolean scheduleAPrinter(final String thingToPrint)
    {
    return schedule.scheduleOnce(new Steppable() { 
        public void step(SimState state) { System.out.println(thingToPrint); } });
    }
```

(for fun, try modifying the above into an accumulator generator, where the accumulators print new values each time they're pulsed by the schedule. Hint: final double[] count = new double[]{i};).

Anyway, using anonymous classes is clean Java and it is fast. And it provides the full power of Lisp's closures to boot, something a message-target mechanism just can't do. This gets to the heart of why Steppable has only one method: because beyond subclassing Steppable to create one-method agents, we just make Anonymous Steppables that call our multi-method agent as we see fit. Steppable has one function only: to specify how the Schedule should call a single method.

### But if you must!

If you really can't get your head wrapped around Java's anonymous classes, we have a special Steppable subclass, called **sim.engine.MethodStep**, which provides Objective-C style message-target functionality. MethodStep allows you to write the same above as:

```
schedule.scheduleOnce(new MethodStep(myObject, "foo"));
schedule.scheduleOnce(new MethodStep(myObject, "bar"));
schedule.scheduleOnce(new MethodStep(myObject, "baz"));
```

This presumes the three methods here take no arguments. If you wish to call foo(*SimState*) rather than foo(), MethodStep has that option:

```
schedule.scheduleOnce(new MethodStep(myObject, "foo", true));
```

Again, this isn't great style. So feel free to use MethodStep when no one is looking. But be sure to hide your private shame.

## How to Create a Field Portrayal Inspector

Inspectors are primarily for objects and for the model proper. What if you want to make several inspectors for various fields? Create the Inspector as you see fit, as some subclass of sim.portrayal.Inspector. Then attach the Inspector to the Display2D (using Display2D.attach(...) ) with an appropriate name. This will put a menu for the inspector in the Display2D's layers menu.

The Social Networks package comes with an example about doing this with the Network field.

## How to Draw Directly On the Display2D *(or)* How to Create a Background Image for the Display2D

There are two different "kinds" of drawing on the Display2D you might want:

1. Draw in a fashion that auto-scales and auto-scrolls like the rest of the portrayals (perhaps a background map of a country that the portrayals appear to move over).- Draw something which retains its location and size regardless of how the Display2D is scaled or scrolled (perhaps an overlay of current statistics).

### Auto-Scaling and Auto-Scrolling

The easiest way to draw in an auto-scaling fashion is to create a SimplePortrayal2D to draw the item; create a 2D Field (say, a Continuous2D); put a random object (say the String "object") into that Field; create a FieldPortrayal2D to draw the Field (say, a ContinuousPortrayal2D), and attach it to your display.

If you use a Continuous2D, you'll want to make that your discretization significantly larger than the object you're storing. If you just have a single object, we suggest making the discretization as large or larger than the entire region of the field (so it's just one big cell).

The most common need is probably to put a map in the background of the screen. For this, your SimplePortrayal2D could be an ImagePortrayal2D. Make sure your field's width and height are of the proportions you want. Then add the object to the exact center of the field you've created. Now attach an ImagePortrayal2D along these lines:

```
myContinuousPortrayal2D.setField(theContinuousField);
MyImageIcon imageIcon = new ImageIcon("MyPicture.gif");
Image image = imageIcon.getImage();
int dimension = (int)Math.min(theContinuousField.width, theContinuousField.height);
myContinuousPortrayal2D.setPortrayalForAll(new ImagePortrayal2D(image, dimension));
myDisplay.attach(myContinuousPortrayal2D, "Overlay");
```

You want to pass in the smaller of the field's width and height. If you're filling the entire background, remember to set Display2D's backdrop to null so it doesn't bother drawing it.

### Scale-Independent

If you want to make an overlay, this is even easier. Create your own subclass of FieldPortrayal2D which overrides the draw method. The relevant coordinates are in the DrawInfo2D. Specifically, the field region is stored in the Rectangle called *draw*, and the displayed region on-screen is stored in the Rectangle called *clip*. Usually clip is what you want. For example, you might define the following FieldPortrayal2D to draw the current ticks in the top-left corner of the screen:

```
FieldPortrayal2D overlay = new FieldPortrayal2D()
    {
    Font font = new Font("SansSerif", 0, 18);  // keep it around for efficiency
    public void draw(Object object, Graphics2D graphics, DrawInfo2D info)
        {
        String s = "";
        if (state !=null)
            s = state.schedule.getTimestamp("Before Simulation", "All Done!");
        graphics.setColor(Color.blue);
        graphics.drawString(s, (int)info.clip.x + 10, 
                (int)(info.clip.y + 10 + font.getStringBounds(s,graphics.getFontRenderContext()).getHeight()));

        }
    };
```

You don't need to set the field of this FieldPortrayal2D -- just leave it empty. All you need to do is attach it to your Display and you're on your way.

Note that this method locks to the top-left corner of the clip region. If you have scrolled out a lot, the clip region will be smaller than the window and located in the center of it. If you want to *always* draw in the top-left corner of the screen, you need to tell the Display2D to stop clipping. Then the clip region will always be the scroll rectangle. However if any of your portrayals spill outside of the clip region, their unclipped drawing will now be visible.

If you're filling the entire background with this method, remember to set Display2D's backdrop to null so it doesn't bother drawing it.

Another way to draw a scale-independent image in the background is to use a TexturePaint wrapped around your image, and use that as the "Paint" to draw the Display2D's backdrop. You could, for example, add this to your setupPortrayals method:

```
Image i = new ImageIcon(getClass().getResource("MyPicture.gif")).getImage();
BufferedImage b = display.getGraphicsConfiguration().createCompatibleImage(i.getWidth(null), i.getHeight(null));
Graphics g = b.getGraphics();
g.drawImage(i,0,0,i.getWidth(null),i.getHeight(null),null);
g.dispose();
display.setBackdrop(new TexturePaint(b, new Rectangle(0,0,i.getWidth(null),i.getHeight(null))));
```

This will tile the image into the scrolled space. Unfortunately, large tiled images are buggy on certain platforms (such as OS X java 1.3.x). So we don't recommend it.
**The PacMan application demo** has an example of an overlay: sim.app.pacman.Overlay.

## How to Make an Applet

As an example, we will convert Conway's Game of Life from Tutorials 1 and 2 into an applet.

### Modify the simulation.classes File

The simulation.classes file, located in the sim/display directory, specifies which files will appear in the pop-up list when the user chooses "New Simulation...". Edit this file to include only those simulations you wish the user to have access to. If you put the word 'ONLY' on a line all by itself, the user will not be able to type in a simulation classname by hand. If the file consists solely of the world 'ONLY', then the "New Simulation..." menu will be grayed out and the user won't be able to make a new simulation at all.

### Build the Jar File

To make an applet you need two things: a web page which sets up the applet in HTML, and the applet code, typically embedded as a jar file. The jar file should contain all the non-standard classes that might be loaded during the course of running the applet, plus any pictures or auxillary data loaded by the applet. We begin by building a basic jar file.

- Go into the sim/app/tutorial1and2 directory and compile all the java files.- Go outside the mason directory. Construct a jar file out of the sim directory by typing the following at the command line

```
jar cvf mason.jar mason
```

This dumps the entire mason directory, java files and all, into a jar file called **mason.jar**. You don't need all those files -- you just need:

- The class files (\*.class)- Any images the class files load (\*.jpg, \*.png)- Any HTML files displayed in the console's "About" tab (index.html)- The sim/display/simulation.classes file

To extract just these files and no others from the MASON directory and stuff them into a jar file, the easiest approach is (in UNIX or on a Mac) to go into the mason directory and type

```
make jar
```

This is still a big file for users to download. If you **really** want to shrink this, you'll need to identify which classes and related files have to be extracted. Generally speaking the only images that need to be extracted for MASON to operate are the PNG files in the display and util/gui directories. The HTML files you need are probably just those in the application directories you wish to make available. And you'll need the simulation.classes file. As to the class files: generally you're safe if you just grab all the class files in the mason directory EXCEPT for various sim/app/... applications you don't wish to include. Certain tutorials have big pictures, and you'll save a lot of space by tossing those tutorials out. If you're not doing Java3D, you can toss out the files in the portrayal3d and display3d directories as well.

### Construct the HTML Page

Applets have changed over the years and certain web browsers <cough>IE</cough> have their own weird ways of doing things. We've constructed a sample web page that you should be able to modify. It's called **SimApplet.html** and is located in the **sim/display/** directory.

Your mason.jar file should be located on the website right next to this HTML file. An example is shown on the MASON home page.

If you want to fire up MASON directly on the simulation of your choice, you'll need to change an applet parameter called **Simulation** in your HTML file. Its value is by default **sim.display.Console**, but you can change it to a simulation class, for example, **sim.app.heatbugs.HeatBugsWithUI**

You can also change what is placed on the button by changing the **Name** parameter. Note that these parameters have to be changed in *several locations* in our version of the file, because different web browsers handle HTML in subtly different ways.

### A Warning About Java Versions

Not all operating systems run Java 1.5. For example, most web browsers on OS X run Java 1.4.2. If you compile your applet class files using Java 1.5, by default it will compile them to a bytecode version which *only runs on Java 1.5 virtual machines*, even if you don't use any new Java 1.5 features. This means your applet won't run on various operating systems.

You can compile for an earlier bytecode version by changing the **target** of your virtual machine. It's probably safe to compile to 1.3 as your target. If you're compiling using javac on the command lline, this can be as easy as saying javac -target 1.3 ... You can do the same thing with jikes, like this: jikes -target 1.3 ...


## How to Parallelize a MASON Application

MASON provides two kinds of parallelization:

- Any given step can be broken into parallel substeps each performed simultaneously. This is particularly helpful for expensive steps which can be readily parallelized on a multi-processor computer.- A step can run asynchronously in the background independent of the simulation. This is for the *rare* occasions when you need to fire off things to run on their own.

Note that parallelization uses threads and is subject to race conditions. If your parallel actions perform writes or access the random number generator, you'll need to synchronize on the schedule. Remember not to hold the lock on the schedule for long!

### Parallel Substeps

MASON provides a Steppable called **ParallelSequence**. If you load this Sequence with Steppables, when the Sequence is stepped, it will spawn independent threads to step each of its contained Steppables independently. It then waits for them to all finish their step() methods, then after they have it will clean up and finish its own step() method. Read the documentation for ParallelSequence carefully. Notably: be careful about putting a RandomSequence inside a ParallelSequence.

The MASON HeatBugs example shows a variety of approaches to improving the efficiency of the HeatBugs Diffuser, up to and including parallelizing it for multiple processors using ParallelSequence.

### Asynchronous Steppables

The **AsynchronousSteppable** class allows you to fire off a Steppable from the Schedule which goes and runs in its own thread indefinitely. The Schedule *does not wait for it to finish*. The AsynchronousSteppable class is Stoppable, and also provides a "Stopper" (a Steppable which you can *schedule* to stop the AsynchronousSteppable at a future time if it has not already stopped).

Generally speaking there are two kinds of Asynchronous Steppable operations: fast one-shot operations and slow or inifinite-loop operations. Fast one-shot operations can simply fire off in their own thread and do their thing, assuming they're fast enough that the user is willing to wait for them to finish after he has pressed the Stop or Pause buttons or is checkpointing out the simulation. Slow or infinite-loop operations must be able to respond to requests to be paused, resumed, and stopped (killed).

We expect that the use of Asynchronous Steppables should be exceedingly rare. Indeed, adding them to MASON required some retooling and we do not know if it works properly: it's essentially untested at this point. Read the AsynchronousSteppable documentation very closely.

## How to Schedule Things Weakly

What if you want to schedule something to occur only if the object still exists? But you don't want the schedule to necessarily hold onto the object if you're low on memory?

MASON provides **WeakSteppable**, a wrapper for Steppables which holds onto them as WeakReferences. It also maintains weak Stoppables as well (though that may change). Keep in mind that just because the WeakReference is being held onto by no one but the Schedule doesn't mean the Schedule will let go of it: this only occurs when Java needs to reclaim the WeakReference, perhaps during garbage collection.

When the WeakSteppable lets go of its weak reference, the WeakSteppable is *still scheduled in the Schedule*. It'll just do nothing when the Schedule gets around to stepping it. However, the time *will* advance to the scheduled point.

## How to Delete Things from the Schedule

Answer: just wait until their time has come up.

Schedule doesn't at present have a way to delete things except when their time is called. The reason is because Schedule uses a data structure called a *binary heap* to work efficiently. Heaps are good for inserting arbitrary objects and then extracting them in sorted order (in our case, sorted order means order of scheduled time). However, other arbitrary deletion of objects from binary heaps is highly inefficient. Instead, the better way to "delete" an object is to "stop" it and forget about it. It'll drop out of the Schedule in due time. How you "stop" an agent depends on whether or not it's a one-shot agent or a repeating agent, which we discuss next.

**One-shot agents.** Agents can be scheduled on the Schedule in two ways: one-shot agents and repeating agents. When an agent is scheduled just once, it sits in the Schedule until its time has come. At that point the agent is deleted from the Schedule and its step() method is called. If you're writing a one-shot agent, and you want your agent out of the Schedule, all you have to do is tell your agent to ignore its sole step() method.

But perhaps this is inconvenient: you might want to reuse the agent and its step() method -- perhaps to reschedule it at a future time instead. Having it ignore future step() calls isn't feasible in that case. Then instead you can wrap it in another Steppable which can be stopped. We provide such an object: sim.engine.TentativeStep. When its step() is called, this object will in turn call step() on its subsidiary Steppable unless stop() has been called, at which time it just forgets about the subsidiary object (sets it to null). You can wrap your Steppable in various TentativeSteps and schedule each of them independently on the Schedule, then stop() various ones at your leisure.

**Repeating agents.** A repeating-scheduled agent has actually just been wrapped in a private 'Repeat' Steppable. When the step() method is called on the 'repeat' object, it simply calls step() on its subsidiary Steppable (your agent), then it reschedules itself in the Schedule for a later date. This 'repeat' object is also a Stoppable: when its stop() method is called, then the subsidiary Steppable is set to null and the 'Repeat' object ceases to function -- no later subsidiary step(), no later rescheduling.

This Stoppable is handed to you when you call scheduleRepeating(...): to delete a repeating object from the Schedule, you merely need to call stop() on the Stoppable and forget about the agent.

## How to Delete Things from a Field

We occasionally get questions about deleting objects from fields. All fields are designed to have objects removed very efficiently. Specifically:

**SparseGrid2D, SparseGrid3D, Continuous2D, Continuous3D.** Use the remove(obj) method declared in these classes' superclass, SparseField. Under no circumstances should you manually remove objects from the Bags of these classes -- there's a lot of unhooking that must be done in different places. Let remove(obj) do the job for you. You can also call removeObjectsAtLocation(location) (and variations thereof) which removes and returns all objects at a given location. And you can call clear() to delete all objects in the entire field.

**ObjectGrid2D, ObjectGrid3D.** You remove elements from these objects by simply deleting the elements from their underlying arrays. The clear() function will delete all elements for you.

**Network.** Use removeEdge(edge) to delete an edge from the graph, and removeNode(node) to delete a node from the graph. Once an edge has been removed from the graph, you can reuse it by adding it to another graph, but you cannot change the nodes it is connected to (they will be added to the other graph as well if not already present). You can also delete all nodes and edges from the graph with clear().

**IntGrid2D, IntGrid3D, DoubleGrid2D, DoubleGrid3D**. There are no removal functions in these fields as there is nothing to "remove".

## How to Add a Slider or Pop-Up Menu to an Inspected Property

By default inspected properties take one of four forms:

- A label (for Strings, numbers, etc., that cannot be modified)- A text field (for Strings, numbers, etc., that can be modified)- A checkbox (for boolean properties)- A button (for Objects that can be inspected further)

It's often the case that you might want to have a numerical property with minimum and maximum bounds. In this case you might want the user to be able to enter values with a slider rather than having to enter them in a text field (which is more cumbersome for real-time control).

It's easy to do. Let's say that your property looks like this:

```
public void setMyProperty(double val) { ... }
public double getMyProperty() { ... }
```

Let's say you want to specify that the minimum of this property is 4 and the maximum is 18.3. What you need to do is add a new method called domMyProperty() like this.

```
public void setMyProperty(double val) { ... }
public double getMyProperty() { ... }
public Object domMyProperty() { return new sim.util.Interval(4.0, 18.3); }
```

The **dom** marker (which is special to MASON) tells MASON that your numerical property has a **domain** bounded by a minimum and maximum. MASON then will use a slider to allow the user to set it to values within that domain. You can do this with any integer (byte/short/int/long) or floating-point (float/double) property. For integer properties you should specify the minimum and maximum as longs:

```
public void setMyOtherProperty(short val) { ... } 
public short getMyOtherProperty() { ... }
public Object domMyOtherProperty() { return new sim.util.Interval(7L, 15L); }
```

Not the use of **L** to denote a long. This is important; otherwise Java will think the Interval is defined in terms of doubles.

If your integer (byte/short/int/long) property is defined in terms of values **0...n**, you also have the option of having MASON provide a pop-up list of **n** names, each of which represents one of those values. For example, for value 0 you might want the pop-up list to display "North", and for value 1 you might want it to display "East", etc. Then when the user picks "East", your property is set to 1. This can be done by providing an array in this fashion:

```
public void setYetAnotherProperty(int val) { ... } 
public int getYetAnotherProperty() { ... }
public Object domYetAnotherProperty() { return new String[] { "North", "East", "South", "West" }; }
```

## How to Draw Faster

**Use Java 1.3.1 on MacOS X when Running on PowerPC** 1.3.1 is hardware accelerated. 1.4.2 and 1.5 *are not!* They are exceptionally slow at drawing 2D, and you will be shocked to find how much faster 1.3.1 is (except on Intel Macs, where at present it is extremely slow -- you must do 1.5 there). However if you need to do 3D visualization, you have no choice but to use 1.4.2 or 1.5. MASON's mason.command script launches with the default Java (1.4.2 or 1.5), while mason.1.3.1.command launches with Java 1.3.1.

**Use a SparseGrid rather than an ObjectGrid** ObjectGrid portrayals generally work by going through the entire ObjectGrid and drawing each and every grid cell, whether it contains an object or not. SparseGrid just goes through its collection of objects and draws each one in turn. Whenever your environment is sparse (you have a large grid but rather few objects inhabiting it), you might try SparseGrid. The trade-off is that SparseGrid stores its objects' locations in a hash table, and so looking up or changing the location of an object requires one or several hashes, plus some wandering through arrays. This is typically an O(1) operation but it incurs a significant overhead, so if you have a *lot* of objects -- your environment isn't very sparse -- then SparseGrid will be significantly slower than ObjectGrid. Try and see.

**Avoid ObjectGrid3D and ValueGrid3D** Java3D is slow. Drawing large, non-sparse 3D grids of objects in Java3D is slow to the point of painful, and requires an enormous amount of additional memory. Avoid it if possible.

**Avoid Java Graphics2D in Custom Simple Portrayals** Use Java AWT graphics whenever possible: Graphics2D is generally much slower.

**Zoom In** MASON's Display2D only draws that portion of the fields which are actually shown on-screen; the rest is carefully ignored (exception: NetworkPortrayal2D). If you show less of the field, fewer objects are drawn.

**Declare an Immutable Field** If you have a field which never changes, you can declare this in certain "Fast" field portrayals (see below), and they will instead convert it into an image and just re-splat the image each time. To take advantage of this speedup in Windows or Linux, you may need to increase your heap memory significantly. The field portrayals which can do this: FastObjectGridPortrayal2D, FastValueGridPortrayal2D, FastHexaObjectGridPortrayal2D, FastHexaValueGridPortrayal2D.

**Use a Faster Field Portrayal** In several situations MASON has both **regular but flexible** and **fast but limited** field portrayals for 2D and 3D fields. All of the "fast" versions of the field portrayals draw their fields as simple grids of rectangles rather than other prettier representations. The idea behind the "fast" versions is that they draw these grids very rapidly using Java tricks rather than individually calling up simple portrayals to draw each grid region. Typically these portrayals represent elements simply as colored rectangles, bypassing simple portrayals entirely. In most cases they can draw these rectangles in two ways which the user can choose in the Options panel.

- Draw each rectangle separately (faster in Windows and Linux in normal memory usage)- Construct an image, with each pixel color being a single grid cell value, then stretch the image to fill the region (much faster in OS X, and faster in Windows and Linux if given more heap memory)

Look further down for a list of fast field portrayals.

To draw their colored rectangles, the fast field portrayals require a ColorMap which maps numerical (double) values to color representations. For example, to translate values of 0.0 through 100.0 to range smoothly from red to blue, (values outside those ranges get thresholded to red or blue respectively), you might write:

```
portrayal.setMap(new sim.util.gui.SimpleColorMap(0.0, 100.0, Color.red, Color.blue));
```

You can also have per-integer colors too, if your values range from 0...n. To set the three integers 0, 1, 2 to three different colors, you could write:

```
portrayal.setMap(new sim.util.gui.SimpleColorMap(new Color[] { Color.red, Color.blue, Color.green }));
```

You can make SimpleColorMaps which do both things at the same time as well. See the SimpleColorMap documentation. Or you can make your own ColorMap that does something more complex (like a nonlinear translation in colors).

What about ObjectGrid2D, which doesn't have numerical values, but has actual Objects? The "fast" ObjectGrid2D portrayals first translate the Objects into numbers, then run the numbers into the ColorMap, using the Portrayals' built-in doubleValue(...) method. The default version of the method works like this: if the object is null, 0.0 is returned. If the object is a java.util.Number or is sim.util.Valuable, then obj.doubleValue() is returned. Else 1.0 is returned. This probably works for most situations you have, but you're welcome to override it to provide more specific values.

*Speed hint #1 for ColorMaps.* If a large portion of your map is going to be the same color, consider making it 100% transparent (new Color(0,0,0,0)). Just set the backdrop color of the Display instead and let it bleed through. If a grid cell is completely transparent, MASON avoids drawing it all, resulting in a major speed boost.

*Speed hint #2 for ColorMaps.* If you're running on Linux or Windows, semi-transparent colors are expensive to draw. Use opaque or fully transparent. On OS X semi-transparency is quite fast.

Last, certain hexagonal portrayals differ in an important way speed-wise. The HexaValueGridPortrayal2D draws with true hexagons, but it does not use an underlying simple portrayal -- instead it just draws the hexagons directly. In fact, it's the *only* non-"fast" portrayal to use a ColorMap rather than a SimplePortrayal, and it does this because the speed gains are to significant. But you can get even faster yet: the FastHexaValueGridPortrayal2D draws its hexagonal values not with hexagons but with rectangles, laid on in brick-wall fashion. This allows it to take advantage of the two approaches to drawing square grids as discussed earlier, and it's much *much* faster than drawing hexagons. The FastHexaObjectGridPortrayal2D likewise draws with rectangles.

Here's a chart of fields and their regular/fast portrayals (as of MASON 11).

|  |  |  |  |  |  |  |  |  |  |  |  |  |  |  |  |  |  |  |  |  |  |  |  |  |  |  |  |  |  |  |  |  |  |  |  |  |  |  |  |  |  |  |  |  |  |  |  |  |  |  |  |  |  |  |  |  |  |  |  |  |  |  |  |  |  |  |  |
| --- | --- | --- | --- | --- | --- | --- | --- | --- | --- | --- | --- | --- | --- | --- | --- | --- | --- | --- | --- | --- | --- | --- | --- | --- | --- | --- | --- | --- | --- | --- | --- | --- | --- | --- | --- | --- | --- | --- | --- | --- | --- | --- | --- | --- | --- | --- | --- | --- | --- | --- | --- | --- | --- | --- | --- | --- | --- | --- | --- | --- | --- | --- | --- | --- | --- | --- | --- |
| **Field** **Layout** **Regular Field Portrayal** **Fast Field Portrayal**| **2D Field Portrayals**| IntGrid2D or DoubleGrid2D Rectangular ValueGridPortrayal2D FastValueGridPortrayal2D|  |  |  |  |  |  |  |  |  |  |  |  |  |  |  |  |  |  |  |  |  |  |  |  |  |  |  |  |  |  |  |  |  |  |  |  |  |  |  |  |  |  |  |  |  |  |  |  |  |  |  |  |  |  |  |  | | --- | --- | --- | --- | --- | --- | --- | --- | --- | --- | --- | --- | --- | --- | --- | --- | --- | --- | --- | --- | --- | --- | --- | --- | --- | --- | --- | --- | --- | --- | --- | --- | --- | --- | --- | --- | --- | --- | --- | --- | --- | --- | --- | --- | --- | --- | --- | --- | --- | --- | --- | --- | --- | --- | --- | --- | | IntGrid2D or DoubleGrid2D Hexagonal HexaValueGridPortrayal2D FastHexaValueGridPortrayal2D|  |  |  |  |  |  |  |  |  |  |  |  |  |  |  |  |  |  |  |  |  |  |  |  |  |  |  |  |  |  |  |  |  |  |  |  |  |  |  |  |  |  |  |  |  |  |  |  |  |  |  |  | | --- | --- | --- | --- | --- | --- | --- | --- | --- | --- | --- | --- | --- | --- | --- | --- | --- | --- | --- | --- | --- | --- | --- | --- | --- | --- | --- | --- | --- | --- | --- | --- | --- | --- | --- | --- | --- | --- | --- | --- | --- | --- | --- | --- | --- | --- | --- | --- | --- | --- | --- | --- | | ObjectGrid2D Rectangular ObjectGridPortrayal2D FastObjectGridPortrayal2D|  |  |  |  |  |  |  |  |  |  |  |  |  |  |  |  |  |  |  |  |  |  |  |  |  |  |  |  |  |  |  |  |  |  |  |  |  |  |  |  |  |  |  |  |  |  |  |  | | --- | --- | --- | --- | --- | --- | --- | --- | --- | --- | --- | --- | --- | --- | --- | --- | --- | --- | --- | --- | --- | --- | --- | --- | --- | --- | --- | --- | --- | --- | --- | --- | --- | --- | --- | --- | --- | --- | --- | --- | --- | --- | --- | --- | --- | --- | --- | --- | | ObjectGrid2D Hexagonal HexaObjectGridPortrayal2D FastHexaObjectGridPortrayal2D|  |  |  |  |  |  |  |  |  |  |  |  |  |  |  |  |  |  |  |  |  |  |  |  |  |  |  |  |  |  |  |  |  |  |  |  |  |  |  |  |  |  |  |  | | --- | --- | --- | --- | --- | --- | --- | --- | --- | --- | --- | --- | --- | --- | --- | --- | --- | --- | --- | --- | --- | --- | --- | --- | --- | --- | --- | --- | --- | --- | --- | --- | --- | --- | --- | --- | --- | --- | --- | --- | --- | --- | --- | --- | | SparseGrid2D Rectangular SparseGridPortrayal2D |  |  |  |  |  |  |  |  |  |  |  |  |  |  |  |  |  |  |  |  |  |  |  |  |  |  |  |  |  |  |  |  |  |  |  |  |  |  |  |  | | --- | --- | --- | --- | --- | --- | --- | --- | --- | --- | --- | --- | --- | --- | --- | --- | --- | --- | --- | --- | --- | --- | --- | --- | --- | --- | --- | --- | --- | --- | --- | --- | --- | --- | --- | --- | --- | --- | --- | --- | | SparseGrid2D Hexagonal HexaSparseGridPortrayal2D |  |  |  |  |  |  |  |  |  |  |  |  |  |  |  |  |  |  |  |  |  |  |  |  |  |  |  |  |  |  |  |  |  |  |  |  | | --- | --- | --- | --- | --- | --- | --- | --- | --- | --- | --- | --- | --- | --- | --- | --- | --- | --- | --- | --- | --- | --- | --- | --- | --- | --- | --- | --- | --- | --- | --- | --- | --- | --- | --- | --- | | Continuous2D ContinuousPortrayal2D |  |  |  |  |  |  |  |  |  |  |  |  |  |  |  |  |  |  |  |  |  |  |  |  |  |  |  |  |  |  |  |  | | --- | --- | --- | --- | --- | --- | --- | --- | --- | --- | --- | --- | --- | --- | --- | --- | --- | --- | --- | --- | --- | --- | --- | --- | --- | --- | --- | --- | --- | --- | --- | --- | | Network NetworkPortrayal2D |  |  |  |  |  |  |  |  |  |  |  |  |  |  |  |  |  |  |  |  |  |  |  |  |  |  |  |  | | --- | --- | --- | --- | --- | --- | --- | --- | --- | --- | --- | --- | --- | --- | --- | --- | --- | --- | --- | --- | --- | --- | --- | --- | --- | --- | --- | --- | | **3D Field Portrayals**| IntGrid3D or DoubleGrid3D Rectangular ValueGridPortrayal3D |  |  |  |  |  |  |  |  |  |  |  |  |  |  |  |  |  |  |  |  | | --- | --- | --- | --- | --- | --- | --- | --- | --- | --- | --- | --- | --- | --- | --- | --- | --- | --- | --- | --- | | SparseGrid3D Rectangular SparseGridPortrayal3D || ObjectGrid3D or ObjectGrid2D Rectangular ObjectGridPortrayal3D |  |  |  |  |  |  |  |  |  |  |  |  |  | | --- | --- | --- | --- | --- | --- | --- | --- | --- | --- | --- | --- | --- | | IntGrid2D or DoubleGrid2D Rectangular ValueGridPortrayal3D ValueGrid2DPortrayal3D|  |  |  |  |  |  |  |  |  | | --- | --- | --- | --- | --- | --- | --- | --- | --- | | SparseGrid2D Rectangular SparseGridPortrayal3D or SparseGrid2DPortrayal3D || Continuous2D or Continuous3D ContinuousPortrayal3D || Network NetworkPortrayal3D | | | | | | | | | | | | | | | | | | | | | | | | | | | | | | | | | | | | | | | | | | | | | | | | | | | | | | | | | | | | | | | | |

## How to Scale or Translate a 2D Field Portrayal's Depiction On-screen

Scaling and translation of field portrayals usually occurs when you need to adjust different portrayals so that they lie properly on top of one another. Translation also occurs when you wish to center the origin. At present MASON has no facilities for flipping the Y axis so that it goes up rather than down. Instead, you may need to adjust your model data's arrangement.

In any of the following cases, you may also find it helpful to eliminate clipping of the display region:

```
display.setClipping(false);
```

**Translating** By default, the origin of a Field Portrayal is in the top-left corner of the screen. If you wish to center the origin on-screen, for example, you need to translate it to the middle of the display area. This can be done by attaching the portrayal to the display not in the usual way, but like this:

```
display.attach(portrayal, "Portrayal Text Goes Here", display.insideDisplay.width / 2.0, 
                                                      display.insideDisplay.height / 2.0, true);
```

If your display has an odd width or height, you may wish to make sure that the origin lies exactly on a pixel:

```
display.attach(portrayal, "Portrayal Text Goes Here", display.insideDisplay.width / 2, 
                                                      display.insideDisplay.height / 2, true);
```

**Scaling**You might also wish to scale the portrayal. By default the scaling adjusts the portrayal so that its width and height exactly fill the width and height of the window. That is, the following statements are identical:

```
display.attach(portrayal, "Portrayal Text Goes Here");
```

  
and

```
display.attach(portrayal, "Portrayal Text Goes Here", 
    new Rectangle2D.Double(0,0, display.insideDisplay.width, display.insideDisplay.height), true);
```

Let's say you want to scale the portrayal so that twice its width and twice its height are displayed on-screen. You could instead attach like this:

```
display.attach(portrayal, "Portrayal Text Goes Here", 
    new Rectangle2D.Double(0,0, display.insideDisplay.width/2.0, display.insideDisplay.height/2.0), true);
```

Again, you may wish to scale exactly to a pixel boundary if the height or width of the window is odd:

```
display.attach(portrayal, "Portrayal Text Goes Here", 
    new Rectangle2D.Double(0,0, display.insideDisplay.width/2, display.insideDisplay.height/2), true);
```

**Translating and Scaling** You can scale and translate the portrayal by providing a Rectangle2D.Double which
defines (1) the location of the portrayal origin and (2) the number of pixels that the portrayal's width and height take up by default. This is basically a combination of the above two issues. Let's say you wish to move the origin to the center of the screen and scale out the portrayal by a factor of 10 (so 100 times as much area is displayed in the window). You could write something along the lines of:

```
display.attach(portrayal, "Portrayal Text Goes Here", 
    new Rectangle2D.Double(display.insideDisplay.width/2.0,  // translated x origin
                           display.insideDisplay.height/2.0, // translated y origin
                           display.insideDisplay.width/100.0, // scaled width
                           display.insideDisplay.height/100.0), // scaled height
    true);
```

...and again, you could change these from doubles to integers to more cleanly adjust to pixel boundaries.

## How to Create a Plug-In Inspector for a Single Property

MASON actually has two kinds of inspectors:

- **Official inspectors** for objects which pop up in response to double-clicking on an object in a Display or pressing the "View" button in a property.- **Additional "Plug-in" inspectors** (subclasses of PropertyInspector) for single properties. These are displayed in a pop-up menu from the magnifying glass icon ()

At present MASON provides plug-in inspectors for streaming a property to a file and for charting the change of a property over time. If you wish to make another inspector, here's what you need to do. Let's say you want to stream a property to standard out.

1. Create a subclass of PropertyInspector.- Add a static String method called name() which returns the text to put in the menu.- Add a static Class[] method called types() which returns an array of Classes representing the types of properties this inspector is capable of inspecting. Return null for all types.- Add a constructor *Foo*(Properties properties, int index, Frame parent, GUIState simulation) which builds a PropertyInspector given property #index in the provided properties. parent is provided in case you want to pop up a dialog and would like it to be associated with a known Frame.- If you wish to proceed to display a Property Inspector, set the protected variable validInspector to true
           near the end of your constructor. Otherwise MASON will assume that you want to give up (perhaps you had popped up
           a dialog box and the user cancelled the request).- If you are proceeding, MASON will then attempt to create a frame and put your inspector in it. If you do not want this to happen -- perhaps you created your own frame already, or your inspector doesn't have any frame for some reason -- then override shouldCreateFrame() to return false. (It returns true by default).- If/when MASON creates a frame for your inspector, it calls the createFrame(Stoppable) method. By default this method registers the frame to be closed when the simulation is restarted, sets the title of the frame in a simple way, and returns the frame. Perhaps you don't want the frame to be closed, or would like to change the title. You might override this method to return your own frame (or call super.createFrame(stoppable) and modify the frame you get back).- If you need to perform something special (flushing out a stream, say) when the inspector is stopped, override
                 the reviseStopper(...) method.- Override updateInspector() to update your inspector.

Last, to tell MASON that it should dynamically load your inspector and plug it in, you need to edit the sim/portrayal/inspector/propertyinspector.classes file and add the classname of your inspector.

The Inspector and PropertyInspector class documentation has further discussion on the issue. Here's a very simple PropertyInspector which writes values out to System.out:

```
import java.awt.*;
import javax.swing.*;
import sim.display.*;
import sim.engine.*;
import sim.util.*;
import sim.portrayal.inspector.*;

public class StreamOutPropertyInspector extends PropertyInspector
    {
    double lastTime  = Schedule.BEFORE_SIMULATION;
    public static String name() { return "Stream to System.out"; }
    public static Class[] types() { return null; } // accepts all types
        
    public StreamOutPropertyInspector(Properties properties, int index, Frame parent, GUIState simulation)
        {
        super(properties,index,parent,simulation);
        add(new JLabel("Streaming " + properties.getName(index) + " to System.out"));
        validInspector = true;  // we always want to go ahead with displaying this inspector
        }

    public void updateInspector()
        {
        double time = simulation.state.schedule.time();
        if (lastTime < time)  // only print if this is a new timestamp
            {
            lastTime = time;
            // print like this...   object/property/timestamp: value
            System.out.println("" + properties.getObject() + "/" + 
                               properties.getName(index) + "/" + 
                               lastTime + ": " + properties.getValue(index));
            }
        }
        
    // We always want to make a frame, so we don't override this
    // public boolean shouldCreateFrame() { return false; }
    
    // We should flush our stream when we're stopped.  So let's wrap the stopper to do this.
    public Stoppable reviseStopper(Stoppable stopper)
        {
        final Stoppable newStopper = super.reviseStopper(stopper);
        return new Stoppable()
            {
            if (newStopper!=null) newStopper.stop();  // wraps the stopper
            System.out.flush();
            };
        }
    }
```

## How to Create an Inspector Holding Other Inspectors

Sometimes it's a pain to squish everything you want to inspect into a single Inspector.
MASON provides a class called TabbedInspector which acts as a holder for multiple Inspectors
(putting them into a JTabPane). This lets you break your inspector into multiple inspectors to simplify
life for you.

It's pretty easy. During your Simple Portrayal's getInspector() method, or while adding a model inspector etc.:

- Create a TabbedInspector.- Specify whether or not you want the Inspector to be *volatile*. The rule we follow is: if the TabbedInspector is declared to be volatile, we assume that *all* of its children are also volatile. If the TabbedInspector is declared to be nonvolatile, we assume that *none* of its children are also volatile.- Specify whether or not you want *all* child inspectors to be updated every time the TabbedInspector is updated; or if you only want the frontmost child inspector to be updated. When the user chooses another front child, it will automatically get updated to refresh itself.- Add your various child inspectors with addInspector(Inspector inspector,String name).- Return the TabbedInspector as your inspector.

## How to Add a Chart Programmatically

MASON has a built-in time-series charting facility, but the user must manually call it up each time he wants to chart something. If you want a chart to appear as a display, that is, as a permanent window in the simulation (and appearing in the Displays tab), here's how to do it.

In your GUIState subclass, you'll need to add some instance variables:

```
    org.jfree.data.xy.XYSeries series;    // the data series we'll add to
    sim.util.media.chart.TimeSeriesChartGenerator chart;  // the charting facility
```

Next, you need to construct the chart in your init method, like so:

```
    JFrame chartFrame;

    public void init(Controller c)
       {
       super.init(c);
       
       
       [do your main code here, then...]
       
       
       chart = new sim.util.media.chart.TimeSeriesChartGenerator();
       chart.setTitle("Put the title of your chart here");
       chart.setRangeAxisLabel("Put the name of your charted series here");
       chart.setDomainAxisLabel("Time");
       chartFrame.createFrame();
       // perhaps you might move the chart to where you like.
       chartFrame.setVisible(true);
       chartFrame.pack();
       c.registerFrame(chartFrame);
       
       // the console automatically moves itself to the right of all
       // of its registered frames -- you might wish to rearrange the
       // location of all the windows, including the console, at this
       // point in time....
       }
```

Next, in your GUIState's start() method, you'll need to create a new series and add it to the chart.
Then you'll need to schedule an event on the GUIState's minischedule to add data to the chart and update
it (updating it allows it to do things like emit movies properly).

```
    public void start()
        {
        super.start();


        [do your main code here, then...]
       
       
        chart.removeAllSeries();
        series = new org.jfree.data.xy.XYSeries(
            "Put a unique name for this series here so JFreeChart can hash with it",
            false);
        chart.addSeries(series, null);
        scheduleRepeatingImmediatelyAfter(new Steppable()
            {
            public void step(SimState state)
               {
               // at this stage we're adding data to our chart.  We
               // need an X value and a Y value.  Typically the X
               // value is the schedule's timestamp.  The Y value
               // is whatever data you're extracting from your 
               // simulation.  For purposes of illustration, let's
               // extract the number of steps from the schedule and
               // run it through a sin wave.
               
               double x = state.schedule.time(); 
               double y = Math.sin(state.schedule.getSteps()) * 10;
               
               // now add the data
               if (x >= state.schedule.EPOCH && x < state.schedule.AFTER_SIMULATION)
                   {
                   series.add(x, y, true);

                   // we're in the model thread right now, so we shouldn't directly
                   // update the chart.  Instead we request an update to occur the next
                   // time that control passes back to the Swing event thread.
                   chart.updateChartLater(state.schedule.getSteps());
                   }
               }
           });
       }
```

Why are we passing the steps into updateChartLater(...)? Because it needs to know if it's written out a movie frame already which reflected that timestep.

Last, you probably want movies to end when the simulation is stopped or finished: and to update themselves one last time to write out that last movieframe. And you certainly want to dispose the JFrame if you're quitting.

```
    public void finish()
        {
        super.finish();

        [do your finishing code here, then...]

	chart.update(state.schedule.getSteps(), true);
	chart.repaint();
        chart.stopMovie();
        }


    public void quit()
        {
        super.quit();

        [do your quitting code here, then...]

        chart.update(state.schedule.getSteps(), true);
        chart.repaint();
        chart.stopMovie();
        if (chartFrame != null)	chartFrame.dispose();
        chartFrame = null;
        }
```

When you run the simulation, you'll notice that the generator doesn't have all the features of the standard one which pops up with inspectors -- notably you don't have the ability to state how often the chart is updated. These are features of the inspector code, not the chart generator. Your programmatic mechanism is updating the chart every time a value is inserted into it. This is expensive, particularly when you have lots of data points.

If you would like the chart to redraw itself once every second of wall-clock time after values have been sent to it (say), ChartGenerator has a little timer facility you can use to have it update itself no further than, in this case, one second after you start the timer. You can replace the updateChartLater method with

Now you just need to tell the series not to update itself automatically when an item is added, and also to turn on the timer (if it's not already on). In the start() method, change the lines:

```
              series.add(x, y, true);
              chart.updateChartLater(state.schedule.getSteps());
```

To...

```
              series.add(x, y, false);  // don't immediately redraw on adding data
              chart.updateChartWithin(state.schedule.getSteps(), 1000);  // update within one second (1000 milliseconds))
```

## How to Hide the Console

In the past we've been asked how to hide the Console and only show the Display2D or Display3D -- perhaps to use MASON as a video game, or in a "Kiosk mode" of sorts.

There are many ways to do this: you could replace the Console with a Controller of a different sort which doesn't display it, for example. But here we're going to take a simple, if somewhat inefficient, approach: use the Console but not display it.

We'll use **HeatBugsWithUI.java** as our target example.

The first step is (not surprisingly) to hide the Console. We do this by commenting out c.setVisible(true) in the **main()** method. Once we've hidden the Console, we need a way to start the simulation. We could do this the hard way -- creating thread locking, calling start() on various things, etc., or we could just do it the simple way: manually press the PLAY button on the Console. We do this by calling c.pressPlay() like this:

```
   public static void main(String[] args)
       {
        HeatBugsWithSimpleUI heatbugs = new HeatBugsWithSimpleUI();
        Console c = new Console(heatbugs);
        //c.setVisible(true);
        c.pressPlay();
        }
```

Easy enough. But the user can still access the Console via a menu on the Display2D or Display3D. This menu option is created with the Display2D/Display3D method createConsoleMenu(). We need to disable this menu. A straightforward way to do this is in HeatBugsWithUI is to create an anonymous subclass of Display2D which overrides this method. In the **init** method we change:

```
        display = new Display2D(400,400,this);
```

...to...

```
        display = new Display2D(400,400,this)
            {
            public void createConsoleMenu() { }
            };
```

At this point our simulation runs fine. But if the user clicks on the close button of the Display2D window, the program doesn't quit! This is because MASON's default behavior is to just hide the Display2D window -- to actually quit a MASON simulation, you click on the Console's close box. But the user can't see the Console any more, so how do we quit? We'll do this by changing the behavior of clicking on the Display2D window. We start by adding the following to the very end of the **init** method:

```
        displayFrame.setDefaultCloseOperation(javax.swing.JFrame.DISPOSE_ON_CLOSE);
```

This tells the window to destroy itself rather than hide itself when closed. Another option is EXIT\_ON\_CLOSE, which terminates the application. But doing so doesn't cleanly shut down MASON -- movies don't get flushed out, and the user's simulation shutdown methods aren't properly called. Instead, we'll tap into the window's machinery called when it is disposed, and use that machinery to quit the program.

Assuming you created your JFrame with display.createFrame(), thus allowing the Display2D to make the frame for you, then your JFrame has some dispose machinery built-in. When such a JFrame is disposed, it first calls quit on its underlying Display2D. We'll hook into this to quit the program cleanly. There are lots of ways to quit the program, but as long as we're using Console, by far the simplest is to tell the Console that *it's* close box was clicked on and let it handle things for us. We do this by extending our anonymous subclass to override the Display2D's quit() method:

```
        display = new Display2D(400,400,this)
            {
            public void createConsoleMenu() { }
            public void quit()
                {
                super.quit();  // let the Display2D do whatever it was doing before!
                ((Console)c).doClose();
                }
            };
```

Where did the **c** variable come from? It's the parameter passed into the **init** method in which this code appears. Java doesn't have proper closures -- all closed variables must be declared final -- so we need to change the signature of the **init** method as well (no biggie):

```
    public void init(final Controller c)
```

Last, you may wish to hide the header bar on the Display2D/Display3D -- the bar which lets the user make movies and snapshots, zoom in and out, change the refresh rate and displayed layers, etc. We already removed the primary difficulty in this bar (the menu option which calls forth the Console), but we can remove the entire bar as well. In the **init** method, just add:

```
        display.remove(display.header);
```

A final note: when the user clicks on the window, he can still generate selection events and inspection events, creating inspectors etc. This isn't a big problem because those inspectors are hidden in the Console and so they don't typically take up a lot of processing power (they're not displayed). But if we want to eliminate the ability to create them at all, we can do that too. In Display2D we say:

```
       display.insideDisplay.removeMouseListeners();
```

This method will delete all the MouseListeners, MouseMotionListeners, and (for good measure) KeyListeners registered with the Display2D.

Next you may wish to prevent scrolling and resizing of the window. To remove the scroll bars, add:

```
		display.display.setVerticalScrollBarPolicy(display.display.VERTICAL_SCROLLBAR_NEVER);
		display.display.setHorizontalScrollBarPolicy(display.display.HORIZONTAL_SCROLLBAR_NEVER);
```

...and to eliminate resizing of the window, after displayFrame has been created, add:

```
		displayFrame.setResizable(false);
```

Last, once you've deleted the header and/or scroll bars, you'll need to repack the window so that it's the right size.

```
		displayFrame.pack();
```

For Display3D, it's somewhat more involved, and we'll get a nicer approach soon.

For a full example of how to hide the console to make an actual video game, see the MASON version of PacMan.

## How to Show that an Object Has Been "Selected" by the Mouse

You may not know it, but MASON has a separate facility for registering objects which have been "selected" by the mouse, typically by clicking once on them. This is different than calling forth inspectors on objects by double-clicking them.

Why would you want to select an object? Typically to hilight it or change its mode to get some immediate information about it. Or perhaps to provide some auxillary code to select objects and then do thing swith them (move them around with keystrokes or cut and paste them or whatnot).

MASON's selection facility is primitive: when you click in a location, every item intersecting that location is selected. But you cannot immediately *tell* as a user that something has been selected. Instead, MASON provides some special portrayals which change their mode based on whether the object has been selected or not. For example, **CircledPortrayal2D** can be set up to only display its "halo" around the object if the object has been selected. You can register a CircledPortrayal2D instead of the previous SimplePortrayal along the lines of:

```
    theFieldPortrayal.setPortrayalForAll(new CircledPortrayal2D(theOriginalSimplePortrayal, Color.red, true));
```

The 'true' means "only display when the underlying portrayal's object has been selected". If there is no underlying portrayal (the object portrays itself), pass in *null* for the portrayal. See **NetworkTestWithUI.java** for an example of doing this.

You can do a similar thing with **LabeledPortrayal2D** and other simple "wrapper" portrayals in 2D and 3D. For example, see **MavDemoWithUI.java** for an example for using both CircledPortrayal2D and LabeledPortrayal2D together in this context. Furthermore, **Balls3DWithUI.java** shows selection using CircledPortrayal3D.

Also if you like you can create your own SimplePortrayal which changes its mode based on whether or not it has been selected. To do this, override the **setSelected** method along these lines:

```
    boolean isSelected;
    public boolean setSelected(LocationWrapper wrapper, boolean selected)
        {
        isSelected = selected;
        return true;  // if I return false, I tell Display2D I reject being selected/deselected
        }
```

Now you could change your drawing to reflect whether your isSelected variable is currently set.

## How to Move an Agent in 2D by Dragging It with the Mouse

Previous versions of MASON suggested a relatively complex movement mechanism. This mechanism is still
plausible (see the commented-out code in HeatBugsWithUI.java or NetworkTestWithUI.java), but instead
you should try using MovablePortrayal2D, a special wrapper portrayal which enables objects to be easily
moved in Display2Ds.


## How to Change the Look of a Provided SimplePortrayal2D Dynamically Based on the Object Status

Many of the standard provided SimplePortrayal2D objects have a **paint** variable (holding the Paint of the portrayal) and a **scale** variable (indicating how much to scale the object by). Let's say you are representing objects with an OvalPortrayal2D. But you'd like the oval to change color and size based on two instance variables in your object, **angriness** and **irritation**. It's actually quite easy. Instead of registering an OvalPortrayal2D with the FieldPortrayal, instead register an anonymous subclass which overrides the **draw** method like this:

```
    OvalPortrayal2D oval = new OvalPortrayal2D()
        {
        public void draw(Object object, Graphics2D graphics, DrawInfo2D info)
            {
            MyObject myobject = (MyObject)object;
            int a = myobject.angriness;  // let's say it goes from 0 to 255
            paint = new java.awt.Color(a, 20, 20);
            double i = myobject.irritation;  // let's say it goes from 0 to 3 
            scale = i + 1;  // range from 1x size to 4x size
            super.draw(object, graphics, info);  // it'll use the new paint and scale values
            }
        };
```

## How to Draw "Trails" or "Mouse Tails" Dragging Behind Objects

You may wish to draw trails behind objects as they move about, thus showing where they've been in the past. MASON has a special SimplePortrayal2D wrapper class which is designed for this purpose: **sim.portrayal.simple.TrailedPortrayal2D**. The class comes with a lot of class documentation describing how you use it. Also the HeatBugsWithUI and FlockersWithUI classes have (commented out) examples of using TrailedPortrayal2D.
