# Supplementary material for: Out of the net: An agent-based model to study human movements influence on local-scale malaria transmission
Source: PLoS One. 2018 Mar 6;13(3):e0193493. doi: 10.1371/journal.pone.0193493 (PMC5839546; doi:10.1371/journal.pone.0193493)
Supplement: S2 File — (ZIP) [file pone.0193493.s002.zip › S2/docs/index.html]

MASON


# M A S O N

MASON is a single-process discrete-event simulation core and visualization library aimed at multiagent simulations with large numbers of agents. The system is written in pure Java and is intended for experienced Java coders who want something general and easily hackable to start with, rather than a domain-specific simulation environment. Some features:

- Simulations can be serialized to *checkpoints* (freeze-dried and written to disk), which can be recovered from at any time, even to different Java platforms and new MASON visualization toolkits.- MASON can be set up to be *guaranteed duplicatable*, meaning that the same simulation parameters will produce the same results regardless of platform.- Libraries are provided for visualizing in 2D and in 3D (using Java3D), to manipulate the model graphically, to take screenshots, and to generate movies (using Java Media Framework).- While the visualization toolkits are fairly large, the core simulation model is intentionally very small, fast, and easy to understand.

MASON is open-source software licensed under the Academic Free License, version 3.0. See the LICENSE file for more information.

- Unpacking and Running MASON- MASON Documentation and Tutorials- Compiling MASON

## Unpacking and Running MASON

The distribution uncompresses into the mason directory. MASON is designed to be run both **with** or **without** certain add-on features which we predict you will want to add on. In most cases, MASON is most easily recompiled with all the add-on libraries installed.

### Quick Start (no add-ons)

In the same **mason** there is a directory
called **start**. Inside this directory there are scripts to fire up MASON.

|  |  |  |  |  |  |  |  |
| --- | --- | --- | --- | --- | --- | --- | --- |
| Windows: double-click on **mason.bat**| OS X: double-click on **mason.1.3.1.command** [for fastest 2D demos but no 3D demos]| (or) OS X: double-click on **mason.command** [for 3D demos]| Linux/X11: exec **mason.sh** | | | | | | | |

Pick an application from the list which appears and have fun!

### Add-On Java3D

To use MASON's 3D toolkit (or view its 3D demo apps), **install Sun's Java3D framework.** Get the version "for the JDK" rather than "for the JRE". We very strongly suggest that Windows users install the OpenGL version. Linux users can get Java3D here. Java3D for MacOS X only runs under MacOS X 10.3.x or later with Java 1.4.2, and can be downloaded at Apple's Download Page.

### Add-On Charting

To do charting, you'll need to install two libraries:

1. jFreeChart. The **jfreechart-1.0.1.jar** and **jcommon-1.0.0.jar** files are located in the **lib** directory of the jFreeChart distribution. Move them to the **mason** directory.- iText. Move the **itext-1.4.jar** file to the **mason** directory.

Alternatively you can download the library collection on the MASON web page, which contains these libraries, and move them into MASON from there.

### Add-On Movie Generation

To make Quicktime movies, you have to install Sun's Java Media Framework.

- MacOS X users should install the "Cross-platform Java" version, and place the **jmf.jar** file directly into the **mason** directory.- Windows and Linux users may install the custom versions of JMF Sun provides for them. But if those versions are frustrating you, you may follow the same instructions as the OS X users (which is simpler).

Alternatively you can download the library collection on the MASON web page, which contains this library, and move it into MASON from there.

### Prettier OS X Experience

- Java's color chooser is poor. You might try the "quaqua-colorchooser-only.jar" file in the Quaqua look and feel distribution.

Alternatively you can download the library collection on the MASON web page, which contains this library, and move it into MASON from there.

## MASON Documentation and Tutorials

### New Users

Start with Tutorial 0, which is a tour through various features of MASON's GUI code, and doesn't require much knowledge of Java.

### Beginning Coders

When you're ready to start coding, go through our seven tutorials located in the mason/sim/app directory. Each has an early HTML file walking through the tutorial, plus completed tutorial class files. Walking through these tutorials will help in learning to code with MASON more than anything else, we think.

If you're the kind of person who likes UML charts (or a bad approximation thereof), take a look at our Simulator Layout diagram, last updated a few versions ago and still pretty accurate.

Last but hardly least, we predict that the Class Documentation will prove useful to you. There is also an overview of the basic MASON classes.

### Advanced Coders

After the Tutorials, you should check out the How-Tos for answers to common things (parallelizing MASON, building an applet, etc.)

The Notes contain vital stuff that any MASON hacker should know.

If you're interested in grokking the internal scenegraphs of the Java3D code (which admittedly can get hairy), we have provided you with a graphical depiction of the important scenegraph structures.

Last, the **app** directory contains a number of example programs illustrating various MASON programs (and of varying quality!).

### Lost?

Hop on the MASON mailing list and post a question! Humans are often the best documentation of all. See the MASON website for instructions.

## Compiling MASON

MASON compilation presumes you are experienced with setting up CLASSPATHs (Windows example, OS X, Linux, etc.), running javac or jikes, and using a Makefile (if you're running Linux or MacOS X).

Here's what needs to go into the CLASSPATH:

1. The **mason** directory itself- All **jar** files located inside the **mason** directory, specifically the jar files for JFreeChart and JCommon, iText, and JMF.

If you're planning on using jikes, you'll also need to set up the JIKESPATH to include everything in the CLASSPATH, plus the various jar files of your java installation. See the jikes documentation for more information.

### Compiling From Eclipse

On this page Steve Lytinen and Steve Railsback have written a nice tutorial for using MASON in Eclipse.

### Compiling from the OS X / Linux Makefile

Make sure your CLASSPATH is set up right. Then:

cd mason; make 3d

...will compile all the files. Try make help to see more options.

If you've not installed Java3D and just want to play with the 2D stuff, try:

cd mason; make

If you get an error about **jikes** not installed, you can modify the Makefile's JAVAC variable to use the **javac** compiler instead. Or if you'd like to try jikes (it's much faster), install it from IBM's site.

### Compiling from the Windows DOS prompt

Make sure your CLASSPATH is set up right. Then try this:

cd mason  
     javac \*/\*.java \*/\*/\*.java \*/\*/\*/\*.java \*/\*/\*/\*/\*.java

### Running an App from the Command Line

Every GUI app class ends with the extension "WithUI.java". Assuming your CLASSPATH is set up properly, you can run any of them directly from the command line. For example:

java sim.app.heatbugs.HeatBugsWithUI

You can also run MASON proper from the command line like this:

java sim.display.Console

In certain situations you may wish to run with more memory. First, certain Java3D applications (notably Particles3D) require a lot of RAM because Java3D is a memory hog. Second, if you're running in Windows or Linux and wish to use the faster stretched image technique for drawing grids of rectangles (see the Options window -- the wrench icon ), increasing RAM will help performance. You can do this by including the -Xmx option in Java. For example, to run MASON directly using 200 megs of RAM:

java -Xmx200M sim.display.Console

MacOS X presents a special gotcha. The 2D MASON code runs much faster on MacOS X when using Java 1.3.1, as 1.4.1 is not presently hardware accelerated on the Mac. But the 3D MASON code only runs on 1.4.1. So if you're doing a MASON 3D app you must run 1.4.1 on the Mac; but if you're doing a MASON 2D app, you'll probably be much happier running 1.3.1. Sorry about that!

If you've upgraded to the latest Java on the Mac, you're running 1.4.1 or later. You can make a given Terminal window use 1.3.1 instead of 1.4.1 java with the command:

(In tcsh)  
     alias java /System/Library/Frameworks/JavaVM.framework/Versions/1.3.1/Home/bin/java  
(In bash)  
     alias java='/System/Library/Frameworks/JavaVM.framework/Versions/1.3.1/Home/bin/java'
