# Supplementary material for: Out of the net: An agent-based model to study human movements influence on local-scale malaria transmission
Source: PLoS One. 2018 Mar 6;13(3):e0193493. doi: 10.1371/journal.pone.0193493 (PMC5839546; doi:10.1371/journal.pone.0193493)
Supplement: S2 File — (ZIP) [file pone.0193493.s002.zip › S2/docs/Java3D.pdf]

What a typical SimplePortrayal3D.getModel() returns

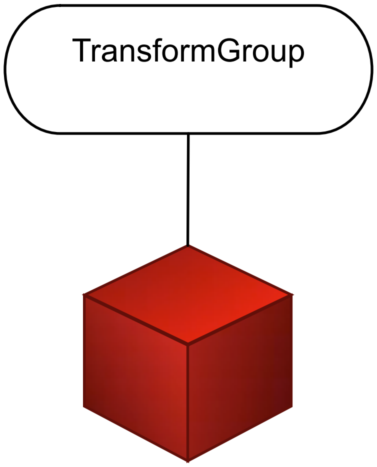

Passed to you via getModel(...), or if null, you have to make one. Owned by the parent (typically a FieldPortrayal3D, or a wrapper like TransformedPortrayal3D or CircledPortrayal3d or LabelledPortrayal3D) and used to translate the SimplePortrayal3D as necessary — don't fool with it except to hang stuff off of it.

The scenegraph which represents your object. Your SimplePortrayal3D can make it anything appropriate. Make it pickable if you want the object inspectable by the user — try SimplePortrayal3D.setPickableFlags()

What TransformedPortrayal.getModel() returns

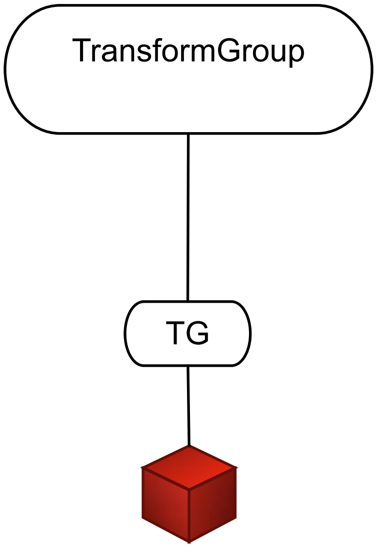

As usual, this transform group shouldn't be played with — it's for the parent's use at its discretion.

The TransformedPortrayal3D uses the underlying SimplePortrayal3D's transform group to transform the model as appropriate.

What CircledPortrayal3D.getModel() returns

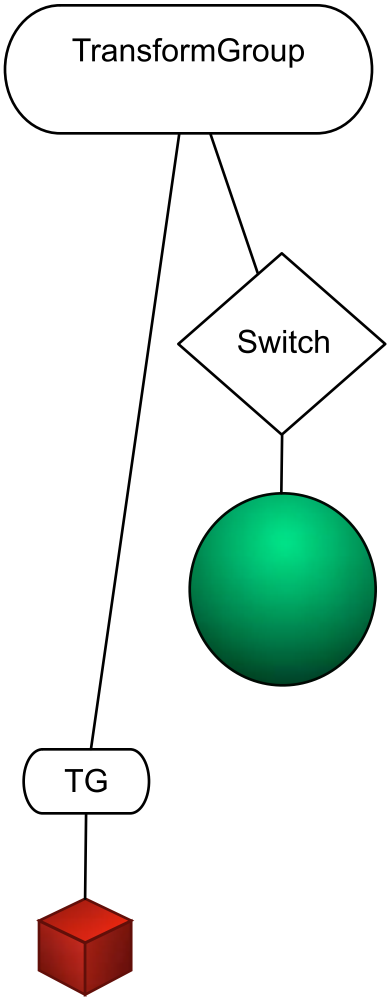

Passed to you via getModel(...), or if null, you have to make one. Owned by the Display3D and used to translate the FieldPortrayal as necessary — don't fool with it except to hang stuff off of it

When a CircledPortrayal2D provides its model, it provides a TransformGroup on which it has hung a Switch and a semitransparent Sphere3D. The Switch turns the Sphere3D on and off.

Here's the Sphere3D.

The CircledPortrayal2D also hangs off of its TransformGroup the model provided by the SimplePortrayal3D you had given it. The subsidiary TransformGroup is not modified.

What LabelledPortrayal3D.getModel() returns

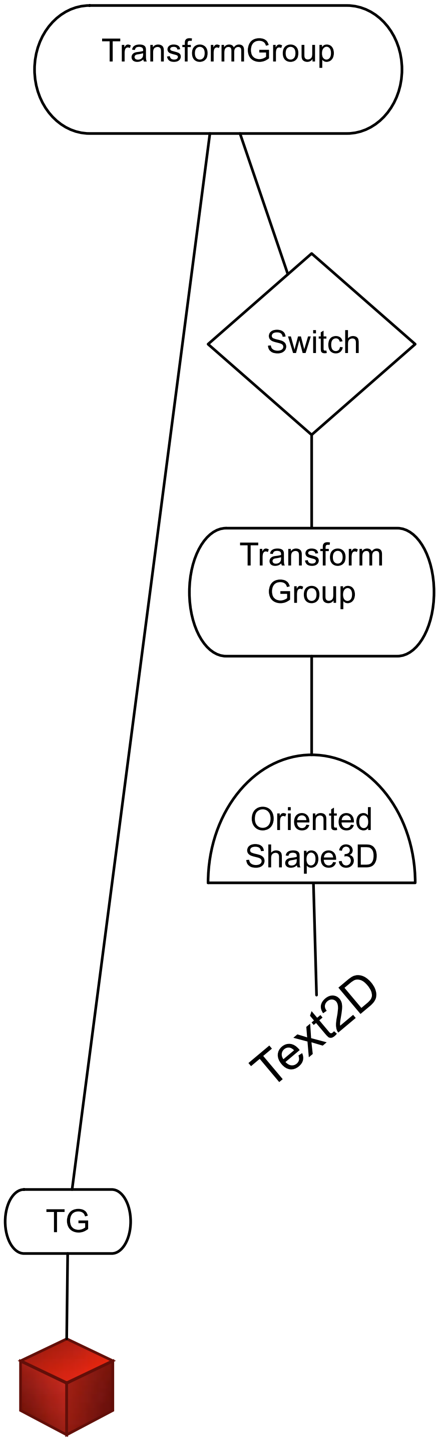

Passed to you via getModel(...), or if null, you have to make one. Owned by the Display3D and used to translate the FieldPortrayal as necessary — don't fool with it except to hang stuff off of it

Like CircledPortrayal3D, LabelledPortrayal3D also uses a switch to turn the label on and off

LabelledPortrayal3D also uses a TransformGroup to shift the label relative to the object. This lets you offset in an (x,y,z) direction.

We then orient the label so that it is always facing the user.

We use a Text2D to make the actual label rather than a Text3D because it is much more efficient. But bugs in the Windows version of Java3D can result in incorrect text size. In the LabelledPortrayal3D comments we have the code to use Text3D if you like.

The subsidiary model is treated just as it is in CircledPortrayal3D.

What a typical FieldPortrayal3D.getModel() returns

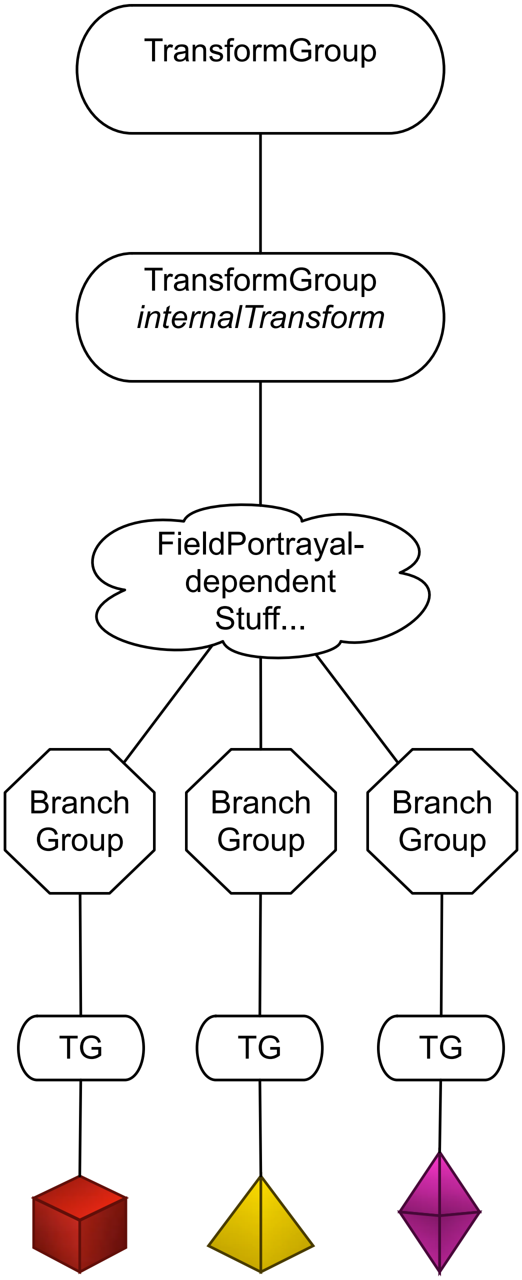

Passed to you via getModel(...), or if null, you have to make one. Owned by the Display3D and used to translate the FieldPortrayal as necessary — don't fool with it except to hang stuff off of it

You can modify this transform to your heart's content

FieldPortrayals may have different internal structures at this point

The TransformGroup-rooted scenegraphs for the objects in the field, as provided by each SimplePortrayal3D's getModel(...) method, get hung somewhere down here, usually themselves rooted by a BranchGroup. The BranchGroup allows the FieldPortrayal3D to delete or add the models as the objects come and go in the field. The TransformGroups are used to transform the scenegraph for each object to move it to the right location in the field.

Typically the field's objects, or other references to them, are stored in the user data of the BranchGroups so when the model is selected the FieldPortrayal knows what object it represents.

What ValueGrid2DPortrayal3D.getModel() returns

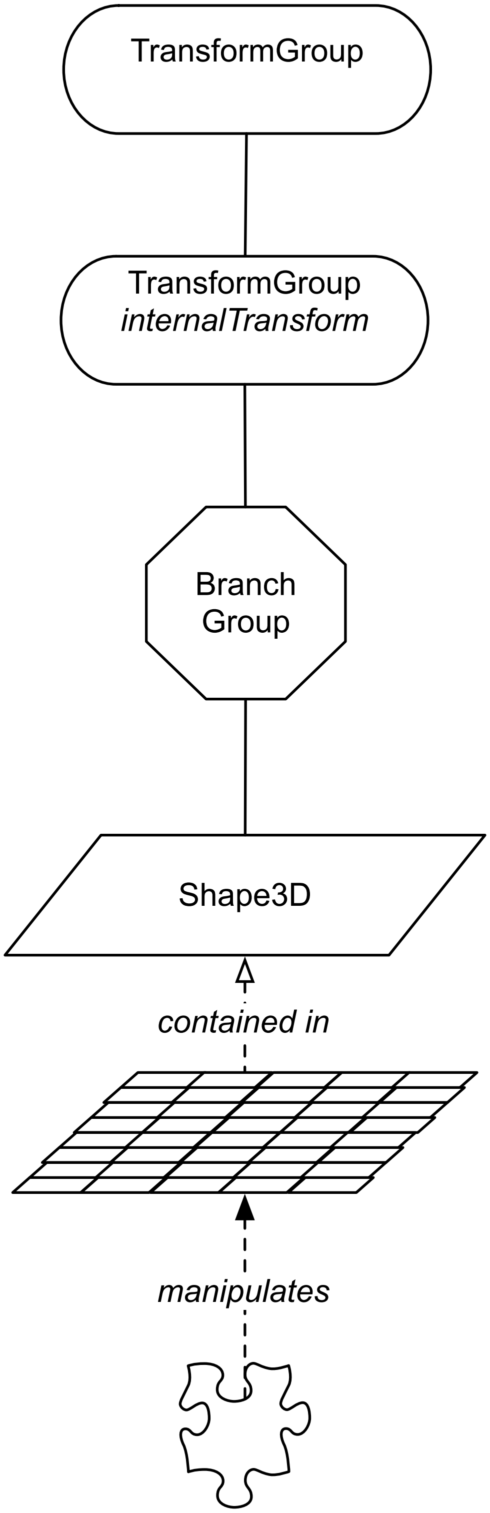

Passed to you via getModel(...), or if null, you have to make one. Owned by the Display3D and used to translate the FieldPortrayal as necessary — don't fool with it except to hang stuff off of it

You can modify this transform to your heart's content

The Shape3D representing the 2-Dimensional Value field is rooted by a BranchGroup so it can be removed if the user changes the dimensions of the underlying field.

The Shape3D which warps to represent the underlying Value field.

The GeometryArray for the Shape3D. This may be a QuadArray or a TriangleStripArray

This is the QuadPortrayal responsible for warping the individual points in the GeometryArray as directed by the ValueGrid2DPortrayal3D. QuadPortrayals can be either MeshPortrayals, which raise points on the GeometryArray in response to value grid values, or TilePortrayals, which raise whole rectangles on the GeometryArray in response to the values.

What EdgePortrayal3D.getModel() returns

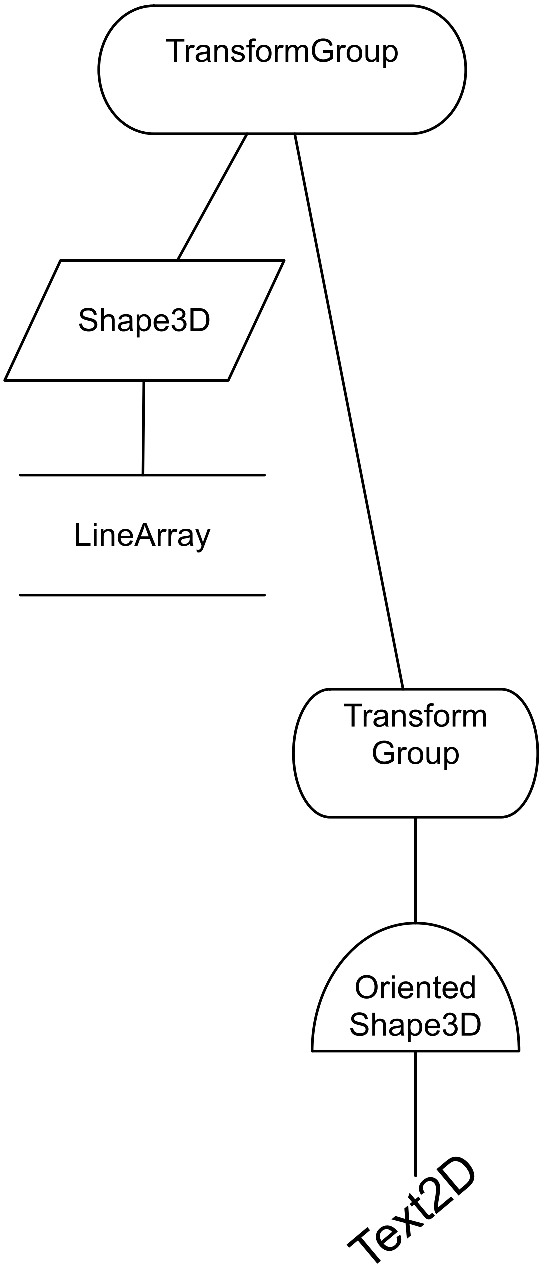

Passed to you via getModel(...), or if null, you have to make one. Owned by the Display3D and used to translate the FieldPortrayal as necessary — don't fool with it except to hang stuff off of it

The Shape3D representing the line which draws the edge

The line data for the above Shape3D

EdgePortrayal may optionally also have a label. If so, this TransformGroup holds the label and shifts it to just off the center of the edge.

We orient the label so it is always facing the user.

We use a Text2D to make the actual label rather than a Text3D because it is much more efficient. But bugs in the Windows version of Java3D can result in incorrect text size. In the LabelledPortrayal3D comments we have the code to use Text3D if you like.

Public scenegraph members of Display3D's CapturingCanvas3D

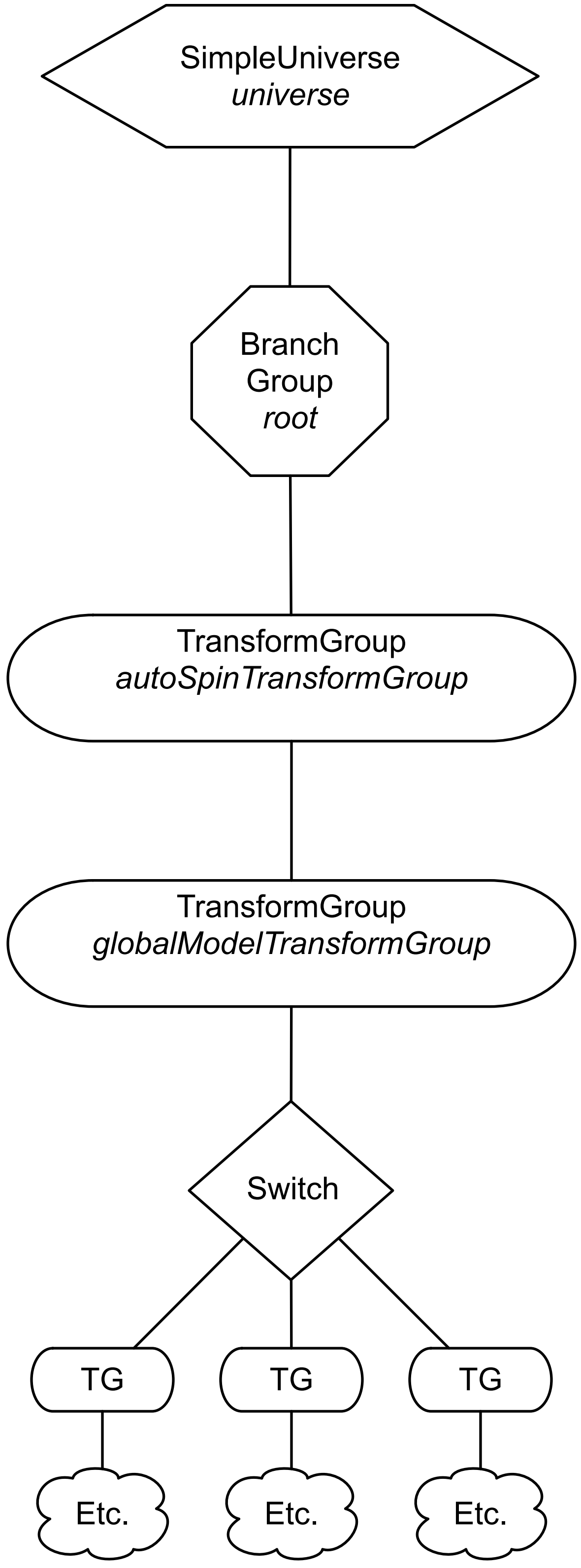

The universe

The top-level BranchGroup for the universe
Hang extra things off of here that you don't want spun by the auto-spinner.

Things hung off of here will get spun by the auto-spinner only

This internal transform is provided for you to transform the entire model as you see fit using Display3D's transformation methods.

Responsible for turning on and off the various fields in the Display3D

The various FieldPortrayal3Ds' models returned by createModel(...) It is here that the TransformGroups are transformed to move the FieldPortrayal3Ds to various locations relative to one another and size them relative to one another, etc. You can in theory add SimplePortrayals here as well.
