# Supplementary material for: Out of the net: An agent-based model to study human movements influence on local-scale malaria transmission
Source: PLoS One. 2018 Mar 6;13(3):e0193493. doi: 10.1371/journal.pone.0193493 (PMC5839546; doi:10.1371/journal.pone.0193493)
Supplement: S2 File — (ZIP) [file pone.0193493.s002.zip › S2/docs/notes.html]

MASON Notes


# Notes

1. Checkpointing- Communicating Between the Model Thread and the Visualization Thread- Using MASON's Multi-threaded Model Facilities- strictfp and Duplication- MacOS X Notes- Windows Notes- X Windows Notes- What You Should Know About Jikes and HotSpot

## Checkpointing

Checkpointing is done using Java's serialization facility. The checkpoint file is a standard Java serialized file, but has been gzipped. Only model objects are checkpointed out, not visualization objects.

If you want to checkpoint out on one platform and read in on another platform, your classes must all have the same **serialversionUIDs**. Different compilers will provide different serialversionUIDs to non-static inner classes (including anonymous classes) and the outer classes they are nested within. If you've got inner classes in your model code, you must provide your own hard-coded serialversionUID. See Tutorial3 for an extended discussion of this. None of this matters if you're writing and reading on the same platform (and with the same compiler).

Some Java classes cannot be checkpointed: for example, various Graphics2D classes. This presents a problem if you rely on them in your model. You may need to make custom serializations for your classes which use these classes. See the MavDemo model code for an example (particularly the Obstacle.java file).

## Communicating Between the Model Thread and the Visualization Thread

MASON's visualization system runs the model underneath in a separate thread.
This can create race conditions for visualization tools. Here are some
synchronization rules for you to follow.

**How it works.**
The Console first calls start(), then creates the underlying play thread.
After the thread dies, the Console calls finish(). Thus code in the start()
and finish() methods are always executed in the plain Swing event thread.

The model thread does the following:

1. Stop if it's been asked to. Else....- Step the GUIState. This in turn locks on the schedule, steps
     GUIState's scheduleImmediate(BEFORE) stuff, steps the schedule,
     steps GUIState's scheduleImmediate(AFTER) stuff, then unlocks the schedule.
     Inside the locks, the stepping causes the model to update, and Display2Ds to:
     - Issue an asynchronous repaint() request if we're on MacOS X- Otherwise draw immediately to the screen- Set the time, rate, and number of steps. This causes asynchronous requests to update the text field.- Stop if it's been asked to. Else...- Block on the event queue using SwingUtilities.invokeAndWait(*a do-nothing Runnable*). This trick causes the model thread to wait until all the Event Queue's repaints and events have gone through. The model thread should be interrupted from this block only if being requested to stop. At any rate, note that at this point in time the model thread no longer is holding onto the schedule lock. Thus if you lock on the schedule in an event in the event queue, you'll block until this point in time, then you'll go through.- Stop if it's been asked to. Else...- Sleep a little if it's been asked to. The model thread can be interrupted from this.- If the simulation has completed (but the thread wasn't stopped by the user), or we're at a requested breakpoint, stop thre thread. Else...- Go to #1.

Also, items stepped in the GUIState's
"extreme" queues are done so while holding onto the schedule lock.

Thus locking on the schedule is one way to know that you're in a safe situation with regard to the underlying model thread (it's waiting for you). Additionally, you should be aware that repaints() can *occur at any time*.

Most event loop and repaint things should be done by acquiring the schedule lock before getting information from the model. Remember to make your locks as brief as possible.

If that's so, then how do we *stop* the thread from the pause or stop button? Because the play thread blocks ont he event queue at step #5, consider the situation of pausing or stopping
it. The play thread has blocked. The user pressed the stop button. We try
to join() the play thread. Now the event loop has blocked on the play thread
and the play thread is blocking on the event loop. Deadlock! To deal with
this, we repeatedly interrupt() (with a 50ms spin-wait) the play thread until
it responds with an "okay, okay, I'm dying, sheesh" signal, and then we join()
it.

**General Locking Rules**

- Your model should never do anything which could block forever or you
  risk deadlock.- If you need to send information to a Swing Widget FROM anything scheduled
    in the GUIState or in the Schedule, you should do so asynchronously, using
    SwingUtilities.invokeLater(). There's no clean way to send a synchronous
    blocking message without risking deadlock.- If you need to send information FROM a Swing Widget TO the model, or
      read model data FROM a Swing Widget method, you can do so either
      asynchronously, synchronously, or as-a-single-thread.
      - asynchronously: load up a Steppable with copies of the information
        you wish to send to the model. Schedule the Steppable
        as a scheduleImmediate(...) in the GUIState.- synchronously: synchronize on state.schedule, then do your code.- as-a-single-thread: put your code inside a Runnable and pass it into
            the Controller's doChangeCode(...) method. Note that this
            is an expensive method: it pauses the model thread, runs
            your code, then unpauses the model thread. Do this only
            for code that's not in a tight loop: for example it's
            appropriate for code responding to a user pressing -Return-
            in a text field.

Your other option is to just bag it and risk the race condition. If you're
just setting a boolean value or an integer or a float, you're probably safe
here. If you're setting a double or a long, or anything bigger (an array?
class?), you're unsafe.


## Using MASON's Multi-threaded Model Facilities

MASON provides two opportunities for parallel threading at the model level: parallel Sequences and asynchronous Steppables.

A **ParallelSequence** is a Steppable which holds a set of subsidiary Steppables, and which can be scheduled on the Schedule. Unlike other Sequences which iterate through these Steppables in series, the ParallelSequence calls all of its subsidiary Steppables in parallel, each in its own thread, then waits until they have completed before it returns. The HeatBugs example code shows one approach to parallelizing the model using a ParallelSequence, resulting in nearly a two-times speedup on a dual-processor machine. ParallelSequences can be serialized.

An **AsynchronousSteppable** is a Steppable which, when stepped, runs in its own thread. Such Steppables may run in an infinite loop in their own thread forever until Stopped either by a later action in the Schedule or by the end of the simulation; or they may fire off a separate thread to do a one-shot asynchronous task in the background and then quit. AsynchronousSteppables can be serialized.

There are important differences between these two parallel models. ParallelSequences perform their actions in sync with the schedule: the ParallelSequence is stepped, it spawns its threads and lets them run, it waits for them to complete, and then it returns from its step. Thus all the actions of a ParallelSequence are done in one timestep and the schedule waits for them to finish. But an AsynchronousSteppable runs in the background, even while the schedule is being paused, and has no notion of "time" with regard to the schedule at all.

As usual, you need to be very careful when using multi-threaded code. ParallelSequences merely need to be written such that each separate Steppable in the sequence doesn't step on other steppables' toes. AsynchronousSteppables are much more fraught with peril -- when modifying or reading from the model, they need to lock on the Schedule to make sure that *neither* the Swing event thread *nor* the underlying model thread are reading/writing the model as well. That goes for the random number generator as well.

AsynchronousSteppables are new to MASON and are certain to have a number of bugs. If you synchronize on the Schedule in an AsynchronousSteppable, and the model thread is trying to call your stop method, we might have a race condition. Our cursory glance doesn't show any obvious race condition we've overlooked in the code, but one never knows.

Both ParallelSequences and (especially) AsynchronousSteppables may break guarantees of **duplication** (see below) because things do not operate in sync any more.

## strictfp and Duplication

MASON is designed to make it as easy as possible for you to have *duplicatable results*,
meaning that they will run the same way on MASON regardless of platform. However,
because of the different implementations of operations with floating point
numbers (such as +, -, /, \*, %), Java operations with doubles and singles
are not guaranteed to return identical results on different platforms,
unless you take the following steps:

1. In all classes which use floating-point math, add the "strictfp" keyword
   to the class header.- Replace all references of Math.*foo*() with StrictMath.*foo*()

This is *supposed* to guarantee identical results; though some platforms
still can get into trouble because they violate the spec. But it's as close as you can get.

Note that strictfp and StrictMath are slower than plain floating-point math. So
we've tried to make it possible for you to choose either to use them or to not use them (they're turned off by default). If you want platform-independent duplication, the following classes in the core of the simcore library should use the
strictfp keyword declaration:

```
sim.field.continuous.Continuous2D
sim.field.continuous.Continuous3D
sim.field.grid.DoubleGrid2D
sim.field.grid.DoubleGrid3D
sim.field.grid.IntGrid2D
sim.field.grid.IntGrid3D
```

Additionally, the following classes should be set to use StrictMath class methods rather than Math class methods:

```
sim.field.grid.DoubleGrid2D
sim.field.grid.DoubleGrid3D
ec.util.MersenneTwisterFast
```

Last, you'll need to add the "strictfp" keyword to the front of all classes in your model; usually
this means your SimState and Steppable subclasses. Anonymous classes
can't be strictfp: you'll need to move them to declared classes instead
if they contain floating point math operations and you want them to be strictfp.

## MacOS X Notes

### Java3D Only Runs on MacOS X Panther (10.3.x)

This means you can't presently run MASON's 3D package on MacOS X 10.2.

### MASON does not run on MacOS 9

MacOS 9 does not support Swing or Java2D.

### Should I use Java 1.4.1 or Java 1.3.1 on MacOS X?

If you're doing 2D drawing, by all means, use Java 1.3.1. It's much faster at drawing than 1.4.1 on the Mac (it's hardware accelerated). If you're doing 3D drawing, you have no choice but to use 1.4.1. Due to 1.3.1 bugs, if you're making a movie, you *definitely* want to use 1.4.1. And if you're only running command-line MASON with no graphics, 1.4.1 is significantly faster. If you've installed 1.4.1, you can still change a given terminal window to treat the java command as Java 1.3.1 rather than 1.4.1 like this:

alias java /System/Library/Frameworks/JavaVM.framework/Versions/1.3.1/Home/bin/java
  
  

### How to do fast 2D MacOS X Graphics

MacOS X is slow at jumping from window to window to draw stuff, so you want to minimize this as much as possible:

- Prevent the Time and Rate fields on the Console from updating themselves. To do so, click on one of them and they will change to say "Hidden" instead.- Close all inspectors and Empty the inspector list.- Click on the 2D Display so that it's in front of the other windows and is active.

### How do I get the SDK for MacOS X?

The SDK, plus the jikes is part of the Developer Tools; to get the latest release, register and log into Apple's Apple Developer Connection. It's free. To get an even more recent version of jikes, you can go here.

### Known MacOS X Platform-Specific Bugs

1. (1.3.1 only) After a few frames of QuickTime (or writing a few snapshots out),
   further such frames will not draw any text. See for example Tutorial5.
   This appears to be due to the Java VM smashing the stack when it compiles
   bytecode to machine code.- (1.3.1 only) The layers menu (the icon in the top-left corner of 2D displays) is flakey. If you select an item in the menu via dragging, it won't get selected and you'll get a spurious "ArrayIndexOutOfBoundsException", due to bugs in Apple's internal menu handling. You have to select items by *clicking* on them.- (1.3.1 only) Due to other Apple menu bugs, switching to or from the Console may produce spurious, harmless "java.lang.StackOverflowError".- (1.4.1 only) Multi-line pop-up lists are drawn with a single line. See for example
         the dialog which pops up when starting a Quicktime movie.- (1.4.1 only) Various spurious warnings can show up in the terminal window. They're all nonsense.

## Windows Notes

### Known Windows Platform-Specific Bugs

1. A Display2D's backdrop is often not repainted on Windows.- Reponses to events are very slow when the model is running full-blast; some events may even be dropped. You can often help matters by lowering the model thread priority in the console, or adding a Delay in the console.- Java3D is flakey on some systems: it does flashing effects every once in a while; we suspect this is due to Java's garbage collection.

## X Windows Notes

### Known X Windows Platform-Specific Bugs

1. Sometimes MASON will hang when loading a 3D application: bugs in the Java3D library.- A Display2D's backdrop is sometimes not repainted properly.- Reponses to events are very slow when the model is running full-blast; some events may even be dropped. You can often help matters by lowering the model thread priority in the console, or adding a Delay in the console.

## What You Should Know About Jikes and HotSpot

By default the Makefile tries to use the jikes compiler rather than javac. That's easily changed -- just change the comments in the Makefile. jikes is much faster than javac but you may not have it on your platform (we suggest you download it though!).

Newer versions of jikes are particularly obnoxious about style warnings. You can turn these style warnings off with the --nowarn option.

### Increasing HotSpot's Memory

MASON's models don't use much memory: but Java3D sure does! Java3D is a memory hog extraordinaire and it's not hard to require 200 megabytes for a small 50x50x50 cluster of objects.

Unfortunately, Java's default heap sizes are in the 20 Megabyte range and it does not increase them dynamically. To manually increase your heap size to, say, 100 Megabytes, you can do:

```
java -Xmx100M -Xms100M ...
```

This is also useful for making your graphics run faster if you're using the Stretched Image method for drawing grids of rectangles, particularly in Linux, which garbage-collects a lot if you're using the Stretched Image method. The big thing you want to get rid of is a rapid rate of "Full" Garbage collections (ordinary collections are no big deal). You can see ordinary and "full" collections occurring with:

```
java -verbose:gc ...
```

If you'd like to profile your code, the easiest way is to run:

```
java -Xprof ...
```

This prints out a profiling statement for each thread after the thread terminates, showing the piggiest interpreted, compiled, and native functions.
