# Supplementary material for: Out of the net: An agent-based model to study human movements influence on local-scale malaria transmission
Source: PLoS One. 2018 Mar 6;13(3):e0193493. doi: 10.1371/journal.pone.0193493 (PMC5839546; doi:10.1371/journal.pone.0193493)
Supplement: S2 File — (ZIP) [file pone.0193493.s002.zip › S2/docs/overview.html]

MASON Class Overview


# MASON Class Overview

## The sim.util and ec.util Packages

This package contains classes whose function is not MASON-only. ec.util is not a subpackage of sim because it contains classes developed for ECJ, an evolutionary computation library provided by George Mason University's ECLab. The sim.util package contains classes developed in conjunction with MASON or converted from other packages gathered online.

**MersenneTwisterFast**   (ec.util) MASON's default random number generator. MersenneTwisterFast is a highly efficient, unsynchronized implementation of the Mersenne Twister RNG. It needs to be in ec.util for MASON to work properly with ECJ.

**Bag, IntBag, DoubleBag**   Bag is an extensible Object array with public access. Significantly (3-4 times) faster than ArrayList or Vector. Bag is a subclass of Collection. IntBag and DoubleBag are extensible arrays of integers and doubles.

**Double2D, Double3D, Int2D, Int3D**   Immutable x,y or x,y,z tuples. These classes are used extensively to describe locations of objects. We use them rather than java.awt.Point etc. because you need an immutable class to work properly with a hashtable. Our tuples have better hash algorithms as well.

**MutableDouble2D, MutableDouble3D, MutableInt2D, MutableInt3D**   Mutable versions of Double2D, Double3D, Int2D, and Int3D. Instances of these classes hash to the same value as their immutable cousins, and so can be used to look up objects stored by their cousins in hash tables (though you wouldn't use a Mutable version as a key *store* a value in a hash table).

**Interval**   A class for specifying full-open intervals between two double values or two long values.

**MutableDouble**   A trivial holder for a single, modifiable double value.

**DoubleDimension2D**   A Dimension2D subclass which holds doubles (for some reason Java doesn't have this).

**Valuable**   A simple interface for objects which return a double value.

**Properties, SimpleProperties, CollectionProperties, Proxiable**   A small collection of classes for examining and manipulating the Java Beans properties of objects. *foo* is a Java Beans property if there exists a getFoo() or isFoo() method (and an optional setFoo(*val*) method). You get the properties from an object with Properties.getProperties(...). Objects can be declared Proxiable, meaning that they offer to the Properties object a proxy to examine instead of the objects themselves.

**WordWrap**   A small class for doing word wrapping either by number of columns or number of pixels (given font information).

**Heap**   A simple, fast binary heap class, with Comparables as keys.

**Utilities**   A few static utility methods.

### sim.util.media

Utilities for manipulating media.

**MovieEncoder**   A class for generating Quicktime movies from BufferedImages using Sun's Java Media Framework (JMF). Written based largely on code from Sun, portions of which are subject to a mild BSD-like License from Sun.

**PngEncoder**   A class for generating PNG images. Used because Sun's image framework is not available on all platforms (and the encoder works just fine). Distributed with permission from David Eisenberg, under the Artistic License.

**PDFEncoder**   A class for generating PDF files from Graphics2D objects, using iText.

### sim.util.media.chart

Utilities for creating Charts using JFreeChart.

**ChartGenerator**   An abstract JPanel which displays a chart using the JFreeChart library.

**SeriesAttributes**   An abstract LabelledList which contains the drawing attributes of a given data series displayed in a ChartGenerator.

**HistogramChartGenerator**   A ChartGenerator which displays data series as histograms.

**HistogramSeriesAttributes**   A SeriesAttributes designed to provide drawing attributes for histogram data series.

**TimeSeriesChartGenerator**   A ChartGenerator which displays data series as time series.

**TimeSeriesAttributes**   A SeriesAttributes designed to provide drawing attributes for time series.

### sim.util.gui

Graphical Interface Widgets.

**AbstractScrollable**   An abstract superclass with basic implementation of the javax.swing.Scrollable interface.

**LabelledList**   A simple class to make it easy to create a 2xN matrix of widgets (often the left column is labels for the right column).

**MovieMaker**   A GUI front-end for MovieEncoder. Relies on Java's reflection library to tentatively access MovieEncoder; if JMF is not installed on a machine, properly gives a graphical interface warning.

**NumberTextField**   (and various *PNG* images). A widget for entering, displaying, and filtering a numerical value.

**PropertyTextField**   A widget for entering, displaying, and filtering a string or boolean value.

**MiniHistogram**   A simple class for small histograms which fit nicely into the space the size of a text field.

**ColorMap**   An interface for mapping double or integer values to colors.

**SimpleColorMap**   A basic implementation of the ColorMap interface.

**ColorWell**   A widget for displaying and changing a Color.

**HTMLBrowser**   A very simple, almost trivial, HTML browser class, used by the Console.

**DialogToolTip**   A tool-tip implemented with a JDialog. This is used to provide tooltips even in Java3D, which does not have any facility for them.

## The sim.util.engine Package

Contains the main simulation superclass, the schedule, and utilities for scheduling.

**SimState**   The superclass that you extend to create your simulation model. Contains at least a Schedule and a MersenneTwisterFast.

**Schedule**   A discrete-event schedule, using heaps and arrays to perform arbitrary schedules and repeated schedules efficiently. Represents time in your model.

**Steppable**   Something that can be scheduled on the schedule to have its step() method called (to be "stepped").

**Stoppable**   When a Steppable is scheduled repeating, a Stoppable is returned, with a function called stop() to stop the repeating from continuing on.

**Sequence**   A Steppable which holds an array of Steppables. When stepped, steps each of the Steppables in turn.

**RandomSequence**   A Sequence which randomizes the order of its Steppables before stepping them.

**ParallelSequence**   A Sequence which steps all of its Steppables simultaneously, in different threads.

**MultiStep**   A Steppable which holds a subsidiary Steppable. Can either call step() N times for each MultiStep.step(), or will call step() once every N MultiStep.step() calls.

**WeakStep**   A Steppable which only weakly holds an object (letting it GC if desired).

**TentativeStep**   A Steppable which provides a Stoppable that can stop it before it is fired.

**Asynchronous**   A simple interface for creating asynchronous thread Steppable objects.

**AsynchronousSteppable**   A basic implementation of the Asynchronous interface.

**MakesSimState**   An interface for classes capable of creating SimState subclasses: largely used internally in SimState.

## The sim.field Package

Contains *fields*, which represent spatial or other neighborhoods. Zero or more fields represent space in your model.

**SparseField**   An abstract superclass of various *sparse fields*. A sparse field is one which uses hash tables rather than arrays to store a sparse number of objects spread over a large area.

### The sim.field.grid Package

Contains *grids*, which are discrete fields. Most grids have a fixed width and height. Grids can be treated toroidally, and 2D grids can be thought of as holding hexagons rather than squares.

**Grid2D, Grid3D**   Top-level interfaces for all grids. Specify basic grid functions, and toroidal and/or hexagonal conversion functions.

**AbstractGrid2D, AbstractGrid3D**   Abstract implementations of the Grid2D and Grid3D interfaces. Superclasses of all but the sparse grids (which subclass instead from SparseField).

**IntGrid2D, IntGrid3D**   Grids of integers. Literally covers for multidimensional Java integer arrays.

**DoubleGrid2D, DoubleGrid3D**   Grids of doubles. Literally covers for multidimensional Java double arrays.

**ObjectGrid2D, ObjectGrid3D**   Grids of Objects. Literally covers for multidimensional Java Object arrays.

**SparseGrid2D, SparseGrid3D**   Sparse grids of Objects. Allow objects to be located anywhere (rather than in a fixed width/height) if necessary, and for multiple Objects to be located at the same location. Implemented with a SparseField. Locations are specified with Int2D and Int3D.

### The sim.field.continuous Package

Contains *continuous fields* which relate Objects to locations defined as tuples of doubles.

**Continuous2D, Continuous3D**   Associate any number of Objects with 2D or 3D tuples of doubles as locations. Efficient neighborhood lookups are done through discretization of the space. Subclasses of SparseField. Locations are specified with Double2D and Double3D.

### The sim.field.network Package

Contains *networks* (directed graphs) which relate Objects to one another via binary relationships.

**Edge**   A directed relationship between two objects. Edges may be labelled with an "info" object (a weight, a name, etc.).

**Network**   Defines directed graphs and networks between arbitrary objects. Relationships are defined using Edge objects.

## The sim.display Package

This package holds the main GUI windows in MASON: the Console and Display2D windows. The Console maintains the simulation and runs the schedule. The Display2D holds various FieldPortrayal2D objects.

**Controller**   The abstract superclass of top-level controllers of a simulation.

**Console**   (and various *PNG* images, plus the *simulation.classes* file). An elaborate Controller which presents a window allowing the user to start and stop the simulation, change its parameters, and manage its various windows and inspectors.

**Display2D**   (and various *PNG* images). A window which allows the user to scroll, scale, take snapshots of, and make movies of various FieldPortrayal2Ds as they are updated during the course of the simulation run.

**GUIState**   The wrapper which allows graphical interface widgets to synchronize with and communicate with an underlying SimState. Packages implementing a graphical interface will need to subclass from this class.

**SimApplet**   A simple class for setting up MASON as an applet.

## The sim.portrayal Package

This package is the most elaborate of any of MASON's packages. It contains facilities for *portraying* (drawing, picking via a graphical interface, inspecting) fields and objects/ints/doubles stored in those fields. Fields are portrayed with *field portrayals* stored in the sim.portrayal.grid, sim.portrayal.continuous, and sim.portrayal.network packages. Objects stored within those fields are portrayed using *simple portrayals*: some useful ones are in the sim.portrayal.simple package.

**Portrayal**   The top-level interface for all portrayals.

**Portrayal2D**   A Portrayal subinterface. The top interface for all 2D portrayals.

**FieldPortrayal**   The abstract superclass of all Field Portrayals. Contains facilities for storing SimplePortrayals and relating them with objects in a Field.

**FieldPortrayal2D**   The abstract superclass of all 2D Field Portrayals.

**SimplePotrayal2D**   The abstract superclass of all 2D Simple Portrayals.

**Inspector**   The abstract superclass of GUI widgets responsible for allowing the user to view and manipulate the internal parameters of objects and fields.

**SimpleInspector**   A concrete implementation of Inspector which does so by letting the user have access to the Java Bean properties of objects. This is done using the Properties class and subclasses.

**DrawInfo2D**   When an object or field needs to be drawn, the scale, location, and clipping information is passed to its portrayal in this wrapper object.

**HexaDrawInfo2D**   A subclass of DrawInfo2D with additional information for Hexagons to draw themselves prettily.

**Oriented2D**   Objects adhering to this interface may be portrayed by sim.portrayal.simple.OrientedPortrayal2D.

**LocationWrapper**   Relates objects with locations in a field. Used to by fields to assist in the generation of inspectors

### The sim.portrayal.grid Package

Portrayals for grids.

**ValueGridPortrayal2D, HexaValueGridPortrayal2D, FastValueGridPortrayal2D, FastHexaValueGridPortrayal2D**   Portrayals for IntGrid2D and DoubleGrid2D. ValueGridPortrayal2D is flexible, while FastValueGridPortrayal2D is faster. HexaValueGridPortrayal2D portrays the fields as hexagonal fields. FastHexaValueGridPortrayal2D is faster (drawing rectangles rather than hexagons).

**ObjectGridPortrayal2D, HexaObjectGridPortrayal2D, FastObjectGridPortrayal2D**   Portrayals for ObjectGrid2D, portraying it in normal fashion or as a hexagonal grid.

**SparseGridPortrayal2D, HexaSparseGridPortrayal2D**   Portrayals for SparseGrid2D, portraying it in normal fashion or as a hexagonal grid.

**DrawPolicy**   Since multiple objects may hold the same polsition in a SparseGrid2D, its portrayals call this optional object to determine which objects to draw at positions and in which order.

### The sim.portrayal.continuous Package

Portrayals for continuous fields.

**ContinuousPortrayal2D**   Portrays a Continuous2D field.

### The sim.portrayal.network Package

Portrayals for networks.

**NetworkPortrayal2D**   Portrays the edges in a Network. The nodes in the field must be also stored in a SparseGrid2D or Continuous2D field, and portrayed with a SparseGridPortrayal2D or ContinuousPortrayal2D respectively.

**SpatialNetwork2D**   Holds the Network and either a SparseGrid2D or Continuous2D. Used by NetworkPortrayal2D to know where to draw edges (by querying the right field for the node locations).

**SimpleEdgePortrayal2D**   A basic SimplePortrayal2D for Edges.

**EdgeDrawInfo2D**   A subclass of DrawInfo2D which adds additional information for drawing Edges.

### The sim.portrayal.simple Package

Various SimplePortrayal2Ds for your enjoyment.

**RectanglePortrayal2D**   Portrays objects as rectangles (usually squares)

**ValuePortrayal2D**   Portrays values (from ValueGridPortrayal2D) as rectangles colored according to the value.

**OvalPortrayal2D**   Portrays objects as ovals (usually circles).

**ShapePortrayal2D**   Portrays objects as provided Java2D shapes.

**ImagePortrayal2D**   Portrays objects as a provided image.

**HexagonalPortrayal2D**   Portrays objects as true hexagons.

**LabelledPortrayal2D**   A wrapper for other 2D Portrayals which adds a text label to them.

**CircledPortrayal2D**   A wrapper for other 2D Portrayals which draws a big circle around them.

**OrientedPortrayal2D**   A wrapper for other 2D Portrayals which draws a line (or other shape) over them pointing in a given direction. The direction is provided by the underlying portrayed object, assuming that it adheres to Oriented2D.

**TransformedPortrayal2D**   A wrapper for other 2D Portrayals which draws transforms them according to a provided AffineTransform (rotates, translates, skews them, etc.).

### The sim.portrayal.inspector Package

Utility inspectors and plug-in property inspectors.

**TabbedInspector**   An inspector which can hold multiple subinspectors in a tabbed pane.

**PropertyInspector**   The abstract superclass of plug-in inspectors designed to inspect individual properties appearing in the primary inspectors of a simulation.

**StreamingPropertyInspector**   A PropertyInspector which can stream a property's values to disk, to a window, to standard out, etc.

**ChartingPropertyInspector**   An abstract PropertyInspector for creating inspectors which chart a property's values using JFreeChart.

**TimeSeriesChartingPropertyInspector**   A ChartingPropertyInspector which charts a property's values using a time series chart.

**HistogramChartingPropertyInspector**   A ChartingPropertyInspector which charts a property's values using a histogram. The property's values must be in the form of an array or other list.

## The sim.display3d Package

This package holds the GUI system for displaying elements in 3D using Java3D. Primarily, this is Display3D.

**Display3D**   A window which allows the user to scale, translate, rotate, zoom in and out, take snapshots of, and make movies of various Portrayal3Ds as they are updated during the course of a simulation run.

**CapturingCanvas3D**   A subclass of Java3D's Canvas3D class which enables Display3D to take snapshots and make movies.

**SelectionBehavior**   A Java3D behavior which enables Display3D to select and inspect objects.

**ToolTipBehavior**   A Java3D behavior which pops up tool tips.

## The sim.portrayal3d Package

This package is an extension of the sim.portrayal to portray objects in 3D space rather than 2D space. The system used is Java3D.

**Portrayal3D**   An interface which extends Portrayal to specify how objects are to be represented in 3D space.

**FieldPortrayal3D**   An abstract subclass of FieldPortrayal which adheres to the Portrayal3D interface. FieldPortrayal3D is the superclass of all Field Portrayals which draw objects in 3D space.

**SparseFieldPortrayal3D**   An abstract subclass of FieldPortrayal3D and the superclass of all 3D portrayals of 2D and 3D sparse fields.

**SimplePortrayal3D**   A concrete implementation of Portrayal3D for simple (non-field) portrayals in 3D. SimplePortrayal3D by default does not display anything. Various subclasses display objects in different ways.

### The sim.portrayal3d.continuous Package

3D Portrayals for continuous fields.

**ContinuousPortrayal3D**   Portrays both Continuous2D and Continuous3D fields in 3D.

### The sim.portrayal3d.network Package

3D Portrayals for networks.

**NetworkPortrayal2D**   Portrays the edges in a Network. The nodes in the field must be also stored in a SparseGrid3D or Continuous3D field, and portrayed with a SparseGridPortrayal3D or ContinuousPortrayal3D respectively.

**SpatialNetwork3D**   Holds the Network and either a SparseGrid3D or Continuous3D. Used by NetworkPortrayal3D to know where to draw edges (by querying the right field for the node locations).

**SimpleEdgePortrayal3D**   A basic SimplePortrayal3D for Edges.

### The sim.portrayal3d.grid Package

3D Portrayals for grids.

**SparseGridPortrayal3D**   Portrays both SparseGrid2D and SparseGrid3D fields in 3D.

**SparseGrid2DPortrayal3D**   Portrays SparseGrid2D fields in 3D by piling up multiple objects located at the same [x,y] location.

**ObjectGridPortrayal3D**   Portrays ObjectGrid2D and ObjectGrid3D fields.

**ValueGridPortrayal3D**   Portrays DoubleGrid2D, DoubleGrid3D, IntGrid2D, and IntGrid3D fields.

**ValueGrid2DPortrayal3D**   Portrays DoubleGrid2D and IntGrid2D fields in 3D by warping a mesh of squares.

**ValueGridCellInfo**   A class used by ValueGrid2DPortrayal3D to communicate information about underlying values to its mesh of squares.

### The sim.portrayal3d.grid.quad Package

Various ways a ValueGrid2DPortrayal3D may draw its underlying squares.

**QuadPortrayal**   An abstract superclass defining how rectangles are portrayed by the ValueGrid2DPortrayal3D.

**TilePortrayal**   A QuadPortrayal which portrays values as the squares themselves (the 'tiles').

**MeshPortrayal**   A QuadPortrayal which portrays values as the intersections of the squares.

### The sim.portrayal3d.simple Package

All manner of SimplePortrayals in 3D.

**ConePortrayal3D**   Portrays objects as cones.

**CubePortrayal3D**   Portrays objects as cubes.

**CylinderPortrayal3D**   Portrays objects as cylinders.

**SpherePortrayal3D**   Portrays objects as spheres.

**ImagePortrayal3D**   Portrays objects as images floating in 3D space.

**Shape3DPortrayal3D**   Portrays objects as Java3D Shape3D objects (provided by you).

**SharedGroupPortrayal3D**   Portrays objects as Java3D SharedGroup objects (provided by you).

**LightPortrayal3D**   Portrays objects as various kinds of Light objects.

**WireFrameBoxPortrayal3D**   Portrays objects as wireframe boxes.

**ValuePortrayal3D**   Portrays values (from ValueGridPortrayal3D) as cubes colored according to the value.

**LabelledPortrayal3D**   A wrapper for other SimplePortrayal3Ds which adds a label.

**TransformedPortrayal3D**   A wrapper for other SimplePortrayal3Ds which transforms them using a provided affine transform (translates, rotates, skews them, etc.).

**CircledPortrayal3D**   A wrapper for other SimplePortrayal3Ds which surrounds them with a large semitransparent sphere.

**Axes**   Draws axes. Primarily used internally by Display3D.

**Arrow**   Draws an arrow. Used to form Axes.
