# Supplementary material for: Out of the net: An agent-based model to study human movements influence on local-scale malaria transmission
Source: PLoS One. 2018 Mar 6;13(3):e0193493. doi: 10.1371/journal.pone.0193493 (PMC5839546; doi:10.1371/journal.pone.0193493)

Basic MASON Model
and its Relationship to GUI Controllers

A model consists of a Schedule, SimState, and MersenneTwisterFast, plus various agents scheduled in the schedule, plus various Fields containing objects the agents are free to manipulate.

The user can manipulate the schedule through the Console, and view and manipulate fields and objects through Display2D and Display3D

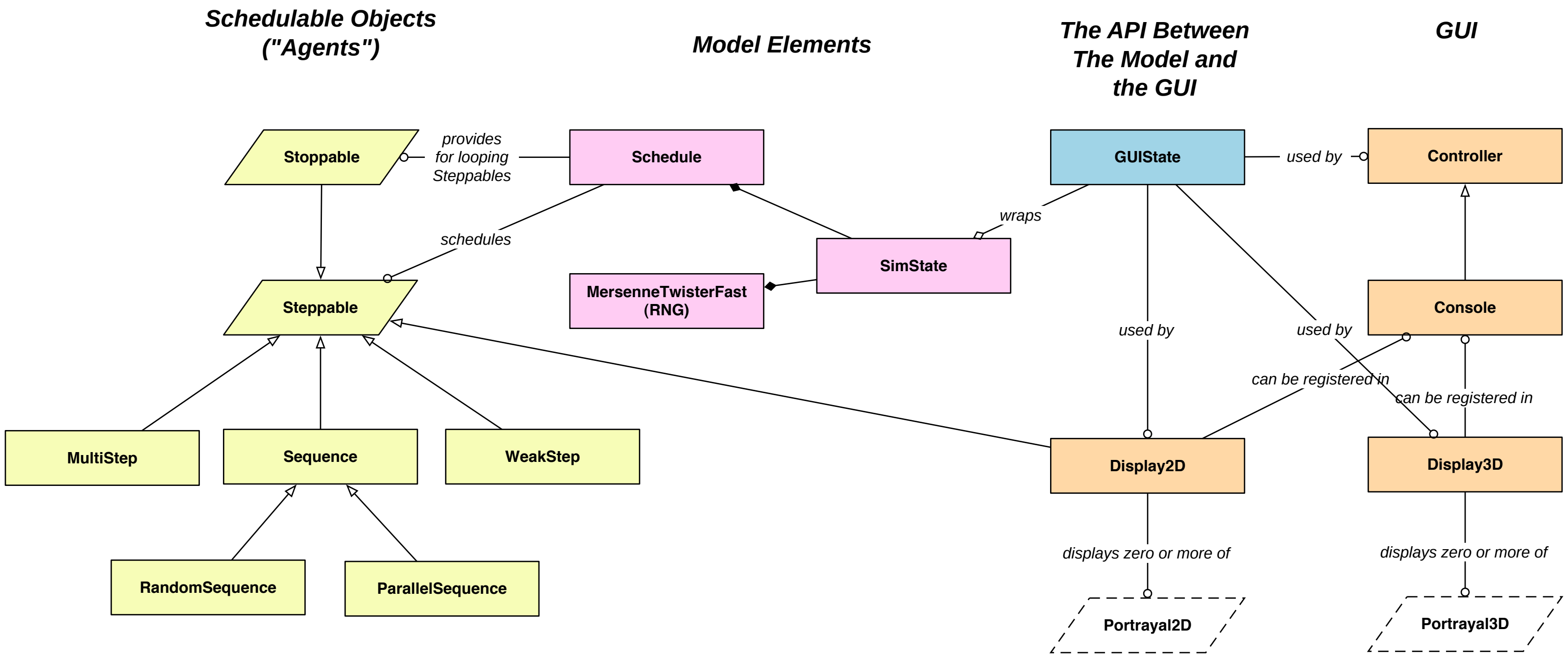

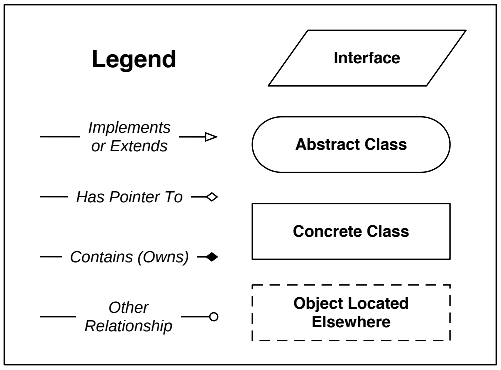

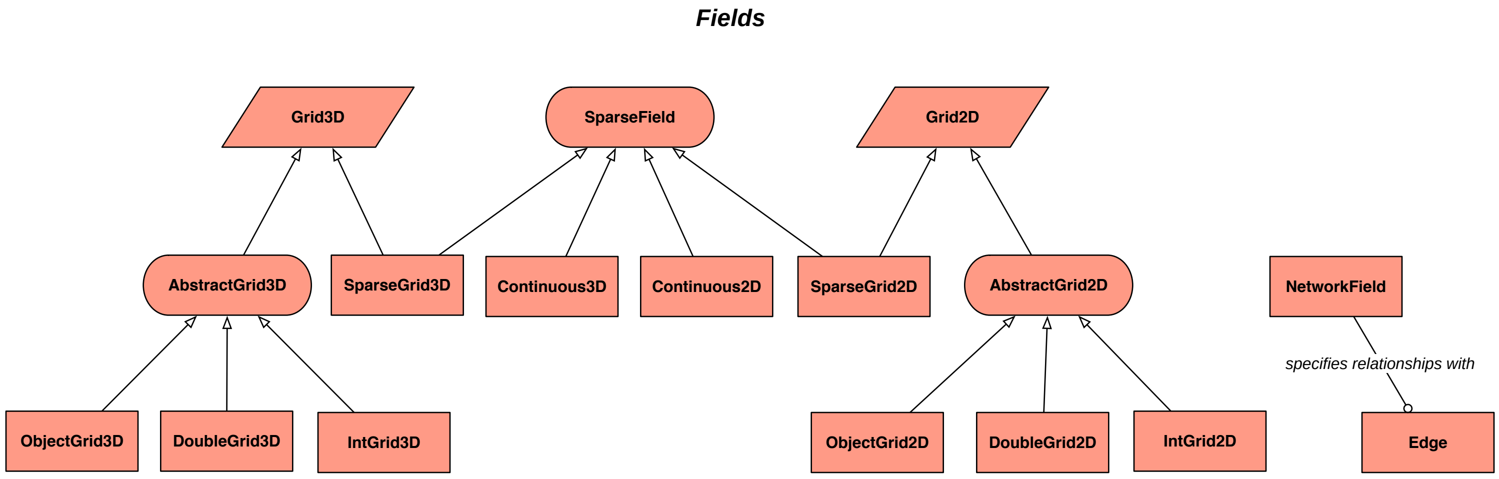

2D Field Portrayals

2D Field Portrayals draw Fields inside a Display2D

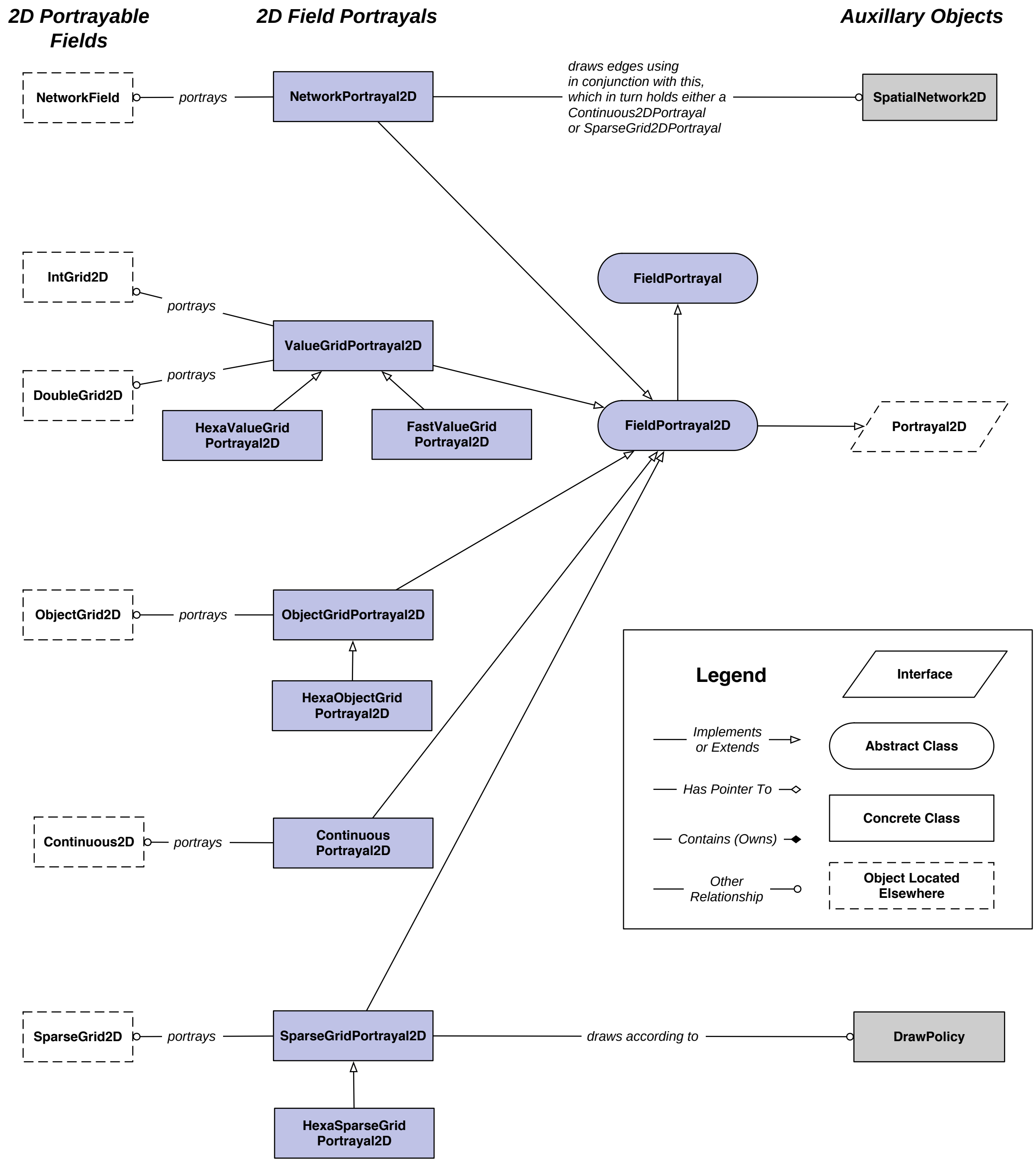

Top-Level Portrayal Facility
and 2D Simple Portrayals

**Portrayals draw and allow the user to inspect and/or manipulate objects.
FieldPortrayals draw/inspect/manipulate Fields, calling upon
SimplePortrayals to draw/inspect/manipulate the objects within those fields.**

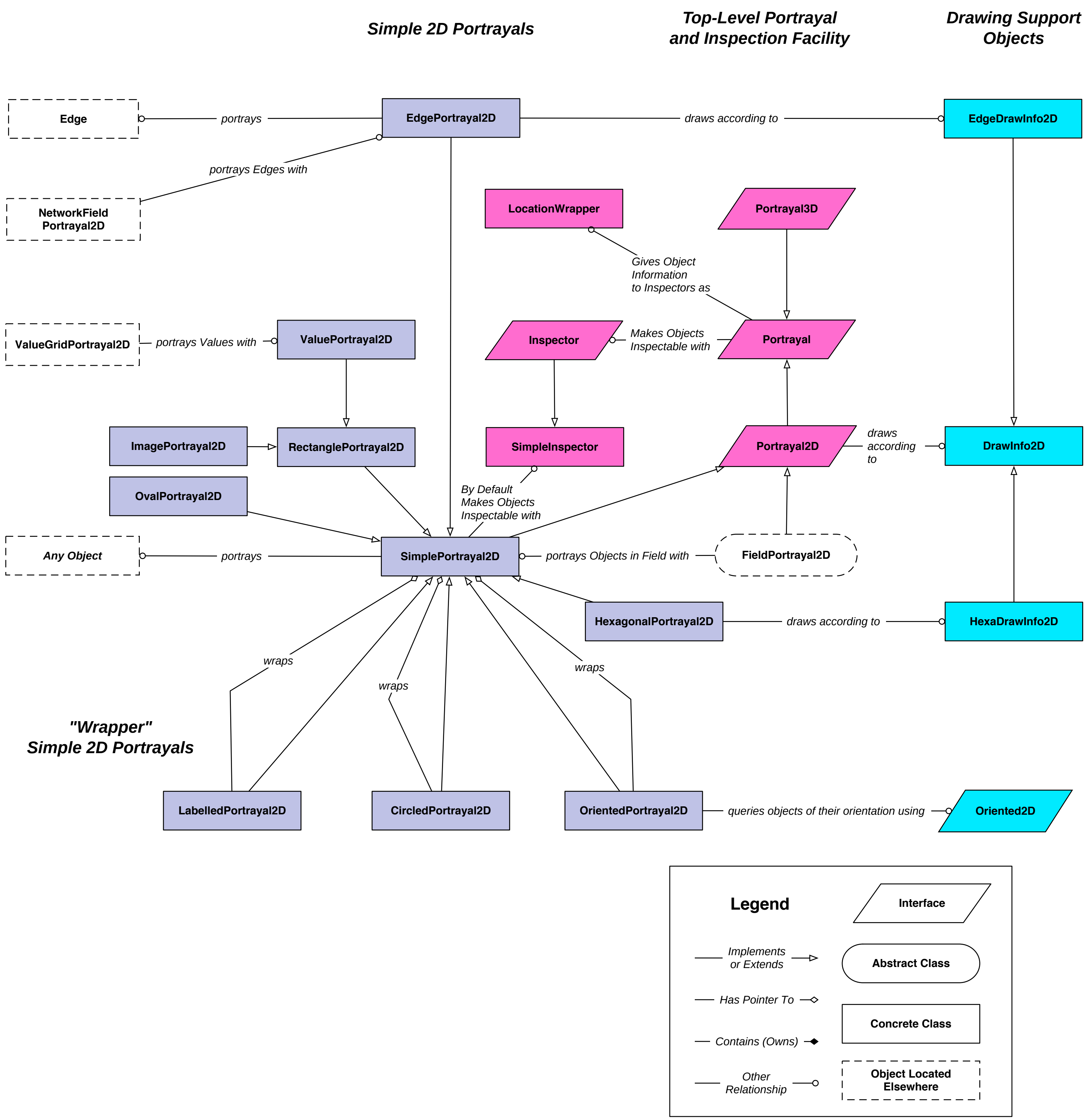

3D Field Portrayals

3D Field Portrayals draw Fields inside a Display3D

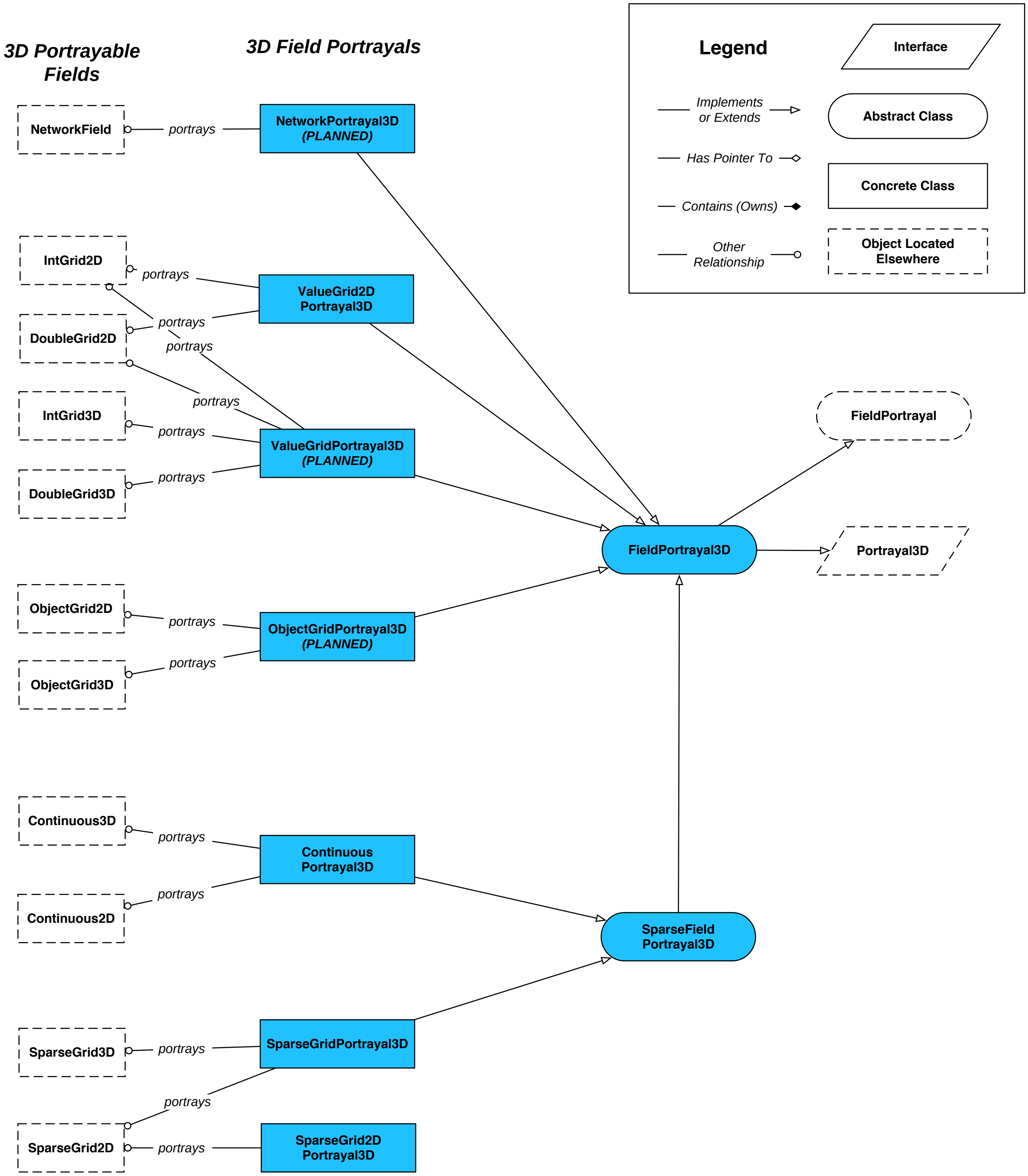

3D Simple Portrayals and QuadPortrayals

3D SimplePortrayals portray the objects within Fields, when called upon to do so by 3D FieldPortrayals. QuadPortrayals draw 2D values in 3D for the ValueGrid2DPortrayal3D FieldPortrayal.

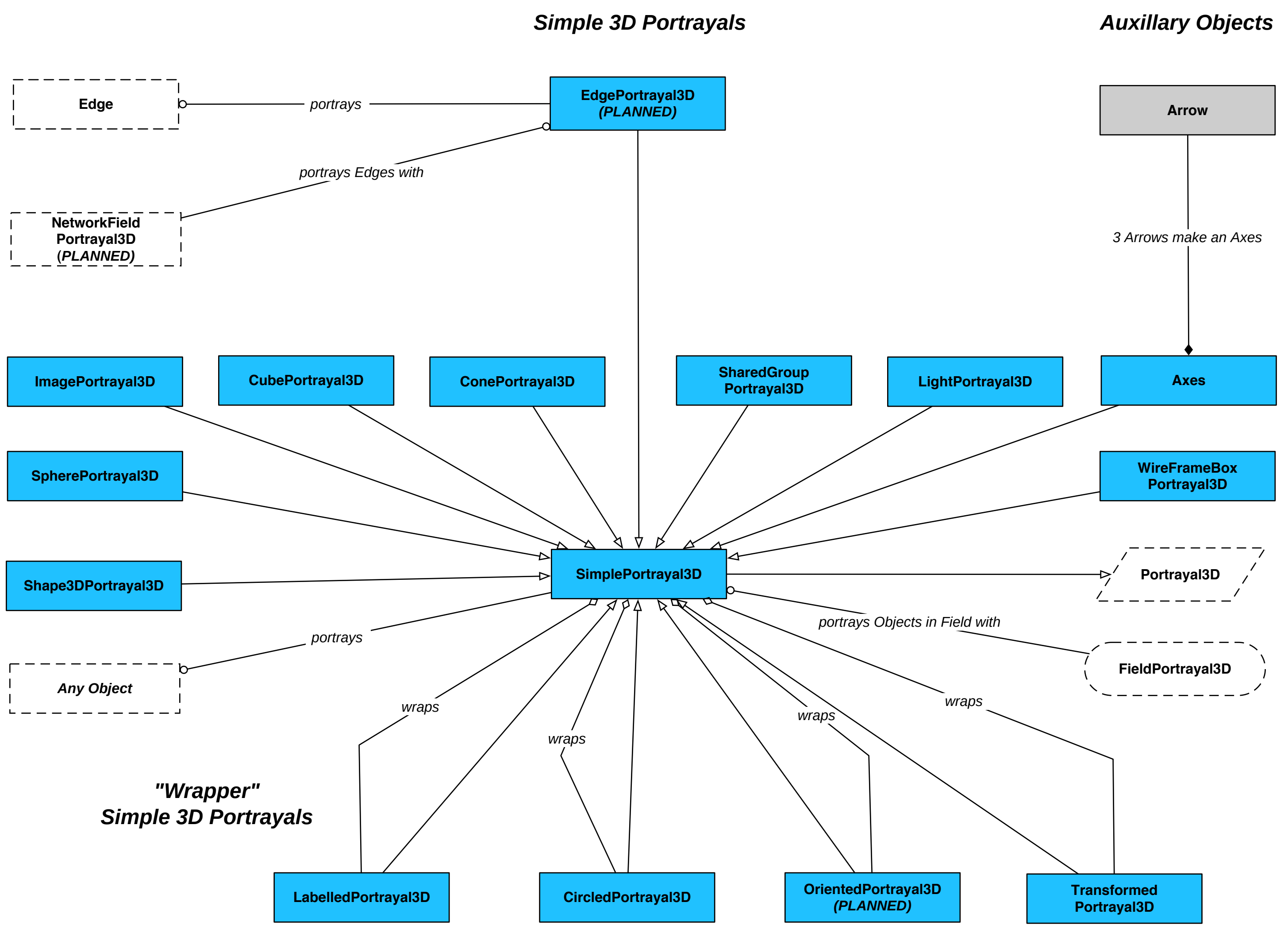

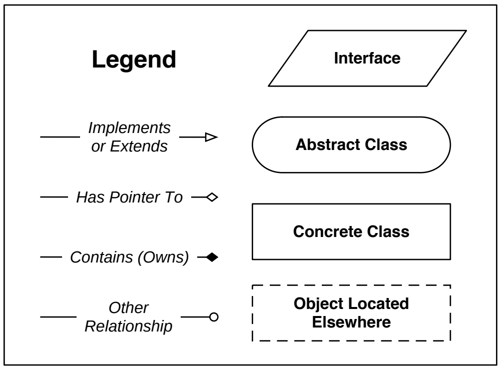

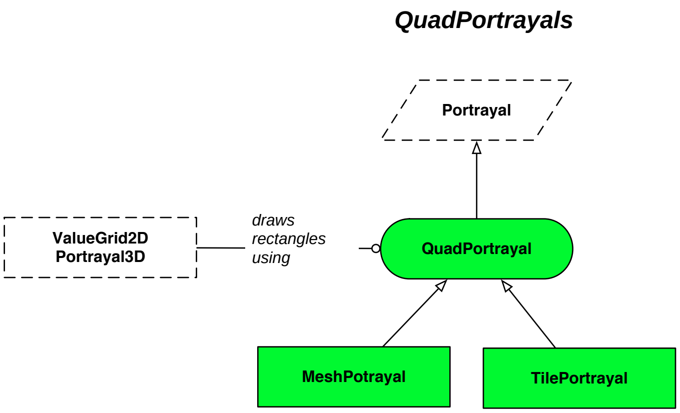

The Big Picture

The GUIState wraps the Simulation Model and separates it from the visualization and user manipulation facilities. The model can be completely broken off and run on its own (without GUIState or Visualization stuff), and can be serialized to/from disk.

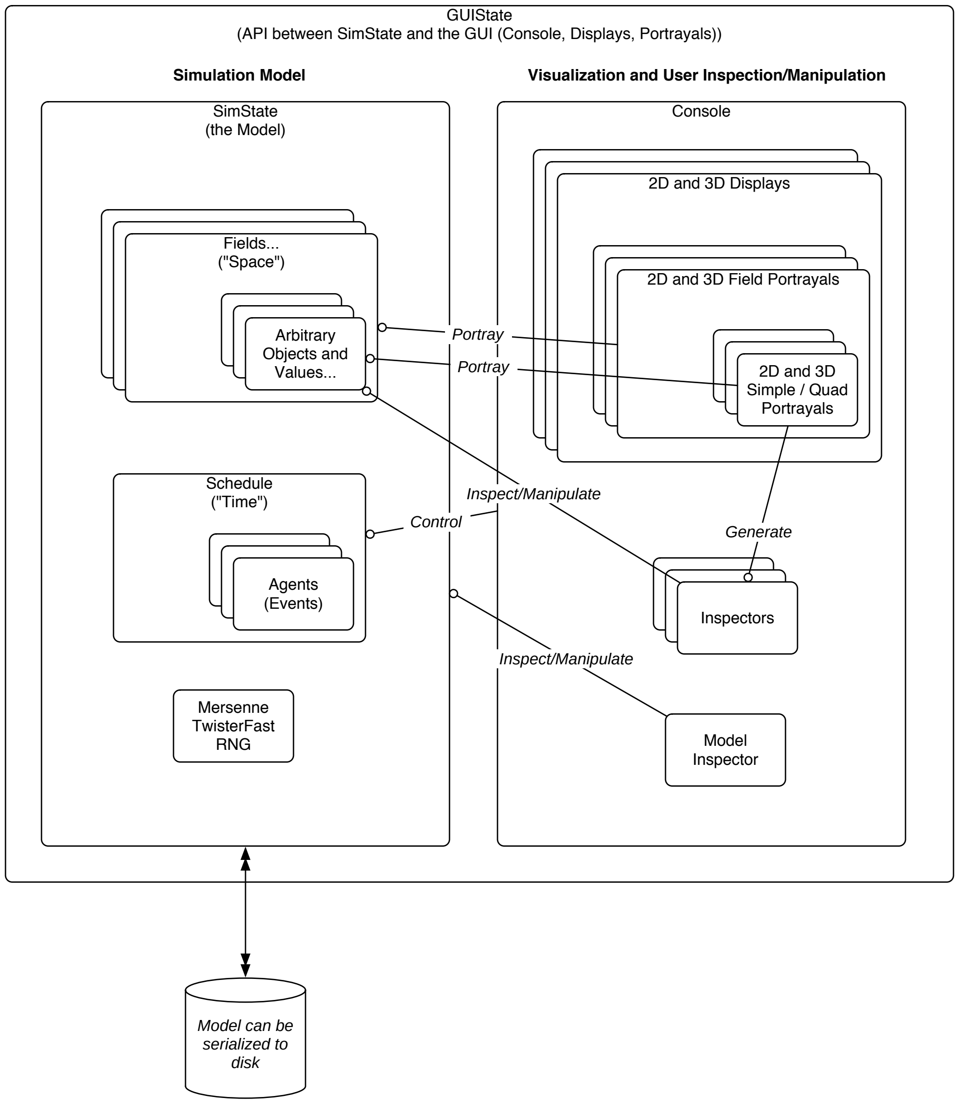

Supplement: S2 File — (ZIP) [file pone.0193493.s002.zip › S2/docs/SimulatorLayout.pdf]
