# Supplementary material for: Out of the net: An agent-based model to study human movements influence on local-scale malaria transmission
Source: PLoS One. 2018 Mar 6;13(3):e0193493. doi: 10.1371/journal.pone.0193493 (PMC5839546; doi:10.1371/journal.pone.0193493)
Supplement: S2 File — (ZIP) [file pone.0193493.s002.zip › S2/sim/app/AmaSim/index.html]

|  |  |
| --- | --- |
| AmaSimBy Francesco Pizzitutti 2014 | |

Malaria Simulation in the Amazon.
AmaSim is an individual based simulation of malaria spreading in the Amazon. It represents mosquito vectors and human hosts at an individual based level.

For more information write to Francesco Pizzitutti: francesco.pizzitutti@gmail.com.
