# Supplementary material for: Out of the net: An agent-based model to study human movements influence on local-scale malaria transmission
Source: PLoS One. 2018 Mar 6;13(3):e0193493. doi: 10.1371/journal.pone.0193493 (PMC5839546; doi:10.1371/journal.pone.0193493)
Supplement: S2 File — (ZIP) [file pone.0193493.s002.zip › S2/sim/display/SimApplet.html]

MASON Applet


The following applet loads the entire MASON toolkit so it may take a second. Please note:

- 3D demos cannot be run unless your computer has Java3D installed.- One or two 3D demos cannot be run as an applet period: they use too much memory.- Mac web browsers create some funkiness. Their Java is not hardware-accelerated and the 2D graphics is *very* slow. You'll have more luck running MASON directly as a Java 1.3.1 application. See the main web page for more information.- Mac web browsers except for Safari operate best when the Java 1.4.2 plugin is running.- Once a simulation is running, you can start another simulation under the File menu.- If you close a display, it is hidden -- you can get it back under the Displays tab in the console window. If you close the the console window, the simulation is quit.
