# Supplementary figures and images for: Out of the net: An agent-based model to study human movements influence on local-scale malaria transmission
Source: PLoS One. 2018 Mar 6;13(3):e0193493. doi: 10.1371/journal.pone.0193493 (PMC5839546; doi:10.1371/journal.pone.0193493)

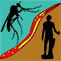

Supplement: S2 File — (ZIP) [file pone.0193493.s002.zip › S2/sim/app/AmaSim/icon.png]

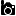

Supplement: S2 File — (ZIP) [file pone.0193493.s002.zip › S2/sim/display/Camera.png]

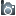

Supplement: S2 File — (ZIP) [file pone.0193493.s002.zip › S2/sim/display/CameraPressed.png]

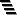

Supplement: S2 File — (ZIP) [file pone.0193493.s002.zip › S2/sim/display/Layers.png]

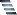

Supplement: S2 File — (ZIP) [file pone.0193493.s002.zip › S2/sim/display/LayersPressed.png]

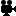

Supplement: S2 File — (ZIP) [file pone.0193493.s002.zip › S2/sim/display/MovieOff.png]

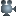

Supplement: S2 File — (ZIP) [file pone.0193493.s002.zip › S2/sim/display/MovieOffPressed.png]

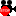

Supplement: S2 File — (ZIP) [file pone.0193493.s002.zip › S2/sim/display/MovieOn.png]

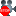

Supplement: S2 File — (ZIP) [file pone.0193493.s002.zip › S2/sim/display/MovieOnPressed.png]

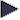

Supplement: S2 File — (ZIP) [file pone.0193493.s002.zip › S2/sim/display/NotPlaying.png]

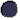

Supplement: S2 File — (ZIP) [file pone.0193493.s002.zip › S2/sim/display/NotRecording.png]

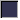

Supplement: S2 File — (ZIP) [file pone.0193493.s002.zip › S2/sim/display/NotStopped.png]

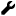

Supplement: S2 File — (ZIP) [file pone.0193493.s002.zip › S2/sim/display/Options.png]

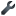

Supplement: S2 File — (ZIP) [file pone.0193493.s002.zip › S2/sim/display/OptionsPressed.png]

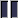

Supplement: S2 File — (ZIP) [file pone.0193493.s002.zip › S2/sim/display/PauseOff.png]

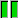

Supplement: S2 File — (ZIP) [file pone.0193493.s002.zip › S2/sim/display/PauseOn.png]

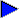

Supplement: S2 File — (ZIP) [file pone.0193493.s002.zip › S2/sim/display/Playing.png]

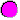

Supplement: S2 File — (ZIP) [file pone.0193493.s002.zip › S2/sim/display/Recording.png]

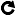

Supplement: S2 File — (ZIP) [file pone.0193493.s002.zip › S2/sim/display/Reload.png]

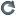

Supplement: S2 File — (ZIP) [file pone.0193493.s002.zip › S2/sim/display/ReloadPressed.png]

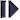

Supplement: S2 File — (ZIP) [file pone.0193493.s002.zip › S2/sim/display/StepOff.png]

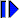

Supplement: S2 File — (ZIP) [file pone.0193493.s002.zip › S2/sim/display/StepOn.png]

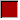

Supplement: S2 File — (ZIP) [file pone.0193493.s002.zip › S2/sim/display/Stopped.png]
